# New World species of the genus *Calliscelio* Ashmead (Hymenoptera, Platygastridae, Scelioninae)

**DOI:** 10.3897/zookeys.648.10935

**Published:** 2017-01-30

**Authors:** Hua-yan Chen, Lubomír Masner, Norman F. Johnson

**Affiliations:** 1 Department of Entomology, The Ohio State University, 1315 Kinnear Road, Columbus, Ohio 43212, U.S.A.; 2 Agriculture and Agri-Food Canada, K.W. Neatby Building, Ottawa, Ontario K1A 0C6, Canada; 3 Department of Evolution, Ecology and Organismal Biology, The Ohio State University, 1315 Kinnear Road, Columbus, Ohio 43212, U.S.A.

**Keywords:** Egg parasitoid, key, revision, Gryllidae

## Abstract

The genus *Calliscelio* Ashmead is presumed to be a diverse group of parasitoids of the eggs of crickets (Orthoptera: Gryllidae). A least one species has been found to be an important factor in depressing cricket pest populations. The New World species of *Calliscelio* are revised. Forty-two species are recognized, 3 are redescribed: *Calliscelio bisulcatus* (Kieffer), *Calliscelio laticinctus* Ashmead, *Calliscelio rubriclavus* (Ashmead), **comb. n.**; and 38 are described as new: *Calliscelio absconditum* Chen & Johnson, **sp. n.**, *Calliscelio absum* Chen & Johnson, **sp. n.**, *Calliscelio alcoa* Chen & Masner, **sp. n.**, *Calliscelio amadoi* Chen & Johnson, **sp. n.**, *Calliscelio armila* Chen & Masner, **sp. n.**, *Calliscelio bidens* Chen & Masner, **sp. n.**, *Calliscelio brachys* Chen & Johnson, **sp. n.**, *Calliscelio brevinotaulus* Chen & Johnson, **sp. n.**, *Calliscelio brevitas* Chen & Johnson, **sp. n.**, *Calliscelio carinigena* Chen & Johnson, **sp. n.**, *Calliscelio crater* Chen & Johnson, **sp. n.**, *Calliscelio crena* Chen & Johnson, **sp. n.**, *Calliscelio eboris* Chen & Johnson, **sp. n.**, *Calliscelio extenuatus* Chen & Johnson, **sp. n.**, *Calliscelio flavicauda* Chen & Johnson, **sp. n.**, *Calliscelio foveolatus* Chen & Johnson, **sp. n.**, *Calliscelio gatineau* Chen & Johnson, **sp. n.**, *Calliscelio glaber* Chen & Masner, **sp. n.**, *Calliscelio granulatus* Chen & Masner, **sp. n.**, *Calliscelio latifrons* Chen & Johnson, **sp. n.**, *Calliscelio levis* Chen & Johnson, **sp. n.**, *Calliscelio longius* Chen & Johnson, **sp. n.**, *Calliscelio magnificus* Chen & Masner, **sp. n.**, *Calliscelio migma* Chen & Johnson, **sp. n.**, *Calliscelio minutia* Chen & Johnson, **sp. n.**, *Calliscelio paraglaber* Chen & Johnson, **sp. n.**, *Calliscelio pararemigio* Chen & Masner, **sp. n.**, *Calliscelio prolixus* Chen & Johnson, **sp. n.**, *Calliscelio punctatifrons* Chen & Johnson, **sp. n.**, *Calliscelio remigio* Chen & Masner, **sp. n.**, *Calliscelio ruga* Chen & Johnson, **sp. n.**, *Calliscelio rugicoxa* Chen & Masner, **sp. n.**, *Calliscelio sfina* Chen & Johnson, **sp. n.**, *Calliscelio storea* Chen & Johnson, **sp. n.**, *Calliscelio suni* Chen & Johnson, **sp. n.**, *Calliscelio telum* Chen & Johnson, **sp. n.**, *Calliscelio torqueo* Chen & Johnson, **sp. n.**, *Calliscelio virga* Chen & Johnson, **sp. n.** Four species are treated as junior synonyms of *Calliscelio rubriclavus* (Ashmead): *Anteris nigriceps* Ashmead, **syn. n.**, *Caloteleia marlattii* Ashmead, **syn. n.**, *Caloteleia grenadensis* Ashmead, **syn. n.**, and *Macroteleia ruskini* Girault, **syn. n.**

## Introduction

The genus *Calliscelio* Ashmead was first erected for a single species, *Calliscelio laticinctus* Ashmead, from the Lesser Antilles (Ashmead 1893). For nearly 80 years it was considered to be a valid genus although it remained narrowly defined. Kieffer (1926), for example, included only two species in his monograph on world Scelionidae (= Platygastridae *sensu* Sharkey 2007). Masner (1972) treated *Calliscelio* as a junior synonym of *Calotelea* Westwood. Shortly thereafter, though, Masner (1976) rejected this hypothesis, treated *Calliscelio* as valid, and also significantly expanded the extent of the genus. Kieffer had described seven other genera over the years, all of which Masner (1976) treated as junior synonyms of *Calliscelio* (see generic synonymy below). Recently, in a survey of external morphology across the superfamily Platygastroidea, Talamas et al. (2016) found that the prioniform sensillum on the mandible is synapomorphy for the genus *Calliscelio* and treated *Crama* Galloway, *Lispoteleia* Galloway, *Xentor* Masner and Johnson, and *Yunkara* Galloway as junior synonyms of *Calliscelio*.

The current concept of *Calliscelio* is a relatively large genus in the subfamily Scelioninae, comprising 77 known species (including 3 fossil species; Johnson 2016). It is a worldwide genus, with species found in all six major biogeographic regions. Interestingly, at least two species, i.e., *Calliscelio rubriclavus* (Ashmead) and *Calliscelio elegans* (Perkins), have extraordinarily broad geographic distributions possibly due to increased globalization of human commerce (Masner et al. 2009). This phenomenon leads to the question: from which part of the world did these species originate? Also, although *Calliscelio* was originally erected based on species in the New World, only 6 species were reported from this region to date, suggesting that more species remain to be discovered. Thus, a comprehensive revision of species of *Calliscelio* based on extensive sampling from the New World is needed to provide a more accurate understanding of the diversity and interrelationships among species in the genus.

Even though *Calliscelio* is a diverse, common, and widespread genus, the available biological data are extremely scanty. The only host record is for *Calliscelio teleogrylli* Hill, which was reported to attack the eggs of *Teleogryllus commodus* (Walker) (Orthoptera: Gryllidae), a widespread pest in pastures in the Pacific region (Hill 1983). By comparison with *Calliscelio teleogrylli*, Masner et al. (2009) suggested that *Calliscelio elegans* may attack the eggs of one or more species of Gryllidae associated with sugar cane.

The goal of this work is to reevaluate the known species of *Calliscelio* from the New World, expand the biogeographic data associated with these species, and to describe new species. The contributions of the authors are as follows. H-y Chen and N.F. Johnson: character definition, generic concept development, species concept development, imaging, key development, manuscript preparation; L. Masner: character definition, generic concept development, species concept development. The authors of the new species are indicated in the heading of each description.

## Materials and methods

This work is based upon specimens in the following collections, with abbreviations used in the text: AMNH, American Museum of Natural History, New York, NY; ANIC, Australian National Insect Collection, Canberra, Australia; BMNH, The Natural History Museum, London, UK; BPBM, Bernice P. Bishop Museum, Honolulu, HI; CAS, California Academy of Sciences, San Francisco, CA; CMNH, Carnegie Museum of Natural History, Pittsburgh, PA; CNCI, Canadian National Collection of Insects, Ottawa, Canada; FSCA, Florida State Collection of Arthropods, Gainesville, FL; IAVH, Instituto Alexander von Humboldt, Villa de Leyva, Columbia; MNHNPY, Museo Nacional de Historia Natural del Paraguay, San Lorenzo; INHS, Illinois Natural History Survey, Urbana, IL; MAIC, M.A. Ivie Private Collection, Bozeman, MT; MEMU, Mississippi State University, Mississippi State, MS; MHNG, Muséum d'Histoire Naturelle, Geneva, Switzerland; MNHN, Muséum National d'Histoire Naturelle, Paris, France; MPEG, Museu Paraense Emílio Goeldi, Belém, PA, Brazil; MZLU, Lund Museum of Zoology, Lund University, Lund, Sweden; MZSP, Museu de Zoologia da Universidade de São Paulo Sao, Brazil; NMNH, National Museum of Natural History, Washington, DC; OSUC, C.A. Triplehorn Insect Collection, Ohio State University, Columbus, OH; UCDC, R.M. Bohart Museum of Entomology, University of California, Davis, CA; UCFC, The Stuart M. Fullerton Collection of Arthropods at the University of Central Florida, Orlando, FL; UCMC, University of Colorado, Boulder, CO; UCRC, University of California, Riverside, CA; USNM, National Museum of Natural History, Washington, DC.

Abbreviations and morphological terms used in text: A1, A2...A12: antennomere 1, 2…12; claval formula: distribution of the large, multiporous basiconic sensilla on the underside of apical antennomeres of the female, with the segment interval specified followed by the number of sensilla per segment (Bin 1981); EH: eye height, length of compound eye measured parallel to dorsoventral midline of head; IOS: interocular space, minimal distance on frons between compound eyes; OD: ocellar diameter, greatest width of ocellus; OOL: ocular ocellar line, shortest distance from inner orbit and outer margin of posterior ocellus (Masner 1980); T1, T2, ... T7: metasomal tergite 1, 2, ... 7; S1, S2, … S7: metasomal sternite 1, 2, … 7. Morphological terminology otherwise generally follows Masner (1980) and Mikó et al. (2007).

Morphological terms used in this work are as in the Hymenoptera Anatomy Ontology (Yoder et al. 2010) (Appendix 1). Identifiers (URIs) in the format HAO_XXXXXXX represent concepts in the HAO and are provided to enable readers to confirm their understanding of the concepts being referenced. To find out more about a given concept, including additional images, notes, references and other metadata, use the identifier as a search term at http://glossary.hymao.org or use the identifier as a web-link.

In the Material Examined section, the specimens studied are recorded in an abbreviated format, using unique identifiers (numbers prefixed with “OSUC”, “CASENT”, “UCFC”, “CMNH”, “MHNG”) for the individual specimens. The label data for all specimens have been georeferenced and recorded in the Hymenoptera On-Line database, and details on the data associated with these specimens can be accessed at the following link, hol.osu.edu, and entering the identifier in the form (note the space between the acronym and the number). The electronic version of the paper contains hyperlinks to external resources. Insofar as possible, the external information conforms to standards developed and maintained through the organization Biodiversity Information Standards (Taxonomic Database Working Group). All new species have been prospectively registered with ZooBank (Polaszek et al. 2005, http://www.zoobank.org), and other taxonomic names, where appropriate, have been retrospectively registered.

Data associated with the genus *Calliscelio* can be accessed at hol.osu.edu/index.html?id=461. The generic and species descriptions were generated by an xBio:D platform application, vSysLab (vsyslab.osu.edu), designed to facilitate the production of a taxon by character data matrices, and to integrate those data with the existing taxonomic, bibliographic and specimen-level database. Data may be exported in both text format and as input files for other applications. The text output for descriptions is in the format of “Character: Character state (s)”. Polymorphic characters are indicated by semicolon-separated character states.

Images and measurements were made using Combine ZP and AutoMontage extended-focus software, using JVC KY-F75U digital camera, Leica Z16 APOA microscope, and 1X objective lens. Images were post-processed with Abobe Photoshop CS3 Extended. A standard set of images is provided for each species: dorsal habitus, lateral habitus, dorsal and lateral views of the head and mesosoma, and anterior view of head. The individual images are archived in Specimage (specimage.osu.edu), the image database at The Ohio State University.

## Taxonomy

### Calliscelio

Taxon classificationAnimaliaHymenopteraPlatygastridae

Ashmead

Calliscelio Ashmead, 1893: 209, 218 (original description. Type: Calliscelio laticinctus Ashmead, by monotypy and original designation, keyed); Ashmead 1894: 216 (keyed); Dalla Torre 1898: 501 (catalog of species); Ashmead 1900: 327 (list of species of West Indies); Ashmead 1903: 91 (keyed); Brues 1908: 27, 28, 33 (diagnosis, list of species, keyed); Kieffer 1908: 122 (keyed); Kieffer 1910b: 66 (keyed); Kieffer 1913: 232 (description); Kieffer 1926: 273, 499 (description, keyed, key to species); Muesebeck and Walkley 1956: 338 (citation of type species); Baltazar 1966: 185 (cataloged, catalog of species of the Philippines); Masner 1972: 839 (junior synonym of Calotelea Westwood); Masner 1976: 34, 36, 43 (description; key to Calliscelio Ashmead, Paridris Kieffer, Oethecoctonus Ashmead, and Probaryconus Kieffer; key to Calotelea Westwood and Calliscelio Ashmead); Mani and Sharma 1982: 178 (description); Galloway and Austin 1984: 8, 27, 28 (description, list of species described from Australia, keyed); Kozlov and Kononova 1985: 19 (description, key to species of the Palearctic); Kozlov and Kononova 1990: 19, 173, 183 (description, key to species of the USSR, keyed); Johnson 1992: 355 (catalog of world species); Kononova 1995: 61, 69 (keyed, diagnosis, key to species of Russian Far East); Austin and Field 1997: 20, 68 (structure of ovipositor system, discussion of phylogenetic relationships); Narendran and Ramesh Babu 1990: 2 (key to species of India); Lê 2000: 31, 46 (keyed, description, key to species); Loiácono and Margaría 2002: 557 (catalog of Brazilian species); Mineo 2004: 174 (distribution in Sicily); Rajmohana 2006: 116, 119, 120 (description, keyed, key to species of India); Kononova and Fursov 2007a: 57 (description); Kononova and Fursov 2007b: 98 (description); Kononova and Kozlov 2008: 23, 257, 258 (description, keyed, key to species of Palearctic region); Rajmohana and Peter 2013: 76 (key to species Calliscelio rugosus Rajmohana & Peter and Calliscelio agaliensis Narendran & Ramesh Babu); Talamas and Buffington 2015: 12. (fossil in Dominican amber); Talamas, Johnston-Jordan and Buffington 2016: 413, 416 (description, synonymy). http://zoobank.org/29B1D7E4-1173-4D61-B695-CA755632F5EAhttp://bioguid.osu.edu/xbiod_concepts/461
Baryteleia Kiefffer, 1926: 273, 544 (original description. Type: Macroteleia nigriceps Kieffer, by original designation, keyed, key to species); Muesebeck and Walkley 1956: 336 (citation of type species); Masner 1976: 36 (junior synonym of Calliscelio Ashmead). http://zoobank.org/AB0DBC82-18D8-431F-9069-783F4874CEC1http://bioguid.osu.edu/xbiod_concepts/8406
Caenoteleia Kieffer, 1926: 266, 550 (original description. Type: Caloteleia elegans Perkins, by monotypy, keyed); Muesebeck and Walkley 1956: 338 (citation of type species); Johnson 1992: 355 (catalog of world species); Masner et al. 2009: 60 (junior synonym of Calliscelio Ashmead, discussion of status). http://zoobank.org/5FEFDDD1-26AD-40D7-A498-BB4B5DF03630http://bioguid.osu.edu/xbiod_concepts/460
Ceratoteleia Kieffer, 1908: 121 (original description. Type Caloteleia grenadensis Ashmead, designated by Kieffer (1926), keyed); Kieffer 1910b: 65, 66, 88 (description, list of species, keyed); Dodd 1913a: 131, 144 (key to species of Australia); Dodd 1913b: 176 (comparison with Macroteleia Westwood); Kieffer 1913: 222 (description); Kieffer 1913: 232 (description); Kieffer 1914a: 315 (description, key to species of Europe and Algeria); Kieffer 1926: 273, 500 (description, keyed, key to species, designation of type species); Nixon 1931: 356 (keyed, key to species of Africa); Nixon 1933: 292 (keyed); Brues 1940: 82 (key to species of Baltic amber); Maneval 1940: 114 (keyed); Risbec 1950: 603 (key to species of Ethiopian region); Muesebeck and Walkley 1951: 705 (catalog of species of U.S. and Canada); Muesebeck and Walkley 1956: 341 (citation of type species); Masner 1976: 36 (junior synonym of Calliscelio Ashmead). http://zoobank.org/70CBF446-7CD7-4211-BF0C-F401DAE92DDAhttp://bioguid.osu.edu/xbiod_concepts/8400
Crama Galloway, 1984: 7, 8, 28 (original description. Type: Baryconus albicoxa Dodd, by original designation, key to Australian species, keyed); Johnson 1992: 364 (catalog of world species); Talamas, Johnston-Jordan and Buffington 2016: 413, 417 (junior synonym of Calliscelio Ashmead). http://zoobank.org/10BDF90E-0D3E-491E-98B2-8B39BB9D5871http://bioguid.osu.edu/xbiod_concepts/466
Glyptoteleia Kieffer, 1926: 272, 487 (original description. Type: Baryconus bisulcatus Kieffer, by monotypy, keyed); Muesebeck and Walkley 1956: 356 (citation of type species); Szabó 1962: 241 (diagnosis); Masner 1976: 36 (junior synonym of Calliscelio Ashmead); De Santis 1980: 312 (catalog of species of Brazil). http://zoobank.org/FFA0AF0B-A126-4A36-8DB4-E25910A88C3Dhttp://bioguid.osu.edu/xbiod_concepts/8405
Lispoteleia Galloway, 1984: 7, 9, 35 (original description. Type: Lispoteleia collina Galloway, by original designation, key to species of Australia, keyed); Johnson 1992: 421 (catalog of world species); Austin and Field 1997: 22, 68 (structure of ovipositor system, discussion of phylogenetic relationships); Talamas, Johnston-Jordan and Buffington 2016: 413, 417 (junior synonym of Calliscelio Ashmead). http://zoobank.org/A458DE09-DAFA-424E-B60E-1A831B19D01Ehttp://bioguid.osu.edu/xbiod_concepts/503
Mesoteleia Kieffer, 1917: 51 (original description. Type: Mesoteleia pallida Kieffer, by monotypy and original designation); Kieffer 1926: 271, 441 (description, keyed); Muesebeck and Walkley 1956: 369 (citation of type species); Baltazar 1966: 182 (cataloged, catalog of species of the Philippines); Masner 1976: 36 (junior synonym of Calliscelio Ashmead). http://zoobank.org/B6CD6365-672B-4E49-B3AE-6AAFA9C42DA6http://bioguid.osu.edu/xbiod_concepts/8404
Prosanteris Kieffer, 1908: 121, 136 (original description. Type: Anteris nigriceps Ashmead, designated by Kieffer (1910b), keyed); Kieffer 1910b: 65, 87 (description, key to subgenera, list of species, keyed); Kieffer 1913: 232 (description); Kieffer 1926: 272, 437 (description, keyed, key to species); Muesebeck and Walkley 1951: 704 (catalog of species of U.S. and Canada); Muesebeck and Walkley 1956: 391 (citation of type species); Muesebeck 1958: 93 (junior synonym of Ceratoteleia Kieffer). http://zoobank.org/31DB13FB-2699-4E1D-A023-1BA21C1B9503http://bioguid.osu.edu/xbiod_concepts/8401
Uroscelio Kieffer, 1914: 291 (original description. Type: Uroscelio luteipes Kieffer, by monotypy and original designation); Kieffer 1926: 268, 409 (description, keyed); Muesebeck and Walkley 1956: 408 (citation of type species); Baltazar 1966: 180 (cataloged, catalog of species of the Philippines); Masner 1976: 36 (junior synonym of Calliscelio Ashmead). http://zoobank.org/91A2A4C7-7D11-4723-8C53-02DEA8A0213Bhttp://bioguid.osu.edu/xbiod_concepts/8403
Xentor Masner & Johnson, 2007: 12, 14 (original description. Type: Xentor schlingeri Masner & Johnson, by original designation, key to species); Talamas, Johnston-Jordan and Buffington 2016: 416 (junior synonym of Calliscelio Ashmead). http://zoobank.org/1578C9FB-4A24-42D6-922D-05CDA2626906http://bioguid.osu.edu/xbiod_concepts/211604
Yunkara Galloway, 1984: 9, 33 (original description. Type: Yunkara inornata Galloway, by monotypy and original designation, keyed); Johnson 1992: 510 (catalog of world species); Talamas, Johnston-Jordan and Buffington 2016: 413, 418 (junior synonym of Calliscelio Ashmead). http://zoobank.org/4FBE9CB9-3B71-4DFB-BCE0-781664A31929http://bioguid.osu.edu/xbiod_concepts/578

#### Description

(based on New World species). Length: 1.27–3.88 mm; body moderately to markedly elongate, robust.

**Head.** Head shape in dorsal view: transverse. Hyperoccipital carina: absent; present. Occipital carina: present, complete medially; present laterally, broadly interrupted medially; completely absent. Occipital carina sculpture: crenulate; unsculptured. OOL: lateral ocellus nearly contiguous with inner orbits, OOL < 0.5 OD; lateral ocellus contiguous with inner orbit. Upper frons: convex, without frontal shelf. Scrobe shape: frons broadly convex, without distinct scrobe. Frons sculpture: scrobe largely smooth, otherwise granulate or variably punctate. Submedian carina: absent. Orbital carina: absent. Inner orbits: diverging ventrally. IOS/EH: IOS distinctly less than EH; IOS slightly greater than EH. Interantennal process: short, often excavate medially. Central keel: present; absent. Torulus opening: laterally on interantennal process. Lower frons striae: absent. Malar sulcus: present. Compound eye size: of normal proportions, not significantly reduced. Compound eye setation: glabrous; sparsely setose; densely setose. Gena: broad, convex, distinctly produced behind eye. Clypeus shape: narrow, slightly convex medially, lateral corner not produced. Apical margin of clypeus: straight. Anteclypeus: absent. Postclypeus: absent. Labrum: not visible. Mandible shape: moderate. Mandibular teeth: apex with 3, acute, subequal teeth. Arrangement of mandibular teeth: transverse. Number of maxillary palpomeres: 4. Shape of maxillary palpomeres: cylindrical. Number of labial palpomeres: 2.

**Antenna.** Number of antennomeres in female: 12. Number of antennomeres in male: 12. Insertion of radicle into A1: parallel to longitudinal axis of A1. Shape of A1: more or less cylindrical, not flattened. Length of A3 of female: distinctly longer than A2. Number of clavomeres in female antenna: 6. Claval formula of female antenna: A12–A7/1-2-2-2-2-1. Arrangement of doubled multiporous plate sensilla on female clava: in longitudinal pairs. Tyloid distribution on male antenna: A5 only. Shape of male flagellum: filiform.

**Mesosoma.** Mesosoma shape in dorsal view: longer than wide. Mesosoma shape in lateral view: longer than high. Medial portion of transverse pronotal carina: weakly indicated laterally; absent. Posterior apex of pronotum in dorsal view: straight, bifid apically to articulate with tegula. Vertical epomial carina: absent. Dorsal epomial carina (lateral portion of transverse pronotal carina of Vilhelmsen et al. 2010): present. Anterior face of pronotum: oblique, visible dorsally, short. Lateral face of pronotum: weakly concave below position of dorsal epomial carina. Netrion: present. Netrion shape: narrow to moderately wide, open ventrally. Anterior portion of mesoscutum: vertical, flexed ventrally to meet pronotum. Mesoscutum shape: semielliptical, excavate at base of wings. Skaphion: absent. Notauli: present, percurrent; present, abbreviated. Parapsidal lines: absent. Admedial lines: absent. Transscutal articulation: well-developed, narrow. Shape of mesoscutellum: quadrate to trapezoidal. Armature of mesoscutellum: absent. Surface of mesoscutellum: convex throughout. Median longitudinal furrow on mesoscutellum: absent. Shape of axillula: small, dorsal margin sinuate. Metascutellum: clearly differentiated. Metascutellar armature: absent. Metascutellar setation: glabrous. Metapostnotum: not defined externally. Extent of metasomal depression of propodeum: percurrent, extending anteriorly to anterior margin of propodeum. Lateral propodeal projection: absent. Mesopleural carina: present, extending at least to sternaulus; absent or strongly abbreviated, present only near mid coxa. Mesal course of acetabular carina: projecting as small spur anteriorly, not separating fore coxae. Mesopleural pit: present. Sternaulus: absent. Posterodorsal corner of mesopleuron: rounded anteriorly.

**Legs.** Number of mid tibial spurs: 1. Number of hind tibial spurs: 1. Dorsal surface of hind coxa: smooth; transversely rugose. Hind tibia shape: cylindrical, ecarinate. Trochantellus: indicated by transverse sulcus on femur.

**Wings.** Wing development of female: macropterous. Wing development of male: macropterous. Tubular veins in fore wing: present. Bulla of fore wing R: absent. Extent of marginal venation of fore wing: distinct marginal or postmarginal veins developed. Origin of r-rs in fore wing: arising from marginal vein along costal margin. Development of basal vein (Rs+M) in fore wing: spectral; nebulous, strongly pigmented; absent. Development of R in hind wing: elongate, extending to costal margin.

**Metasoma.** Number of external terga in female: 6. Number of external sterna in female: 6. Number of external terga in male: 7. Number of external sterna in male: 7. Shape of metasoma: lanceolate. Laterotergites: present, narrow. Laterosternites: present. T1 of female: more or less evenly convex; produced medially into cylindrical or elliptical horn housing ovipositor. Relative size of metasomal segments: T2–T4 largest, subequal in size. Terga with basal crenulae: T2. Sublateral carinae on tergites: absent. Median longitudinal carina on metasomal terga: absent. Shape of female T6: flattened. Shape of posterior margin of male T7: rounded. Anterior margin of S1: not produced anteriorly, concave. Distribution of felt fields: absent. Ovipositor type: *Scelio*-type (Austin and Field 1997).

#### Diagnosis.

*Calliscelio* may be distinguished from other genera of the subfamily by the combination of the following characters: eyes glabrous in many species but in some with short hairs or even densely hairy; skaphion never developed; metanotum medially produced into a transverse plate or lamella, neither spinose nor toothed laterally; propodeum usually unarmed, often excavate to contain T1 horn, only in a few species with posterolateral corner acute; T6 in females often elongate, sword-like, depressed dorsoventrally. *Calliscelio* is most similar to *Holoteleia* Kieffer and *Probaryconus* Kieffer in the tribe Calliscelionini and *Calotelea* in Psilanteridini in body shape and some external characters. The following key is used to separate these genera with the fewest characters possible.

#### Key to separate *Calliscelio*, *Calotelea*, *Holoteleia* and *Probaryconus*

**Table:** 

1	Skaphion indicated posteriorly by more or less distinct rim	***Calotelea* Westwood**
–	Skaphion never developed, no rim posteriorly	**2**
2	Genal striae present; epomial carina present	***Probaryconus* Kieffer**
–	Genal striae absent; epomial carina absent	**3**
3	Metanotum medially notably wider than at sides, expanded into lamella	***Calliscelio* Ashmead**
–	Metanotum narrow, strip-like, medially not produced into lamella	***Holoteleia* Kieffer**

#### New World species of *Calliscelio* Ashmead

*Calliscelio absconditum* Chen & Johnson, sp. n.

*Calliscelio absum* Chen & Johnson, sp. n.

*Calliscelio alcoa* Chen & Masner, sp. n.

*Calliscelio amadoi* Chen & Johnson, sp. n.

*Calliscelio armila* Chen & Masner, sp. n.

*Calliscelio bidens* Chen & Masner, sp. n.

*Calliscelio bisulcatus* (Kieffer, 1910)

*Calliscelio brachys* Chen & Johnson, sp. n.

*Calliscelio brevinotaulus* Chen & Johnson, sp. n.

*Calliscelio brevitas* Chen & Johnson, sp. n.

*Calliscelio carinigena* Chen & Johnson, sp. n.

*Calliscelio crater* Chen & Johnson, sp. n.

*Calliscelio crena* Chen & Johnson, sp. n.

*Calliscelio eboris* Chen & Johnson, sp. n.

*Calliscelio elegans* (Perkins)

*Calotelea tanugatra* Narendran

*Calliscelio extenuatus* Chen & Johnson, sp. n.

*Calliscelio flavicauda* Chen & Johnson, sp. n.

*Calliscelio foveolatus* Chen & Johnson, sp. n.

*Calliscelio gatineau* Chen & Johnson, sp. n.

*Calliscelio glaber* Chen & Masner, sp. n.

*Calliscelio granulatus* Chen & Masner, sp. n.

*Calliscelio laticinctus* Ashmead, 1893

*Calliscelio latifrons* Chen & Johnson, sp. n.

*Calliscelio levis* Chen & Johnson, sp. n.

*Calliscelio longius* Chen & Johnson, sp. n.

*Calliscelio magnificus* Chen & Masner, sp. n.

*Calliscelio migma* Chen & Johnson, sp. n.

*Calliscelio minutia* Chen & Johnson, sp. n.

*Calliscelio paraglaber* Chen & Johnson, sp. n.

*Calliscelio pararemigio* Chen & Masner, sp. n.

*Calliscelio prolixus* Chen & Johnson, sp. n.

*Calliscelio punctatifrons* Chen & Johnson, sp. n.

*Calliscelio remigio* Chen & Masner, sp. n.

*Calliscelio rubriclavus* (Ashmead, 1887), comb. n.

*Anteris nigriceps* Ashmead, 1893, syn. n.

*Caloteleia Marlattii* Ashmead, 1893, syn. n.

*Caloteleia grenadensis* Ashmead, 1896, syn. n.

*Macroteleia ruskini* Girault, 1920, syn. n.

*Calliscelio ruga* Chen & Johnson, sp. n.

*Calliscelio rugicoxa* Chen & Masner, sp. n.

*Calliscelio sfina* Chen & Johnson, sp. n.

*Calliscelio storea* Chen & Johnson, sp. n.

*Calliscelio suni* Chen & Johnson, sp. n.

*Calliscelio telum* Chen & Johnson, sp. n.

*Calliscelio torqueo* Chen & Johnson, sp. n.

*Calliscelio virga* Chen & Johnson, sp. n.

#### Key to females of *Calliscelio* of the New World

**Table:** 

1	Occipital carina complete medially (Figs 19, 37, 43, 49, 55, 73, 91, 103, 109, 115, 121, 133, 139, 145, 151, 157, 163, 169, 181, 193, 199, 217, 235, 247, 259, 271)	**2**
–	Occipital carina interrupted medially (Figs 25, 31, 61, 67, 79, 85, 97, 127, 175, 187, 205, 229, 241, 253, 265)	**28**
2	Eye bare (Figs 38, 74, 92, 110, 116, 122, 140, 152, 158, 164, 170, 182, 194, 218)	**3**
–	Eye setose (Figs 44, 56, 50, 103, 133, 146, 200, 236, 248, 260, 272)	**17**
3	A4 distinctly shorter than A3 (Figs 18, 36, 72, 90, 114, 118, 182)	**4**
–	A4 approximately as long as or distinctly longer than A3 (Figs 10, 140, 150, 162, 166, 190)	**11**
4	Horn on T1 weakly developed, smooth (Figs 117, 183)	**5**
–	Horn on T1 large and distinct, variably sculptured (Figs 21, 39, 123, 217)	**6**
5	Metascutellum without a longitudinal median carina (Fig. 181); foveolae of scutoscutellar sulcus between notauli smaller than those along margin of axilla (Fig. 181); posterior vertex smooth throughout (Fig. 181)	***Calliscelio paraglaber* Chen & Johnson, sp. n.**
–	Metascutellum with a longitudinal median carina (Fig. 115); foveolae of scutoscutellar sulcus between notauli as large as those along margin of axilla (Fig. 115); posterior vertex smooth to coriaceous (Fig. 115)	***Calliscelio foveolatus* Chen & Johnson, sp. n.**
6	T6 strongly elongate, at least 2.0× longer than wide (Figs 63, 93)	**7**
–	T6 short, at most 1.5× longer than wide (Figs 21, 39, 123, 224)	**8**
7	Setae on frons short (Fig. 74); posterior vertex granulate to rugulose or densely punctate (Fig. 73); mesoscutum largely coriaceous with dense and fine punctures at posterior extreme (Fig. 73)	***Calliscelio brevitas* Chen & Johnson, sp. n.**
–	Setae on frons long (Fig. 92); posterior vertex largely smooth with sparse fine punctures (Fig. 91); mesoscutum with anterior margin rugulose, remainder smooth (Fig. 91)	***Calliscelio crena* Chen & Johnson, sp. n.**
8	R1 as long as r-rs (Figs 13, 212); IOS slightly less than or greater than EH (Figs 122, 218)	**9**
–	R1 as long as 2.0× length of r-rs (Figs 21, 39); IOS distinctly less than EH (Figs 20, 38)	**10**
9	Mesepisternum below mesopleural depression densely punctate (Fig. 211); horn on T1 large and distinct, granulate or rugose dorsally (Fig. 217)	***Calliscelio rubriclavus* (Ashmead)**
–	Mesepisternum below mesopleural depression smooth (Fig. 119); horn on T1 present as a small bulge, dorsally granulate medially, with V-shaped keels on edge (Fig. 123)	***Calliscelio gatineau* Chen & Johnson, sp. n.**
10	Posterior vertex largely smooth with sparse fine punctures (Fig. 37); Rs+M dark, nebulous (Fig. 39); T1 horn transversely striate (Fig. 39)	***Calliscelio amadoi* Chen & Johnson, sp. n.**
–	Posterior vertex granulate to rugulose (Fig. 19); Rs+M spectral (Fig. 21); T1 horn with V-shaped striae (Fig. 21)	***Calliscelio absconditum* Chen & Johnson, sp. n.**
11	T6 short, at most 1.5× longer than wide (Figs 111, 153, 171, 195)	**12**
–	T6 strongly elongate, at least 2.0× longer than wide (Figs 159, 165)	**15**
12	Horn on T1 absent or weakly developed (Figs 153, 195); dorsal propodeum not excavate medially, lateral propodeal carinae meeting anteromedially (Figs 151, 193)	**13**
–	Horn on T1 present as a small bulge (Figs 111, 171); dorsal propodeum shallowly excavate medially, with lateral propodeal carinae widely separated (Fig. 109, 169)	**14**
13	A4 distinctly longer than A3 (Fig. 192); upper frons densely setose (Fig. 194); T1 longitudinally striate medially (Fig. 195)	***Calliscelio prolixus* Chen & Johnson, sp. n.**
–	A4 as long as A3 (Fig. 150); upper frons sparsely setose (Fig. 152); T1 smooth medially (Fig. 153)	***Calliscelio levis* Chen & Johnson, sp. n.**
14	Scutoscutellar sulcus strongly foveolate medially (Fig. 109); upper frons densely setose (Fig. 110); T3 smooth throughout (Fig. 111)	***Calliscelio flavicauda* Chen & Johnson, sp. n.**
–	Scutoscutellar sulcus weakly foveolate medially (Fig. 169); upper frons sparsely setose (Fig. 170); T3 with longitudinal submedian striae (Fig. 171)	***Calliscelio migma* Chen & Johnson, sp. n.**
15	Horn on T1 smooth (Fig. 165); foveolae of scutoscutellar sulcus between notauli smaller than those along margin of axilla (Fig. 163)	***Calliscelio magnificus* Chen & Masner, sp. n.**
–	Horn on T1 at least partly transversely striate (Figs 139, 159); foveolae of scutoscutellar sulcus between notauli as large as those along margin of axilla (Fig. 139, 157)	**16**
16	A4 distinctly longer than A3 (Fig. 10); A5 longer than A3 (Fig. 10)	***Calliscelio longius* Chen & Johnson, sp. n.**
–	A4 slightly longer than A3 (Fig. 140); A5 shorter than A3 (Fig. 140)	***Calliscelio laticinctus* Ashmead**
17	Median keels on propodeum present (Figs 49, 235)	**18**
–	Median keels on propodeum absent (Figs 43, 55, 103, 133, 145, 199, 247, 259, 271)	**19**
18	Horn on T1 absent, anterior margin of T1 longitudinally striate (Fig. 81); hind coxae smooth (Fig. 47); T6 short, slightly longer than wide (Fig. 51)	***Calliscelio bidens* Chen & Masner, sp. n.**
–	Horn on T1 weakly developed, rugose dorsally (Fig. 237); hind coxae rugose (Fig. 233); T6 distinctly elongate, 2.0× longer than wide (Fig. 237)	***Calliscelio rugicoxa* Chen & Masner, sp. n.**
19	T6 strongly elongate, at least 2.0× longer than wide (Figs 135, 261)	**20**
–	T6 short, at most 1.5× longer than wide (Figs 45, 57, 105, 147, 201, 249, 273)	**22**
20	Fore wing strikingly banded, with dark bands basally, medially and apically, separated by light bands; R1 only slightly longer than r-rs	***Calliscelio elegans* (Perkins)**
–	Fore wing hyaline; R1 approximately as long as 2.0× length of r-rs	**21**
21	Setae of upper frons short, dense (Fig. 134); netrion rugose (Fig. 131); horn on T1 longitudinally striate (Fig. 135)	***Calliscelio granulatus* Chen & Johnson, sp. n.**
–	Setae of upper frons long, sparse (Fig. 260); netrion smooth (Fig. 257); horn on T1 rugulose (Fig. 261)	***Calliscelio telum* Chen & Johnson, sp. n.**
22	Horn on T1 absent (Fig. 105); dorsal propodeum not excavate medially (Fig. 103)	***Calliscelio extenuatus* Chen & Johnson, sp. n.**
–	Horn on T1 present (Figs 45, 57, 147, 201, 249, 273); dorsal propodeum excavate medially (Figs 43, 55, 145, 199, 247, 271)	**23**
23	Horn on T1 variably sculptured (Figs 45, 147, 249)	**24**
–	Horn on T1 smooth (Figs 57, 201, 273)	**26**
24	Notaulus abbreviated, at most reaching middle of mesoscutum (Fig. 145); T3 with submedian longitudinal striae (Fig. 147)	***Calliscelio latifrons* Chen & Johnson, sp. n.**
–	Notaulus percurrent (Figs 43, 247); T3 smooth throughout (Figs 45, 249)	**25**
25	Horn on T1 rugulose (Fig. 249); legs orange throughout (Fig. 244)	***Calliscelio storea* Chen & Johnson, sp. n.**
–	Horn on T1 smooth to rugulose medially, with V-shaped keels laterally (Fig. 45); legs with coxae and femora white, otherwise pale yellow throughout (Fig. 40)	***Calliscelio armila* Chen & Masner, sp. n.**
26	Frons below median ocellus densely punctate (Fig. 200); posterior vertex densely punctate (Fig. 199); R distinctly longer than r-rs (Fig. 201)	***Calliscelio punctatifrons* Chen & Johnson, sp. n.**
–	Frons below median ocellus largely smooth with sparse fine punctures (Figs 56, 272); posterior vertex granulate (Figs 55, 271); R approximately as long as r-rs (Fig. 268)	**27**
27	Metascutellum approximately 4.0× wider than long, smooth (Fig. 271); mesopleural carina absent (Fig. 269); mesepisternum below mesopleural depression smooth throughout (Fig. 269)	***Calliscelio virga* Chen & Johnson, sp. n.**
–	Metascutellum approximately 2.0× wider than long, rugose anteriorly, smooth posteriorly (Fig. 55); mesopleural carina present (Fig. 53); mesepisternum below mesopleural depression with a row of foveae along mesopleural carina	***Calliscelio bisulcatus* (Kieffer)**
28	Horn on T1 absent or at most weakly developed (Figs 27, 63, 99, 177, 261)	**29**
–	Horn on T1 present (Figs 33, 69, 87, 129, 189, 207, 243, 267)	**34**
29	Notaulus abbreviated, at most reaching middle of mesoscutum (Figs 25, 61)	**30**
–	Notaulus percurrent (Figs 97, 175, 229, 259)	**31**
30	A4 distinctly shorter than A3 (Fig. 22); postgena behind outer orbit coriaceous (Fig. 23)	***Calliscelio absum* Chen & Johnson, sp. n.**
–	A4 as long as A3 (Fig. 60); postgena behind outer orbit smooth (Fig. 59)	***Calliscelio brachys* Chen & Johnson, sp. n.**
31	Eye hairy (Fig. 229); A6 distinctly longer than wide	***Calliscelio ruga* Chen & Johnson, sp. n.**
–	Eye bare (Figs 98, 176, 260); A6 subquadrate or distinctly transverse	**32**
32	Fore wing hyaline with an infuscate band in the middle (Fig. 174); A4 distinctly shorter than A3; postgena behind outer orbit granulate (Fig. 173)	***Calliscelio minutia* Chen & Johnson, sp. n.**
–	Fore wing entirely hyaline (Figs 99, 258); A4 as long as A3; postgena behind outer orbit smooth (Figs 95, 257)	**33**
33	Central keel of frons absent (Fig. 260); frons below median ocellus smooth (Fig. 260); Rs+M nebulose, weakly pigmented (Fig. 261)	***Calliscelio suni* Chen & Johnson, sp. n.**
–	Central keel of frons present (Fig. 98); frons below median ocellus coriaceous (Fig. 98); Rs+M spectral (Fig. 99)	***Calliscelio eboris* Chen & Johnson, sp. n.**
34	Frons and posterior vertex smooth (Figs 127, 128, 187, 188, 205, 206, 265, 266)	**35**
–	Frons and posterior vertex variably sculptured (Figs 31, 32, 67, 68, 79, 80, 85, 86, 241, 242)	**38**
35	T6 strongly elongate, approximately 3.0× longer than wide (Figs 189, 207); Metascutellum finely granulate (Figs 187, 205)	**36**
–	T6 short, slightly longer than wide (Figs 129, 267); Metascutellum finely smooth (Figs 127, 265)	**37**
36	Head strongly transverse in dorsal view (Fig. 205); T1 horn transversely striate (Fig. 207)	***Calliscelio remigio* Chen & Masner, sp. n.**
–	Head subglobose in dorsal view (Fig. 187); T1 horn densely and concentrically striate anteriorly, smooth posteriorly (Fig. 189)	***Calliscelio pararemigio* Chen & Masner, sp. n.**
37	A4 distinctly longer than A3 (Fig. 264); horn on T1 smooth (Fig. 267)	***Calliscelio torqueo* Chen & Johnson, sp. n.**
–	A4 distinctly shorter than A3 (Fig. 126); horn on T1 concentrically striate (Fig. 129)	***Calliscelio glaber* Chen & Masner, sp. n.**
38	Hyperoccipital carina present (Figs 79, 85)	**39**
–	Hyperoccipital carina absent (Figs 31, 67, 241)	**40**
39	Postgena behind outer orbit with large foveae (Fig. 83); horn on T1 large and distinct, rugose medially, with V-shaped keels laterally (Fig. 87)	***Calliscelio crater* Chen & Johnson, sp. n.**
–	Postgena with a carina along outer orbit (Fig. 77); horn on T1 weakly indicated, smooth to somewhat transversely striate (Fig. 69)	***Calliscelio carinigena* Chen & Johnson, sp. n.**
40	Mesoscutum densely punctate (Fig. 31); horn on T1 densely and transversely striate (Fig. 33); T6 strongly elongate, tapering apically (Fig. 33)	***Calliscelio alcoa* Chen & Masner, sp. n.**
–	Mesoscutum granulate (Figs 67, 241); horn on T1 with V-shaped striae (Figs 69, 243); T6 short, subtriangular (Figs 69, 243)	**41**
41	Notaulus abbreviated, at most reaching anteriorly to middle of mesoscutum (Fig. 67); ventral metapleural area largely smooth (Fig. 65); S3 largely smooth with sparse fine punctures	***Calliscelio brevinotaulus* Chen & Johnson, sp. n.**
–	Notaulus percurrent (Fig. 241); ventral metapleural area rugose throughout (Fig. 239); S3 densely punctate medially, longitudinally striate laterally	***Calliscelio sfina* Chen & Johnson, sp. n.**

#### Key to males

(unknown for *Calliscelio amadoi*, *Calliscelio bidens*, *Calliscelio brevitas*, *Calliscelio foveolatus*, *Calliscelio gatineau*, *Calliscelio levis*, *Calliscelio prolixus*, *Calliscelio rugicoxa*, *Calliscelio ruga* and *Calliscelio storea*)

**Table:** 

1	Occipital carina complete medially (Figs 19, 43, 55, 91, 103, 109, 133, 139, 145, 157, 163, 169, 181, 199, 217, 259, 271)	**2**
–	Occipital carina interrupted medially (Figs 25, 31, 61, 67, 79, 85, 97, 127, 175, 187, 205, 241, 259, 265)	**19**
2	Eye bare (Figs 20, 92, 110, 140, 158, 164, 170, 182)	**3**
–	Eye setose (Figs 44, 103, 133, 200, 260, 272)	**11**
3	Rs+M spectral (Figs 21, 212); R distinctly shorter than r-rs	**4**
–	Rs+M nebulose, pigmented (Figs 15, 90, 159, 165, 171, 183); R as long as or longer than r-rs	**5**
4	Mesepisternum below mesopleural depression densely punctate (Fig. 211); IOS slightly less than EH (Fig. 218); R1 as long as r-rs	***Calliscelio rubriclavus* (Ashmead)**
–	Mesepisternum below mesopleural depression smooth (Fig. 17); IOS distinctly less than EH (Fig. 20); R1 approximately as long as 2.0× length of r-rs	***Calliscelio absconditum* sp. n.**
5	Upper frons densely setose (Figs 110, 140, 158, 164)	**6**
–	Upper frons sparsely setose (Figs 92, 170, 182)	**9**
6	Metascutellum smooth (Fig. 163)	***Calliscelio magnificus* Chen & Masner, sp. n.**
–	Metascutellum rugose (Figs 109, 139, 157)	**7**
7	A11 approximately 4.5× longer than wide	***Calliscelio longuis* Chen & Johnson, sp. n.**
–	A11 approximately 3.0× longer than wide	**8**
8	T3 smooth throughout (Fig. 111); hind femora brown	***Calliscelio flavicauda* Chen & Johnson, sp. n.**
–	T3 with longitudinal submedian striae; hind femora yellow	***Calliscelio laticinctus* Ashmead**
9	A11 approximately 2.0× longer than wide; length of T5 tyloid greater than 0.5× length of A5	***Calliscelio crena* Chen & Johnson, sp.n**
–	A11 approximately 4.0× longer than wide; length of T5 tyloid approximately 0.3× length of A5	**10**
10	Mesoscutum smooth throughout (Fig. 181); T3 smooth throughout (Fig. 183)	***Calliscelio paraglaber* Chen & Johnson, sp. n.**
–	Mesoscutum coriaceous or smooth with sparse punctures (Fig. 169); T3 with longitudinal submedian striae (Fig. 171)	***Calliscelio migma* Chen & Johnson, sp. n.**
11	R1 approximately as long as r-rs	**12**
–	R1 at least 2.0× length of r-rs	**14**
12	Fore wing strikingly banded, with dark bands basally, medially and apically, separated by light bands	***Calliscelio elegans* (Perkins)**
–	Fore wing hyaline (Figs 54, 268)	**13**
13	Metascutellum rugose (Fig. 55); mesepisternum below mesopleural depression largely smooth with a row of foveae along mesopleural carina	***Calliscelio bisulcatus* (Kieffer)**
–	Metascutellum smooth (Fig. 271); mesepisternum below mesopleural depression smooth throughout (Fig. 269)	***Calliscelio virga* Chen & Johnson, sp. n.**
14	IOS slightly greater than EH (Fig. 146); antennal flagellomeres moniliform, A11 as long as wide (Fig. 5)	***Calliscelio latifrons* Chen & Johnson, sp. n.**
–	IOS less than EH (Figs 44, 104, 134, 200, 260); antennal flagellomeres filiform, A11 at least 2.5× longer than wide (Fig. 2)	**15**
15	Posterior vertex densely punctate (Fig. 199); mesostutellum smooth with sparse fine punctures (Fig. 199)	***Calliscelio punctatifrons* Chen & Johnson, sp. n.**
–	Posterior vertex granulate (Figs 43, 103, 133, 259); mesoscutellum granulate (Figs 43, 103, 133, 259)	**16**
16	R1 greater than 3.0× length of r-rs	***Calliscelio extenuatus* Chen & Johnson, sp. n.**
–	R1 approximately as long as 2.0× length of r-rs	**17**
17	Frons below median ocellus largely smooth with sparse fine punctures (Fig. 44); IOS slightly less than EH (Fig. 44)	***Calliscelio armila* Chen & Masner, sp. n.**
–	Frons below median ocellus granulate (Figs 134, 260); IOS distinctly less than EH (Figs 134, 260)	**18**
18	Rs+M nebulose, strongly pigmented; dorsal propodeum with one or two longitudinal keels lateral to median keels	***Calliscelio telum* Chen & Johnson, sp. n.**
–	Rs+M spectral; dorsal propodeum rugose rugose throughout, only with medial keels	***Calliscelio granulatus* Chen & Masner, sp. n.**
19	Notaulus abbreviated, at most reach anteriorly to middle of mesoscutum (Figs 15, 61, 61)	**20**
–	Notaulus percurrent (Figs 31, 79, 85, 97, 127, 175, 187, 205, 241, 259, 265)	**22**
20	Netrion rugose (Fig. 65); mesopleural carina absent (Fig. 65)	***Calliscelio brevinotaulus* Chen & Johnson, sp. n.**
–	Netrion smooth (Figs 23, 59); mesopleural carina present (Figs 23, 59)	**21**
21	Postgena behind outer orbit smooth (Fig. 59); length of A5 tyloid approximately 0.3× length of A5; dorsal propodeum with one or two longitudinal keels lateral to median keel	***Calliscelio brachys* Chen & Johnson, sp. n.**
–	Postgena behind outer orbit coriaceous (Fig. 23); length of A5 tyloid longer than 0.5× length of A5; lateral propodeal area rugose throughout	***Calliscelio absum* Chen & Johnson, sp. n.**
22	Frons below median ocellus smooth (Figs 128, 188, 206, 260, 266)	**23**
–	Frons below median ocellus sculptured (Figs 32, 80, 86, 98, 176, 242)	**27**
23	Rs+M nebulose, pigmented (Figs 258, 264)	**24**
–	Rs+M spectral (Figs 126, 186, 204)	**25**
24	Metascutellum rugose (Fig. 259); R distinctly shorter than r-rs; T3 smooth throughout (Fig. 261)	***Calliscelio suni* Chen & Johnson, sp. n.**
–	Metascutellum smooth (Fig. 265); R approximately as long as r-rs; T3 with longitudinal submedian striae (Fig. 267)	***Calliscelio torqueo* Chen & Johnson, sp. n.**
25	Mesopleural carina absent (Fig. 125); mesoscutellum smooth throughout (Fig. 127)	***Calliscelio glaber* Chen & Masner, sp. n.**
–	Mesopleural carina present (Figs 185, 203); mesoscutellum with sparse fine punctures (Fig. 187, 205)	**26**
26	Head strongly transverse in dorsal view (Fig. 205); S3 densely punctate or punctate rugose (Fig. 207)	***Calliscelio remigio* Chen & Masner, sp. n.**
–	Head subglobose in dorsal view (Fig. 187); S3 largely smooth with sparse fine punctures (Fig. 189)	***Calliscelio pararemigio* Chen & Masner, sp. n.**
27	Mesoscutum densely punctate (Fig. 31); R approximately as long as r-rs;	***Calliscelio alcoa* Chen & Masner, sp. n.**
–	Mesoscutum coriaceous or granulate (Figs 79, 85, 97, 175, 241); R distinctly shorter than r-rs;	**28**
28	Hyperoccipital carina present (Figs 79, 85, 97)	**29**
–	Hyperoccipital carina absent (Figs 175, 241)	**31**
29	Postgena behind outer orbit with large foveae (Fig. 83)	***Calliscelio crater* Chen & Johnson, sp. n.**
–	Postgena behind outer orbit without foveae (Figs 77, 95)	**30**
30	Posterior vertex transversely striate (Fig. 97); postgena behind outer orbit smooth (Fig. 95)	***Calliscelio eboris* Chen & Johnson, sp. n.**
–	Posterior vertex granulate to rugulose (Fig. 79); postgena behind outer orbit with a carina along outer orbit (Fig. 77)	***Calliscelio carinigena* Chen & Johnson, sp. n.**
31	S3 smooth (Fig. 177); fore wing hyaline with infuscate band in the middle (Fig. 174); ventral metapleural area smooth dorsally, densely punctate ventrally (Fig. 173)	***Calliscelio minutia* Chen & Johnson, sp. n.**
–	S3 densely punctate medially, longitudinally striate laterally (Fig. 243); fore wing hyaline throughout (Fig. 240); ventral metapleural area rugose (Fig. 239)	***Calliscelio sfina* Chen & Johnson, sp. n.**

### Calliscelio absconditum

Taxon classificationAnimaliaHymenopteraPlatygastridae

Chen & Johnson sp. n.

http://zoobank.org/7A1C9B4D-B25D-403F-9DE3-B2278A4BFED8

http://bioguid.osu.edu/xbiod_concepts/384359

Figures 16–21

#### Description.

Body length of female: 2.04–2.54 mm (n=20). Body length of male: 1.70–2.36 mm (n=20). Color of head: variably brown to black. Color of antennal clava (A7–A12): dark brown to black. Shape of head: subglobose. Central keel of frons: absent. Setation of upper frons: with sparse, long setae. IOS/EH: IOS distinctly less than EH. Sculpture of ventrolateral frons: smooth to coriaceous. Sculpture of frons below median ocellus: coriaceous. Sculpture of posterior vertex: granulate to rugulose. Hyperoccipital carina: absent. Occipital carina medially: complete, weakly crenulate throughout. Length of OOL: less than 0.5× ocellar diameter. Sculpture of postgena behind outer orbit: largely smooth with small granulate area. Ocular setae: absent. A4 in female: distinctly shorter than A3. A5 in female: shorter than A3, as long as wide. Shape of female A6: distinctly wider than long. Form of male antennal flagellomeres: filiform, A11 approximately 2.0× longer than wide. Length of A5 tyloid in male: greater than 0.5× length of A5.

Color of mesosoma in female: orange throughout; variably orange to pale brown. Color of mesosoma in male: variably orange to pale brown; dark brown throughout. Sculpture of dorsal pronotal area: rugose. Sculpture of lateral pronotal area: smooth throughout. Sculpture of netrion: smooth. Notaulus: percurrent. Sculpture of mesoscutum: granulate. Shape of mesoscutellum: semiellipsoidal. Foveolae of scutoscutellar sulcus between notauli: as large as those along margin of axilla. Sculpture of mesoscutellum: smooth with sparse fine punctures. Shape of metascutellum: posterior margin straight, approximately 3.0× wider than long. Sculpture of metascutellum in female: rugose. Sculpture of metascutellum in male: rugose. Dorsal propodeum in female: shallowly excavate medially, with lateral propodeal carinae widely separated. Sculpture of dorsal propodeum in female: rugose. Sculpture of dorsal propodeum in male: rugose. Median keels on propodeum in female: absent. Mesopleural carina: absent. Sculpture of mesepisternum below mesopleural depression: smooth. Sculpture of ventral metapleural area: largely smooth with an oblique carina. Color of legs: pale yellow throughout. Sculpture of hind coxa: smooth.

Color of fore wing: hyaline. Rs+M: spectral. Setae on R: long, erect, surpassing the margin of the wing. Length of R: distinctly shorter than r-rs. Length of R1: approximately as long as 2.0× length of r-rs.

Color of metasoma in female: orange throughout; variably orange to pale brown. Color of metasoma in male: brown throughout. Horn on T1 in female: large and distinct. Sculpture of T1 horn dorsally: smooth to rugulose medially, with V-shaped keels laterally; with V-shaped striae. Sculpture of posterior margin of T1 in female: longitudinally striate throughout. Sculpture of T1 in male: longitudinally striate. Development of longitudinal striae on T2 in female: reaching posterior margin of T2. Sculpture of T3: smooth; smooth medially, coriaceous laterally. Shape of T6 in female: short, wider than long. Sculpture of S3: smooth to coriaceous.

#### Diagnosis.

This species is similar to *Calliscelio brevinotaulus*, *Calliscelio carinigena*, *Calliscelio crater* and *Calliscelio sfina* in color pattern, size, and habitus. It may be distinguished by the complete occipital carina and granulate to rugulose posterior vertex (Fig. 19).

**Figures 1–12.**
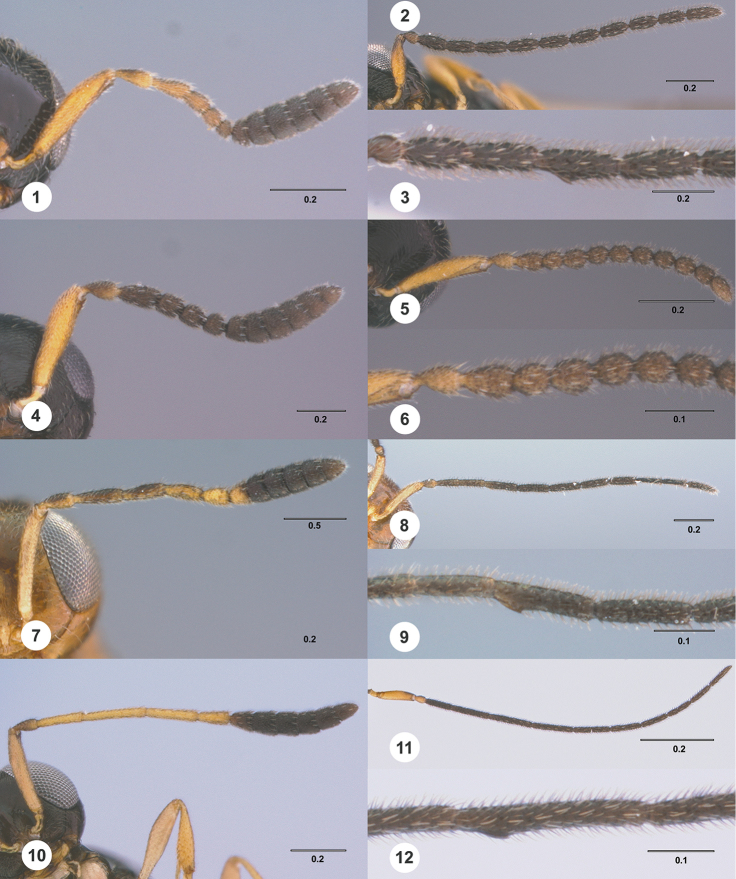
**1** *Calliscelio punctatifrons* sp. n., female, holotype (OSUC 191124), Antenna. **2–3** *Calliscelio punctatifrons* sp. n., male, paratype (OSUC 191125) **2** Antenna **3** Tyloid **4** *Calliscelio latifrons* sp. n., female, holotype (OSUC 323077), Antenna **5–6** *Calliscelio latifrons* sp. n., male, paratype (OSUC 323075) **5** Antenna **6** Tyloid **7** *Calliscelio alcoa* sp. n., female, holotype (OSUC 458212), Antenna **8–9** *Calliscelio alcoa* sp. n., male, paratype (OSUC 458222) **8** Antenna **9** Tyloid **10** *Calliscelio longius* sp. n., female, paratype (OSUC 193935), Antenna **11–12** *Calliscelio longius* sp. n., male, paratype (OSUC 193596) **11** Antenna **12** Tyloid. Scale bars in millimeters.

**Figures 13–15.**
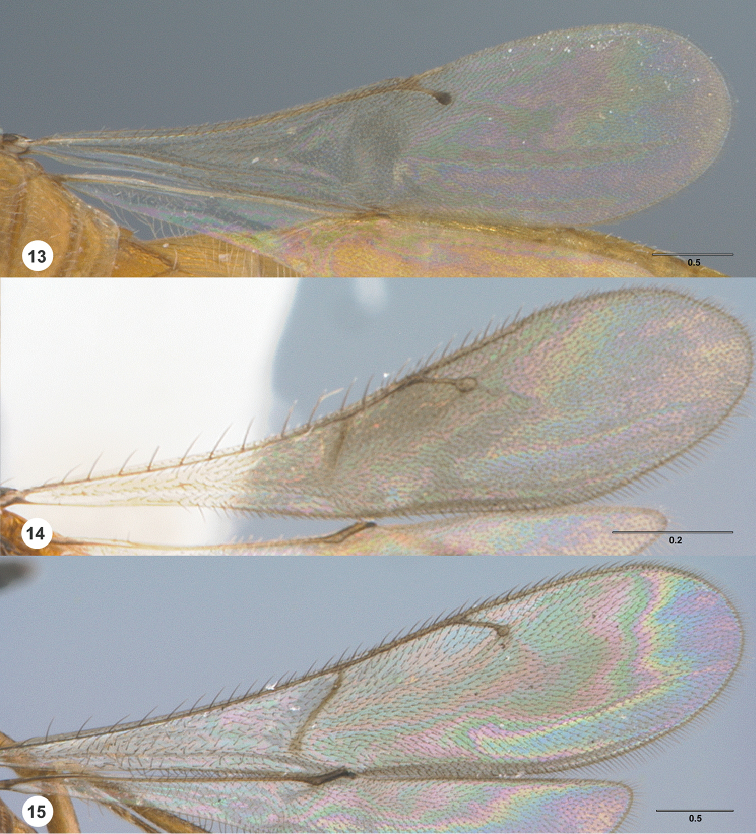
Fore wing, dorsal view **13** *Calliscelio gatineau* sp. n., female, holotype (OSUC 534340) **14** *Calliscelio storea* sp. n., female, holotype (OSUC 546117) **15** *Calliscelio laticinctus* Ashmead, female (OSUC 458242). Scale bars in millimeters.

**Figures 16–21.**
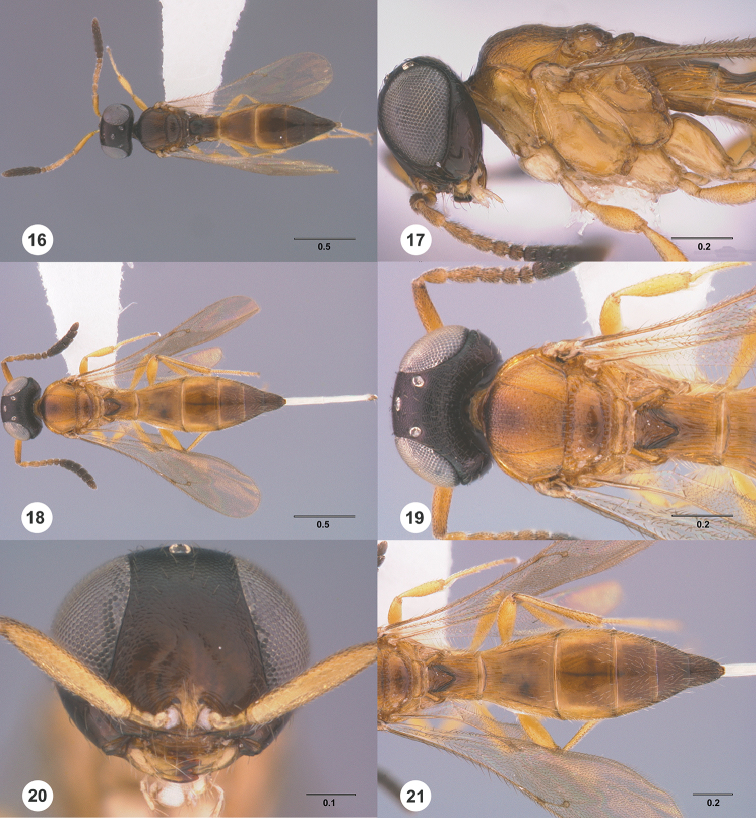
**16** *Calliscelio absconditum* sp. n., female, paratype (OSUC 134791), dorsal habitus **17–20** *Calliscelio absconditum* sp. n., female, holotype (OSUC 323924). **17** Head and mesosoma, lateral view **18** Dorsal habitus **19** Head and mesosoma, dorsal view **20** Head, anterior view **21** Metasoma, dorsal view. Scale bars in millimeters.

#### Etymology.

The epithet is used as a noun in apposition derived from the Latin word for hidden, in reference to the obscurity of the occipital carina from dorsal view.

#### Link to distribution map.

[http://hol.osu.edu/map-full.html?id=384359]

#### Material examined.

Holotype, female: **PARAGUAY**: Canindeyú Dept., Jejuí-mí, Bosque Mbaracayú Natural Reserve, 10.I.1997, B. Garcete, OSUC 323924 (deposited in MNHNPY). Paratypes: (59 females, 104 males) **BOLIVIA**: 7 females, OSUC 534032, 534038, 534040, 534054–534055, 534058, 534186 (CNCI). **BRAZIL**: 11 females, 28 males, OSUC 534520 (CNCI); OSUC 10439, 10444, 10601, 10610, 10653, 110189, 131761, 131787, 131827, 134436, 134786, 134875, 510887, 826 (MZSP); OSUC 10040, 10502, 10557, 10633, 10718, 10949, 110085, 110139, 110159, 110215, 11065, 112, 12321, 130, 131695, 131754, 131816, 131840, 131892, 133022, 133090, 134370, 134681, 134791 (OSUC). **PARAGUAY**: 41 females, 76 males, OSUC 534107–534115, 534559 (CNCI); OSUC 534683, 534695–534697, 534699–534700, 534702, 534704, 534706–534707, 534713–534714, 534726–534728, 534731–534749, 570521–570525, 570527–570534 (MNHNPY); OSUC 150602–150603, 150606, 150610–150611, 165099, 176064, 276773–276777, 276796, 278657–278658, 278661–278662, 278664–278665, 278668–278670, 278673, 278676, 278679, 322990, 323001–323003, 323027–323029, 323032, 323035, 323920–323923, 323925–323927, 412079–412082, 412085, 534725, 570526, 577174, 577176, 577178–577180, 577188–577189, 577340, 577343, 577346, 577349–577350 (OSUC).

### Calliscelio absum

Taxon classificationAnimaliaHymenopteraPlatygastridae

Chen & Johnson sp. n.

http://zoobank.org/2877EC6A-9602-499F-9075-0CCC91166CD9

http://bioguid.osu.edu/xbiod_concepts/384780

Figures 22–27

#### Description.

Body length of female: 1.49–2.58 mm (n=20). Body length of male: 1.49–1.60 mm (n=20). Color of head: black throughout; dark brown; orange throughout. Color of antennal clava (A7–A12): dark brown to black. Shape of head: subglobose. Central keel of frons: absent. Setation of upper frons: with sparse, short setae. IOS/EH: IOS distinctly less than EH. Sculpture of ventrolateral frons: smooth to granulate. Sculpture of frons below median ocellus: smooth; coriaceous. Sculpture of posterior vertex: smooth; coriaceous. Hyperoccipital carina: absent. Occipital carina medially: interrupted. Length of OOL: less than 0.5× ocellar diameter. Sculpture of postgena behind outer orbit: coriaceous. Ocular setae: absent. A4 in female: distinctly shorter than A3. A5 in female: shorter than A3, as long as wide. Shape of female A6: distinctly wider than long. Form of male antennal flagellomeres: filiform, A11 approximately 2.5× longer than wide. Length of A5 tyloid in male: greater than 0.5× length of A5.

Color of mesosoma in female: orange throughout; black throughout; orange to pale brown. Color of mesosoma in male: orange throughout; dark brown throughout; black throughout. Sculpture of dorsal pronotal area: smooth. Sculpture of lateral pronotal area: smooth throughout; smooth anteriorly, granulate posteriorly. Sculpture of netrion: smooth. Notaulus: abbreviated, at most reaching middle of mesoscutum. Sculpture of mesoscutum: coriaceous. Shape of mesoscutellum: semiellipsoidal. Foveolae of scutoscutellar sulcus between notauli: smaller than those along margin of axilla. Sculpture of mesoscutellum: coriaceous; anterior half granulate, posterior half smooth. Shape of metascutellum: posterior margin rounded, approximately 3.0× wider than long. Sculpture of metascutellum in female: with short longitudinal carinae. Sculpture of metascutellum in male: rugose. Dorsal propodeum in female: not excavate medially, lateral propodeal carinae meeting anteromedially. Sculpture of dorsal propodeum in female: rugose. Sculpture of dorsal propodeum in male: rugose. Median keels on propodeum in female: absent. Mesopleural carina: present. Sculpture of mesepisternum below mesopleural depression: smooth. Sculpture of ventral metapleural area: largely smooth with an oblique carina. Color of legs: pale yellow throughout; white throughout. Sculpture of hind coxa: smooth.

Color of fore wing: hyaline. Rs+M: spectral. Setae on R: long, erect, surpassing the margin of the wing. Length of R: distinctly shorter than r-rs. Length of R1: greater than 3.0× length of r-rs.

Color of metasoma in female: dark brown; orange throughout; yellow throughout. Color of metasoma in male: orange throughout; brown throughout. Horn on T1 in female: absent; weakly developed. Sculpture of T1 horn dorsally: smooth. Sculpture of posterior margin of T1 in female: longitudinally striate throughout. Sculpture of T1 in male: longitudinally striate. Development of longitudinal striae on T2 in female: reaching the middle of T2 medially; reaching posterior margin of T2. Sculpture of T3: smooth; smooth medially, longitudinally striate laterally. Shape of T6 in female: short, wider than long. Sculpture of S3: smooth.

**Figures 22–27.**
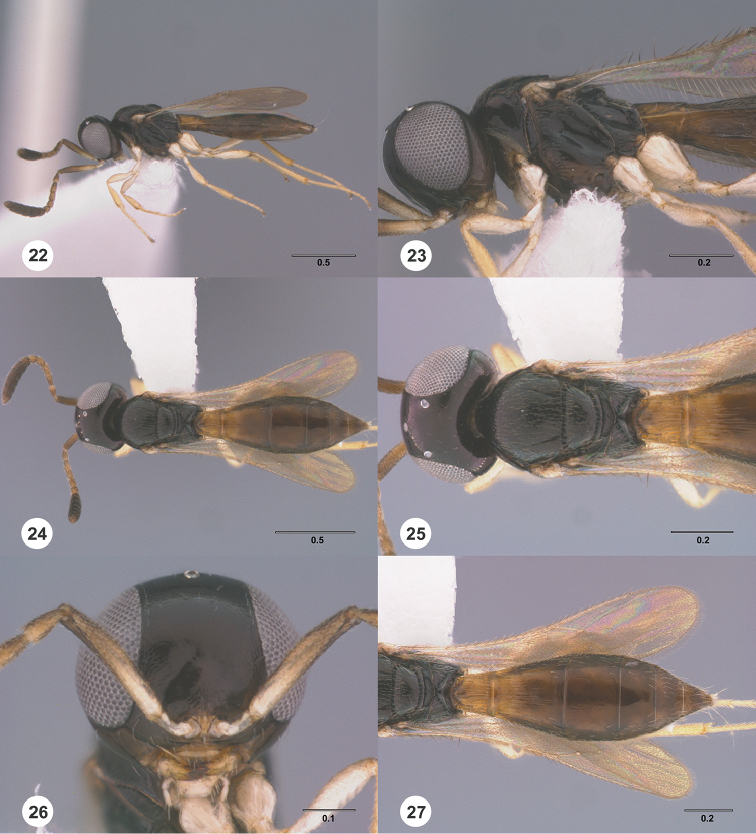
*Calliscelio absum* sp. n., female, holotype (OSUC 190716). **22** Lateral habitus **23** Head and mesosoma, lateral view **24** Dorsal habitus **25** Head and mesosoma, dorsal view **26** Head, anterior view **27** Metasoma, dorsal view. Scale bars in millimeters.

#### Diagnosis.

This species is most similar to *Calliscelio brachys* in size and abbreviated notaulus. It may be distinguished from *Calliscelio brachys* in having A4 distinctly shorter than A3, A6 distinctly transverse in the female.

#### Etymology.

The specific epithet is to be treated as a noun in apposition, derived from the Latin for “be away,” and refers to the abbreviated notaulus.

#### Link to distribution map.

[http://hol.osu.edu/map-full.html?id=384780]

#### Material examined.

Holotype, female: **COLOMBIA**: Magdalena Dept., Zaino, M.567, 50m, 11°20'N 74°02'W, Tayrona Natural National Park, 28.VII–14.VIII.2000, Malaise trap, R. Henriquez, OSUC 190716 (deposited in IAVH). Paratypes: (146 females, 106 males) **BELIZE**: 42 females, 27 males, OSUC 534029 (CNCI); OSUC 185847, 185849–185851, 185854, 237727–237729, 246215–246217, 247494, 342738–342741, 397563, 398893, 47953, 47963, 47971, 48049–48052, 48054, 64034, 64038, 64041, 64043, 64073, 64077, 64090, 64096–64097, 64102, 64109, 64111, 64116, 91676, 91685, 91687, 91689, 91693, 91695–91696, 91699–91700, 91702, 93079, 93526, 93590–93593, 93714, 93735, 93743, 93745, 94041–94042, 94046–94047, 94083–94084, 94087–94089 (OSUC). **COLOMBIA**: 75 females, 59 males, OSUC 557540–557545, 557558, 557560, 557574–557575, 557586, 557629–557632 (CNCI); OSUC 188886, 188888, 189393–189394, 189397, 190667–190669, 190675–190676, 190679, 192201, 192203, 192206–192207, 192210, 192212–192213, 192217–192223, 192382–192384, 192391–192392, 192395–192396, 192398–192399, 194186–194188, 194190, 194192, 194195 (IAVH); OSUC 170498–170502, 189905, 189907–189909, 190096, 190099, 190101, 190711–190712, 190714, 190717–190718, 190993, 191058–191060, 191063, 191144, 191240, 191244, 191252, 191264–191266, 191269–191271, 191277, 191279–191280, 191282, 191740, 192208, 192224, 193702–193703, 193705, 193771, 193773–193776, 193808, 194191, 194197, 194199, 259569–259571, 259578, 259580–259581, 259584–259587, 259590–259591, 279453, 279455–279459, 364087–364089, 369978, 370059, 370061–370062, 370065–370067 (OSUC). **COSTA RICA**: 1 female, OSUC 532552 (CNCI). **HONDURAS**: 12 females, OSUC 399367, 399373, 399383, 399388–399390, 399406, 399408, 410435, 410440, 410443, 410447 (MZLU). **MEXICO**: 7 females, 13 males, OSUC 534463, 534465–534470, 534479–534482, 534487 (CNCI); OSUC 55922, 55928, 55937, 55949, 576983–576986 (OSUC). **TRINIDAD AND TOBAGO**: 2 females, OSUC 546012, 546025 (CNCI). **VENEZUELA**: 7 females, 7 males, OSUC 545849–545850, 545854, 545856–545857, 545870, 545900–545901, 545975–545978, 557666, 557677 (CNCI).

### Calliscelio alcoa

Taxon classificationAnimaliaHymenopteraPlatygastridae

Chen & Masner sp. n.

http://zoobank.org/A4769E20-E5A0-42CE-8768-D3C39939FC9D

http://bioguid.osu.edu/xbiod_concepts/362051

Figures 7–9 , 28–33

#### Description.

Body length of female: 2.86–3.10 mm (n=10). Body length of male: 2.42–2.54 mm (n=16). Color of head: orange throughout. Color of antennal clava (A7–A12): A7 dark orange, remainder dark brown to black. Shape of head: subglobose. Central keel of frons: absent. Setation of upper frons: with dense, short setae. IOS/EH: IOS distinctly less than EH. Sculpture of ventrolateral frons: granulate to finely punctate. Sculpture of frons below median ocellus: largely smooth with sparse fine punctures. Sculpture of posterior vertex: densely punctate. Hyperoccipital carina: absent. Occipital carina medially: interrupted. Length of OOL: less than 0.5× ocellar diameter. Sculpture of postgena behind outer orbit: smooth. Ocular setae: absent. A4 in female: distinctly shorter than A3. A5 in female: shorter than A3, distinctly longer than wide. Shape of female A6: distinctly longer than wide. Form of male antennal flagellomeres: filiform, A11 approximately 3.0× longer than wide. Length of A5 tyloid in male: approximately 0.3× length of A5.

Color of mesosoma in female: orange throughout. Color of mesosoma in male: orange throughout. Sculpture of dorsal pronotal area: rugose. Sculpture of lateral pronotal area: smooth anteriorly, punctate rugulose posteriorly. Sculpture of netrion: rugulose. Notaulus: percurrent. Sculpture of mesoscutum: densely punctate. Shape of mesoscutellum: semiellipsoidal. Foveolae of scutoscutellar sulcus between notauli: smaller than those along margin of axilla. Sculpture of mesoscutellum: smooth with sparse fine punctures. Shape of metascutellum: posterior margin somewhat rounded, approximately 4.0× wider than long. Sculpture of metascutellum in female: rugose anteriorly, smooth posteriorly. Sculpture of metascutellum in male: rugose. Dorsal propodeum in female: deeply excavate medially, with lateral propodeal carinae widely separated, running subparallel to accommodate T1 horn. Sculpture of dorsal propodeum in female: rugose. Sculpture of dorsal propodeum in male: rugose with one or two longitudinal keels lateral to median keel. Median keels on propodeum in female: absent. Mesopleural carina: present. Sculpture of mesepisternum below mesopleural depression: smooth. Sculpture of ventral metapleural area: smooth. Color of legs: pale yellow throughout. Sculpture of hind coxa: smooth.

Color of fore wing: hyaline. Rs+M: nebulose, weakly pigmented. Setae on R: long, erect, surpassing the margin of the wing. Length of R: approximately as long as r-rs. Length of R1: approximately as long as 2.0× length of r-rs.

Color of metasoma in female: yellow to dark brown. Color of metasoma in male: T3 yellow, otherwise brown to dark brown. Horn on T1 in female: large and distinct. Sculpture of T1 horn dorsally: densely and transversely striate. Sculpture of posterior margin of T1 in female: longitudinally striate throughout. Sculpture of T1 in male: longitudinally striate. Development of longitudinal striae on T2 in female: reaching posterior margin of T2. Sculpture of T3: smooth medially, longitudinally striate laterally. Shape of T6 in female: distinctly elongate, approximately 3.5× longer than wide. Sculpture of S3: largely smooth with sparse and fine punctures.

**Figures 28–33.**
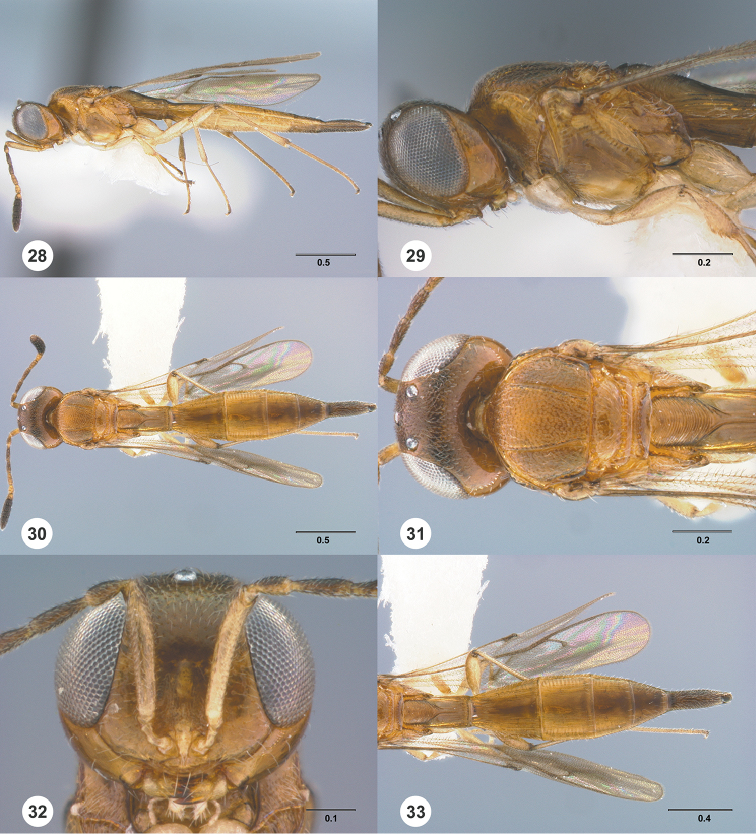
*Calliscelio alcoa* sp. n., female, holotype (OSUC 458212). **28** Lateral habitus **29** Head and mesosoma, lateral view **30** Dorsal habitus **31** Head and mesosoma, dorsal view **32** Head, anterior view **33** Metasoma, dorsal view. Scale bars in millimeters.

#### Diagnosis.

The medially interrupted occipital carina, densely punctate mesoscutum and densely and transversely striate T1 horn in the female distinguish this species of *Calliscelio* from all others.

#### Etymology.

The specific epithet refers to the locality on the label of the holotype and should be treated as a noun in apposition.

#### Link to distribution map.

[http://hol.osu.edu/map-full.html?id=362051]

#### Material examined.

Holotype, female: **DOMINICAN REPUBLIC**: Pedernales Prov., 25km of Alcoa Road, dry montane forest, Baoruco (Bahoruco) Mountains, 700m, 18.I.1989, L. Masner, OSUC 458212 (deposited in CNCI). Paratypes: **DOMINICAN REPUBLIC**: 9 females, 16 males, CMNH-486,529 (CMNH); OSUC 458213–458232, 458251, 458327–458328, 534379 (CNCI).

### Calliscelio amadoi

Taxon classificationAnimaliaHymenopteraPlatygastridae

Chen & Johnson sp. n.

http://zoobank.org/6571E5B9-E640-47B3-AD2C-D654DC26829F

http://bioguid.osu.edu/xbiod_concepts/384810

Figures 34–39

#### Description.

Body length of female: 1.35–2.05 mm (n=20). Color of head: brown throughout; yellow throughout; yellow, becoming darker at vertex. Color of antennal clava (A7–A12): A7 orange, remainder dark brown to black. Shape of head: subglobose. Central keel of frons: present. Setation of upper frons: with sparse, long setae. IOS/EH: IOS distinctly less than EH. Sculpture of ventrolateral frons: smooth with sparse punctures. Sculpture of frons below median ocellus: largely smooth with sparse fine punctures. Sculpture of posterior vertex: largely smooth with sparse fine punctures. Hyperoccipital carina: absent. Occipital carina medially: complete, weakly crenulate throughout. Length of OOL: less than 0.5× ocellar diameter. Sculpture of postgena behind outer orbit: smooth. Ocular setae: absent. A4 in female: distinctly shorter than A3. A5 in female: shorter than A3, slightly longer than wide. Shape of female A6: as long as wide.

Color of mesosoma in female: orange throughout; yellow throughout; dark brown. Sculpture of dorsal pronotal area: rugose. Sculpture of lateral pronotal area: smooth anteriorly, granulate posteriorly. Sculpture of netrion: smooth. Notaulus: percurrent or nearly so. Sculpture of mesoscutum: coriaceous. Shape of mesoscutellum: semiellipsoidal. Foveolae of scutoscutellar sulcus between notauli: as large as those along margin of axilla. Sculpture of mesoscutellum: smooth with sparse fine punctures. Shape of metascutellum: posterior margin straight, approximately 4.0× wider than long. Sculpture of metascutellum in female: smooth with a longitudinal, median carina. Dorsal propodeum in female: shallowly excavate medially, with lateral propodeal carinae widely separated. Sculpture of dorsal propodeum in female: rugose. Median keels on propodeum in female: absent. Mesopleural carina: absent. Sculpture of mesepisternum below mesopleural depression: smooth. Sculpture of ventral metapleural area: smooth. Color of legs: pale yellow throughout. Sculpture of hind coxa: smooth.

Color of fore wing: hyaline. Rs+M: nebulose, strongly pigmented. Setae on R: long, erect, surpassing the margin of the wing. Length of R: approximately as long as r-rs. Length of R1: greater than 3.0× length of r-rs.

Color of metasoma in female: orange to pale brown; orange throughout; yellow throughout. Horn on T1 in female: large and distinct. Sculpture of T1 horn dorsally: rugulose; transversely striate. Sculpture of posterior margin of T1 in female: longitudinally striate throughout. Development of longitudinal striae on T2 in female: present on anterior margin of T2 medially, reaching posterior margin of T2 laterally. Sculpture of T3: smooth with longitudinal submedian striae. Shape of T6 in female: short, approximately 1.5× longer than wide. Sculpture of S3: smooth.

**Figures 34–39.**
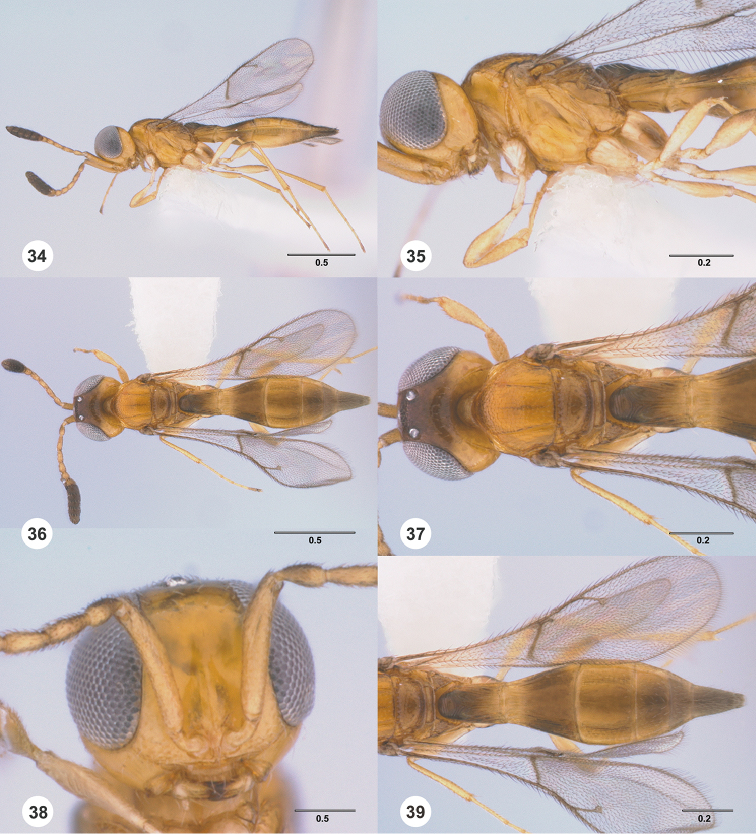
*Calliscelio amadoi* sp. n., female, holotype (OSUC 193589). **34** Lateral habitus **35** Head and mesosoma, lateral view **36** Dorsal habitus **37** Head and mesosoma, dorsal view **38** Head, anterior view **39** Metasoma, dorsal view. Scale bars in millimeters.

#### Diagnosis.

This species is most similar to *Calliscelio levis* and *Calliscelio migma*. It may be separated from *Calliscelio levis* by the well-developed T1 horn in the female, from *Calliscelio migma* by having A5 in female slightly longer than wide, and A6 quadrate, whereas both antennomeres are distinctly longer than wide in *Calliscelio migma*.

#### Etymology.

The epithet is used as a genitive noun derived from the name of the collector of the holotype.

#### Link to distribution map.

[http://hol.osu.edu/map-full.html?id=384810]

#### Material examined.

Holotype, female: **COLOMBIA**: Amazonas Dept., Amacayacu Natural N.P., M.840, 150m, 03°46'S 70°18'W, San Martín, 16.IX–24.IX.2000, Malaise trap, B. Amado, OSUC 193589 (deposited in IAVH). Paratypes: (50 females) **BRAZIL**: 4 females, OSUC 322670, 348261, 371852, 376063 (OSUC). **COLOMBIA**: 7 females, OSUC 191148, 193587 (IAVH); OSUC 178096, 191040, 259754, 262600, 276046 (OSUC). **ECUADOR**: 15 females, OSUC 458510, 458533, 534249–534251, 553237, 553242–553243, 553246, 553406–553407, 553530, 553651, 553687 (CNCI); OSUC 534659 (OSUC). **FRENCH GUIANA**: 10 females, OSUC 458384, 458413, 458421, 458459–458460, 458474, 546106–546108, 546135 (CNCI). **GRENADA**: 6 females, OSUC 534255–534260 (CNCI). **PERU**: 5 females, OSUC 553952, 553955, 553967, 554016, 554021 (CNCI). **TRINIDAD AND TOBAGO**: 3 females, OSUC 534601, 534605–534606 (CNCI).

### Calliscelio armila

Taxon classificationAnimaliaHymenopteraPlatygastridae

Chen & Masner sp. n.

http://zoobank.org/6AB94D9B-DEF6-4FE0-BC1B-5F5BAB7A15F1

http://bioguid.osu.edu/xbiod_concepts/362054

Figures 40–45

#### Description.

Body length of female: 2.12–2.51 mm (n=11). Body length of male: 2.05–2.27 mm (n=13). Color of head: black throughout; dark brown. Color of antennal clava (A7–A12): black. Shape of head: subglobose. Central keel of frons: absent. Setation of upper frons: with sparse, long setae. IOS/EH: IOS slightly less than EH. Sculpture of ventrolateral frons: smooth to rugulose. Sculpture of frons below median ocellus: largely smooth with sparse fine punctures. Sculpture of posterior vertex: coriaceous. Hyperoccipital carina: absent. Occipital carina medially: weakly developed, irregularly sculptured. Length of OOL: greater than 0.5× ocellar diameter. Sculpture of postgena behind outer orbit: coriaceous. Ocular setae: sparse, short. A4 in female: distinctly shorter than A3. A5 in female: shorter than A3, as long as wide. Shape of female A6: distinctly wider than long. Form of male antennal flagellomeres: filiform, A11 approximately 3.0× longer than wide. Length of A5 tyloid in male: approximately 0.3× length of A5.

Color of mesosoma in female: variably orange to pale brown. Color of mesosoma in male: orange throughout; variably orange to pale brown. Sculpture of dorsal pronotal area: rugose. Sculpture of lateral pronotal area: largely smooth, granulate ventrally and posteriorly. Sculpture of netrion: rugose. Notaulus: percurrent or nearly so. Sculpture of mesoscutum: granulate. Shape of mesoscutellum: semiellipsoidal. Foveolae of scutoscutellar sulcus between notauli: smaller than those along margin of axilla. Sculpture of mesoscutellum: granulate. Shape of metascutellum: posterior margin straight, approximately 3.5× wider than long. Sculpture of metascutellum in female: rugose anteriorly, smooth posteriorly. Sculpture of metascutellum in male: rugose. Dorsal propodeum in female: deeply excavate medially, with lateral propodeal carinae widely separated, running subparallel to accommodate T1 horn. Sculpture of dorsal propodeum in female: rugulose. Sculpture of dorsal propodeum in male: rugose. Median keels on propodeum in female: absent. Mesopleural carina: absent. Sculpture of mesepisternum below mesopleural depression: smooth. Sculpture of ventral metapleural area: smooth. Color of legs: coxae to femur white, remainder of the legs pale yellow. Sculpture of hind coxa: smooth.

Color of fore wing: hyaline. Rs+M: spectral. Setae on R: long, erect, surpassing the margin of the wing. Length of R: distinctly shorter than r-rs. Length of R1: approximately as long as 2.0× length of r-rs.

Color of metasoma in female: orange to pale brown. Color of metasoma in male: T3–T4 orange, otherwise brown. Horn on T1 in female: large and distinct. Sculpture of T1 horn dorsally: smooth to rugulose medially, with V-shaped keels laterally. Sculpture of posterior margin of T1 in female: longitudinally striate throughout. Sculpture of T1 in male: longitudinally striate. Development of longitudinal striae on T2 in female: present on the anterior margin of T2. Sculpture of T3: smooth. Shape of T6 in female: short, slightly longer than wide. Sculpture of S3: largely smooth with sparse and fine punctures.

**Figures 40–45.**
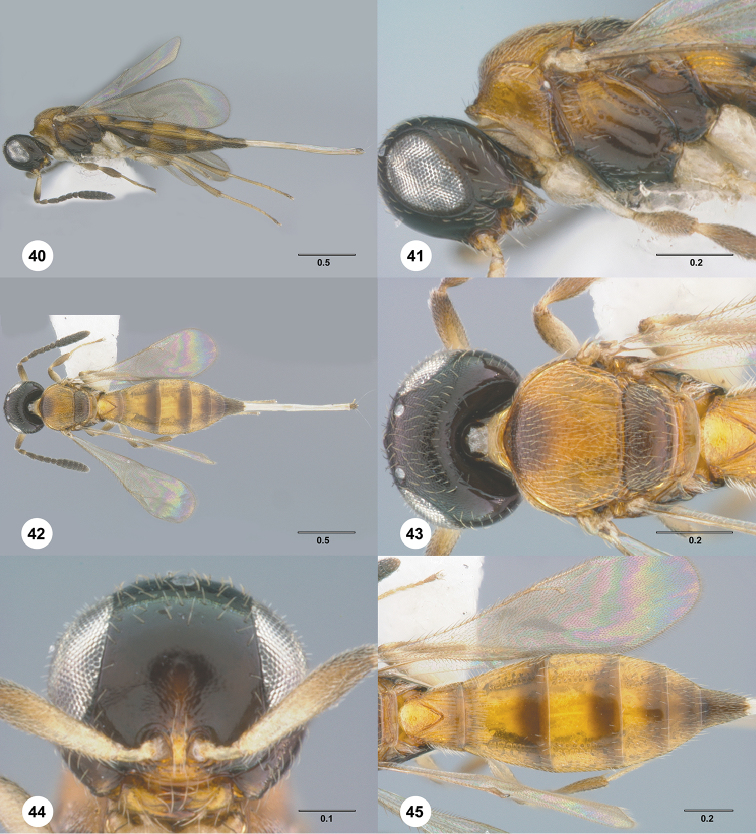
*Calliscelio armila* sp. n., female, holotype (OSUC 458342). **40** Lateral habitus **41** Head and mesosoma, lateral view **42** Dorsal habitus **43** Head and mesosoma, dorsal view **44** Head, anterior view **45** Metasoma, dorsal view. Scale bars in millimeters.

#### Diagnosis.

This species is most similar to *Calliscelio latifrons* in the female T1 horn, size and habitus but can be easily distinguished by its smooth upper frons and T3.

#### Etymology.

The specific epithet is to be treated as a noun in apposition, derived from the Latin for “bracelet” and refers to the rim on T1 horn.

#### Link to distribution map.

[http://hol.osu.edu/map-full.html?id=362054]

#### Material examined.

Holotype, female: **HAITI**: Sud Dept., Hotte Mts., S slope of Mt. Formon, 31km NW Les Cayes, disturbed forest & fields, 1405m, 18°20'N, 74°01'W, Formond, 7.IX–8.IX.1995, R. Davidson, G. Onore & J. Rawlins, OSUC 458342 (deposited in CNCI). Paratypes: **HAITI**: 10 females, 13 males, OSUC 458334–458341, 458343–458357 (CNCI).

### Calliscelio bidens

Taxon classificationAnimaliaHymenopteraPlatygastridae

Chen & Masner sp. n.

http://zoobank.org/0200D54C-C0FF-41DC-9D85-829CCD8BF6AE

http://bioguid.osu.edu/xbiod_concepts/362052

Figures 46–51

#### Description.

Body length of female: 3.35 mm (n=1). Color of head: brown throughout. Color of antennal clava (A7–A12): dark brown to black. Shape of head: subglobose. Central keel of frons: present. Setation of upper frons: with dense, short setae. IOS/EH: IOS distinctly less than EH. Sculpture of ventrolateral frons: smooth with sparse punctures. Sculpture of frons below median ocellus: granulate to finely punctate. Sculpture of posterior vertex: punctate rugose. Hyperoccipital carina: absent. Occipital carina medially: complete, strongly crenulate throughout. Length of OOL: greater than 0.5× ocellar diameter. Sculpture of postgena behind outer orbit: smooth. Ocular setae: sparse, short. A4 in female: as long as A3. A5 in female: shorter than A3, distinctly longer than wide. Shape of female A6: distinctly longer than wide.

Color of mesosoma in female: dark brown. Sculpture of dorsal pronotal area: areolate. Sculpture of lateral pronotal area: smooth dorsally, rugulose ventrally. Sculpture of netrion: rugulose. Notaulus: percurrent or nearly so. Sculpture of mesoscutum: densely punctate. Shape of mesoscutellum: semiellipsoidal. Foveolae of scutoscutellar sulcus between notauli: as large as those along margin of axilla. Sculpture of mesoscutellum: densely punctate. Shape of metascutellum: posterior marging rounded, approximately 4.0× wider than long. Sculpture of metascutellum in female: rugose. Dorsal propodeum in female: not excavate medially, lateral propodeal carinae meeting anteromedially. Sculpture of dorsal propodeum in female: rugose. Median keels on propodeum in female: present. Mesopleural carina: present. Sculpture of mesepisternum below mesopleural depression: smooth. Sculpture of ventral metapleural area: largely smooth, rugose ventrally. Color of legs: orange yellow. Sculpture of hind coxa: smooth.

Color of fore wing: hyaline. Rs+M: nebulose, strongly pigmented. Setae on R: long, erect, surpassing the margin of the wing. Length of R: distinctly shorter than r-rs. Length of R1: greater than 3.0× length of r-rs.

Color of metasoma in female: dark brown. Horn on T1 in female: absent. Sculpture of posterior margin of T1 in female: longitudinally striate throughout. Development of longitudinal striae on T2 in female: reaching posterior margin of T2. Sculpture of T3: smooth. Shape of T6 in female: short, slightly longer than wide. Sculpture of S3: smooth.

**Figures 46–51.**
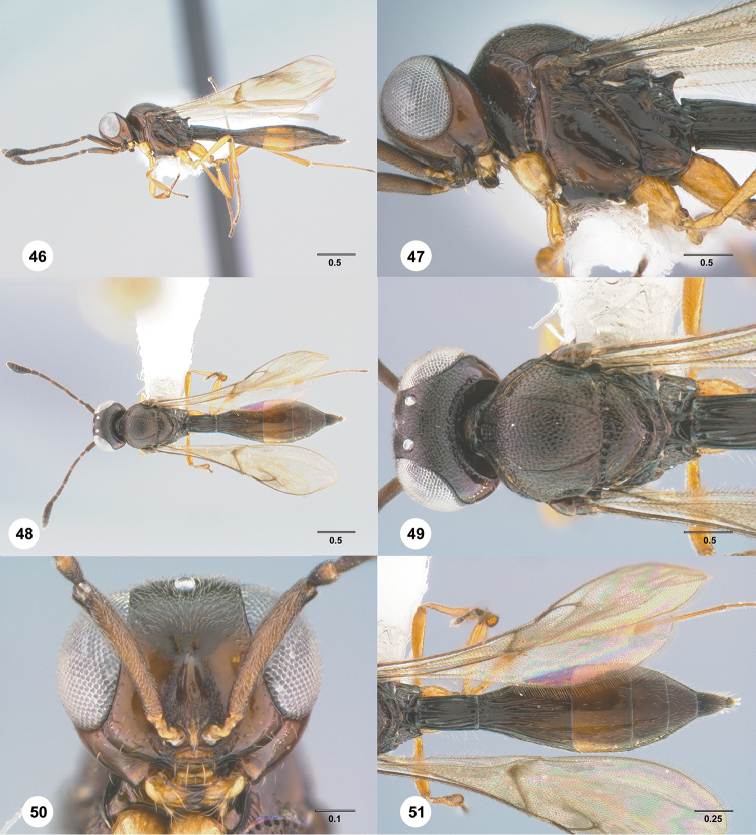
*Calliscelio bidens* sp. n., female, holotype (OSUC 458326). **46** Lateral habitus **47** Head and mesosoma, lateral view **48** Dorsal habitus **49** Head and mesosoma, dorsal view **50** Head, anterior view **51** Metasoma, dorsal view. Scale bars in millimeters.

#### Diagnosis.

This species is most similar to *Calliscelio rugicoxa* with which it shares the characters of the hairy compound eyes and the median keels on propodeum in female. It can be distinguished by its smooth hind coxa and the absence of a T1 horn in the female.

#### Etymology.

The specific epithet means “with two teeth,” refering to the median keels of propodeum of the species and should be treated as a noun in apposition.

#### Link to distribution map.

[http://hol.osu.edu/map-full.html?id=362052]

#### Material examined.

Holotype, female: **COLOMBIA**: Valle del Cauca Dept., 650m, 03°26'N, 76°48'W, Farallones de Cali Natural National Park, 8.V–19.VI.2001, Malaise trap, S. Sarria, OSUC 458326 (deposited in CNCI).

### Calliscelio bisulcatus

Taxon classificationAnimaliaHymenopteraPlatygastridae

(Kieffer)

http://zoobank.org/3C2921D8-CDFA-4CDA-86F2-C61304A4730B

http://bioguid.osu.edu/xbiod_concepts/4139

Figures 52–57

Baryconus bisulcatus Kieffer, 1910a: 320 (original description, keyed).
Baryconus (Baryconus) bisulcatus Kieffer: Kieffer 1910b: 84 (subgeneric assignment).
Glyptoteleia bisulcata (Kieffer): Kieffer 1926: 487 (generic transfer, description).
Calliscelio bisulcatus (Kieffer): Masner 1976: 38 (description, generic transfer, type information).

#### Description.

Body length of female: 1.75–2.18 mm (n=20). Body length of male: 1.70–2.12 mm (n=20). Color of head: black throughout; dark brown; orange throughout. Color of antennal clava (A7–A12): dark brown to black. Shape of head: subglobose. Central keel of frons: absent. Setation of upper frons: with sparse, long setae. IOS/EH: IOS distinctly less than EH. Sculpture of ventrolateral frons: smooth with sparse punctures. Sculpture of frons below median ocellus: largely smooth with sparse fine punctures. Sculpture of posterior vertex: granulate. Hyperoccipital carina: absent. Occipital carina medially: complete, strongly crenulate throughout. Length of OOL: less than 0.5× ocellar diameter. Sculpture of postgena behind outer orbit: granulate. Ocular setae: dense, long. A4 in female: distinctly shorter than A3. A5 in female: shorter than A3, as long as wide. Shape of female A6: distinctly wider than long. Form of male antennal flagellomeres: filiform, A11 approximately 2.0× longer than wide. Length of A5 tyloid in male: greater than 0.5× length of A5.

Color of mesosoma in female: orange throughout; variably orange to pale brown. Color of mesosoma in male: orange throughout; variably orange to pale brown. Sculpture of dorsal pronotal area: rugose. Sculpture of lateral pronotal area: smooth throughout. Sculpture of netrion: rugose. Notaulus: percurrent or nearly so. Sculpture of mesoscutum: smooth with sparse punctures. Shape of mesoscutellum: semiellipsoidal. Foveolae of scutoscutellar sulcus between notauli: as large as those along margin of axilla. Sculpture of mesoscutellum: smooth with sparse fine punctures. Shape of metascutellum: posterior margin straight, approximately 2.0× wider than long. Sculpture of metascutellum in female: rugose anteriorly, smooth posteriorly. Sculpture of metascutellum in male: rugose. Dorsal propodeum in female: shallowly excavate medially, with lateral propodeal carinae widely separated. Sculpture of dorsal propodeum in female: rugose. Sculpture of dorsal propodeum in male: rugose. Median keels on propodeum in female: absent. Mesopleural carina: present. Sculpture of mesepisternum below mesopleural depression: largely smooth with a row of foveae along mesopleural carina. Sculpture of ventral metapleural area: smooth dorsally, densely punctate ventrally. Color of legs: orange throughout. Sculpture of hind coxa: smooth.

Color of fore wing: hyaline. Rs+M: spectral. Setae on R: long, erect, surpassing the margin of the wing. Length of R: approximately as long as r-rs. Length of R1: approximately as long as r-rs.

Color of metasoma in female: orange throughout; variably orange to pale brown. Color of metasoma in male: variably orange to pale brown. Horn on T1 in female: present as a small bulge. Sculpture of T1 horn dorsally: smooth. Sculpture of posterior margin of T1 in female: longitudinally striate throughout. Sculpture of T1 in male: longitudinally striate. Development of longitudinal striae on T2 in female: reaching the middle of T2 medially. Sculpture of T3: smooth. Shape of T6 in female: short, slightly longer than wide. Sculpture of S3: smooth.

**Figures 52–57.**
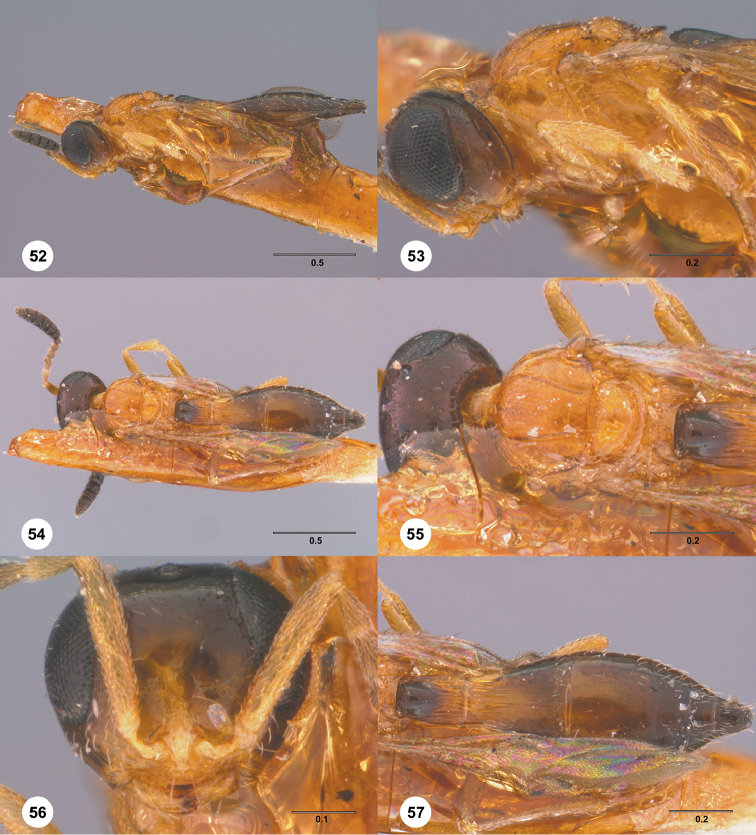
*Calliscelio bisulcatus* (Kieffer), female, holotype (CAS Type No. 9590). **52** Lateral habitus **53** Head and mesosoma, lateral view **54** Dorsal habitus **55** Head and mesosoma, dorsal view **56** Head, anterior view **57** Metasoma, dorsal view. Scale bars in millimeters.

#### Diagnosis.

This species is similar to *Calliscelio punctatifrons* in the shape of metascutellum and sculpture of T1 horn in the female but can be distinguished by its largely smooth upper frons, granulate posterior vertex and smaller body size.

#### Link to distribution map.

[http://hol.osu.edu/map-full.html?id=4139]

#### Material examined.

Holotype, female, *Baryconus bisulcatus*: **BRAZIL**: PA, no date, Baker, CAS Type No. 9590 (deposited in CASC). Other material: (216 females, 71 males) **ARGENTINA**: 8 females, 8 males, OSUC 534117–534124, 534436, 534440–534442 (CNCI); OSUC 63157 (OSUC); OSUC 577254, 577258–577259 (UCRC). **BELIZE**: 14 females, 1 male, OSUC 534192, 534199–534200, 534202, 534206–534210, 534302–534307 (CNCI). **BOLIVIA**: 6 females, 2 males, OSUC 534027–534028, 534044, 534049–534053 (CNCI). **BRAZIL**: 43 females, 23 males, OSUC 534521–534522, 557242, 557297, 557299, 557309–557310 (CNCI); OSUC 110142, 110175, 110182, 111340, 111379, 111382, 111522, 111527, 111531, 111633, 111693–111694, 111697, 111701, 111712–111713, 111920, 111922, 111939, 112040, 112070, 112090, 112186, 112453, 112781, 112806, 113027, 12276, 12356, 130739, 130748, 131475, 132140, 132282, 132534, 132690–132691, 132717, 133048, 133057, 133124, 134453, 134484, 137972, 138060, 232036, 374721–374722, 374726–374727, 48518, 48526, 48548, 48563, 48565, 48568, 48573, 55927, 813 (OSUC). **COLOMBIA**: 2 females, 3 males, OSUC 557536, 557576, 557624–557625 (CNCI); OSUC 369977 (OSUC). **COSTA RICA**: 10 females, 1 male, OSUC 532493, 532618–532622, 532646, 532718–532719, 532722, 532740 (CNCI). **ECUADOR**: 34 females, 10 males, OSUC 458496, 458498, 458500, 458503, 458505–458506, 458508, 458515, 458529, 534229, 534235, 534239–534240, 534242, 553262, 553386–553387, 553389, 553399, 553408–553410, 553497–553499, 553551, 553559, 553594–553595, 553622, 553625, 553634–553636, 553649–553650, 553681, 577329–577330, 577335 (CNCI); OSUC 534658, 534660–534661, 534672 (OSUC). **FRENCH GUIANA**: 15 females, OSUC 458392, 458398, 546112, 546119, 546122, 546127–546128, 546136–546138, 546140–546141, 546144–546145, 546148 (CNCI). **MEXICO**: 2 females, OSUC 534459 (CNCI); OSUC 377895 (OSUC). **PANAMA**: 3 females, OSUC 534083, 534085, 553877 (CNCI). **PARAGUAY**: 7 females, 18 males, OSUC 185325, 276696, 276729–276731, 276901–276907, 280295, 323025–323026, 323054–323055, 323067, 577193–577194, 577341, 577354, 583310–583311, 583314 (OSUC). **PERU**: 14 females, 1 male, OSUC 534389 (CNCI); OSUC 323933–323937, 323940–323943, 323945, 323947, 323949–323951 (OSUC). **SURINAME**: 19 females, OSUC 534562–534567, 534570–534571, 534573–534579, 534582, 553631–553633 (CNCI). **TRINIDAD AND TOBAGO**: 22 females, OSUC 545999, 546002–546003, 546013, 546027–546028, 546050–546052, 546057, 546077, 546082, 546086, 546088–546089, 546093–546096, 546099–546100, 553676 (CNCI). **URUGUAY**: 5 females, OSUC 534610–534614 (CNCI). **VENEZUELA**: 12 females, 4 males, OSUC 545881, 545884, 545887, 545893, 545895, 545897–545898, 545942–545943, 557648–557649, 557651–557654 (CNCI); OSUC 55923 (OSUC).

### Calliscelio brachys

Taxon classificationAnimaliaHymenopteraPlatygastridae

Chen & Johnson sp. n.

http://zoobank.org/D4F29FBA-909B-4DE6-969B-BBA983996BD5

http://bioguid.osu.edu/xbiod_concepts/384702

Figures 58–63

#### Description.

Body length of female: 1.52–1.85 mm (n=20). Body length of male: 1.45–1.88 mm (n=20). Color of head: orange throughout. Color of antennal clava (A7–A12): A7–A9 brown, A10–A12 yellow. Shape of head: subglobose. Central keel of frons: absent. Setation of upper frons: with sparse, short setae. IOS/EH: IOS distinctly less than EH. Sculpture of ventrolateral frons: smooth to coriaceous. Sculpture of frons below median ocellus: coriaceous. Sculpture of posterior vertex: smooth; smooth to transversely striate. Hyperoccipital carina: absent. Occipital carina medially: interrupted. Length of OOL: less than 0.5× ocellar diameter. Sculpture of postgena behind outer orbit: smooth. Ocular setae: absent. A4 in female: as long as A3. A5 in female: shorter than A3, distinctly longer than wide. Shape of female A6: as long as wide. Form of male antennal flagellomeres: filiform, A11 approximately 3.5× longer than wide. Length of A5 tyloid in male: approximately 0.3× length of A5.

Color of mesosoma in female: orange throughout; orange to pale brown. Color of mesosoma in male: orange throughout; variably orange to pale brown. Sculpture of dorsal pronotal area: smooth. Sculpture of lateral pronotal area: smooth throughout. Sculpture of netrion: smooth. Notaulus: abbreviated, at most reaching middle of mesoscutum. Sculpture of mesoscutum: granulate. Shape of mesoscutellum: semiellipsoidal. Foveolae of scutoscutellar sulcus between notauli: smaller than those along margin of axilla. Sculpture of mesoscutellum: granulate. Shape of metascutellum: posterior margin rounded, approximately 2.5× wider than long. Sculpture of metascutellum in female: rugulose with a longitudinal, median carina. Sculpture of metascutellum in male: rugose. Dorsal propodeum in female: not excavate medially, lateral propodeal carinae meeting anteromedially. Sculpture of dorsal propodeum in female: rugose. Sculpture of dorsal propodeum in male: rugose with one or two longitudinal keels lateral to median keel. Median keels on propodeum in female: absent. Mesopleural carina: present. Sculpture of mesepisternum below mesopleural depression: smooth. Sculpture of ventral metapleural area: largely smooth with an oblique carina. Color of legs: orange throughout; pale yellow throughout. Sculpture of hind coxa: smooth.

Color of fore wing: hyaline. Rs+M: spectral. Setae on R: long, erect, surpassing the margin of the wing. Length of R: distinctly shorter than r-rs. Length of R1: greater than 3.0× length of r-rs.

Color of metasoma in female: orange throughout; variably orange to pale brown. Color of metasoma in male: variably orange to pale brown. Horn on T1 in female: absent. Sculpture of posterior margin of T1 in female: longitudinally striate throughout. Sculpture of T1 in male: longitudinally striate. Development of longitudinal striae on T2 in female: present on the anterior margin of T2. Sculpture of T3: smooth. Shape of T6 in female: short, slightly longer than wide. Sculpture of S3: smooth.

**Figures 58–63.**
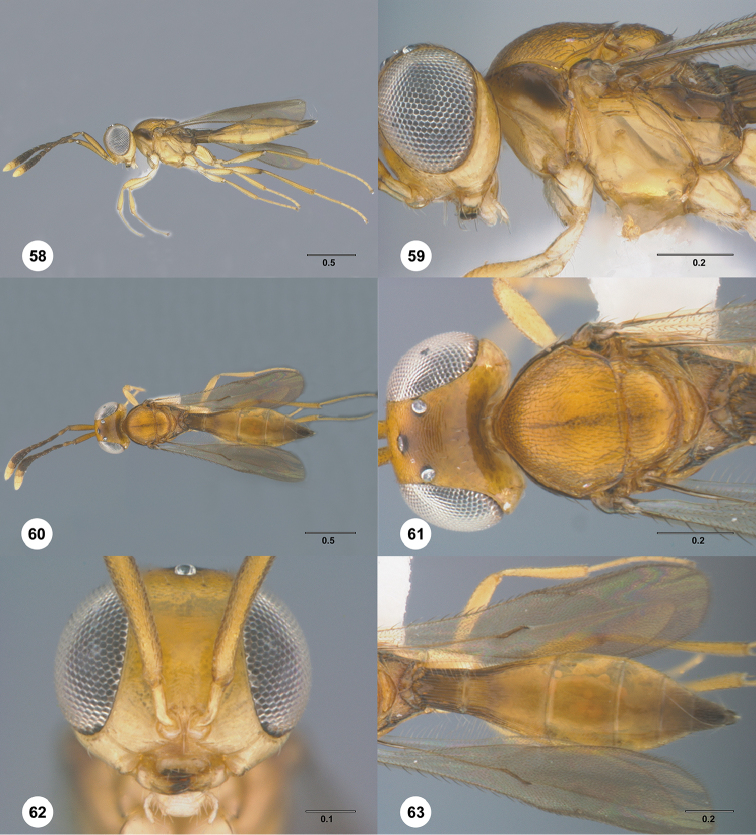
*Calliscelio brachys* sp. n., female, holotype (OSUC 193349). **58** Lateral habitus **59** Head and mesosoma, lateral view **60** Dorsal habitus **61** Head and mesosoma, dorsal view **62** Head, anterior view **63** Metasoma, dorsal view. Scale bars in millimeters.

#### Diagnosis.

This species shares the character of the abbreviated notaulus with *Calliscelio absum* and *Calliscelio brevinotaulus*. It can be separated from *Calliscelio absum* by having A4 as long as A3 in the female and the length of the A5 tyloid in the male is approximately 0.3× length of A5; from *Calliscelio brevinotaulus* it may be distinguished by the absence of the T1 horn in the female.

#### Etymology.

The epithet is derived the Greek word for short, in reference to the abbreviated notaulus, and is intended to be treated as a noun in apposition.

#### Link to distribution map.

[http://hol.osu.edu/map-full.html?id=384702]

#### Material examined.

Holotype, female: **COLOMBIA**: Amazonas Dept., Amacayacu Natural N.P., M.842, 150m, 03°46'S 70°18'W, San Martín, 16.VIII–24.VIII.2000, Malaise trap, B. Amado, OSUC 193349 (deposited in IAVH). Paratypes: (74 females, 26 males) **BOLIVIA**: 1 female, OSUC 534149 (CNCI). **COLOMBIA**: 63 females, 24 males, OSUC 557548, 557582 (CNCI); OSUC 152149–152150, 152152, 152154, 182229, 182592, 188678, 188682, 188955, 189186, 189190–189191, 189193, 189195, 189226, 193282, 193284, 193541, 193570, 193584, 193597, 193903, 231811, 249895, 253463, 259760, 259762, 272083, 272087, 275804–275805, 276184, 276236, 276244–276245, 279657, 363591, 364075 (IAVH); OSUC 152162, 162499, 162501, 162509, 162584, 162586–162587, 162594, 162596, 162599, 162606–162607, 162609, 170495, 182596, 182722–182723, 182740, 188551, 188941, 188944, 188963, 189183, 189267–189268, 189271, 189273, 189279, 191094, 191150, 192353, 192358, 193320–193321, 193329, 193338, 193340, 193538, 193598–193599, 193814, 231829, 267807, 267962, 280198, 372644–372645 (OSUC). **ECUADOR**: 5 females, 1 male, OSUC 458488, 458497, 458536, 534237, 553561, 577338 (CNCI). **PERU**: 5 females, 1 male, OSUC 553970, 553972, 554010, 554023–554024, 554032 (CNCI).

### Calliscelio brevinotaulus

Taxon classificationAnimaliaHymenopteraPlatygastridae

Chen & Johnson sp. n.

http://zoobank.org/9D986838-195F-44DD-BF84-3D933502679D

http://bioguid.osu.edu/xbiod_concepts/363559

Figures 64–69

#### Description.

Body length of female: 1.78–2.98 mm (n=20). Body length of male: 1.78–2.41 mm (n=20). Color of head: black throughout; variably orange to dark brown. Color of antennal clava (A7–A12): dark brown to black. Shape of head: subglobose. Central keel of frons: absent. Setation of upper frons: with sparse, short setae. IOS/EH: IOS distinctly less than EH. Sculpture of ventrolateral frons: transversely rugulose to granulate. Sculpture of frons below median ocellus: granulate. Sculpture of posterior vertex: granulate. Hyperoccipital carina: absent. Occipital carina medially: interrupted. Length of OOL: less than 0.5× ocellar diameter. Sculpture of postgena behind outer orbit: largely smooth with small granulate area. Ocular setae: absent. A4 in female: distinctly shorter than A3. A5 in female: shorter than A3, as long as wide. Shape of female A6: subquadrate. Form of male antennal flagellomeres: filiform, approximately 2.0× longer than wide. Length of A5 tyloid in male: greater than 0.5× length of A5.

Color of mesosoma in female: orange throughout; variably orange to pale brown. Color of mesosoma in male: orange throughout; variably orange to pale brown. Sculpture of dorsal pronotal area: rugose. Sculpture of lateral pronotal area: smooth throughout; smooth anteriorly, granulate posteriorly. Sculpture of netrion: rugose. Notaulus: abbreviated, at most reaching middle of mesoscutum. Sculpture of mesoscutum: granulate. Shape of mesoscutellum: semiellipsoidal. Foveolae of scutoscutellar sulcus between notauli: smaller than those along margin of axilla. Sculpture of mesoscutellum: granulate. Shape of metascutellum: posterior margin rounded, 2.5× wider than long. Sculpture of metascutellum in female: rugose. Sculpture of metascutellum in male: rugose. Dorsal propodeum in female: shallowly excavate medially, with lateral propodeal carinae widely separated. Sculpture of dorsal propodeum in female: rugose. Sculpture of dorsal propodeum in male: rugose with one or two longitudinal keels lateral to median keel. Median keels on propodeum in female: absent. Mesopleural carina: absent. Sculpture of mesepisternum below mesopleural depression: smooth. Sculpture of ventral metapleural area: largely smooth, rugose ventrally. Color of legs: orange throughout; mid and hind coxae dark brown to black, otherwise yellow throughout. Sculpture of hind coxa: smooth.

Color of fore wing: hyaline. Rs+M: spectral. Setae on R: long, erect, surpassing the margin of the wing. Length of R: distinctly shorter than r-rs. Length of R1: greater than 3.0× length of r-rs.

Color of metasoma in female: orange throughout; variably orange to pale brown. Color of metasoma in male: orange throughout; variably orange to pale brown. Horn on T1 in female: weakly indicated. Sculpture of T1 horn dorsally: with V-shaped striae. Sculpture of posterior margin of T1 in female: longitudinally striate throughout. Sculpture of T1 in male: longitudinally striate. Development of longitudinal striae on T2 in female: reaching posterior margin of T2. Sculpture of T3: smooth; largely smooth with submedian longitudinal striae. Shape of T6 in female: short, slightly longer than wide. Sculpture of S3: largely smooth with sparse and fine punctures.

**Figures 64–69.**
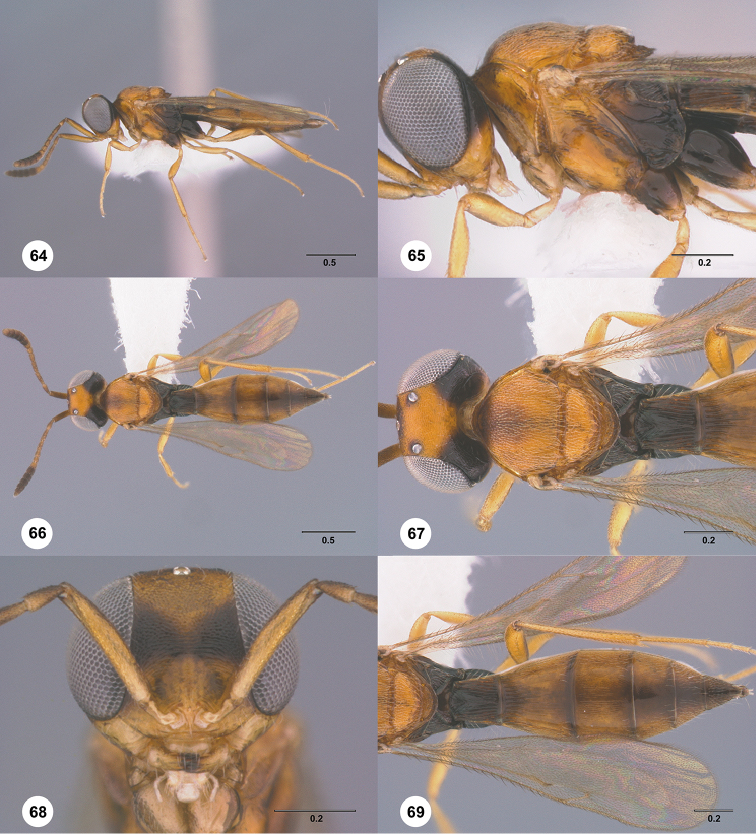
*Calliscelio brevinotaulus* sp. n., female, holotype (OSUC 190678). **64** Lateral habitus **65** Head and mesosoma, lateral view **66** Dorsal habitus **67** Head and mesosoma, dorsal view **68** Head, anterior view **69** Metasoma, dorsal view. Scale bars in millimeters.

#### Diagnosis.

This species is most similar to *Calliscelio brachys* in color, size and the abbreviated notaulus but can be distinguished by the granulate upper frons and that A4 is shorter than A3.

#### Etymology.

The epithet refers to the short notauli in this species and is intended to be used as a noun in apposition.

#### Link to distribution map.

[http://hol.osu.edu/map-full.html?id=363559]

#### Material examined.

Holotype, female: **COLOMBIA**: Magdalena Dept., Zaino, M.564, 50m, 11°20'N 74°02'W, Tayrona Natural National Park, 14.VIII–30.VIII.2000, Malaise trap, R. Henriquez, OSUC 190678 (deposited in IAVH). Paratypes: (264 females, 99 males) **BELIZE**: 7 females, 5 males, OSUC 534277, 534279, 534283–534284, 534290–534292, 534301, 534308, 534310–534311, 534313 (CNCI). **BRAZIL**: 29 females, 1 male, OSUC 348007, 348118, 353716, 363846, 366704–366705, 371850, 374564, 374566, 374593, 376520–376521, 380186, 577137 (MZSP); OSUC 326244, 347202, 347274, 347666, 347738, 347969, 348098, 349378, 349773, 351396, 351679, 351831–351832, 351839–351840, 351843 (OSUC). **COLOMBIA**: 70 females, 61 males, OSUC 557546, 557554, 557559, 557561, 557569–557573, 557584, 557621, 557628, 557634–557639 (CNCI); OSUC 170449–170452, 170497, 170503–170504, 170506, 188889, 189906, 189910, 190095, 190097–190098, 190100, 190124, 190706–190707, 190709, 190986, 190988–190989, 190992, 190994, 191057, 191061, 191143, 191739, 191742–191743, 191745, 192204, 192211, 192214, 193379, 193701, 193706–193709, 193772, 259573, 259583, 269666, 273785, 364080–364086, 370058, 370060, 370063–370064 (IAVH); OSUC 188887, 188890, 189390–189391, 189396, 189398, 190666, 190672–190673, 190677, 190680–190681, 190683, 191064, 191241–191242, 191245, 191247, 191249–191250, 191253–191258, 191268, 191272–191276, 191278, 192202, 192215–192216, 192385–192390, 192394, 193704, 193778, 194185, 194189, 194194, 194200, 259572, 259574, 259577, 259579, 259582, 259588–259589, 279454 (OSUC). **COSTA RICA**: 52 females, 22 males, OSUC 532464, 532479–532486, 532506, 532516, 532518–532519, 532523–532526, 532532, 532565, 532572–532574, 532577–532578, 532583, 532585–532588, 532590–532591, 532602–532608, 532611, 532632–532634, 532643, 532665–532666, 532675–532676, 532678–532681, 532685, 532687, 532698–532700, 532717, 532720–532721, 532732, 532734–532735, 532737–532739, 532751–532752, 532756–532757, 532788, 532790, 532834, 532919 (CNCI); OSUC 237329 (OSUC). CURAÇAO: 2 males, OSUC 532749–532750 (CNCI). **ECUADOR**: 30 females, OSUC 458501–458502, 458532, 458543–458544, 534228, 553383–553384, 553396, 553398, 553404, 553417, 553483, 553500, 553537, 553548, 553558, 553591, 553593, 553626, 553629, 553637–553639, 553679, 553683, 553710, 553712, 553737, 553746 (CNCI). **HONDURAS**: 4 females, OSUC 534142–534144, 534146 (CNCI). **MEXICO**: 19 females, 4 males, OSUC 533982–533997, 534001–534003, 534025, 534460, 534488–534489 (CNCI). **PANAMA**: 26 females, 2 males, OSUC 534066–534068, 534082, 534084, 534101–534102, 553773–553775, 553808–553809, 553814, 553816, 553820, 553872, 553904, 553919, 553926–553927, 553938–553940, 553944–553947 (CNCI); OSUC 55955 (OSUC). **PERU**: 1 female, OSUC 554025 (CNCI). **SURINAME**: 1 female, OSUC 534585 (CNCI). **VENEZUELA**: 25 females, 2 males, OSUC 532724, 545833–545834, 545847–545848, 545851, 545864–545868, 545879–545880, 545899, 545958–545959, 545968–545973, 557656, 557658, 557673 (CNCI); OSUC 334558, 360696 (OSUC).

### Calliscelio brevitas

Taxon classificationAnimaliaHymenopteraPlatygastridae

Chen & Johnson sp. n.

http://zoobank.org/10B4E8E2-0970-4E2A-85EC-7B373111AFDF

http://bioguid.osu.edu/xbiod_concepts/367290

Figures 70–75

#### Description.

Body length of female: 1.83–2.88 mm (n=20). Color of head: black throughout; orange throughout; variably orange to dark brown. Color of antennal clava (A7–A12): A7 dark orange, remainder dark brown to black; dark brown to black. Shape of head: subglobose. Central keel of frons: present. Setation of upper frons: with sparse, short setae. IOS/EH: IOS distinctly less than EH. Sculpture of ventrolateral frons: granulate; smooth with sparse punctures. Sculpture of frons below median ocellus: largely smooth with sparse fine punctures. Sculpture of posterior vertex: densely punctate; granulate to rugulose. Hyperoccipital carina: absent. Occipital carina medially: complete, weakly crenulate throughout. Length of OOL: less than 0.5× ocellar diameter. Sculpture of postgena behind outer orbit: smooth. Ocular setae: absent. A4 in female: distinctly shorter than A3. A5 in female: shorter than A3, distinctly longer than wide. Shape of female A6: as long as wide.

Color of mesosoma in female: orange throughout; black throughout; variably orange to pale brown. Sculpture of dorsal pronotal area: rugose. Sculpture of lateral pronotal area: smooth throughout; largely smooth, granulate ventrally and posteriorly. Sculpture of netrion: smooth; rugose. Notaulus: percurrent or nearly so. Sculpture of mesoscutum: largely coriaceous with dense and fine punctures at poster end. Shape of mesoscutellum: semiellipsoidal. Foveolae of scutoscutellar sulcus between notauli: as large as those along margin of axilla. Sculpture of mesoscutellum: smooth with sparse fine punctures. Shape of metascutellum: posterior margin straight, approximately 4.0× wider than long. Sculpture of metascutellum in female: rugose. Sculpture of metascutellum in male: rugose. Dorsal propodeum in female: deeply excavate medially, with lateral propodeal carinae widely separated, running subparallel to accommodate T1 horn. Sculpture of dorsal propodeum in female: rugose. Sculpture of dorsal propodeum in male: rugose. Median keels on propodeum in female: absent. Mesopleural carina: absent. Sculpture of mesepisternum below mesopleural depression: smooth. Sculpture of ventral metapleural area: smooth; largely smooth, rugose ventrally. Color of legs: pale yellow throughout; coxae pale yellow, otherwise orange to pale brown. Sculpture of hind coxa: smooth.

Color of fore wing: hyaline. Rs+M: nebulose, strongly pigmented. Setae on R: long, erect, surpassing the margin of the wing. Length of R: approximately as long as r-rs. Length of R1: greater than 3.0× length of r-rs.

Color of metasoma in female: variably yellow to pale brown. Color of metasoma in male: brown throughout; reddish brown. Horn on T1 in female: large and distinct. Sculpture of T1 horn dorsally: transversely striate. Sculpture of posterior margin of T1 in female: longitudinally striate throughout. Sculpture of T1 in male: longitudinally striate. Development of longitudinal striae on T2 in female: reaching posterior margin of T2; present on anterior margin of T2 medially, reaching posterior margin of T2 laterally. Sculpture of T3: smooth with longitudinal submedian striae. Shape of T6 in female: distinctly elongate, at least 2.0× longer than wide. Sculpture of S3: densely punctate.

**Figures 70–75.**
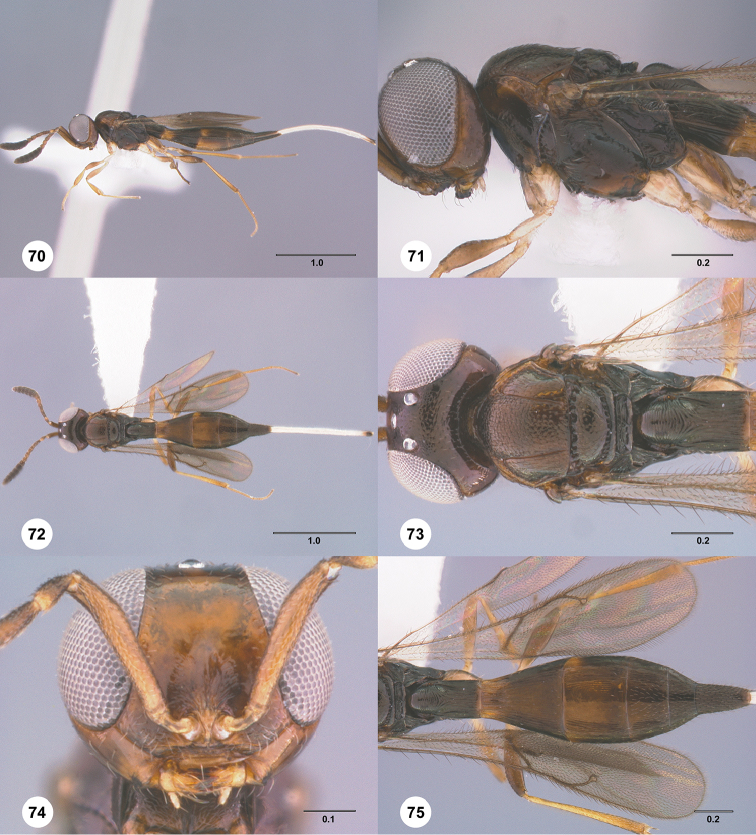
*Calliscelio brevitas* sp. n., female, holotype (OSUC 190705). **70** Lateral habitus **71** Head and mesosoma, lateral view **72** Dorsal habitus **73** Head and mesosoma, dorsal view **74** Head, anterior view **75** Metasoma, dorsal view. Scale bars in millimeters.

#### Diagnosis.

This species is most similar to *Calliscelio laticinctus* and *Calliscelio longius* in color, size and habitus. It may be distinguished from them by having A6 quadrate in female, whereas A6 is distinctly longer than wide in *Calliscelio laticinctus* and *Calliscelio longius*.

#### Etymology.

The epithet is used as a noun in apposition derived from the Latin word for shortness, in reference to the shortened A5 and A6.

#### Link to distribution map.

[http://hol.osu.edu/map-full.html?id=367290]

#### Material examined.

Holotype, female: **COLOMBIA**: Magdalena Dept., Zaino, M.567, 50m, 11°20'N 74°02'W, Tayrona Natural National Park, 28.VII–14.VIII.2000, Malaise trap, R. Henriquez, OSUC 190705 (deposited in IAVH). Paratypes: (355 females) **BRAZIL**: 11 females, OSUC 534537, 534539 (CNCI); OSUC 121827, 252086, 323928, 326208, 348271, 349206, 355169, 376519, 376528 (OSUC). **COLOMBIA**: 126 females, OSUC 557562–557564, 557568, 557581, 557583, 557590, 557592–557597, 557603, 557626–557627 (CNCI); OSUC 144164, 144230, 162601, 166586, 170369–170371, 178098, 178162, 178174, 178192–178193, 178195, 190125, 191183, 191206, 191212, 191243, 191248, 191381, 191383, 193128, 193130, 193179, 193193, 193294, 193322, 193326, 193328, 193863–193864, 193866–193868, 193879, 193906, 193910, 193913, 262616, 267805, 363594, 76997 (IAVH); OSUC 178021, 178191, 182228, 182482, 182754–182756, 182762, 188688, 188726, 188730, 188951, 188954, 189176, 189199, 189277, 189282, 189285, 189287, 189291–189292, 189297, 190310, 191096, 191098, 191827, 192354–192355, 192359, 193166, 193293, 193560, 193563, 193565–193566, 193572, 193576, 193689–193691, 194196, 202078, 232297–232298, 253454–253455, 268911, 269217, 269350, 269356, 275800, 275803, 275808, 275811, 279350, 279899, 279903, 279908–279909, 279911, 280179, 280181, 280195, 280204–280205, 363600, 374718, 377424 (OSUC). **COSTA RICA**: 133 females, OSUC 532458, 532503, 532535, 532570, 532616–532617, 532627–532628, 532630–532631, 532635, 532637–532639, 532660, 532771, 532774, 532784–532785, 532825, 532841–532845, 532847–532848, 532850, 532852–532868, 532870–532872, 532875–532885, 532887–532888, 532891–532892, 532899–532901, 532903–532916, 532928, 532935, 532938–532940, 532942–532949, 534138, 557113–557132, 557134–557139, 557142–557154 (CNCI). **ECUADOR**: 29 females, OSUC 458494, 553255, 553376, 553440, 553443, 553461, 553469–553471, 553473, 553478, 553510–553511, 553513–553514, 553518, 553524–553526, 553540, 553542, 553565, 553568–553570, 553590 (CNCI); OSUC 534663, 534668, 534670 (OSUC). **FRENCH GUIANA**: 25 females, OSUC 458393–458397, 458404–458405, 458410–458411, 458423, 458427, 458444–458446, 458462, 458464, 458469, 458472–458473, 458478, 458480–458481, 546104, 546132, 546149 (CNCI). **PANAMA**: 17 females, OSUC 534100, 553747, 553756, 553767–553770, 553782, 553784, 553810, 553905, 553915, 553924–553925, 553930–553932 (CNCI). **PERU**: 12 females, OSUC 534416, 553991, 553993, 554035, 554043, 554047, 554049 (CNCI); OSUC 570539–570540, 570546 (OSUC); OSUC 228135, 228191 (USNM). **SURINAME**: 1 female, OSUC 534583 (CNCI). **VENEZUELA**: 1 female, OSUC 557696 (CNCI).

### Calliscelio carinigena

Taxon classificationAnimaliaHymenopteraPlatygastridae

Chen & Johnson sp. n.

http://zoobank.org/ADB95787-EBBB-4A36-B75D-5CE10D93826F

http://bioguid.osu.edu/xbiod_concepts/363281

Figures 76–81

#### Description.

Body length of female: 1.70–2.46 mm (n=20). Body length of male: 2.00–2.29 mm (n=20). Color of head: black throughout; orange throughout; variably brown to black. Color of antennal clava (A7–A12): dark brown to black. Shape of head: subglobose. Central keel of frons: absent. Setation of upper frons: with sparse, long setae. IOS/EH: IOS distinctly less than EH. Sculpture of ventrolateral frons: smooth to granulate. Sculpture of frons below median ocellus: granulate. Sculpture of posterior vertex: granulate to rugulose above hyperoccipital carina, smooth below. Hyperoccipital carina: present. Occipital carina medially: interrupted. Length of OOL: less than 0.5× ocellar diameter. Sculpture of postgena behind outer orbit: with a carina along outer orbit. Ocular setae: absent. A4 in female: distinctly shorter than A3. A5 in female: shorter than A3, as long as wide. Shape of female A6: distinctly wider than long. Form of male antennal flagellomeres: filiform, A11 approximately 3.0× longer than wide. Length of A5 tyloid in male: longer than 0.5× length of A5.

Color of mesosoma in female: orange throughout. Color of mesosoma in male: orange throughout; variably orange to pale brown. Sculpture of dorsal pronotal area: rugose. Sculpture of lateral pronotal area: smooth throughout. Sculpture of netrion: smooth. Notaulus: percurrent or nearly so. Sculpture of mesoscutum: granulate. Shape of mesoscutellum: semiellipsoidal. Foveolae of scutoscutellar sulcus between notauli: smaller than those along margin of axilla. Sculpture of mesoscutellum: granulate. Shape of metascutellum: posterior margin rounded, 2.5× wider than long. Sculpture of metascutellum in female: rugose. Sculpture of metascutellum in male: rugose. Dorsal propodeum in female: shallowly excavate medially, with lateral propodeal carinae widely separated. Sculpture of dorsal propodeum in female: rugose. Sculpture of dorsal propodeum in male: rugose. Median keels on propodeum in female: absent. Mesopleural carina: present. Sculpture of mesepisternum below mesopleural depression: smooth. Sculpture of ventral metapleural area: largely smooth, rugose ventrally. Color of legs: orange throughout; pale yellow throughout. Sculpture of hind coxa: smooth.

Color of fore wing: hyaline. Rs+M: spectral. Setae on R: long, erect, surpassing the margin of the wing. Length of R: distinctly shorter than r-rs. Length of R1: greater than 3.0× length of r-rs.

Color of metasoma in female: orange throughout; yellow throughout; T1 horn and T6 pale brown, otherwise orange throughout. Color of metasoma in male: orange throughout; variably orange to pale brown. Horn on T1 in female: weakly developed. Sculpture of T1 horn dorsally: smooth to somewhat transversely striate. Sculpture of posterior margin of T1 in female: smooth medially, longitudinally striate laterally. Sculpture of T1 in male: longitudinally striate. Development of longitudinal striae on T2 in female: reaching the middle of T2 medially. Sculpture of T3: smooth. Shape of T6 in female: short, wider than long. Sculpture of S3: smooth.

**Figures 76–81.**
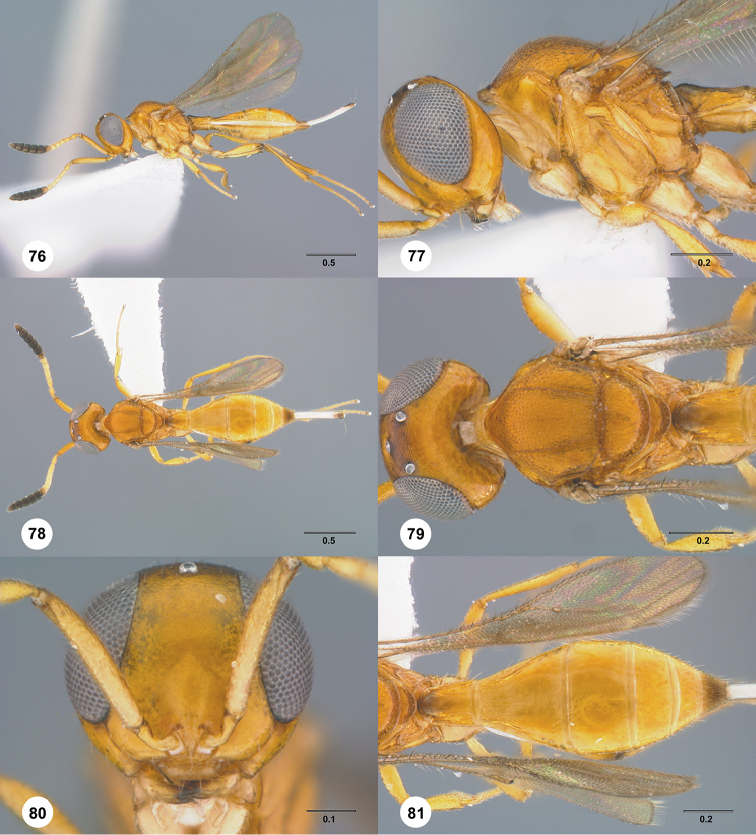
*Calliscelio carinigena* sp. n., female, holotype (OSUC 343907). **76** Lateral habitus **77** Head and mesosoma, lateral view **78** Dorsal habitus **79** Head and mesosoma, dorsal view **80** Head, anterior view **81** Metasoma, dorsal view. Scale bars in millimeters.

#### Diagnosis.

This species is most similar to *Calliscelio crater* and *Calliscelio sfina*. It can be separated from *Calliscelio crater* by the presence of a carina along the outer orbit on the postgena rather than large foveae, and from *Calliscelio sfina* by the presence of the hyperoccipital carina.

#### Etymology.

The epithet is a compound noun in reference to the genal carina along the outer orbit. It is intended to be used as a noun in apposition.

#### Link to distribution map.

[http://hol.osu.edu/map-full.html?id=363281]

#### Material examined.

Holotype, female: **BRAZIL**: SP, Pilões Trail, Boracéia Biological Station, pt.4, 23°39'05.0"S 45°53'40.1"W, Salesópolis, 19.X–22.X.2002, yellow pan trap, A. P. Aguiar & J. S. Freitas, OSUC 343907 (deposited in MZSP). Paratypes: (941 females, 591 males) **ARGENTINA**: 1 female, OSUC 534443 (CNCI). **BOLIVIA**: 4 females, 1 male, OSUC 534041–534043, 534056, 534064 (CNCI). **BRAZIL**: 929 females, 582 males, OSUC 557167–557179, 557182–557187, 557189–557196, 557210–557219, 557221–557241, 557244–557245, 557247, 557249–557253, 557256–557281, 557284–557295, 557300–557308, 557311–557317, 557322–557324, 557326–557336, 557339, 557341, 557343–557348, 557350–557359, 557363–557374, 557378–557394, 557396–557408 (CNCI); OSUC 577148, 577309–577311, 577313, 577379–577381, 577384–577387, 577389 (MNHN); OSUC 135930, 135955, 136029, 136037, 136420, 136453, 136953, 136964, 137063, 137244, 137263, 137306, 137403, 137408, 137429, 137694–137695, 137702, 137711–137712, 137740, 137743, 137753, 137762, 137766, 137768, 137771, 137775, 137777, 137806, 137890, 137894, 137912, 137916, 137927, 138530, 138536, 138542–138543, 138546, 138693, 138699–138701, 138721–138722, 138724, 138894, 138987, 139006, 139016, 139029, 150129–150152, 150154–150155, 150157, 150208–150250, 150265–150279, 150281–150285, 150357, 150371–150372, 150393–150394, 150419, 237725–237726, 318263–318266, 318268–318276, 318278–318280, 318961–318963, 319014–319021, 319023–319027, 319080–319139, 319340–319348, 319390–319400, 320901, 322525–322527, 322529–322531, 322541–322543, 322550–322551, 323094–323098, 323286, 323308, 323324, 323328, 323336, 343702, 343899–343906, 343908–343913, 343915–343931, 345915–345917, 345919, 345922, 345925, 345938, 345945, 345955, 345958–345959, 345962–345965, 345967–345968, 345970–345978, 345980–345983, 345985–345988, 345991, 345997, 346002, 346004–346006, 346011–346012, 346021, 346056–346057, 346059, 346062, 346068, 346072–346075, 346095, 346097–346099, 346112–346113, 349591, 349593, 352850–352856, 352859–352861, 353270–353272, 353870–353873, 356331, 356946–356947, 356949–356950, 356967–356969, 356973, 356975, 356982, 357249, 357251, 357253–357254, 357259–357261, 357264, 357319, 363768–363773, 363880, 366659, 371817–371823, 371825–371827, 372536, 372539, 373870–373872, 374591, 374599–374621, 375255, 375261, 375288, 375290, 375293, 375296, 376556, 376921, 376959, 377461–377465, 377467–377480, 377933–377959, 377961–377968, 377970–377977, 378977, 378982–378984, 381077–381078, 381080–381085, 433842, 433844, 433852, 576982, 577005–577013, 577034–577035, 577048, 577058–577059, 577064–577066, 577071, 577097, 577100, 577113–577115, 577122–577124, 577217, 577221–577223, 577228–577229, 577237–577245, 577262–577267, 577269–577271, 577277–577278, 577281–577282, 577284–577286, 577289, 577294, 583206–583208, 583213–583225, 583233, 583235, 583240, 583242, 583259–583265, 583269, 583281–583288, 583293–583295, 583297 (MZSP); OSUC 135931, 135938, 135952–135953, 135966, 135979, 135989–135990, 136007, 136009, 136125, 136128, 136135, 136190, 136192, 136195, 136217, 136225, 136242, 136364–136365, 136368, 136370, 136373, 136375, 136379–136380, 136392, 136423, 136450, 136511–136512, 136519, 136526, 136529, 136535, 136547–136548, 136550, 136560, 136567, 136571, 136618, 136628, 136638, 136646, 136648, 136659, 136669, 136696, 136705, 136709, 136735–136736, 136760–136761, 136785, 136834, 136839–136840, 136843, 136849, 136852, 136858, 136864–136865, 136874, 136881, 136883, 136892, 136906, 136910, 136913, 136919, 136928, 136933, 136950–136951, 136959, 136962, 136967, 136969–136970, 136972, 136983, 136986, 136998, 137005, 137008, 137010, 137013, 137017–137018, 137027, 137054–137055, 137059, 137068–137069, 137075, 137079, 137084, 137119, 137134, 137173–137174, 137178, 137180, 137185, 137188–137189, 137200, 137206, 137212–137213, 137223–137224, 137227, 137230, 137234–137235, 137238, 137241, 137246, 137248, 137250, 137256, 137311, 137314, 137336, 137342, 137344–137345, 137349, 137354, 137357, 137361–137362, 137368–137370, 137372, 137379, 137384, 137393, 137398–137401, 137409, 137413, 137419–137423, 137428, 137434–137435, 137438, 137441–137442, 137448, 137455, 137507, 137509, 137523, 137525, 137531, 137533–137535, 137543, 137547–137549, 137551, 137555, 137564–137565, 137573, 137576, 137581, 137587, 137591, 137606–137609, 137646, 137649–137650, 137653–137654, 137656, 137660–137661, 137663–137664, 137668, 137685, 137718, 137729–137730, 137738, 137745, 137788, 137796, 137809, 137822, 137834, 137840, 137849, 137852–137853, 137861, 137863, 137865, 137873, 137880, 137886–137887, 137889, 137899, 138421, 138426, 138431–138432, 138434–138435, 138438, 138441–138445, 138448–138449, 138452, 138455, 138460–138462, 138465, 138468–138469, 138471, 138473, 138478, 138484, 138489, 138508–138509, 138515, 138520–138521, 138525, 138553–138554, 138560, 138562, 138566–138568, 138573, 138575, 138583, 138585–138586, 138593, 138597, 138613–138615, 138622, 138627, 138631–138632, 138636–138637, 138639, 138641, 138643, 138647, 138651, 138669–138670, 138677–138678, 138680, 138682, 138685, 138688, 138691–138692, 138705, 138710, 138713, 138715, 138725, 138737, 138739, 138743, 138746, 138749–138750, 138753, 138755–138757, 138760–138761, 138770–138771, 138774, 138776, 138783, 138786, 138788, 138791, 138805, 138808, 138813, 138815, 138819, 138821–138822, 138824, 138828, 138833, 138841, 138846, 138857, 138873, 138876, 138878, 138880, 138889–138890, 138892, 138895–138896, 138898, 138900, 138906, 138922, 138925, 138942, 138955, 138958, 138982, 139013, 139015, 139017, 139021, 139065, 139067, 139079–139080, 139089, 139091, 139093–139094, 139096, 139102, 139106, 139109, 139113, 139117–139119, 139121, 139130–139131, 139133, 139136–139137, 139143, 139146–139147, 139152, 139157–139159, 139164–139166, 139168, 139172, 139175, 139179–139180, 150158, 150160–150163, 150696–150700, 150736–150737, 150743, 150964, 150985, 151153–151154, 151163, 151189–151193, 151216–151223, 151225–151226, 151247–151252, 151254, 151267, 151270–151271, 151290–151293, 151309, 151344–151345, 151368–151373, 151384, 151386, 151398–151399, 151410, 151452–151480, 151488, 151492–151494, 151498, 151506, 151517–151519, 151526, 151593, 151629–151632, 319022, 322988, 323099, 323319, 323526, 334296–334298, 343914, 346078, 352843–352845, 352847–352849, 359023–359025, 365999–366000, 366656, 367439, 377481, 377960, 378585, 378591–378592, 40024, 40036, 40046, 40061, 40071, 40099, 40164, 40175, 40303, 42295, 42306–42309, 42312, 42315, 42320, 42328–42330, 42334, 42338, 42342–42343, 42354, 42361, 42363–42364, 42367–42368, 42375, 42378–42379, 427454, 433823, 433832, 433837, 463268, 463307–463308, 510886, 510888, 510893, 55909–55921, 55957–55997, 577015–577016, 577018, 577020, 583270–583280, 583298–583304, 583306–583307, 58793, 61197, 61236, 62250, 62261, 62268, 62284, 62287, 62301, 62312, 62333, 62348 (OSUC). **COLOMBIA**: 3 males, OSUC 162611, 191322, 191324 (OSUC). **ECUADOR**: 4 males, OSUC 534673–534675, 534679 (OSUC). **PARAGUAY**: 1 female, 1 male, OSUC 278829, 577183 (OSUC). **TRINIDAD AND TOBAGO**: 2 females, OSUC 546058, 546060 (CNCI). **VENEZUELA**: 4 females, OSUC 545888, 545891–545892, 557655 (CNCI).

### Calliscelio crater

Taxon classificationAnimaliaHymenopteraPlatygastridae

Chen & Johnson sp. n.

http://zoobank.org/3643BD0B-9BF3-4C41-A861-2AB72C701CA3

http://bioguid.osu.edu/xbiod_concepts/363278

Figures 82–87

#### Description.

Body length of female: 1.74–2.35 mm (n=20). Body length of male: 1.86–2.36 mm (n=20). Color of head: black throughout; variably orange to dark brown. Color of antennal clava (A7–A12): dark brown to black. Shape of head: subglobose. Central keel of frons: absent. Setation of upper frons: with sparse, long setae. IOS/EH: IOS distinctly less than EH. Sculpture of ventrolateral frons: smooth to granulate. Sculpture of frons below median ocellus: granulate. Sculpture of posterior vertex: granulate above hyperoccipital carina, smooth to rugulose below. Hyperoccipital carina: present. Occipital carina medially: interrupted. Length of OOL: less than 0.5× ocellar diameter. Sculpture of postgena behind outer orbit: with large foveae. Ocular setae: absent. A4 in female: distinctly shorter than A3. A5 in female: shorter than A3, as long as wide. Shape of female A6: subquadrate. Form of male antennal flagellomeres: filiform, A11 approximately 2.0× longer than wide. Length of A5 tyloid in male: greater than 0.5× length of A5.

Color of mesosoma in female: orange throughout. Color of mesosoma in male: orange throughout; variably orange to pale brown. Sculpture of dorsal pronotal area: rugose. Sculpture of lateral pronotal area: smooth anteriorly, granulate posteriorly. Sculpture of netrion: smooth. Notaulus: percurrent or nearly so. Sculpture of mesoscutum: granulate. Shape of mesoscutellum: semiellipsoidal. Foveolae of scutoscutellar sulcus between notauli: smaller than those along margin of axilla. Sculpture of mesoscutellum: granulate. Shape of metascutellum: posterior margin straight, approximately 2.5× wider than long. Sculpture of metascutellum in female: rugose. Sculpture of metascutellum in male: rugose. Dorsal propodeum in female: shallowly excavate medially, with lateral propodeal carinae widely separated. Sculpture of dorsal propodeum in female: rugose. Sculpture of dorsal propodeum in male: rugose. Median keels on propodeum in female: absent. Mesopleural carina: absent. Sculpture of mesepisternum below mesopleural depression: smooth. Sculpture of ventral metapleural area: largely smooth, rugose ventrally. Color of legs: orange throughout. Sculpture of hind coxa: smooth.

Color of fore wing: hyaline. Rs+M: spectral. Setae on R: long, erect, surpassing the margin of the wing. Length of R: distinctly shorter than r-rs. Length of R1: approximately as long as 2.0× length of r-rs.

Color of metasoma in female: orange throughout; T1 horn and T6 pale brown, otherwise orange throughout. Color of metasoma in male: variably orange to pale brown. Horn on T1 in female: present as a small bulge. Sculpture of T1 horn dorsally: rugose medially, with V-shaped keels laterally. Sculpture of posterior margin of T1 in female: longitudinally striate throughout. Sculpture of T1 in male: longitudinally striate. Development of longitudinal striae on T2 in female: reaching the middle of T2 medially. Sculpture of T3: coriaceous; granulate; smooth medially, coriaceous laterally; longitudinally striate throughout. Shape of T6 in female: short, slightly longer than wide. Sculpture of S3: smooth to coriaceous.

**Figures 82–87.**
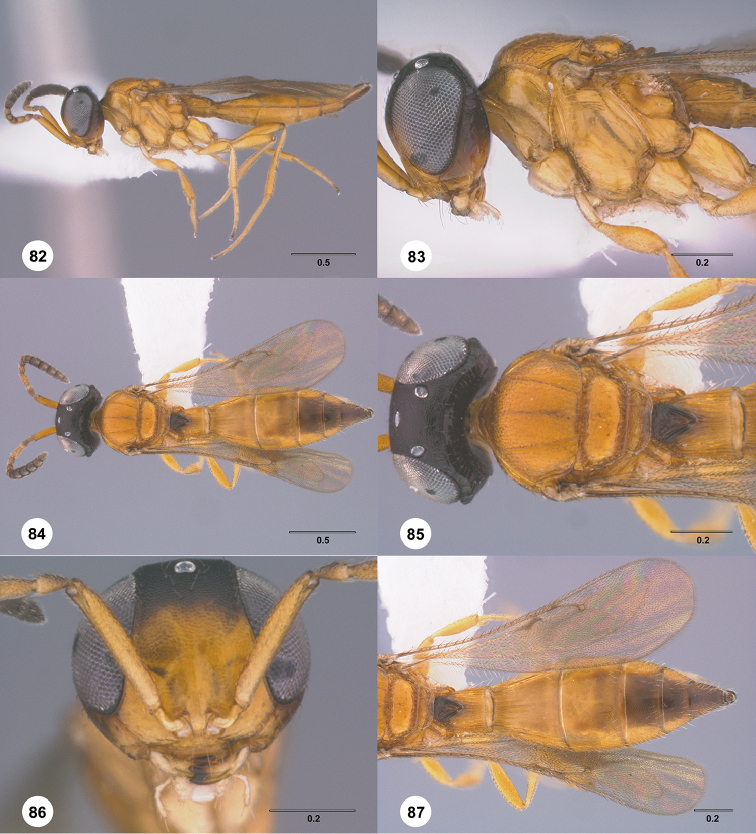
*Calliscelio crater* sp. n., female, holotype (OSUC 276803). **82** Lateral habitus **83** Head and mesosoma, lateral view **84** Dorsal habitus **85** Head and mesosoma, dorsal view **86** Head, anterior view **87** Metasoma, dorsal view. Scale bars in millimeters.

#### Diagnosis.

This species is similar to *Calliscelio carinigena* and *Calliscelio sfina* in color, size and habitus, but it can be easily recognized by the large foveae on the postgena (postgena of *Calliscelio sfina* granulate, whereas *Calliscelio carinigena* with a carina along outer orbit).

#### Etymology.

The specific epithet refers to the foveae on the postgena and should be treated as a noun in apposition.

#### Link to distribution map.

[http://hol.osu.edu/map-full.html?id=363278]

#### Material examined.

Holotype, female: **PARAGUAY**: Guairá Dept., Amambay Stream, Pa'i Thermal Waterfall, second growth forest, 221m, 25°44'56"S 56°15'12"W, Melgarejo, 24.III–25.III.2005, yellow pan trap, Garcete, OSUC 276803 (deposited in MNHNPY). Paratypes: (228 females, 322 males) **BOLIVIA**: 8 females, OSUC 534035–534037, 534039, 534057, 534059, 534161, 534165 (CNCI). **BRAZIL**: 202 females, 276 males, OSUC 534519, 534524, 534546–534547, 534621 (CNCI); OSUC 577298, 577305 (MNHN); OSUC 10431, 10463, 10584, 10625, 10833, 10857, 110188, 110198, 131683, 134141, 134350, 134445, 134795, 134802, 134829, 134845, 135140, 135614, 135652-135653, 137956, 137964, 137982, 138015, 138041, 138116, 138181, 147790, 151160, 151162, 232033, 232035, 323264–323266, 323269–323271, 323273–323274, 323276–323279, 323281, 323283–323285, 323288–323291, 323296–323297, 323299–323301, 323303–323304, 323311–323316, 323318, 323320, 323322, 323326, 323332–323335, 323525, 323529–323530, 323533–323537, 323539–323541, 323543, 323545, 323548, 323550, 323553–323557, 323561–323562, 323564–323565, 323914, 323918, 356945, 357216–357223, 357226–357228, 357257–357258, 357262–357263, 357273, 357278, 358790, 358944, 358984, 375277, 375285–375287, 375295, 375300, 376550–376551, 376553, 376555, 376947–376954, 376956–376958, 376960–376961, 376964, 433819, 510906, 534641–534642, 534647, 577019, 577022–577031, 577036, 577039–577042, 577044–577045, 577047, 577049–577057, 577062, 577091–577093, 577095, 577098, 577102, 577109–577112, 577198, 577205–577210, 577212–577214, 577218–577219, 577230–577233, 577235–577236, 577249–577252, 577268, 577276, 577279–577280, 577283, 577288, 577290–577292, 577295, 583228–583229, 583234, 583237–583239, 583241, 583243–583245, 583267–583268 (MZSP); OSUC 10135, 10363, 10467, 10473, 10523–10524, 10527, 10572–10573, 10581, 10702, 10712, 12201, 12262, 12266, 12325, 131326, 131681, 131720, 131771, 131825, 131923, 131942, 131997, 134140, 134185, 134523, 134565, 134682, 13550, 137264, 137987, 138000, 138479, 13902, 150741–150742, 150744, 150746–150747, 150749–150750, 150956, 150959, 150974, 150984, 151097, 151152, 151155–151158, 151161, 151266, 151268–151269, 151346, 151383, 151385, 151387, 151401–151404, 151416–151423, 151485–151487, 151549, 159, 187, 206, 216, 323511–323512, 323514, 323516, 323523–323524, 323912, 323919, 347618–347619, 349106, 357241–357242, 357247–357248, 357250, 357252, 357312–357313, 357315–357318, 357320, 357323, 357325, 358786–358788, 358942, 358974, 358981–358983, 358992, 363867, 363869–363875, 363877–363879, 363883–363887, 363889–363891, 366657, 366665, 372528, 372530–372534, 372538, 372540–372544, 375246, 375248–375250, 375252, 375254, 375256–375260, 375262, 375264–375268, 375270–375276, 375278–375284, 375297, 376552, 376557–376564, 376922, 376924–376929, 376931–376932, 376934, 376936–376940, 376944–376946, 376965, 477163–477164, 48517, 48537, 48543, 48552, 510889–510892, 510894–510898, 510900, 510905, 534643–534645, 534649, 534651–534656, 539, 577021, 577046, 712 (OSUC). **PARAGUAY**: 17 females, 46 males, OSUC 348870, 404893–404894, 534698, 534701, 534703, 534705, 534708–534712, 534715–534719, 577175, 577184–577187, 577339, 577348, 577351–577353 (MNHNPY); OSUC 577177, 577181–577182, 577190, 577342 (MZSP); OSUC 150604, 150608–150609, 150612, 185381, 266183, 276794–276795, 276797, 276799–276802, 277313–277314, 278659–278660, 278671–278672, 278680, 278827, 278831, 280439, 323004, 323030–323031, 323033, 323913, 353273, 534729–534730 (OSUC). **VENEZUELA**: 1 female, OSUC 545855 (CNCI).

### Calliscelio crena

Taxon classificationAnimaliaHymenopteraPlatygastridae

Chen & Johnson sp. n.

http://zoobank.org/C53F1631-C068-4EF9-AB3D-39BA37F7A09F

http://bioguid.osu.edu/xbiod_concepts/384634

Figures 88–93

#### Description.

Body length of female: 2.95–3.51 mm (n=20). Body length of male: 2.24–2.37 mm (n=6). Color of head: black throughout; dark brown. Color of antennal clava (A7–A12): dark brown to black. Shape of head: subglobose. Central keel of frons: absent. Setation of upper frons: with sparse, long setae. IOS/EH: IOS distinctly less than EH. Sculpture of ventrolateral frons: smooth with sparse punctures. Sculpture of frons below median ocellus: largely smooth with sparse fine punctures. Sculpture of posterior vertex: largely smooth with sparse fine punctures. Hyperoccipital carina: absent. Occipital carina medially: complete, strongly crenulate throughout. Length of OOL: less than 0.5× ocellar diameter. Sculpture of postgena behind outer orbit: smooth. Ocular setae: absent. A4 in female: distinctly shorter than A3. A5 in female: shorter than A3, distinctly longer than wide. Shape of female A6: as long as wide. Form of male antennal flagellomeres: filiform, A11 approximately 2.0× longer than wide. Length of A5 tyloid in male: greater than 0.5× length of A5.

Color of mesosoma in female: brown. Color of mesosoma in male: brown throughout. Sculpture of dorsal pronotal area: rugose. Sculpture of lateral pronotal area: smooth anteriorly, granulate posteriorly. Sculpture of netrion: rugulose. Notaulus: percurrent or nearly so. Sculpture of mesoscutum: anterior margin rugulose, remainder smooth. Shape of mesoscutellum: semiellipsoidal. Foveolae of scutoscutellar sulcus between notauli: as large as those along margin of axilla. Sculpture of mesoscutellum: smooth with sparse fine punctures. Shape of metascutellum: posterior margin straight, approximately 4.0× wider than long. Sculpture of metascutellum in female: rugose. Sculpture of metascutellum in male: rugose. Dorsal propodeum in female: deeply excavate medially, with lateral propodeal carinae widely separated, running subparallel to accommodate T1 horn. Sculpture of dorsal propodeum in female: rugose. Sculpture of dorsal propodeum in male: rugose with one or two longitudinal keels lateral to median keel. Median keels on propodeum in female: absent. Mesopleural carina: absent. Sculpture of mesepisternum below mesopleural depression: smooth. Sculpture of ventral metapleural area: largely smooth, rugose ventrally. Color of legs: orange throughout; coxae to femurs pale yellow, otherwise orange. Sculpture of hind coxa: smooth.

Color of fore wing: hyaline. Rs+M: nebulose, strongly pigmented. Setae on R: long, erect, surpassing the margin of the wing. Length of R: approximately as long as r-rs. Length of R1: greater than 3.0× length of r-rs.

Color of metasoma in female: dark brown. Color of metasoma in male: brown throughout. Horn on T1 in female: large and distinct. Sculpture of T1 horn dorsally: transversely striate. Sculpture of posterior margin of T1 in female: longitudinally striate throughout. Sculpture of T1 in male: longitudinally striate. Development of longitudinal striae on T2 in female: reaching posterior margin of T2. Sculpture of T3: smooth with longitudinal submedian striae. Shape of T6 in female: distinctly elongate, approximately 3.0× longer than wide. Sculpture of S3: smooth.

**Figures 88–93.**
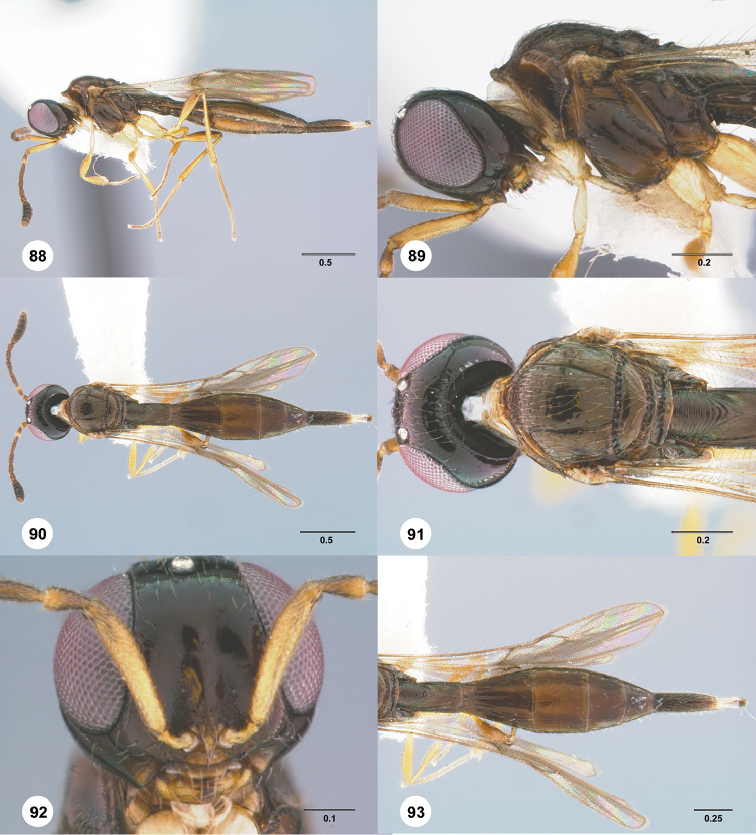
*Calliscelio crena* sp. n., female, holotype (OSUC 553462). **88** Lateral habitus **89** Head and mesosoma, lateral view **90** Dorsal habitus **91** Head and mesosoma, dorsal view **92** Head, anterior view **93** Metasoma, dorsal view. Scale bars in millimeters.

#### Diagnosis.

This species is most similar to *Calliscelio brevitas* in color, size and habitus but can be distinguished by its strongly crenulate occipital carina and relatively smooth posterior vertex (*Calliscelio brevitas* with occipital carina weakly crenulate, posterior vertex densely punctate).

#### Etymology.

The epithet is used as a noun in apposition derived from the Latin word for notch, in reference to the strongly crenulate occipital carina medially.

#### Link to distribution map.

[http://hol.osu.edu/map-full.html?id=384634]

#### Material examined.

Holotype, female: **ECUADOR**: Santo Domingo de los Tsáchilas Prov., 16km SE Santo Domingo de los Colorados, Tinalandia, 680m, 4.V–25.VII.1985, malaise trap/flight intercept trap, S. Peck & J. Peck, OSUC 553462 (deposited in CNCI). Paratypes: (28 females, 6 males) **COLOMBIA**: 3 females, OSUC 534555, 557567 (CNCI); OSUC 279904 (OSUC). **ECUADOR**: 25 females, 6 males, OSUC 534227, 553367, 553373, 553382, 553437–553439, 553450–553451, 553456, 553464–553466, 553468, 553547, 553600, 553653, 553669, 553675, 553680, 553692–553699, 553705, 553707, 553717 (CNCI).

### Calliscelio eboris

Taxon classificationAnimaliaHymenopteraPlatygastridae

Chen & Johnson sp. n.

http://zoobank.org/71300D0B-D933-4E9B-A689-0C7EA098F418

http://bioguid.osu.edu/xbiod_concepts/384800

Figures 94–99

#### Description.

Body length of female: 1.83–2.27 mm (n=20). Body length of male: 1.94–2.19 mm (n=20). Color of head: variably pale yellow to brown; reddish orange throughout. Color of antennal clava (A7–A12): A7–A9 brown, A10–A12 white; A7 brown, remainder orange. Shape of head: subglobose. Central keel of frons: present. Setation of upper frons: with sparse, long setae. IOS/EH: IOS distinctly less than EH. Sculpture of ventrolateral frons: smooth to coriaceous. Sculpture of frons below median ocellus: coriaceous. Sculpture of posterior vertex: transversely striate. Hyperoccipital carina: present. Occipital carina medially: interrupted. Length of OOL: less than 0.5× ocellar diameter. Sculpture of postgena behind outer orbit: smooth. Ocular setae: absent. A4 in female: as long as A3. A5 in female: shorter than A3, distinctly longer than wide. Shape of female A6: as long as wide. Form of male antennal flagellomeres: filiform, A11 approximately 3.0× longer than wide. Length of A5 tyloid in male: greater than 0.5× length of A5.

Color of mesosoma in female: variably yellow to pale brown; reddish orange throughout. Color of mesosoma in male: orange throughout; variably orange to pale brown. Sculpture of dorsal pronotal area: rugose. Sculpture of lateral pronotal area: smooth throughout. Sculpture of netrion: smooth. Notaulus: percurrent or nearly so. Sculpture of mesoscutum: coriaceous. Shape of mesoscutellum: semiellipsoidal. Foveolae of scutoscutellar sulcus between notauli: as large as those along margin of axilla. Sculpture of mesoscutellum: smooth with sparse fine punctures. Shape of metascutellum: posterior margin rounded, approximately 3.0× wider than long. Sculpture of metascutellum in female: smooth with a longitudinal, median carina. Sculpture of metascutellum in male: smooth with longitudinal, median carina. Dorsal propodeum in female: not excavate medially, lateral propodeal carinae meeting anteromedially. Sculpture of dorsal propodeum in female: rugose with one or two longitudinal keels lateral median keel. Sculpture of dorsal propodeum in male: rugose with one or two longitudinal keels lateral median keel. Median keels on propodeum in female: absent. Mesopleural carina: present. Sculpture of mesepisternum below mesopleural depression: smooth. Sculpture of ventral metapleural area: largely smooth, rugose ventrally. Color of legs: orange throughout; pale yellow throughout. Sculpture of hind coxa: smooth.

Color of fore wing: hyaline. Rs+M: spectral. Setae on R: short, decumbent, hardly exceeding the margin of the wing. Length of R: distinctly shorter than r-rs. Length of R1: greater than 3.0× length of r-rs.

Color of metasoma in female: orange throughout; variably orange to pale brown. Color of metasoma in male: variably orange to pale brown. Horn on T1 in female: absent. Sculpture of posterior margin of T1 in female: longitudinally striate throughout. Sculpture of T1 in male: longitudinally striate. Development of longitudinal striae on T2 in female: reaching the middle of T2 medially. Sculpture of T3: smooth. Shape of T6 in female: short, wider than long. Sculpture of S3: smooth.

**Figures 94–99.**
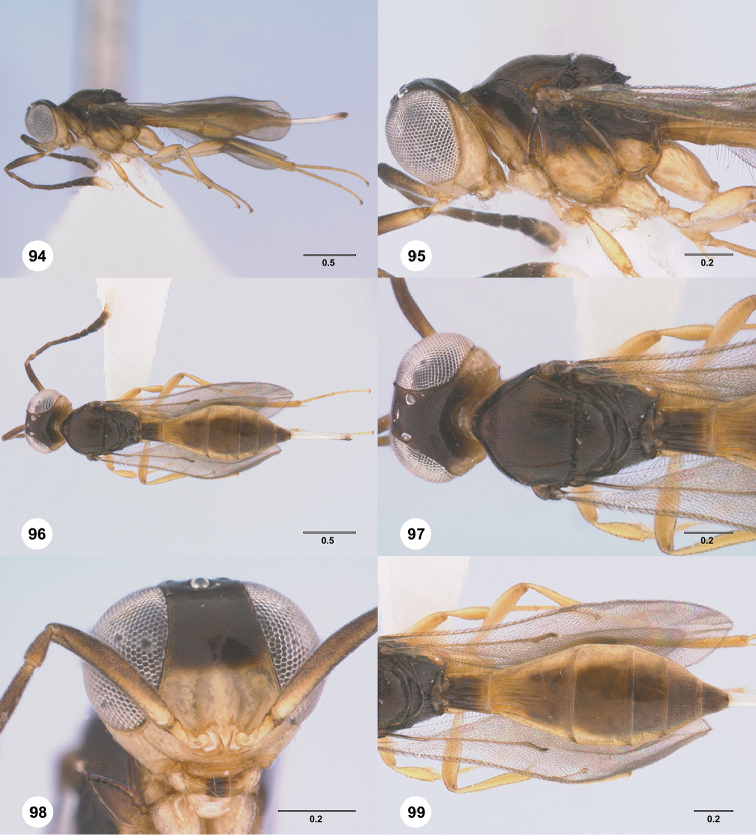
*Calliscelio eboris* sp. n., female, holotype (OSUC 193404). **94** Lateral habitus **95** Head and mesosoma, lateral view **96** Dorsal habitus **97** Head and mesosoma, dorsal view **98** Head, anterior view **99** Metasoma, dorsal view. Scale bars in millimeters.

#### Diagnosis.

This species is most similar to *Calliscelio brachys* and *Calliscelio suni*. It can be separated from *Calliscelio brachys* by the percurrent notaulus, and from *Calliscelio suni* by the spectral Rs+M and the presence of a central keel on the frons.

#### Etymology.

The epithet is used as a noun in apposition derived from the Latin word for ivory, in reference to the white A11–A12 of the female antennae.

#### Link to distribution map.

[http://hol.osu.edu/map-full.html?id=384800]

#### Material examined.

Holotype, female: **COLOMBIA**: Amazonas Dept., Amacayacu Natural N.P., M.84, 150m, 03°41'S 70°15'W, Matamata, 6.IV–15.IV.2000, Malaise trap, A. Parente, OSUC 193404 (deposited in IAVH). Paratypes: (58 females, 26 males) **BOLIVIA**: 12 females, 5 males, OSUC 534030, 534150–534151, 534156–534157, 534166–534171, 534173–534176, 534178–534179 (CNCI). **COLOMBIA**: 30 females, 19 males, OSUC 557578–557580 (CNCI); OSUC 143977, 152163, 152166, 162512, 162608, 176895, 189272, 193278, 193324–193325, 193327, 193424, 193461, 193932, 269448, 274572, 276241 (IAVH); OSUC 152164, 182227, 182485, 182492–182493, 182496, 182591, 182718–182720, 182733, 188948, 188959, 188962, 189175, 189205, 189216, 189275, 189283, 189290, 190305–190306, 192356, 193583, 193600, 231804, 231821, 272090, 279658 (OSUC). **PERU**: 11 females, 2 males, OSUC 553953, 553956, 553965, 554055–554064 (CNCI). **VENEZUELA**: 5 females, OSUC 223877–223881 (USNM).

### Calliscelio elegans

Taxon classificationAnimaliaHymenopteraPlatygastridae

(Perkins)

http://zoobank.org/6B386A5E-E5DF-48CB-A4C9-C82160E262C5

http://bioguid.osu.edu/xbiod_concepts/245756

Caloteleia elegans Perkins, 1910: 624 (original description).
Caenoteleia elegans (Perkins): Kieffer 1926: 550 (generic transfer, description).
Calotelea tanugatra Narendran, 1998: 71 (original description, keyed); Rajmohana K. 2006: 122, 123 (description, keyed); Rajmohana, Peter and Narendran 2013: 8 (junior synonym of Calliscelio elegans (Perkins), type information).
Calliscelio elegans (Perkins): Masner, Johnson and Musetti 2009: 61 (description, diagnosis, generic transfer); Rajmohana, Peter and Narendran 2013: 8 (description of male, synonymy).

#### Description.

See Masner et al. (2009) and Rajmohana et al. (2013).

#### Diagnosis.

*Callliscelio elegans* is easily distinguished within *Calliscelio* based on the combination of the following characters: fore wing with three darkened and two white bands; head and mesosonotum granulose; metascutellum extremely narrow and weakly concave medially to accommodate T1 horn (Masner et al. 2009).

#### Link to distribution map.

[http://hol.osu.edu/map-full.html?id=245756]

#### Material examined.

Non-type material: (61 females) **AUSTRALIA**: 1 female, OSUC 256856 (CNCI). **BELIZE**: 4 females, OSUC 256871–256874 (CNCI). **BENIN**: 3 females, OSUC 256882–256884 (CNCI). **CHRISTMAS ISLAND**: 4 females, OSUC 256857–256860 (ANIC). **FIJI**: 2 females, FBA015304, OSUC 256864 (BPBM). **FRENCH POLYNESIA**: 8 females, OSUC 256861–256863 (CNCI); OSUC 256866–256867 (UCDC); UCRC ENT 111562, 135651-135652 (UCRC). **GUAM**: 1 female, OSUC 256854 (CNCI). **INDIA**: 3 females, OSUC 256899–256901 (CNCI). **INDONESIA**: 4 females, OSUC 256893–256896 (CNCI). **IVORY COAST**: 2 females, OSUC 256885–256886 (CNCI). **MADAGASCAR**: 2 females, CASENT 2029779, OSUC 215759 (CAS). **MAURITIUS**: 1 female, MHNG 0002 (MHNG). **MEXICO**: 5 females, OSUC 256868–256870 (CNCI); OSUC 256875 (NMNH); OSUC 583205 (OSUC). **NEPAL**: 4 females, OSUC 256890–256892, 256898 (CNCI). **NIGERIA**: 1 female, OSUC 256881 (CNCI). **PUERTO RICO**: 2 females, OSUC 256876–256877 (CNCI). **SAMOA**: 1 female, OSUC 256865 (BPBM). **SRI LANKA**: 2 females, OSUC 256902–256903 (CNCI). **THAILAND**: 3 females, OSUC 256897, 256907, 321997 (OSUC). **UNITED STATES**: 3 females, OSUC 256855 (CNCI); OSUC 256878 (NMNH); UCFC 0 079 680 (UCFC). **VENEZUELA**: 2 females, OSUC 256879–256880 (CNCI). **YEMEN**: 3 females, OSUC 256887–256889 (CNCI).

### Calliscelio extenuatus

Taxon classificationAnimaliaHymenopteraPlatygastridae

Chen & Johnson sp. n.

http://zoobank.org/CE5AD0AA-DCC9-4645-AE36-45C93748A3EF

http://bioguid.osu.edu/xbiod_concepts/384360

Figures 100–105

#### Description.

Body length of female: 1.34–1.74 mm (n=20). Body length of male: 1.81 mm (n=1). Color of head: brown throughout; orange throughout; orange to pale brown. Color of antennal clava (A7–A12): dark brown to black; A7, A8 brown, A9–A12 white to pale yellow. Shape of head: subglobose. Central keel of frons: absent. Setation of upper frons: glabrous. IOS/EH: IOS distinctly less than EH. Sculpture of ventrolateral frons: smooth to coriaceous; smooth to granulate. Sculpture of frons below median ocellus: smooth; smooth to granulate. Sculpture of posterior vertex: rugose; granulate to rugulose. Hyperoccipital carina: absent. Occipital carina medially: weakly indicated, irregularly sculptured. Length of OOL: less than 0.5× ocellar diameter. Sculpture of postgena behind outer orbit: largely smooth with small granulate area. Ocular setae: sparse, short. A4 in female: distinctly shorter than A3. A5 in female: shorter than A3, as long as wide. Shape of female A6: distinctly wider than long.

Color of mesosoma in female: orange throughout; yellow throughout; orange to pale brown. Sculpture of dorsal pronotal area: rugose. Sculpture of lateral pronotal area: smooth throughout. Sculpture of netrion: smooth. Notaulus: percurrent or nearly so. Sculpture of mesoscutum: granulate. Shape of mesoscutellum: semiellipsoidal. Foveolae of scutoscutellar sulcus between notauli: smaller than those along margin of axilla. Sculpture of mesoscutellum: granulate. Shape of metascutellum: posterior margin rounded, approximately 3.0× wider than long. Sculpture of metascutellum in female: rugose. Dorsal propodeum in female: not excavate medially, lateral propodeal carinae meeting anteromedially. Sculpture of dorsal propodeum in female: rugose. Median keels on propodeum in female: absent. Mesopleural carina: absent. Sculpture of mesepisternum below mesopleural depression: smooth. Sculpture of ventral metapleural area: largely smooth, rugose ventrally. Color of legs: orange throughout; pale yellow throughout. Sculpture of hind coxa: smooth.

Color of fore wing: hyaline. Rs+M: nebulose, weakly pigmented. Setae on R: long, erect, surpassing the margin of the wing. Length of R: distinctly shorter than r-rs. Length of R1: greater than 3.0× length of r-rs.

Color of metasoma in female: orange throughout; variably orange to pale brown; yellow throughout. Horn on T1 in female: absent. Sculpture of posterior margin of T1 in female: longitudinally striate throughout. Development of longitudinal striae on T2 in female: reaching the middle of T2 medially; reaching posterior margin of T2. Sculpture of T3: smooth. Shape of T6 in female: short, wider than long. Sculpture of S3: largely smooth with sparse and fine punctures.

**Figures 100–105.**
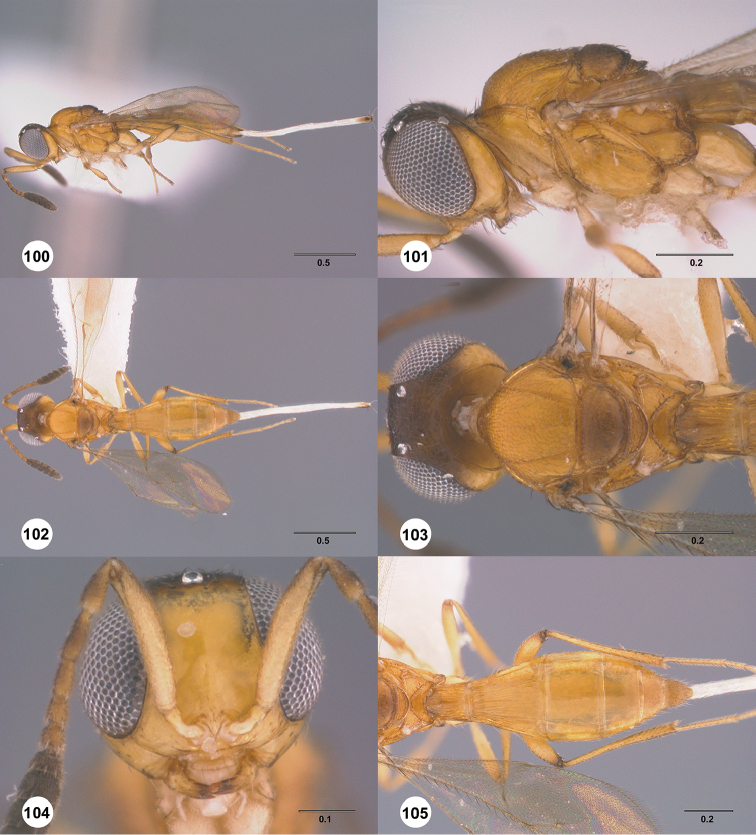
*Calliscelio extenuatus* sp. n., female, holotype (OSUC 48454). **100** Lateral habitus **101** Head and mesosoma, lateral view **102** Dorsal habitus **103** Head and mesosoma, dorsal view **104** Head, anterior view **105** Metasoma, dorsal view. Scale bars in millimeters.

#### Diagnosis.

This species is most similar to *Calliscelio ruga* and *Calliscelio suni* in size and habitus. It can be separated from *Calliscelio ruga* by the complete occipital carina and the shortened A6, from *Calliscelio suni* by the hairy compound eyes.

#### Etymology.

The epithet is an adjective, the Latin word for faint, weak and reduced, in reference to the weakly indicated occipital carina.

#### Link to distribution map.

[http://hol.osu.edu/map-full.html?id=384360]

#### Material examined.

Holotype, female: **VENEZUELA**: Aragua St., Rancho Grande Biological Station, 1100m, 10°22'N, 67°41'W, Henri Pittier National Park, 10.VIII–13.VIII.1994, flight intercept trap, M. Archangelsky, OSUC 48454 (deposited in OSUC). Paratypes: (170 females, 1 male) **COLOMBIA**: 1 female, OSUC 279960 (OSUC). **COSTA RICA**: 1 female, OSUC 237330 (OSUC). **TRINIDAD AND TOBAGO**: 5 females, OSUC 546023, 546097–546098, 546101–546102 (CNCI). **VENEZUELA**: 163 females, 1 male, OSUC 545835–545846, 545853, 545858–545861, 545873–545878, 545902, 545905–545934, 545938, 545944–545947, 545951–545954, 545960–545967, 545979–545990, 557647, 557650, 557661–557663, 557709–557710 (CNCI); OSUC 146712, 146714, 146716, 146747, 146764, 146768, 146777–146778, 146786, 334303–334308, 334310–334311, 334422–334423, 334511, 334514–334515, 334520–334522, 334524, 334536, 334539, 334542–334544, 334546, 334549–334551, 45296, 45300, 46280, 46286, 46299, 46532, 46537, 46563, 46580, 48128, 48139, 48146, 48166, 48176, 48315, 48326, 48371, 48387, 48396, 48408, 48426, 48459, 48480, 48492, 48605, 48623, 48628, 48641, 48723, 48839, 48881, 48885, 63892–63893, 63905, 63908, 63911, 63917, 64582 (OSUC).

### Calliscelio flavicauda

Taxon classificationAnimaliaHymenopteraPlatygastridae

Chen & Johnson sp. n.

http://zoobank.org/9A4B41F8-DDB0-449E-9E73-F5818AC5293A

http://bioguid.osu.edu/xbiod_concepts/363560

Figures 106–111

#### Description.

Body length of female: 1.95–1.99 mm (n=20). Body length of male: 1.90–2.05 mm (n=2). Color of head: brown throughout; orange throughout; orange to pale brown. Color of antennal clava (A7–A12): dark brown to black. Shape of head: subglobose. Central keel of frons: present. Setation of upper frons: with dense, short setae. IOS/EH: IOS distinctly less than EH. Sculpture of ventrolateral frons: granulate. Sculpture of frons below median ocellus: smooth; smooth to coriaceous. Sculpture of posterior vertex: granulate to rugulose. Hyperoccipital carina: absent. Occipital carina medially: complete, weakly crenulate throughout. Length of OOL: less than 0.5× ocellar diameter. Sculpture of postgena behind outer orbit: largely smooth with small granulate area. Ocular setae: absent. A4 in female: distinctly longer than A3. A5 in female: shorter than A3, distinctly longer than wide. Shape of female A6: distinctly longer than wide. Form of male antennal flagellomeres: filiform, A11 approximately 3.0× longer than wide. Length of A5 tyloid in male: approximately 0.3× length of A5.

Color of mesosoma in female: orange throughout; orange to pale brown; dark brown. Color of mesosoma in male: dark brown throughout. Sculpture of dorsal pronotal area: rugose. Sculpture of lateral pronotal area: smooth throughout. Sculpture of netrion: smooth. Notaulus: percurrent or nearly so. Sculpture of mesoscutum: coriaceous. Shape of mesoscutellum: semiellipsoidal. Foveolae of scutoscutellar sulcus between notauli: as large as those along margin of axilla. Sculpture of mesoscutellum: smooth with sparse fine punctures. Shape of metascutellum: posterior margin straight, approximately 4.0× wider than long. Sculpture of metascutellum in female: smooth with a longitudinal, median carina. Sculpture of metascutellum in male: smooth. Dorsal propodeum in female: shallowly excavate medially, with lateral propodeal carinae widely separated. Sculpture of dorsal propodeum in female: rugose. Sculpture of dorsal propodeum in male: rugose with one or two longitudinal keels lateral to median keel. Median keels on propodeum in female: absent. Mesopleural carina: present. Sculpture of mesepisternum below mesopleural depression: smooth. Sculpture of ventral metapleural area: smooth. Color of legs: hind femur brown, otherwise yellow. Sculpture of hind coxa: smooth.

Color of fore wing: hyaline. Rs+M: nebulose, strongly pigmented. Setae on R: long, erect, surpassing the margin of the wing. Length of R: distinctly longer than r-rs. Length of R1: greater than 3.0× length of r-rs.

Color of metasoma in female: anterior margin of T2 and T6 yellow, otherwise brown. Color of metasoma in male: anterior margin of T2 yellow, otherwise brown to black. Horn on T1 in female: present as a small bulge. Sculpture of T1 horn dorsally: transversely striate. Sculpture of posterior margin of T1 in female: smooth medially, longitudinally striate laterally. Sculpture of T1 in male: longitudinally striate. Development of longitudinal striae on T2 in female: reaching the middle of T2 medially. Sculpture of T3: smooth. Shape of T6 in female: distinctly elongate, approximately 2.0× longer than wide. Sculpture of S3: largely smooth with sparse and fine punctures.

**Figures 106–111.**
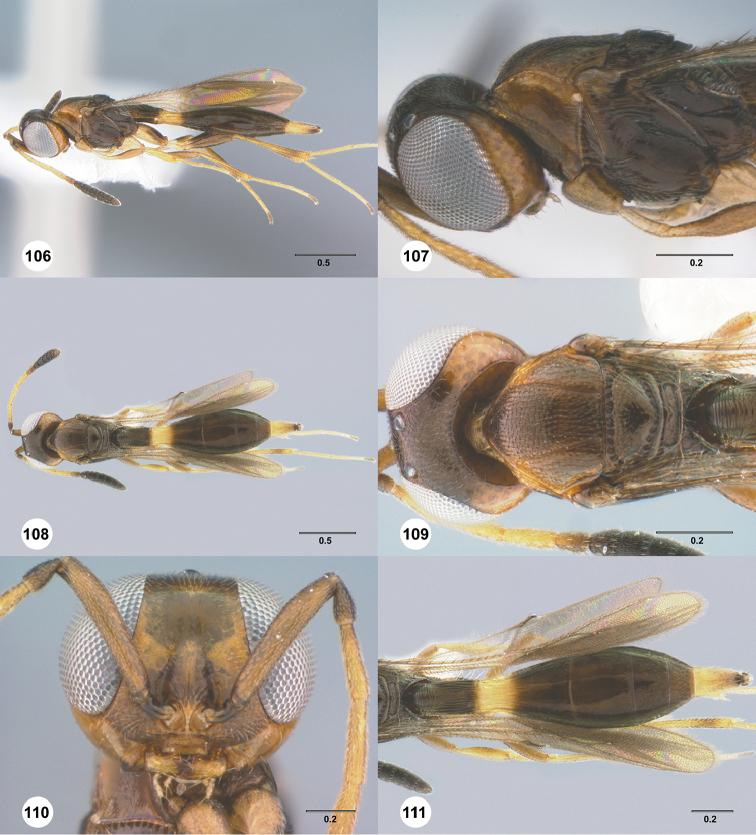
*Calliscelio flavicauda* sp. n., female, holotype (OSUC 553509). **106** Lateral habitus **107** Head and mesosoma, lateral view **108** Dorsal habitus **109** Head and mesosoma, dorsal view **110** Head, anterior view **111** Metasoma, dorsal view. Scale bars in millimeters.

#### Diagnosis.

Females of this species are most similar to *Calliscelio migma* in size and habitus but can be distinguished by the strongly foveolate scutoscutellar sulcus and the smooth T3. Males of *Calliscelio flavicauda* are similar to *Calliscelio laticintus* and *Calliscelio longius*. It may be separated from *Calliscelio laticinctus* by the smooth T3 and the brown hind femora, from *Calliscelio longius* that A11 is approximately 3.0× longer than wide.

#### Etymology.

The specific epithet meaning “yellow tail” refers to the yellow T6 in female and should be treated as a noun in apposition.

#### Link to distribution map.

[http://hol.osu.edu/map-full.html?id=363560]

#### Material examined.

Holotype, female: **ECUADOR**: Sucumbíos Prov., 270m, 00°30'S, 76°30'W, Sacha Lodge, 3.VII–13.VII.1994, Malaise trap, P. Hibbs, OSUC 553509 (deposited in CNCI). Paratypes: (52 females, 2 males) **BRAZIL**: 1 female, OSUC 534533 (CNCI). **COLOMBIA**: 11 females, OSUC 557602 (CNCI); OSUC 143969, 152156–152157, 178097, 178184, 182734, 189289, 193281, 193905, 262951 (OSUC). **ECUADOR**: 22 females, 2 males, OSUC 458499, 458509, 458511, 458535, 534224, 534232, 534234, 553377, 553442, 553506–553508, 553512, 553515, 553517, 553520–553523, 553562, 553566–553567, 553571, 553686 (CNCI). **FRENCH GUIANA**: 2 females, OSUC 546103 (CNCI); OSUC 570550 (OSUC). **PERU**: 16 females, OSUC 534417, 534421, 553992, 553994, 554007, 554036, 554038–554042, 554044–554046, 554050 (CNCI); OSUC 343060 (USNM).

### Calliscelio foveolatus

Taxon classificationAnimaliaHymenopteraPlatygastridae

Chen & Johnson sp. n.

http://zoobank.org/5734D0C9-1954-4A66-8E9E-412371890123

http://bioguid.osu.edu/xbiod_concepts/384809

Figures 112–117

#### Description.

Body length of female: 1.92–2.30 mm (n=10). Color of head: black throughout; reddish orange throughout. Color of antennal clava (A7–A12): black; A7 orange, remainder dark brown to black. Shape of head: subglobose. Central keel of frons: absent. Setation of upper frons: with sparse, short setae. IOS/EH: IOS distinctly less than EH. Sculpture of ventrolateral frons: smooth with sparse punctures. Sculpture of frons below median ocellus: largely smooth with sparse fine punctures. Sculpture of posterior vertex: coriaceous; largely smooth with irregular fine sculpture. Hyperoccipital carina: absent. Occipital carina medially: complete, weakly crenulate throughout. Length of OOL: less than 0.5× ocellar diameter; greater than 0.5× ocellar diameter. Sculpture of postgena behind outer orbit: smooth. Ocular setae: absent. A4 in female: distinctly shorter than A3. A5 in female: shorter than A3, distinctly longer than wide. Shape of female A6: slightly longer than wide.

Color of mesosoma in female: orange throughout; black throughout; variably yellow to pale brown. Sculpture of dorsal pronotal area: rugose. Sculpture of lateral pronotal area: smooth throughout. Sculpture of netrion: smooth. Notaulus: percurrent or nearly so. Sculpture of mesoscutum: coriaceous; coriaceous anteriorly, smooth with sparse fine punctures posteriorly. Shape of mesoscutellum: semiellipsoidal. Foveolae of scutoscutellar sulcus between notauli: as large as those along margin of axilla. Sculpture of mesoscutellum: smooth with sparse fine punctures. Shape of metascutellum: posterior margin straight, approximately 4.0× wider than long. Sculpture of metascutellum in female: smooth with a longitudinal, median carina. Dorsal propodeum in female: not excavate medially, lateral propodeal carinae meeting anteromedially. Sculpture of dorsal propodeum in female: rugose. Median keels on propodeum in female: absent. Mesopleural carina: present. Sculpture of mesepisternum below mesopleural depression: smooth. Sculpture of ventral metapleural area: smooth. Color of legs: pale yellow throughout. Sculpture of hind coxa: smooth.

Color of fore wing: hyaline. Rs+M: nebulose, strongly pigmented. Setae on R: long, erect, surpassing the margin of the wing. Length of R: approximately as long as r-rs. Length of R1: greater than 3.0× length of r-rs.

Color of metasoma in female: dark brown. Horn on T1 in female: weakly developed. Sculpture of T1 horn dorsally: smooth. Sculpture of posterior margin of T1 in female: longitudinally striate throughout. Development of longitudinal striae on T2 in female: reaching the middle of T2 medially. Sculpture of T3: smooth with longitudinal submedian striae. Shape of T6 in female: short, slightly longer than wide. Sculpture of S3: largely smooth with sparse and fine punctures.

**Figures 112–117.**
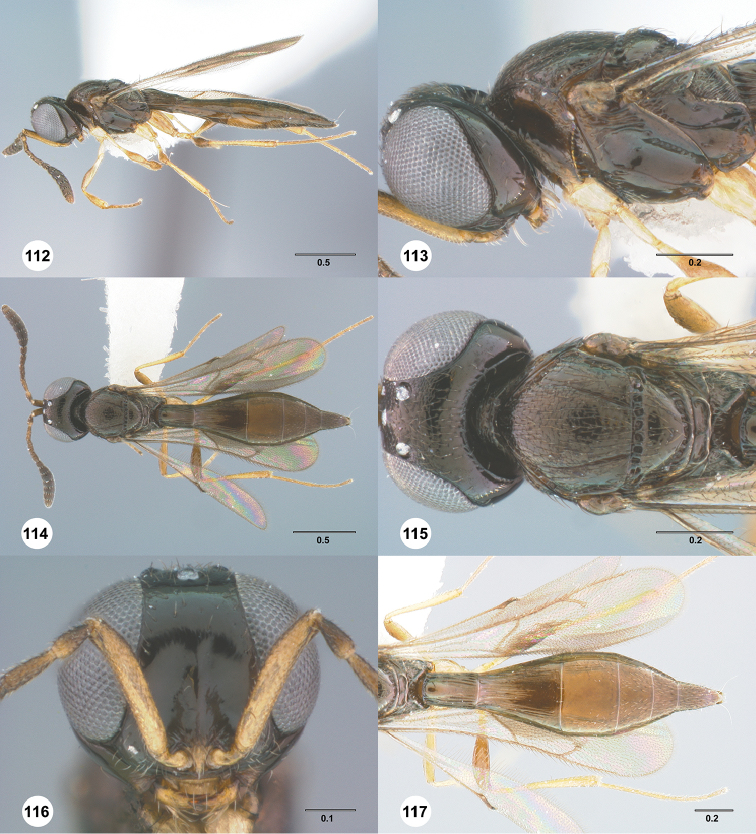
*Calliscelio foveolatus* sp. n., female, holotype (OSUC 532869). **112** Lateral habitus **113** Head and mesosoma, lateral view **114** Dorsal habitus **115** Head and mesosoma, dorsal view **116** Head, anterior view **117** Metasoma, dorsal view. Scale bars in millimeters.

#### Diagnosis.

This species is most similar to *Calliscelio levis* but can be distinguished by the foveolae of the scutoscutellar sulcus between the notauli which are as large as those along the margin of axilla and that A4 is distinctly shorter than A3.

#### Etymology.

The epithet is an adjective, derived from the Latin word for small hole, in reference to the sculpture of the scutoscutellar sulcus.

#### Link to distribution map.

[http://hol.osu.edu/map-full.html?id=384809]

#### Material examined.

Holotype, female: **COSTA RICA**: Puntarenas Prov., 24km W Piedras Blancas, Golfo Dulce, 200m, VI.1989–VIII.1989, Hanson & Goulet, OSUC 532869 (deposited in CNCI). Paratypes: (9 females) **BOLIVIA**: 1 female, OSUC 534177 (CNCI). **COSTA RICA**: 2 females, OSUC 532802, 532846 (CNCI). **ECUADOR**: 1 female, OSUC 553581 (CNCI). **PERU**: 4 females, OSUC 534393–534394, 534402, 553989 (CNCI). **VENEZUELA**: 1 female, OSUC 545903 (CNCI).

### Calliscelio gatineau

Taxon classificationAnimaliaHymenopteraPlatygastridae

Chen & Johnson sp. n.

http://zoobank.org/0C15B75D-0968-4247-BD87-9C08687543C8

http://bioguid.osu.edu/xbiod_concepts/362062

**Figures 118–123.**
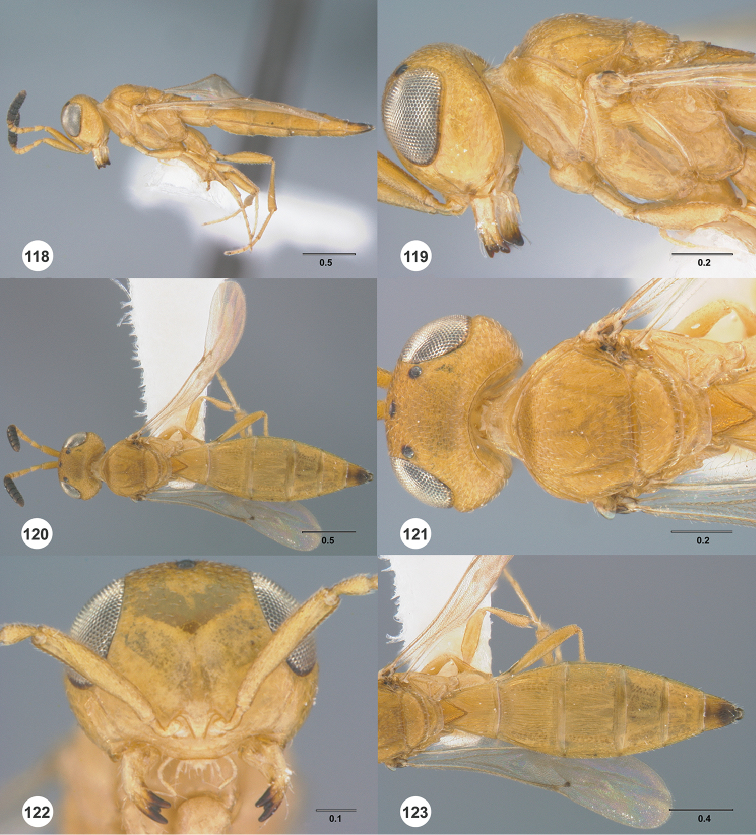
*Calliscelio gatineau* sp. n., female, holotype (OSUC 534340). **118** Lateral habitus **119** Head and mesosoma, lateral view **120** Dorsal habitus **121** Head and mesosoma, dorsal view **122** Head, anterior view **123** Metasoma, dorsal view. Scale bars in millimeters.

Figures 13 , 118–123

#### Description.

Body length of female: 2.97 mm (n=1). Color of head: orange throughout. Color of antennal clava (A7–A12): dark brown to black. Shape of head: subglobose. Central keel of frons: absent. Setation of upper frons: with sparse, short setae. IOS/EH: IOS slightly greater than EH. Sculpture of ventrolateral frons: smooth with sparse punctures. Sculpture of frons below median ocellus: granulate to finely punctate. Sculpture of posterior vertex: granulate. Hyperoccipital carina: absent. Occipital carina medially: complete, weakly crenulate throughout. Length of OOL: less than 0.5× ocellar diameter. Sculpture of postgena behind outer orbit: granulate. Ocular setae: absent. A4 in female: distinctly shorter than A3. A5 in female: shorter than A3, as long as wide. Shape of female A6: distinctly wider than long.

Color of mesosoma in female: orange throughout. Sculpture of dorsal pronotal area: rugose. Sculpture of lateral pronotal area: rugulose throughout. Sculpture of netrion: rugose. Notaulus: percurrent or nearly so. Sculpture of mesoscutum: granulate. Shape of mesoscutellum: semiellipsoidal. Foveolae of scutoscutellar sulcus between notauli: absent. Sculpture of mesoscutellum: granulate. Shape of metascutellum: posterior somewhat rounded, approximately 2.5× wider than long. Sculpture of metascutellum in female: rugose. Dorsal propodeum in female: shallowly excavate medially, with lateral propodeal carinae widely separated. Sculpture of dorsal propodeum in female: rugose. Median keels on propodeum in female: absent. Mesopleural carina: absent. Sculpture of mesepisternum below mesopleural depression: smooth. Sculpture of ventral metapleural area: smooth. Color of legs: orange throughout. Sculpture of hind coxa: smooth.

Color of fore wing: hyaline. Rs+M: spectral. Setae on R: short, decumbent, hardly exceeding the margin of the wing. Length of R: distinctly shorter than r-rs. Length of R1: approximately as long as r-rs.

Color of metasoma in female: orange throughout. Horn on T1 in female: present as a small bulge. Sculpture of T1 horn dorsally: granulate medially, with V-shaped keels laterally. Sculpture of posterior margin of T1 in female: longitudinally striate throughout. Development of longitudinal striae on T2 in female: reaching posterior margin of T2. Sculpture of T3: longitudinally striate throughout. Shape of T6 in female: short, wider than long. Sculpture of S3: longitudinally striate.

#### Diagnosis.

This species is most similar to the orange females of *Calliscelio rubriclavus* but can be distinguished based on the smooth sculpture of the mesepisternum below the mesopleural depression and the medially granulate T1 horn , with V-shaped keels laterally.

#### Etymology.

The specific epithet refers to the locality on the label of the holotype and should be treated as a noun in apposition.

#### Link to distribution map.

[http://hol.osu.edu/map-full.html?id=362062]

#### Material examined.

Holotype, female: **CANADA**: QC, path/ridge/woodpile, Gatineau Park, IX–XI.1995, L. Masner, OSUC 534340 (deposited in CNCI).

### Calliscelio glaber

Taxon classificationAnimaliaHymenopteraPlatygastridae

Chen & Masner sp. n.

http://zoobank.org/E7A2A6C2-E787-4F5A-BC99-958CF2FF06DF

http://bioguid.osu.edu/xbiod_concepts/362055

Figures 124–129

#### Description.

Body length of female: 1.79–2.17 mm (n=20). Body length of male: 1.80–2.36 mm (n=20). Color of head: brown throughout; orange throughout; orange to pale brown. Color of antennal clava (A7–A12): dark brown to black. Shape of head: subglobose. Central keel of frons: absent. Setation of upper frons: glabrous. IOS/EH: IOS distinctly less than EH. Sculpture of ventrolateral frons: smooth. Sculpture of frons below median ocellus: smooth. Sculpture of posterior vertex: smooth. Hyperoccipital carina: absent. Occipital carina medially: interrupted. Length of OOL: less than 0.5× ocellar diameter. Sculpture of postgena behind outer orbit: smooth. Ocular setae: absent. A4 in female: distinctly shorter than A3. A5 in female: shorter than A3, distinctly longer than wide. Shape of female A6: as long as wide. Form of male antennal flagellomeres: filiform, A11 approximately 3.0× longer than wide. Length of A5 tyloid in male: approximately 0.3× length of A5.

Color of mesosoma in female: orange throughout; orange to pale brown. Color of mesosoma in male: orange throughout. Sculpture of dorsal pronotal area: rugose. Sculpture of lateral pronotal area: smooth anteriorly, punctate rugulose posteriorly. Sculpture of netrion: smooth. Notaulus: percurrent or nearly so. Sculpture of mesoscutum: smooth throughout. Shape of mesoscutellum: semiellipsoidal. Foveolae of scutoscutellar sulcus between notauli: absent. Sculpture of mesoscutellum: smooth throughout. Shape of metascutellum: posterior margin rounded, approximately 2.5× wider than long. Sculpture of metascutellum in female: smooth. Sculpture of metascutellum in male: smooth. Dorsal propodeum in female: shallowly excavate medially, with lateral propodeal carinae widely separated. Sculpture of dorsal propodeum in female: rugose. Sculpture of dorsal propodeum in male: rugose. Median keels on propodeum in female: absent. Mesopleural carina: absent. Sculpture of mesepisternum below mesopleural depression: smooth. Sculpture of ventral metapleural area: smooth. Color of legs: orange yellow. Sculpture of hind coxa: smooth.

Color of fore wing: hyaline. Rs+M: spectral. Setae on R: long, erect, surpassing the margin of the wing. Length of R: approximately as long as r-rs. Length of R1: approximately as long as 2.0× length of r-rs.

Color of metasoma in female: orange throughout. Color of metasoma in male: orange throughout. Horn on T1 in female: present as a small bulge. Sculpture of T1 horn dorsally: concentrically striate. Sculpture of posterior margin of T1 in female: longitudinally striate throughout. Sculpture of T1 in male: longitudinally striate. Development of longitudinal striae on T2 in female: reaching posterior margin of T2. Sculpture of T3: largely smooth with submedian longitudinal striae. Shape of T6 in female: short, slightly longer than wide. Sculpture of S3: largely smooth with sparse and fine punctures.

**Figures 124–129.**
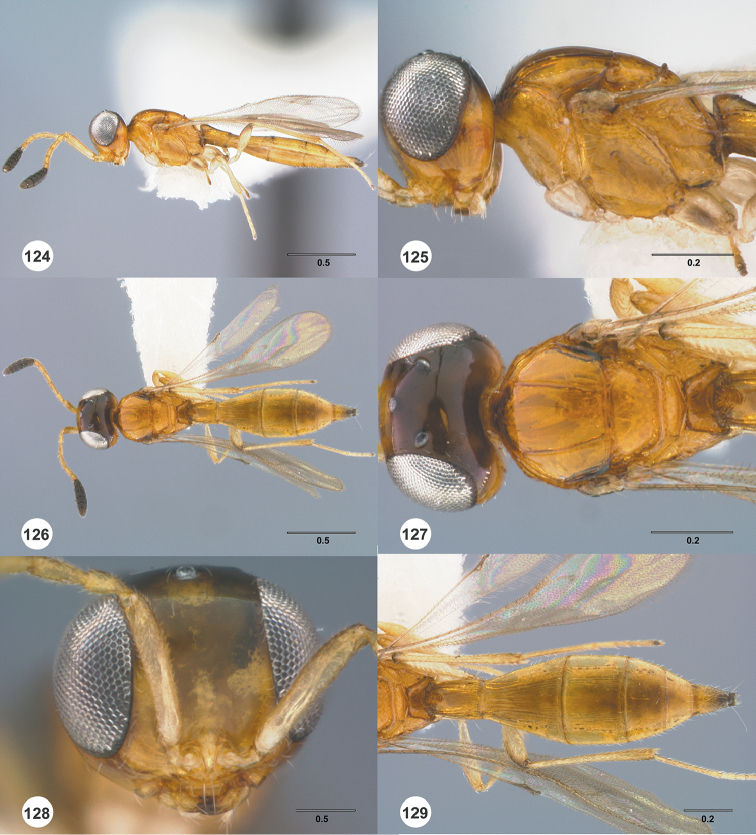
*Calliscelio glaber* sp. n., female, holotype (OSUC 458136). **124** Lateral habitus **125** Head and mesosoma, lateral view **126** Dorsal habitus **127** Head and mesosoma, dorsal view **128** Head, anterior view **129** Metasoma, dorsal view. Scale bars in millimeters.

#### Diagnosis.

This species is most similar to *Calliscelio paraglaber* in smooth body sculpture and size but can be distinguished by the interrupted occipital carina (occipital carina complete in *Calliscelio paraglaber*).

#### Etymology.

The epithet is an adjective and Latin word for smooth, in reference to the smooth body surface.

#### Link to distribution map.

[http://hol.osu.edu/map-full.html?id=362055]

#### Material examined.

Holotype, female: **DOMINICAN REPUBLIC**: Pedernales Prov., km25 of Alcoa Road, dry montane forest, Baoruco (Bahoruco) Mountains, 700m, 18.I.1989, L. Masner, OSUC 458136 (deposited in CNCI). Paratypes: (27 females, 20 males) **CUBA**: 2 females, 1 male, OSUC 458145–458146, 458241 (CNCI). **DOMINICAN REPUBLIC**: 25 females, 19 males, OSUC 458137–458144, 458147–458176, 534372–534373, 534376, 534594–534596 (CNCI).

### Calliscelio granulatus

Taxon classificationAnimaliaHymenopteraPlatygastridae

Chen & Masner sp. n.

http://zoobank.org/DC45F6BB-FDE6-4E77-8A00-9B2FD32359E4

http://bioguid.osu.edu/xbiod_concepts/362058

Figures 130–135

#### Description.

Body length of female: 2.31–2.53 mm (n=8). Body length of male: 2.24–2.26 mm (n=4). Color of head: orange throughout. Color of antennal clava (A7–A12): dark brown to black. Shape of head: subglobose. Central keel of frons: absent. Setation of upper frons: with dense, short setae. IOS/EH: IOS distinctly less than EH. Sculpture of ventrolateral frons: granulate. Sculpture of frons below median ocellus: granulate. Sculpture of posterior vertex: granulate. Hyperoccipital carina: absent. Occipital carina medially: complete, weakly crenulate throughout. Length of OOL: less than 0.5× ocellar diameter. Sculpture of postgena behind outer orbit: granulate. Ocular setae: dense, short. A4 in female: distinctly shorter than A3. A5 in female: shorter than A3, as long as wide. Shape of female A6: distinctly wider than long. Form of male antennal flagellomeres: filiform, A11 approximately 3.0× longer than wide. Length of A5 tyloid in male: greater than 0.5× length of A5.

Color of mesosoma in female: orange throughout. Color of mesosoma in male: orange throughout. Sculpture of dorsal pronotal area: rugose. Sculpture of lateral pronotal area: largely smooth, granulate ventrally and posteriorly. Sculpture of netrion: rugulose. Notaulus: percurrent or nearly so. Sculpture of mesoscutum: granulate. Shape of mesoscutellum: semiellipsoidal. Foveolae of scutoscutellar sulcus between notauli: smaller than those along margin of axilla. Sculpture of mesoscutellum: granulate. Shape of metascutellum: posterior margin straight, approximately 4.0× wider than long. Sculpture of metascutellum in female: rugose. Sculpture of metascutellum in male: rugose. Dorsal propodeum in female: deeply excavate medially, with lateral propodeal carinae widely separated, running subparallel to accommodate T1 horn. Sculpture of dorsal propodeum in female: rugose. Sculpture of dorsal propodeum in male: rugose. Median keels on propodeum in female: absent. Mesopleural carina: present. Sculpture of mesepisternum below mesopleural depression: smooth. Sculpture of ventral metapleural area: smooth. Color of legs: orange yellow. Sculpture of hind coxa: smooth.

Color of fore wing: hyaline. Rs+M: nebulose, weakly pigmented. Setae on R: long, erect, surpassing the margin of the wing. Length of R: distinctly shorter than r-rs. Length of R1: approximately as long as 2.0× length of r-rs.

Color of metasoma in female: orange with pale brown patch on T1, T2 and T6. Color of metasoma in male: orange with pale brown patch on T2 and T5–T7. Horn on T1 in female: large and distinct. Sculpture of T1 horn dorsally: longitudinally striate. Sculpture of posterior margin of T1 in female: longitudinally striate throughout. Sculpture of T1 in male: longitudinally striate. Development of longitudinal striae on T2 in female: reaching the middle of T2 medially; reaching posterior margin of T2. Sculpture of T3: smooth. Shape of T6 in female: distinctly elongate, approximately 2.0× longer than wide. Sculpture of S3: smooth.

**Figures 130–135.**
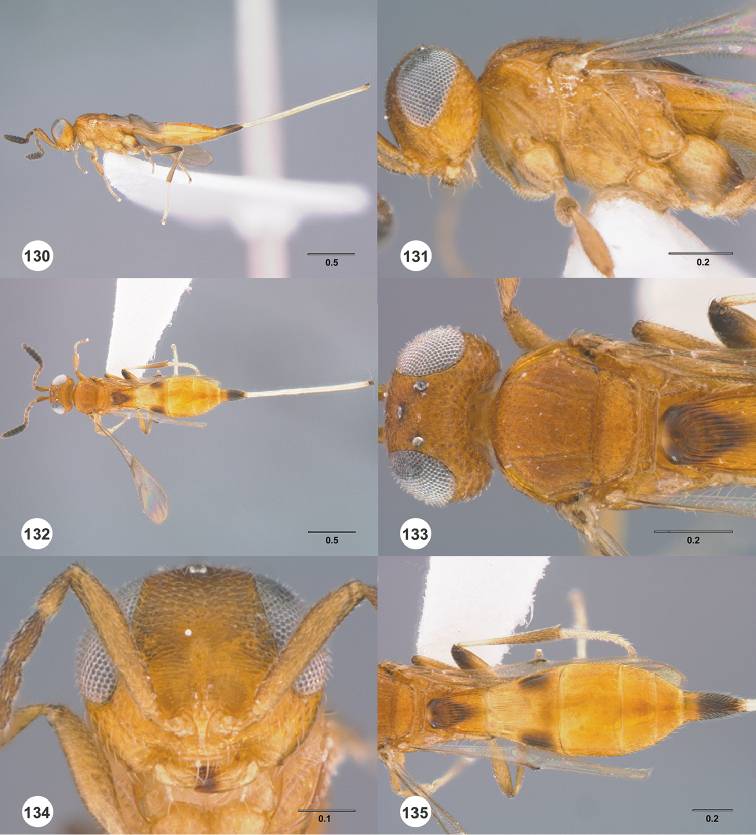
*Calliscelio granulatus* sp. n., female, holotype (OSUC 534599). **130** Lateral habitus **131** Head and mesosoma, lateral view **132** Dorsal habitus **133** Head and mesosoma, dorsal view **134** Head, anterior view **135** Metasoma, dorsal view. Scale bars in millimeters.

#### Diagnosis.

This species is most similar to *Calliscelio telum* in hairy compound eyes and habitus but can be distinguished by its rugulose netrion and longitudinally striate T1 horn.

#### Etymology.

The adjectival specific epithet refers to the sculpture of the body.

#### Link to distribution map.

[http://hol.osu.edu/map-full.html?id=362058]

#### Material examined.

Holotype, female: **CUBA**: Santiago de Cuba Prov., botanical garden/disturbed and scrub forest, Santiago de Cuba, 5–50m, 5.XII–17.XII.1995, flight intercept trap, S. Peck, OSUC 534599 (deposited in CNCI). Paratypes: **CUBA**: 7 females, 4 males, OSUC 458304–458314 (CNCI).

### Calliscelio laticinctus

Taxon classificationAnimaliaHymenopteraPlatygastridae

Ashmead

http://zoobank.org/A4202C74-7737-441D-86A1-876224167CFA

http://bioguid.osu.edu/xbiod_concepts/4152

**Figures 136–141.**
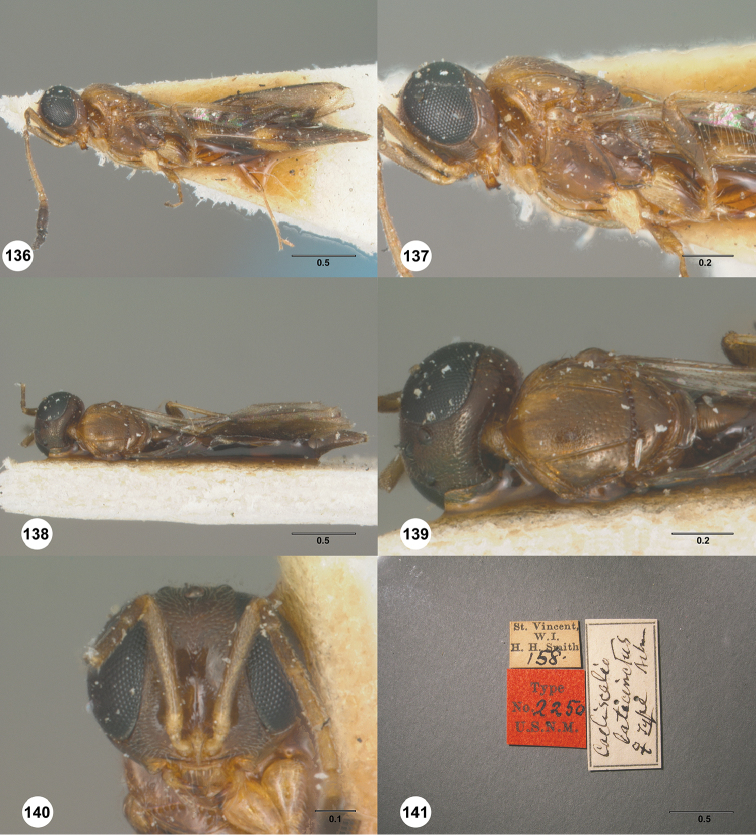
*Calliscelio laticinctus* Ashmead, female, syntype (USNMENT01059105). **136** Lateral habitus **137** Head and mesosoma, lateral view **138** Dorsal habitus **139** Head and mesosoma, dorsal view **140** Head, anterior view **141** Specimen labels. Scale bars in millimeters.

Figures 15 , 136–141

Calliscelio laticinctus Ashmead, 1893: 219 (original description); Ashmead 1894: 223 (redescribed as new); Ashmead 1900: 327 (distribution); Kieffer 1926: 499 (description, keyed); Masner 1976: 38 (description, type information).

#### Description.

Body length of female: 1.97–3.01 mm (n=20). Body length of male: 1.82–2.34 mm (n=20). Color of head: yellow throughout. Color of antennal clava (A7–A12): black. Shape of head: subglobose. Central keel of frons: present. Setation of upper frons: with dense, short setae. IOS/EH: IOS distinctly less than EH. Sculpture of ventrolateral frons: granulate. Sculpture of frons below median ocellus: coriaceous. Sculpture of posterior vertex: granulate. Hyperoccipital carina: absent. Occipital carina medially: complete, weakly crenulate throughout. Length of OOL: less than 0.5× ocellar diameter. Sculpture of postgena behind outer orbit: smooth. Ocular setae: absent. A4 in female: as long as A3. A5 in female: shorter than A3, distinctly longer than wide. Shape of female A6: distinctly longer than wide. Form of male antennal flagellomeres: filiform, A11 approximately 3.0× longer than wide. Length of A5 tyloid in male: approximately 0.3× length of A5.

Color of mesosoma in female: yellow throughout. Color of mesosoma in male: orange throughout; variably orange to pale brown; brown throughout. Sculpture of dorsal pronotal area: rugose. Sculpture of lateral pronotal area: smooth throughout. Sculpture of netrion: smooth. Notaulus: percurrent or nearly so. Sculpture of mesoscutum: granulate. Shape of mesoscutellum: semiellipsoidal. Foveolae of scutoscutellar sulcus between notauli: as large as those along margin of axilla. Sculpture of mesoscutellum: granulate. Shape of metascutellum: posterior margin straight, approximately 4.0× wider than long. Sculpture of metascutellum in female: rugose. Sculpture of metascutellum in male: rugose. Dorsal propodeum in female: deeply excavate medially, with lateral propodeal carinae widely separated, running subparallel to accommodate T1 horn. Sculpture of dorsal propodeum in female: rugose. Sculpture of dorsal propodeum in male: rugose. Median keels on propodeum in female: absent. Mesopleural carina: absent. Sculpture of mesepisternum below mesopleural depression: smooth. Sculpture of ventral metapleural area: smooth. Color of legs: orange throughout; pale yellow throughout. Sculpture of hind coxa: smooth.

Color of fore wing: hyaline. Rs+M: nebulose, weakly pigmented. Setae on R: long, erect, surpassing the margin of the wing. Length of R: distinctly longer than r-rs. Length of R1: greater than 3.0× length of r-rs.

Color of metasoma in female: dark brown; orange throughout; variably orange to black. Color of metasoma in male: brown throughout; variably yellow to pale brown; black throughout. Horn on T1 in female: large and distinct. Sculpture of T1 horn dorsally: concentrically striate. Sculpture of posterior margin of T1 in female: longitudinally striate throughout. Sculpture of T1 in male: longitudinally striate. Development of longitudinal striae on T2 in female: present on anterior margin of T2 medially, reaching posterior margin of T2 laterally. Sculpture of T3: largely smooth with submedian longitudinal striae. Shape of T6 in female: distinctly elongate, approximately 2.5× longer than wide. Sculpture of S3: largely smooth with sparse and fine punctures.

#### Diagnosis.

Females of this species are most similar to *Calliscelio longius* and *Calliscelio prolixus* in color, size and habitus. It can be separated from *Calliscelio longius* by A4 is slightly longer than A3 and A5 shorter than A3, from *Calliscelio prolixus* by the presence of T1 horn and the elongate T6. Males of *Calliscelio laticinctus* are similar to *Calliscelio flavicauda* and *Calliscelio longius*. It may be separated from *Calliscelio flavicauda* by T3 with submedian longitudinal striae and the brown hind femora, from *Calliscelio longius* by A11 is approximately 3.0× longer than wide.

#### Link to distribution map.

[http://hol.osu.edu/map-full.html?id=4152]

#### Material examined.

Syntype, female, *Calliscelio laticinctus*: **SAINT VINCENT AND THE GRENADINES**: Saint Vincent Island, no date, H. H. Smith, USNMENT01059105 (deposited in USNM). Syntype, female, *Calliscelio laticinctus*: **SAINT VINCENT AND THE GRENADINES**: Saint Vincent Island, no date, H. H. Smith, USNMENT01059354 (deposited in USNM). Other material: (323 females, 44 males) **BRAZIL**: 162 females, 32 males, OSUC 534536, 534538 (CNCI); OSUC 252092, 252094, 252097, 252105–252106, 252137, 252139, 252145, 254591, 322151, 322535–322537, 322539, 322548, 322557, 322720, 323005, 323007, 323080–323081, 323083–323084, 323086–323090, 323092–323093, 323959, 326149, 326194, 326220, 326222, 326235, 343696, 344403, 344481, 345098, 345119, 345205, 345207, 345333, 346169, 346870, 347431, 347438, 347452, 347482, 348267, 348488, 348496, 348669, 348677, 348992, 349017, 349024, 349191, 349194, 349196, 349198, 349513, 349780, 349782, 351241, 352056, 352061–352062, 352066–352067, 352071, 352249, 352359, 352799, 353038–353039, 353176, 354567, 354919, 355162, 355324–355328, 357042, 361705, 363838, 363840–363842, 363845, 363847, 366692, 366711, 366780, 366782, 366807, 366943, 368420, 370883–370886, 370888–370889, 370891, 370893–370896, 370899, 370906, 370929, 373762, 373770, 374565, 374567, 374575, 374586–374587, 374592, 374597, 374623–374630, 374737, 374742, 375308, 375310, 375315, 376518, 376524, 376976, 376978–376979, 376981–376982, 376985–376988, 377482–377491, 377494, 377504, 378011–378013, 378052, 378065, 378069, 378975–378976, 380179, 380183–380185, 380187–380189, 380193, 477165, 534688–534691, 55934, 55941, 55943, 577134, 577156, 577159, 577161, 583248–583249 (OSUC); OSUC 225355, 237645 (USNM). **COLOMBIA**: 39 females, 1 male, OSUC 534554, 557600–557601, 557641 (CNCI); OSUC 152155, 162597, 178015, 178205, 182484, 188778, 189200, 189211, 190987, 190990–190991, 191141, 191180, 191251, 192205, 192209, 193353, 193462–193463, 193547, 232296, 232303, 259575–259576, 259757, 262613, 267804, 269431, 272086, 274573, 274973, 276235, 279926, 279929, 363586, 364067 (OSUC). **DOMINICA**: 5 females, 1 male, OSUC 458242–458243, 458245–458248 (CNCI). **ECUADOR**: 4 females, OSUC 458237, 553441, 553477, 557140 (CNCI). **FRENCH GUIANA**: 31 females, 1 male, OSUC 458388, 458400–458401, 458412, 458434–458435, 458437, 458441–458443, 458449–458453, 458455, 458461, 458463, 458465–458468, 458470, 458475–458477, 458479, 546105, 546111, 546139, 546150 (CNCI); OSUC 570551 (OSUC). **GRENADA**: 2 females, OSUC 534253–534254 (CNCI). **GUYANA**: 11 females, OSUC 458236, 458252–458261 (CNCI). **PANAMA**: 2 females, OSUC 553757, 553771 (CNCI). **PERU**: 2 females, OSUC 534422, 534426 (CNCI). **SAINT VINCENT AND THE GRENADINES**: 1 female, OSUC 458244 (CNCI). **SURINAME**: 2 females, OSUC 534584, 534586 (CNCI). **VENEZUELA**: 62 females, 9 males, OSUC 534314–534339, 545956, 557659–557660, 557667, 557681, 557683, 557685–557695, 557698 (CNCI); OSUC 146713, 146715, 146741, 146760, 334309, 334312, 334344, 334347–334348, 334438, 334513, 334516, 334527, 334538, 334552, 360647, 360650, 360738, 367061–367062, 48118, 48901, 576990–576992, 64571, 79763 (OSUC).

### Calliscelio latifrons

Taxon classificationAnimaliaHymenopteraPlatygastridae

Chen & Johnson sp. n.

http://zoobank.org/F45AC467-663F-4811-B40F-AFC2493439BE

http://bioguid.osu.edu/xbiod_concepts/367272

Figures 4–6 , 142–147

#### Description.

Body length of female: 1.85–2.50 mm (n=11). Body length of male: 1.70–2.05 mm (n=10). Color of head: black throughout. Color of antennal clava (A7–A12): dark brown to black. Shape of head: subglobose. Central keel of frons: absent. Setation of upper frons: with sparse, short setae. IOS/EH: IOS slightly greater than EH. Sculpture of ventrolateral frons: granulate. Sculpture of frons below median ocellus: granulate. Sculpture of posterior vertex: granulate. Hyperoccipital carina: absent. Occipital carina medially: complete, weakly crenulate throughout. Length of OOL: greater than 0.5× ocellar diameter. Sculpture of postgena behind outer orbit: granulate. Ocular setae: sparse, short. A4 in female: distinctly shorter than A3. A5 in female: shorter than A3, as long as wide. Shape of female A6: as long as wide. Form of male antennal flagellomeres: moniliform, A11 as long as wide. Length of A5 tyloid in male: greater than 0.5× length of A5.

Color of mesosoma in female: black throughout; orange to pale brown. Color of mesosoma in male: dark brown throughout. Sculpture of dorsal pronotal area: granulate. Sculpture of lateral pronotal area: granulate throughout. Sculpture of netrion: rugulose. Notaulus: percurrent; abbreviated, at most reaching middle of mesoscutum. Sculpture of mesoscutum: granulate. Shape of mesoscutellum: transverse. Foveolae of scutoscutellar sulcus between notauli: absent. Sculpture of mesoscutellum: granulate. Shape of metascutellum: posterior margin straight, approximately 4.0× wider than long. Sculpture of metascutellum in female: granulate. Sculpture of metascutellum in male: granulate. Dorsal propodeum in female: deeply excavate medially, with lateral propodeal carinae widely separated, running subparallel to accommodate T1 horn. Sculpture of dorsal propodeum in female: rugose. Sculpture of dorsal propodeum in male: rugose. Median keels on propodeum in female: absent. Mesopleural carina: present. Sculpture of mesepisternum below mesopleural depression: granulate. Sculpture of ventral metapleural area: smooth dorsally, granulate ventrally. Color of legs: orange throughout. Sculpture of hind coxa: smooth.

Color of fore wing: hyaline. Rs+M: spectral. Setae on R: long, erect, surpassing the margin of the wing. Length of R: distinctly shorter than r-rs. Length of R1: approximately as long as 2.0× length of r-rs.

Color of metasoma in female: variably orange to pale brown. Color of metasoma in male: variably orange to pale brown; pale brown throughout. Horn on T1 in female: present as a small bulge. Sculpture of T1 horn dorsally: granulate. Sculpture of posterior margin of T1 in female: striate rugose. Sculpture of T1 in male: longitudinally rugose. Development of longitudinal striae on T2 in female: reaching posterior margin of T2. Sculpture of T3: smooth medially, longitudinally striate laterally. Shape of T6 in female: short, slightly longer than wide. Sculpture of S3: granulate.

**Figures 142–147.**
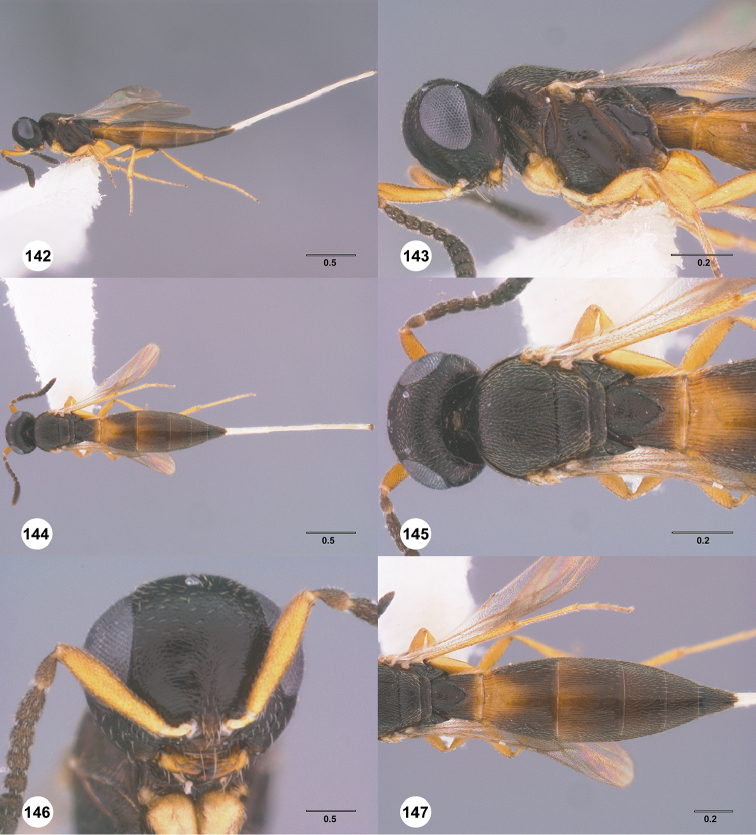
*Calliscelio latifrons* sp. n., female, holotype (OSUC 323077). **142** Lateral habitus **143** Head and mesosoma, lateral view **144** Dorsal habitus **145** Head and mesosoma, dorsal view **146** Head, anterior view **147** Metasoma, dorsal view. Scale bars in millimeters.

#### Diagnosis.

This species is most similar to *Calliscelio armila* but can be easily distinguished by the granulate upper frons and the longitudinally striate T3.

#### Etymology.

The epithet is a compound noun in apposition referring to the wide IOS.

#### Link to distribution map.

[http://hol.osu.edu/map-full.html?id=367272]

#### Material examined.

Holotype, female: **PARAGUAY**: Presidente Hayes Dept., 151m, 23°48'S 60°46'W, Escalante Lagoon, 27.XI.2003, yellow pan trap, B. Garcete, OSUC 323077 (deposited in MNHNPY). Paratypes: (10 females, 10 males) **ARGENTINA**: 2 females, OSUC 534438–534439 (CNCI). **BRAZIL**: 1 female, OSUC 534517 (CNCI). **PARAGUAY**: 7 females, 10 males, OSUC 322992, 363637, 534686–534687 (MNHNPY); OSUC 150574–150575, 276703, 322991, 322993, 323075–323076, 323078, 363707, 363711–363712, 434082–434083 (OSUC).

### Calliscelio levis

Taxon classificationAnimaliaHymenopteraPlatygastridae

Chen & Johnson sp. n.

http://zoobank.org/06FA4764-0408-4049-9F2C-A5997337F090

http://bioguid.osu.edu/xbiod_concepts/384811

Figures 148–153

#### Description.

Body length of female: 1.52–1.77 mm (n=13). Color of head: variably brown to black. Color of antennal clava (A7–A12): A7 orange, remainder dark brown to black; A7 dark brown, A8–A12 golden yellow. Shape of head: subglobose. Central keel of frons: absent. Setation of upper frons: with sparse, short setae. IOS/EH: IOS distinctly less than EH. Sculpture of ventrolateral frons: smooth with sparse punctures. Sculpture of frons below median ocellus: smooth. Sculpture of posterior vertex: coriaceous. Hyperoccipital carina: absent. Occipital carina medially: complete, weakly crenulate throughout. Length of OOL: greater than 0.5× ocellar diameter. Sculpture of postgena behind outer orbit: smooth. Ocular setae: absent. A4 in female: as long as A3. A5 in female: shorter than A3, distinctly longer than wide. Shape of female A6: distinctly longer than wide.

Color of mesosoma in female: orange throughout; variably yellow to pale brown. Sculpture of dorsal pronotal area: rugose. Sculpture of lateral pronotal area: smooth throughout. Sculpture of netrion: rugulose. Notaulus: percurrent or nearly so. Sculpture of mesoscutum: coriaceous. Shape of mesoscutellum: semiellipsoidal. Foveolae of scutoscutellar sulcus between notauli: smaller than those along margin of axilla. Sculpture of mesoscutellum: smooth with sparse fine punctures. Shape of metascutellum: posterior margin somewhat rounded, approximately 3.5× wider than long. Sculpture of metascutellum in female: smooth. Dorsal propodeum in female: not excavate medially, lateral propodeal carinae meeting anteromedially. Sculpture of dorsal propodeum in female: rugose. Median keels on propodeum in female: absent. Mesopleural carina: present. Sculpture of mesepisternum below mesopleural depression: smooth. Sculpture of ventral metapleural area: largely smooth, rugose ventrally. Color of legs: orange yellow. Sculpture of hind coxa: smooth.

Color of fore wing: hyaline. Rs+M: nebulose, strongly pigmented. Setae on R: long, erect, surpassing the margin of the wing. Length of R: approximately as long as r-rs. Length of R1: greater than 3.0× length of r-rs.

Color of metasoma in female: variably yellow to pale brown. Horn on T1 in female: weakly developed. Sculpture of T1 horn dorsally: smooth. Sculpture of posterior margin of T1 in female: longitudinally striate throughout. Development of longitudinal striae on T2 in female: present on anterior margin of T2 medially, reaching posterior margin of T2 laterally. Sculpture of T3: smooth; smooth with longitudinal submedian striae. Shape of T6 in female: short, subtriangular. Sculpture of S3: smooth.

**Figures 148–153.**
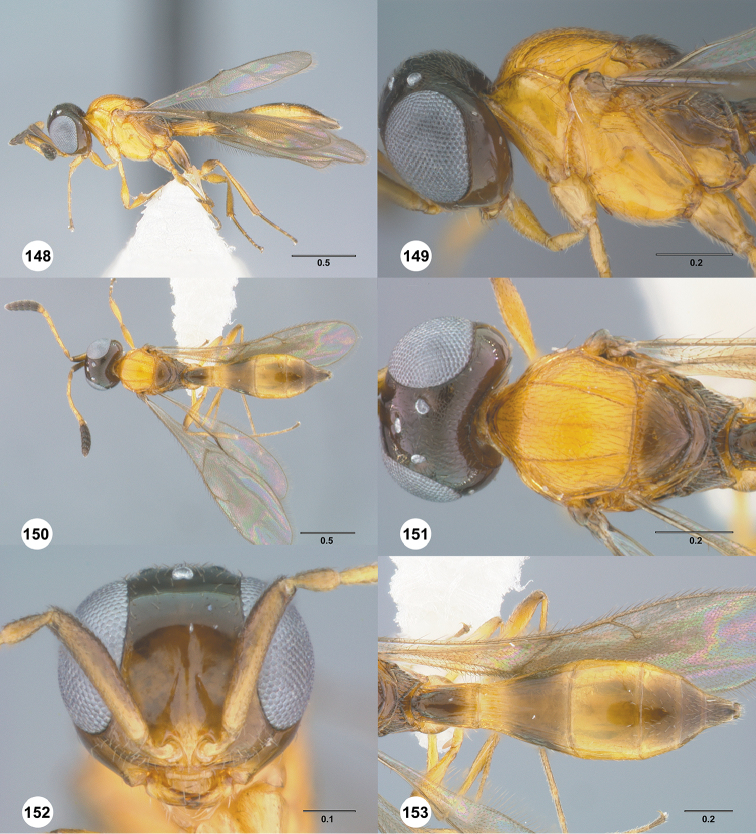
*Calliscelio levis* sp. n., female, holotype (OSUC 553454). **148** Lateral habitus **149** Head and mesosoma, lateral view **150** Dorsal habitus **151** Head and mesosoma, dorsal view **152** Head, anterior view **153** Metasoma, dorsal view. Scale bars in millimeters.

#### Diagnosis.

This species is most similar to *Calliscelio foveolatus* but can be distinguished because the foveolae of the scutoscutellar sulcus between the notauli are smaller than those along the margin of the axilla and A4 is as long as A3.

#### Etymology.

The epithet is an adjective, Latin word for smooth, polished and bald, in reference to the smooth T3.

#### Link to distribution map.

[http://hol.osu.edu/map-full.html?id=384811]

#### Material examined.

Holotype, female: **ECUADOR**: Napo Prov., Baeza, 2000m, 20.II–25.II.1979, Malaise trap, W. Mason, OSUC 553454 (deposited in CNCI). Paratypes: (12 females) **ECUADOR**: 10 females, OSUC 458530–458531, 553452, 553455, 553484, 553573, 553579–553580, 553582, 553691 (CNCI). **PERU**: 2 females, OSUC 534395, 534415 (CNCI).

### Calliscelio longius

Taxon classificationAnimaliaHymenopteraPlatygastridae

Chen & Johnson sp. n.

http://zoobank.org/36D661EE-96E2-4EB5-BB2F-0056C9FF514B

http://bioguid.osu.edu/xbiod_concepts/384815

Figures 10–12 , 154–159

#### Description.

Body length of female: 2.23–2.97 mm (n=20). Body length of male: 1.86–1.91 mm (n=3). Color of head: black throughout; orange throughout; variably brown to black; variably orange to dark brown. Color of antennal clava (A7–A12): black. Shape of head: subglobose. Central keel of frons: present. Setation of upper frons: with dense, short setae. IOS/EH: IOS distinctly less than EH. Sculpture of ventrolateral frons: granulate. Sculpture of frons below median ocellus: coriaceous; smooth to coriaceous. Sculpture of posterior vertex: granulate. Hyperoccipital carina: absent. Occipital carina medially: complete, weakly crenulate throughout. Length of OOL: less than 0.5× ocellar diameter. Sculpture of postgena behind outer orbit: smooth. Ocular setae: absent. A4 in female: distinctly longer than A3. A5 in female: longer than A3, distinctly longer than wide. Shape of female A6: length distinctly greater than width. Form of male antennal flagellomeres: filiform, A11 approximately 4.5× longer than wide. Length of A5 tyloid in male: approximately 0.3× length of A5.

Color of mesosoma in female: orange throughout; black throughout; variably orange to pale brown. Color of mesosoma in male: brown throughout; black throughout. Sculpture of dorsal pronotal area: rugose. Sculpture of lateral pronotal area: smooth throughout. Sculpture of netrion: smooth. Notaulus: percurrent or nearly so. Sculpture of mesoscutum: coriaceous; granulate. Shape of mesoscutellum: semiellipsoidal. Foveolae of scutoscutellar sulcus between notauli: as large as those along margin of axilla. Sculpture of mesoscutellum: smooth with sparse fine punctures. Shape of metascutellum: posterior margin straight, approximately 4.0× wider than long. Sculpture of metascutellum in female: smooth with a longitudinal, median carina. Sculpture of metascutellum in male: rugose. Dorsal propodeum in female: deeply excavate medially, with lateral propodeal carinae widely separated, running subparallel to accommodate T1 horn. Sculpture of dorsal propodeum in female: rugose. Sculpture of dorsal propodeum in male: rugose with one or two longitudinal keels lateral to median keel. Median keels on propodeum in female: absent. Mesopleural carina: present. Sculpture of mesepisternum below mesopleural depression: smooth. Sculpture of ventral metapleural area: smooth. Color of legs: orange yellow; hind femur brown, otherwise yellow. Sculpture of hind coxa: smooth.

Color of fore wing: hyaline. Rs+M: nebulose, strongly pigmented. Setae on R: long, erect, surpassing the margin of the wing. Length of R: distinctly longer than r-rs. Length of R1: greater than 3.0× length of r-rs.

Color of metasoma in female: dark brown; orange throughout; variably orange to black. Color of metasoma in male: brown throughout; variably yellow to pale brown; black throughout. Horn on T1 in female: large and distinct. Sculpture of T1 horn dorsally: transversely striate; smooth to somewhat transversely striate. Sculpture of posterior margin of T1 in female: longitudinally striate throughout. Sculpture of T1 in male: longitudinally striate. Development of longitudinal striae on T2 in female: reaching posterior margin of T2; present on anterior margin of T2 medially, reaching posterior margin of T2 laterally. Sculpture of T3: smooth; smooth with longitudinal submedian striae. Shape of T6 in female: distinctly elongate, approximately 3.5× longer than wide. Sculpture of S3: largely smooth with sparse and fine punctures.

**Figures 154–159.**
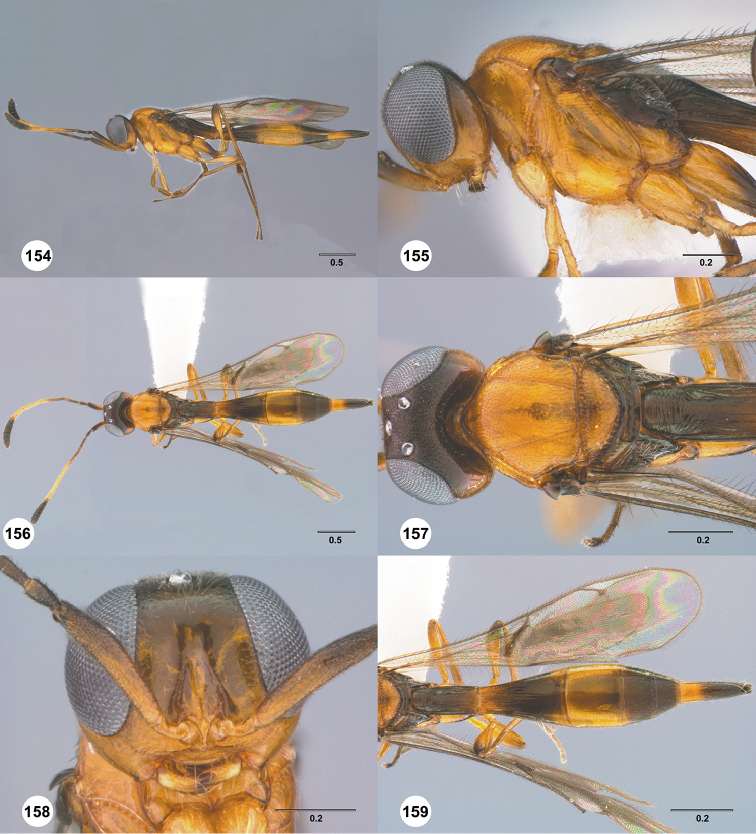
*Calliscelio longius* sp. n., female, holotype (OSUC 374730). **154** Lateral habitus **155** Head and mesosoma, lateral view **156** Dorsal habitus **157** Head and mesosoma, dorsal view **158** Head, anterior view **159** Metasoma, dorsal view. Scale bars in millimeters.

#### Diagnosis.

The females of this species are most similar to *Calliscelio laticinctus* and *Calliscelio prolixus* in color, size and habitus. It can be separated from *Calliscelio laticinctus* by A4 distinctly longer than A3 and A5 longer than A3, from *Calliscelio prolixus* by the presence of T1 horn and the elongate T6.

#### Etymology.

The epithet is an adjective, the Latin word for longer, in reference to A5 longer than A3.

#### Link to distribution map.

[http://hol.osu.edu/map-full.html?id=384815]

#### Material examined.

Holotype, female: **COLOMBIA**: Nariño Dept., Permanent Parcel, M.918, 1885m, 01°15'N 78°15'W, La Planada Nature Reserve, 16.VIII–2.IX.2000, Malaise trap, G. Oliva, OSUC 374730 (deposited in IAVH). Paratypes: (102 females, 3 males) **BRAZIL**: 3 females, OSUC 534528–534529, 534532 (CNCI). **COLOMBIA**: 62 females, 1 male, OSUC 557598 (CNCI); OSUC 152153, 152158–152159, 162497, 162588–162589, 162600, 178167, 178187, 178190, 182483, 182735, 182749–182751, 188924, 188926, 188947, 188952, 188956, 188958, 189208, 189212, 189293, 189300, 190935, 191037–191038, 191041, 191301, 191305, 191316, 191318, 193200, 193299, 193319, 193544, 193561–193562, 193567–193569, 193581–193582, 193596, 193685–193686, 193832, 193841, 193915, 193935, 262614–262615, 274571, 274574, 275802, 276238, 372641, 374729, 374731–374732, 377427 (OSUC). **ECUADOR**: 18 females, 1 male, OSUC 458238, 458487, 553371, 553436, 553444–553445, 553463, 553467, 553474, 553476, 553519, 553527, 553538, 553546, 553596, 553606, 553678, 553690, 557141 (CNCI). **PERU**: 17 females, 1 male, OSUC 534406, 534413, 534423–534425, 553964, 554034, 554037, 554048, 554051, 554054 (CNCI); OSUC 323931 (OSUC); OSUC 228066–228067, 228070, 228134, 231999, 343061 (USNM). **VENEZUELA**: 2 females, OSUC 557699–557700 (CNCI).

### Calliscelio magnificus

Taxon classificationAnimaliaHymenopteraPlatygastridae

Chen & Masner sp. n.

http://zoobank.org/809ACB6E-A75D-4C0F-8F0E-1643F54AE167

http://bioguid.osu.edu/xbiod_concepts/362060

Figures 160–165

#### Description.

Body length of female: 2.85–3.88 mm (n=19). Body length of male: 2.86–3.00 mm (n=7). Color of head: dark brown; orange to pale brown. Color of antennal clava (A7–A12): dark brown to black. Shape of head: subglobose. Central keel of frons: present. Setation of upper frons: with sparse, short setae. IOS/EH: IOS distinctly less than EH. Sculpture of ventrolateral frons: smooth to rugulose. Sculpture of frons below median ocellus: smooth; coriaceous. Sculpture of posterior vertex: granulate. Hyperoccipital carina: absent. Occipital carina medially: complete, weakly crenulate throughout. Length of OOL: less than 0.5× ocellar diameter. Sculpture of postgena behind outer orbit: largely smooth with small granulate area. Ocular setae: absent. A4 in female: distinctly longer than A3. A5 in female: shorter than A3, distinctly longer than wide. Shape of female A6: length distinctly greater than width. Form of male antennal flagellomeres: filiform, A11 approximately 3.5× longer than wide. Length of A5 tyloid in male: approximately 0.3× length of A5.

Color of mesosoma in female: variably orange to pale brown. Color of mesosoma in male: variably orange to pale brown. Sculpture of dorsal pronotal area: rugose. Sculpture of lateral pronotal area: smooth throughout. Sculpture of netrion: smooth. Notaulus: percurrent or nearly so. Sculpture of mesoscutum: granulate. Shape of mesoscutellum: semiellipsoidal. Foveolae of scutoscutellar sulcus between notauli: smaller than those along margin of axilla. Sculpture of mesoscutellum: smooth with sparse fine punctures. Shape of metascutellum: broad, short. Sculpture of metascutellum in female: smooth with a longitudinal, median carina. Sculpture of metascutellum in male: smooth. Dorsal propodeum in female: deeply excavate medially, with lateral propodeal carinae widely separated, running subparallel to accommodate T1 horn. Sculpture of dorsal propodeum in female: rugose. Sculpture of dorsal propodeum in male: rugose with one or two longitudinal keels lateral median keel. Median keels on propodeum in female: absent. Mesopleural carina: present. Sculpture of mesepisternum below mesopleural depression: smooth. Sculpture of ventral metapleural area: smooth. Color of legs: orange yellow; pale brown. Sculpture of hind coxa: smooth.

Color of fore wing: hyaline. Rs+M: nebulose, strongly pigmented. Setae on R: long, erect, surpassing the margin of the wing. Length of R: approximately as long as r-rs. Length of R1: greater than 3.0× length of r-rs.

Color of metasoma in female: variably orange to pale brown. Color of metasoma in male: variably orange to pale brown. Horn on T1 in female: large and distinct. Sculpture of T1 horn dorsally: smooth. Sculpture of posterior margin of T1 in female: longitudinally striate throughout. Sculpture of T1 in male: longitudinally striate. Development of longitudinal striae on T2 in female: present on anterior margin of T2 medially, reaching posterior margin of T2 laterally. Sculpture of T3: smooth with longitudinal submedian striae. Shape of T6 in female: distinctly elongate, approximately 3.0× longer than wide. Sculpture of S3: largely smooth with sparse and fine punctures.

**Figures 160–165.**
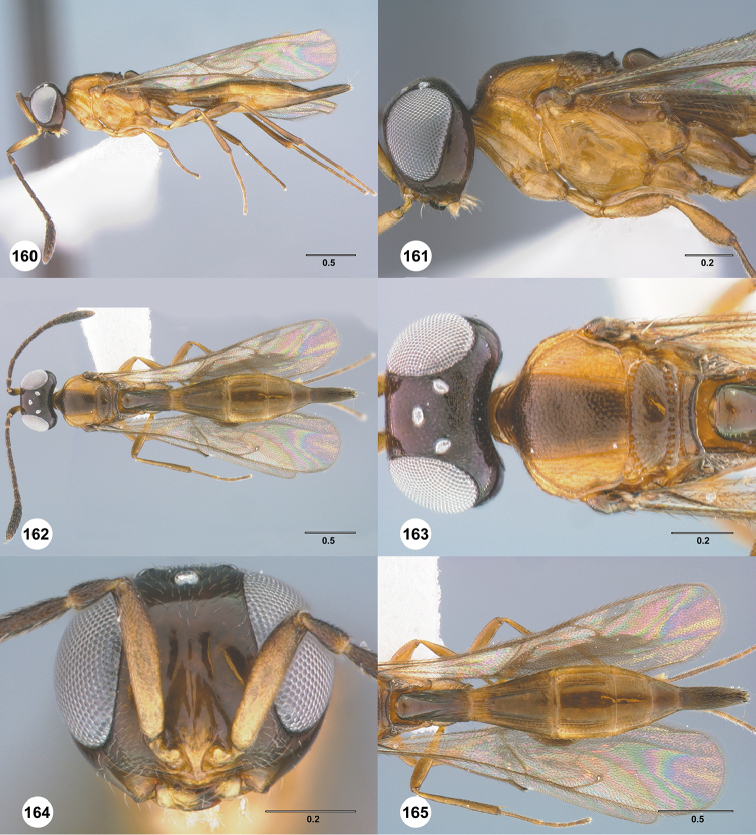
*Calliscelio magnificus* sp. n., female, holotype (OSUC 458272). **160** Lateral habitus **161** Head and mesosoma, lateral view **162** Dorsal habitus **163** Head and mesosoma, dorsal view **164** Head, anterior view **165** Metasoma, dorsal view. Scale bars in millimeters.

#### Diagnosis.

The females of this species are easily distinguished from other *Calliscelio* species by the combination of large and smooth T1 horn, elongate T6 and large body size. The males are most similar to *Calliscelio flavocauda*, *Calliscelio laticinctus* and *Calliscelio longius* but can be separated by the smooth metascutellum.

#### Etymology.

The specific epithet is to be treated as a noun in apposition, derived from the Latin for “splendid” and refers to our impression of this species.

#### Link to distribution map.

[http://hol.osu.edu/map-full.html?id=362060]

#### Material examined.

Holotype, female: **PERU**: Huánuco Reg., 39km NE Tingo Maria, Azul Range, 1700m, 11.I–14.I.1983, Newton & Thayer, OSUC 458272 (deposited in CNCI). Paratypes: (20 females, 8 males) **COSTA RICA**: 2 females, OSUC 532663, 532746 (CNCI). **ECUADOR**: 1 female, OSUC 553449 (CNCI). **PERU**: 13 females, 8 males, OSUC 458263–458264, 458266–458271, 458273–458284, 534405 (CNCI). **VENEZUELA**: 4 females, OSUC 458233–458235, 557668 (CNCI).

### Calliscelio migma

Taxon classificationAnimaliaHymenopteraPlatygastridae

Chen & Johnson sp. n.

http://zoobank.org/2F3A637F-06EC-4ECE-8E73-D337BA903320

http://bioguid.osu.edu/xbiod_concepts/384750

Figures 166–171

#### Description.

Body length of female: 1.34–2.16 mm (n=20). Body length of male: 1.43–1.94 mm (n=20). Color of head: yellow throughout; orange throughout. Color of antennal clava (A7–A12): dark brown to black; A7, A8 brown, A9–A12 white to pale yellow. Shape of head: subglobose. Central keel of frons: absent. Setation of upper frons: with dense, short setae; with sparse, short setae. IOS/EH: IOS distinctly less than EH. Sculpture of ventrolateral frons: smooth to granulate. Sculpture of frons below median ocellus: smooth; granulate. Sculpture of posterior vertex: smooth; granulate to rugulose. Hyperoccipital carina: absent. Occipital carina medially: weakly indicated, irregularly sculptured. Length of OOL: less than 0.5× ocellar diameter; greater than 0.5× ocellar diameter. Sculpture of postgena behind outer orbit: smooth. Ocular setae: absent. A4 in female: distinctly longer than A3. Shape of female A5: length distinctly greater than width. Shape of female A6: length distinctly greater than width. Form of male antennal flagellomeres: thread-like, length at least 4.0× greater than width. Length of A5 tyloid in male: approximately 0.3× length of A5.

Color of mesosoma in female: orange throughout; yellow throughout. Color of mesosoma in male: orange throughout; yellow throughout. Sculpture of dorsal pronotal area: rugose; smooth. Sculpture of lateral pronotal area: smooth throughout. Sculpture of netrion: smooth. Notaulus: percurrent or nearly so. Sculpture of mesoscutum: smooth with sparse punctures; coriaceous. Shape of mesoscutellum: semiellipsoidal. Scutoscutellar sulcus medially: weakly foveolate. Sculpture of mesoscutellum: smooth with sparse fine punctures; coriaceous. Shape of metascutellum: broad, short. Sculpture of metascutellum in female: smooth; rugose. Sculpture of metascutellum in male: rugose; smooth. Dorsal propodeum in female: shallowly excavate medially, with lateral propodeal carinae widely separated. Sculpture of lateral propodeal area in female: rugose. Sculpture of lateral propodeal area in male: rugose with one or two longitudinal keels lateral to median keel; longitudinally striate. Median keels on propodeum in female: absent. Mesopleural carina: absent. Sculpture of mesepisternum below mesopleural depression: smooth. Sculpture of ventral metapleural area: smooth; largely smooth, rugose ventrally. Color of legs: orange throughout; pale yellow throughout. Sculpture of hind coxa: smooth.

Color of fore wing: hyaline. Rs+M: nebulose, strongly pigmented. Setae on R: long, erect, surpassing the margin of the wing. Length of R: approximately as long as r-rs. Length of R1: greater than 3.0× length of r-rs.

Color of metasoma in female: orange throughout; yellow throughout. Color of metasoma in male: orange throughout; variably orange to pale brown. Horn on T1 in female: large and distinct; present as a small bulge. Sculpture of T1 horn dorsally: smooth; rugulose; transversely striate; smooth to somewhat transversely striate. Sculpture of posterior margin of T1 in female: longitudinally striate throughout. Sculpture of T1 in male: longitudinally striate. Development of longitudinal striae on T2 in female: present on anterior margin of T2 medially, reach posterior margin of T2 laterally. Sculpture of T3: smooth with longitudinal submedian striae; longitudinally striate throughout. Shape of T6 in female: short, subtriangular. Sculpture of S3: largely smooth with sparse and fine punctures.

**Figures 166–171.**
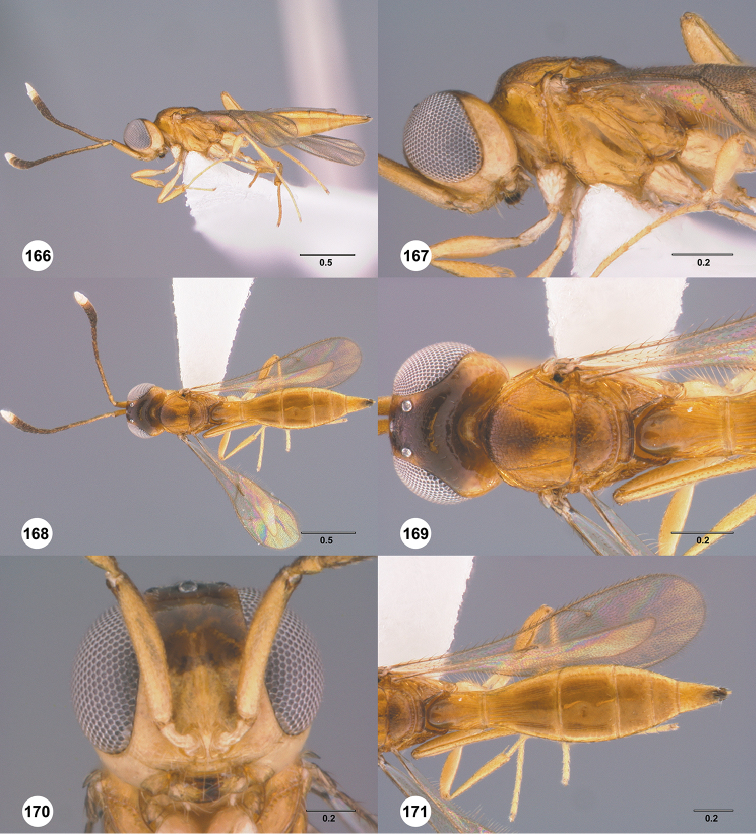
*Calliscelio migma* sp. n., female, holotype (OSUC 380181). **166** Lateral habitus **167** Head and mesosoma, lateral view **168** Dorsal habitus **169** Head and mesosoma, dorsal view **170** Head, anterior view **171** Metasoma, dorsal view. Scale bars in millimeters.

#### Diagnosis.

This species is most similar to *Calliscelio amadoi* in color and size but it can be easily distinguished by having A5 and A6 in female distinctly longer than wide, whereas in *Calliscelio amadoi* A5 is slightly longer than wide, A6 quadrate.

#### Etymology.

The epithet is used as a noun in apposition derived from the Greek word for mixture, in reference to the variations in female antennae color and T1 horn sculpture.

#### Link to distribution map.

[http://hol.osu.edu/map-full.html?id=384750]

#### Material examined.

Holotype, female: **BRAZIL**: PA, Melgaço Mpio., Igarapé Tijucaquara, trail, M11, P05128, 01°44'12.8"S 51°29'56.6"W, Caxiuanã, 18.XI–24.XI.2003, Malaise trap, A. P. Aguiar & J. Dias, OSUC 380181 (deposited in MPEG). Paratypes: (661 females, 82 males) **BOLIVIA**: 1 female, OSUC 534172 (CNCI). **BRAZIL**: 233 females, 41 males, OSUC 534513–534516, 534531, 534535, 534543–534545, 557166, 557201–557202, 557204, 557208–557209, 557395 (CNCI); OSUC 252141, 322371, 322377, 322671, 326226, 326230, 344407–344408, 344487, 345100, 345312, 345325, 345331, 345589, 346174, 346180, 346888, 346946, 346954, 346964, 347060, 347207–347208, 347213, 347229, 347427, 347437, 348008, 348115, 348641, 348678, 349368, 349636, 349659, 349685, 349771, 349783, 349785, 351289, 351329, 351462, 351467, 351525, 351528, 351673, 351677–351678, 351680–351683, 351815, 351823, 351833, 351836–351838, 352357, 352800, 352802, 352806, 352810, 352812, 353174–353175, 353177, 353717, 353725–353726, 353732, 353951–353953, 354075, 354082, 354710, 354831, 354885, 354892, 354895, 354898, 354900, 362588–362589, 363839, 363843–363844, 363849, 370897, 370905, 370908, 370915, 370923–370924, 371841–371842, 371844, 376057, 376541, 378963, 477166–477167, 577157, 577162, 577173, 583256 (MZSP); OSUC 251768, 252087, 322150, 322152, 322370, 322532, 322545–322547, 323091, 323958, 323964, 323970, 326189, 326397, 326404, 326502–326503, 326505, 326538, 337221–337222, 346041, 346997, 347244, 347277, 347293, 347295, 347655, 347660, 347671, 347678, 347852, 347871, 347954, 348074–348075, 348081, 348094, 348097, 348117, 348356, 349102–349103, 349107, 349111, 349201, 351425, 353047, 353426, 354797, 357021, 362607, 366698, 366701, 366712, 366721, 366778–366779, 366790, 366808, 366938–366939, 366944–366945, 366953, 366964–366966, 366974, 371836, 371839, 371849, 372549–372550, 373757, 373761, 373773, 373779, 374557, 374560–374561, 374717, 374739–374741, 376486, 376498–376500, 376505, 376509, 376527, 376530–376531, 376538, 376542, 376966, 377495–377497, 378014–378015, 378033, 378035, 378041, 378054–378055, 378063, 378080, 378085, 378595, 378597, 378960–378962, 378967, 378969, 378972–378973, 378979, 380182, 45697, 45721, 55930–55932, 55946, 55953, 61378, 61404, 61446, 61454, 61499, 61504, 61518, 61580, 63283, 63288, 63292, 63295, 63309, 63319, 63330, 63353, 63362, 63431, 63439, 63538, 63643–63644, 63665 (OSUC). **COLOMBIA**: 330 females, 19 males, OSUC 557547, 557549, 557555–557556, 557599, 557622, 557640, 557645–557646 (CNCI); OSUC 144165, 152145, 152147–152148, 162500, 162506, 162510, 162514, 162590, 162592, 162605, 162614, 162621–162623, 178018–178019, 178091, 178157–178159, 178161, 178165–178166, 178169–178173, 178175–178177, 178183, 178185, 178188–178189, 178194, 178196–178197, 178199–178200, 178202–178204, 178206, 179458–179459, 182585–182587, 182589–182590, 182594, 182721, 182736, 182738–182739, 188624, 188684, 188921, 188923, 188928–188930, 188950, 188953, 189276, 189278, 189280, 189284, 189288, 189301–189302, 189392, 191095, 193318, 193323, 193332, 193335–193337, 193339, 193341, 193343, 193345, 193354, 193356, 193359, 193394, 193396–193397, 193400, 193403, 193537, 193540, 193546, 193590, 231806–231809, 231813–231817, 231819, 231822, 231828, 232300, 259764, 272088, 275806–275807, 275809, 276187, 276195–276196, 276240, 278953, 279660, 363589, 363599, 364074, 372635 (IAVH); OSUC 143967–143968, 143972, 152133–152140, 152142–152144, 152146, 162498, 162502, 162585, 162595, 162598, 162604, 176896, 178164, 182225–182226, 182488–182490, 182498, 182500, 182593, 182744–182748, 182763–182765, 182767, 188549, 188681, 188687, 188922, 188925, 188927, 188931–188932, 188935–188936, 188938–188939, 189177–189178, 189180–189182, 189184, 189187–189189, 189192, 189194, 189196–189197, 189201–189202, 189204, 189206–189207, 189209, 189214, 189281, 189286, 189298–189299, 190307–190309, 190312, 191039, 191177–191179, 191181, 191186, 191207, 191319, 191826, 193277, 193279–193280, 193283, 193285–193287, 193290, 193333–193334, 193344, 193346, 193350–193352, 193357, 193398–193399, 193402, 193405–193406, 193412, 193423, 193468–193470, 193539, 193545, 193550, 193571, 193573–193574, 193577–193580, 193585–193586, 193588, 193592, 193594, 193777, 193780, 193901, 193904, 193907–193909, 193911–193912, 193914, 193934, 193936–193937, 193939, 194198, 231805, 231823, 231825–231826, 249898, 253452, 253456, 253461, 259751, 259755, 259763, 262602–262605, 262617–262618, 262942–262944, 262946–262947, 262949–262950, 262952, 262955–262960, 262965, 267810, 269349, 269351, 269354, 269433–269435, 269437–269439, 269483, 272082, 272084, 272091, 273456, 274971, 275028–275029, 275812–275815, 275857–275859, 276192, 276233–276234, 279656, 279659, 363588, 364078, 372632–372633, 372637–372638, 372643, 76996 (OSUC). **COSTA RICA**: 1 male, OSUC 245173 (OSUC). **ECUADOR**: 41 females, 3 males, OSUC 458485, 458491–458493, 532693–532694, 534223, 534230–534231, 534233, 534243, 534246–534247, 553247, 553385, 553390–553391, 553393–553395, 553397, 553405, 553415, 553457–553460, 553549, 553557, 553563–553564, 553585, 553592, 553627–553628, 553682, 553730, 577332 (CNCI); OSUC 534657, 534664–534667, 534671 (OSUC). **FRENCH GUIANA**: 26 females, 10 males, OSUC 458389, 458391, 458399, 458407–458408, 458417–458420, 458424–458425, 458430–458433, 458436, 458438–458440, 458447–458448, 458454, 458456–458458, 533981, 534553, 546110, 546129, 546131, 546133–546134, 546142–546143, 546146–546147 (CNCI). **PERU**: 30 females, 8 males, OSUC 534390, 534400, 534414, 534418, 534420, 534429, 553954, 553957–553961, 553963, 553966, 553978, 553990, 554000, 554002, 554005–554006, 554008–554009, 554030, 554033, 554053 (CNCI); OSUC 323932, 570535–570536, 570545, 570547–570548 (OSUC); OSUC 228068–228069, 228071, 228074, 228193–228194, 232000 (USNM).

#### Comments.

This species is well supported by many characters, although there are variations in female antennae color and T1 horn sculpture. The common color of the female antennal club is dark brown to black, while there are a few exceptions that A7, A8 brown, A9–A12 white to pale yellow. T1 horn usually is smooth, while a few species rugulose, or transversely striate, or smooth to somewhat transversely striate. These variations are gradual among specimens. Therefore we consider them as intraspecific rather than interspecific.

### Calliscelio minutia

Taxon classificationAnimaliaHymenopteraPlatygastridae

Chen & Johnson sp. n.

http://zoobank.org/89839644-E7FF-40CF-834B-5C380C230B9B

http://bioguid.osu.edu/xbiod_concepts/363279

Figures 172–177

#### Description.

Body length of female: 1.70–2.06 mm (n=20). Body length of male: 1.77–1.94 mm (n=8). Color of head: yellow throughout. Color of antennal clava (A7–A12): dark brown to black. Shape of head: subglobose. Central keel of frons: absent. Setation of upper frons: with sparse, long setae. IOS/EH: IOS distinctly less than EH. Sculpture of ventrolateral frons: granulate. Sculpture of frons below median ocellus: granulate. Sculpture of posterior vertex: granulate. Hyperoccipital carina: absent. Occipital carina medially: interrupted. Length of OOL: less than 0.5× ocellar diameter. Sculpture of postgena behind outer orbit: granulate. Ocular setae: absent. A4 in female: distinctly shorter than A3. A5 in female: shorter than A3, as long as wide. Shape of female A6: distinctly wider than long. Form of male antennal flagellomeres: filiform, A11 approximately 2.0× longer than wide. Length of A5 tyloid in male: greater than 0.5× length of A5.

Color of mesosoma in female: yellow throughout; yellow with mesoscutellum pale brown. Color of mesosoma in male: yellow throughout; variably yellow to pale brown. Sculpture of dorsal pronotal area: rugose. Sculpture of lateral pronotal area: smooth anteriorly, granulate posteriorly. Sculpture of netrion: smooth. Notaulus: percurrent or nearly so. Sculpture of mesoscutum: granulate. Shape of mesoscutellum: semiellipsoidal. Foveolae of scutoscutellar sulcus between notauli: absent. Sculpture of mesoscutellum: granulate. Shape of metascutellum: posterior margin rounded, approximately 3.0× wider than long. Sculpture of metascutellum in female: smooth. Sculpture of metascutellum in male: rugose. Dorsal propodeum in female: not excavate medially, lateral propodeal carinae meeting anteromedially. Sculpture of dorsal propodeum in female: smooth to rugulose. Sculpture of dorsal propodeum in male: rugulose. Median keels on propodeum in female: absent. Mesopleural carina: present. Sculpture of mesepisternum below mesopleural depression: smooth. Sculpture of ventral metapleural area: smooth dorsally, densely punctate ventrally. Color of legs: pale yellow throughout; hind coxa pale brown, otherwise pale yellow. Sculpture of hind coxa: smooth.

Color of fore wing: hyaline with infuscate band in the middle. Rs+M: spectral. Setae on R: long, erect, surpassing the margin of the wing. Length of R: distinctly shorter than r-rs. Length of R1: approximately as long as 2.0× length of r-rs.

Color of metasoma in female: yellow with variable pale brown patches; yellow throughout. Color of metasoma in male: variably yellow to pale brown. Horn on T1 in female: absent. Sculpture of posterior margin of T1 in female: longitudinally striate throughout. Sculpture of T1 in male: longitudinally striate. Development of longitudinal striae on T2 in female: reaching posterior margin of T2. Sculpture of T3: smooth. Shape of T6 in female: short, wider than long. Sculpture of S3: smooth.

**Figures 172–177.**
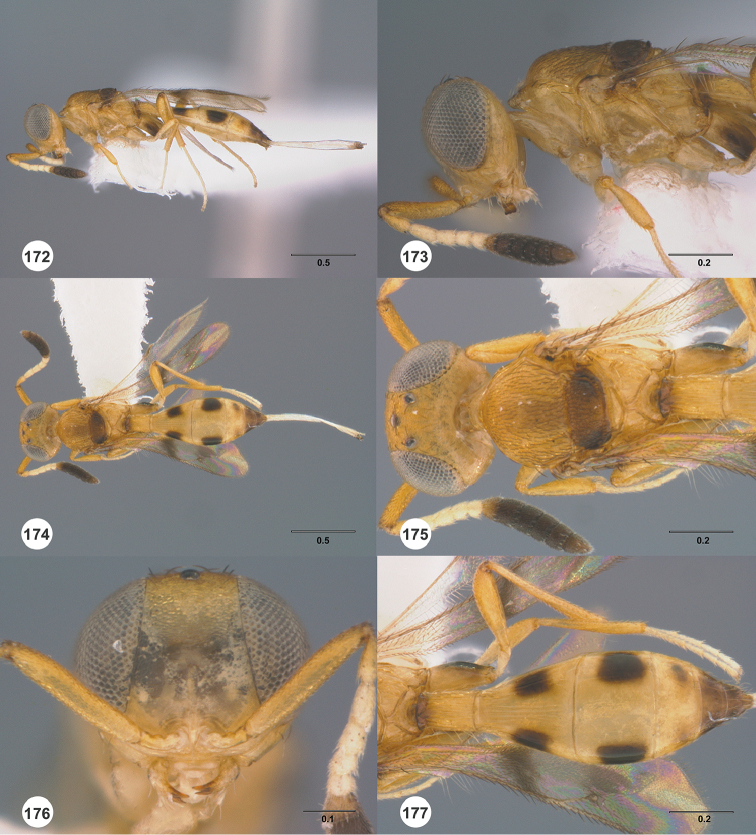
*Calliscelio minutia* sp. n., female, holotype (OSUC 433847). **172** Lateral habitus **173** Head and mesosoma, lateral view **174** Dorsal habitus **175** Head and mesosoma, dorsal view **176** Head, anterior view **177** Metasoma, dorsal view. Scale bars in millimeters.

#### Diagnosis.

This species is most similar to *Calliscelio suni* in color, size and habitus, and it is also similar to small specimens of *Calliscelio sfina*. It can be separated by the presence of an infuscate band in the middle of the fore wing.

#### Etymology.

The epithet is used as a noun in apposition derived from the Latin word for smallness, in reference to the body size.

#### Link to distribution map.

[http://hol.osu.edu/map-full.html?id=363279]

#### Material examined.

Holotype, female: **BRAZIL**: BA, Mata da Esperança, YPT T3, 14°46'S 39°04'W, Ilheus, 18.V–21.V.2002, yellow pan trap, A. M. Penteado-Dias et al., OSUC 433847 (deposited in MZSP). Paratypes: (23 females, 8 males) **BRAZIL**: 23 females, 7 males, OSUC 534541 (CNCI); OSUC 433803, 433818 (MZSP); OSUC 150797–150798, 150800, 150965, 150973, 322554–322556, 367440–367441, 378985, 427459–427460, 427462–427463, 433806–433807, 433820, 433822, 433826–433827, 433849–433851, 577073, 583209, 583246 (OSUC). **COLOMBIA**: 1 male, OSUC 193408 (OSUC).

### Calliscelio paraglaber

Taxon classificationAnimaliaHymenopteraPlatygastridae

Chen & Johnson sp. n.

http://zoobank.org/4C30C899-9BA9-429D-A1EB-C1D4DA175750

http://bioguid.osu.edu/xbiod_concepts/362057

Figures 178–183

#### Description.

Body length of female: 1.73–2.27 mm (n=20). Body length of male: 1.60–1.63 mm (n=14). Color of head: black throughout. Color of antennal clava (A7–A12): dark brown to black. Shape of head: subglobose. Central keel of frons: absent. Setation of upper frons: with sparse, long setae. IOS/EH: IOS distinctly less than EH. Sculpture of ventrolateral frons: smooth. Sculpture of frons below median ocellus: smooth. Sculpture of posterior vertex: smooth. Hyperoccipital carina: absent. Occipital carina medially: weakly indicated, irregularly sculptured. Length of OOL: less than 0.5× ocellar diameter. Sculpture of postgena behind outer orbit: smooth. Ocular setae: absent. A4 in female: distinctly shorter than A3. A5 in female: shorter than A3, distinctly longer than wide. Shape of female A6: as long as wide. Form of male antennal flagellomeres: filiform, A11 approximately 4.0× longer than wide. Length of A5 tyloid in male: approximately 0.3× length of A5.

Color of mesosoma in female: black throughout. Color of mesosoma in male: black throughout. Sculpture of dorsal pronotal area: rugose. Sculpture of lateral pronotal area: smooth throughout. Sculpture of netrion: smooth. Notaulus: percurrent or nearly so. Sculpture of mesoscutum: smooth throughout. Shape of mesoscutellum: semiellipsoidal. Foveolae of scutoscutellar sulcus between notauli: smaller than those along margin of axilla. Sculpture of mesoscutellum: smooth throughout. Shape of metascutellum: posterior margin somewhat rounded, approximately 4.0× wider than long. Sculpture of metascutellum in female: smooth. Sculpture of metascutellum in male: smooth. Dorsal propodeum in female: not excavate medially, lateral propodeal carinae meeting anteromedially. Sculpture of dorsal propodeum in female: rugose. Sculpture of dorsal propodeum in male: rugose. Median keels on propodeum in female: absent. Mesopleural carina: absent. Sculpture of mesepisternum below mesopleural depression: smooth. Sculpture of ventral metapleural area: smooth. Color of legs: pale yellow throughout. Sculpture of hind coxa: smooth.

Color of fore wing: hyaline. Rs+M: nebulose, weakly pigmented. Setae on R: long, erect, surpassing the margin of the wing. Length of R: approximately as long as r-rs. Length of R1: greater than 3.0× length of r-rs.

Color of metasoma in female: T1 yellow, otherwise dark brown to black. Color of metasoma in male: brown throughout. Horn on T1 in female: weakly developed. Sculpture of T1 horn dorsally: smooth. Sculpture of posterior margin of T1 in female: longitudinally striate throughout. Sculpture of T1 in male: longitudinally striate. Development of longitudinal striae on T2 in female: reaching the middle of T2 medially; reaching posterior margin of T2. Sculpture of T3: smooth with longitudinal submedian striae. Shape of T6 in female: short, slightly longer than wide. Sculpture of S3: largely smooth with sparse and fine punctures.

**Figures 178–183.**
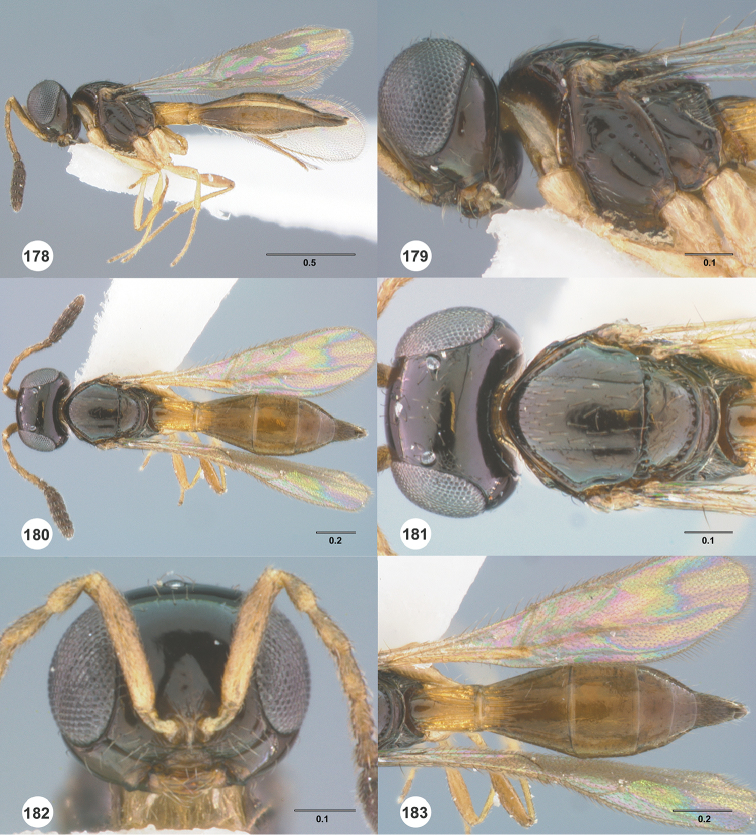
*Calliscelio paraglaber* sp. n., female, holotype (OSUC 532684). **178** Lateral habitus **179** Head and mesosoma, lateral view **180** Dorsal habitus **181** Head and mesosoma, dorsal view **182** Head, anterior view **183** Metasoma, dorsal view. Scale bars in millimeters.

#### Diagnosis.

This species is most similar to *Calliscelio glaber* and *Calliscelio torqueo* in its smooth head and metascutellum. It may be separated from those two species by the complete occipital carina.

#### Etymology.

The name refers to the high degree of similarity with *Calliscelio glaber* and is intended to be used as a noun in apposition.

#### Link to distribution map.

[http://hol.osu.edu/map-full.html?id=362057]

#### Material examined.

Holotype, female: **COSTA RICA**: Puntarenas Prov., 24km W Piedras Blancas, Golfo Dulce, 200m, VI.1989–VIII.1989, Hanson & Goulet, OSUC 532684 (deposited in CNCI). Paratypes: (41 females, 14 males) **COLOMBIA**: 3 females, OSUC 191100–191101, 269212 (OSUC). **COSTA RICA**: 32 females, 14 males, OSUC 532459–532460, 532462, 532491, 532507–532510, 532517, 532521, 532527–532530, 532533, 532551, 532553–532554, 532576, 532589, 532592–532594, 532597–532601, 532610, 532612–532613, 532667–532669, 532673, 532677, 532682, 532686, 532688–532689, 532691–532692, 532708, 532783, 532791, 532929 (CNCI). **GUYANA**: 2 females, OSUC 534274–534275 (CNCI). **MEXICO**: 2 females, OSUC 534013, 557255 (CNCI). **VENEZUELA**: 2 females, OSUC 557665, 557671 (CNCI).

### Calliscelio pararemigio

Taxon classificationAnimaliaHymenopteraPlatygastridae

Chen & Masner sp. n.

http://zoobank.org/D91754CD-B065-431B-B920-3428604B4DF5

http://bioguid.osu.edu/xbiod_concepts/362061

Figures 184–189

#### Description.

Body length of female: 2.53–3.38 mm (n=10). Body length of male: 2.11–2.75 mm (n=5). Color of head: black throughout. Color of antennal clava (A7–A12): dark brown to black; A12 pale yellow, remainder dark brown to black. Shape of head: subglobose. Central keel of frons: absent. Setation of upper frons: with sparse, long setae. IOS/EH: IOS distinctly less than EH. Sculpture of ventrolateral frons: smooth with sparse punctures. Sculpture of frons below median ocellus: smooth. Sculpture of posterior vertex: smooth. Hyperoccipital carina: absent. Occipital carina medially: interrupted. Length of OOL: greater than 0.5× ocellar diameter. Sculpture of postgena behind outer orbit: smooth. Ocular setae: absent. A4 in female: as long as A3. A5 in female: shorter than A3, distinctly longer than wide. Shape of female A6: distinctly longer than wide. Form of male antennal flagellomeres: filiform, A11 approximately 3.0× longer than wide. Length of A5 tyloid in male: approximately 0.3× length of A5.

Color of mesosoma in female: orange throughout; variably yellow to pale brown. Color of mesosoma in male: orange throughout. Sculpture of dorsal pronotal area: rugose. Sculpture of lateral pronotal area: smooth anteriorly, granulate posteriorly. Sculpture of netrion: rugulose. Notaulus: percurrent or nearly so. Sculpture of mesoscutum: smooth with sparse punctures; coriaceous; densely punctate. Shape of mesoscutellum: semiellipsoidal. Foveolae of scutoscutellar sulcus between notauli: smaller than those along margin of axilla. Sculpture of mesoscutellum: smooth with sparse fine punctures. Shape of metascutellum: posterior margin somewhat rounded, approximately 4.0× wider than long. Sculpture of metascutellum in female: finely crenulate. Sculpture of metascutellum in male: finely crenulate. Dorsal propodeum in female: deeply excavate medially, with lateral propodeal carinae widely separated, running subparallel to accommodate T1 horn. Sculpture of dorsal propodeum in female: rugose. Sculpture of dorsal propodeum in male: rugose with one or two longitudinal keels lateral median keel. Median keels on propodeum in female: absent. Mesopleural carina: present. Sculpture of mesepisternum below mesopleural depression: smooth. Sculpture of ventral metapleural area: smooth. Color of legs: coxae to femur white, remainder of the legs pale yellow; pale yellow throughout. Sculpture of hind coxa: smooth.

Color of fore wing: hyaline. Rs+M: spectral. Setae on R: long, erect, surpassing the margin of the wing. Length of R: approximately as long as r-rs. Length of R1: approximately as long as 2.0× length of r-rs.

Color of metasoma in female: orange throughout; variably orange to pale brown. Color of metasoma in male: orange throughout; variably orange to pale brown. Horn on T1 in female: large and distinct. Sculpture of T1 horn dorsally: densely and concentrically striate anteriorly, smooth posteriorly. Sculpture of posterior margin of T1 in female: longitudinally striate throughout. Sculpture of T1 in male: longitudinally striate. Development of longitudinal striae on T2 in female: reaching posterior margin of T2. Sculpture of T3: smooth with longitudinal submedian striae. Shape of T6 in female: distinctly elongate, approximately 3.0× longer than wide. Sculpture of S3: largely smooth with sparse and fine punctures.

**Figures 184–189.**
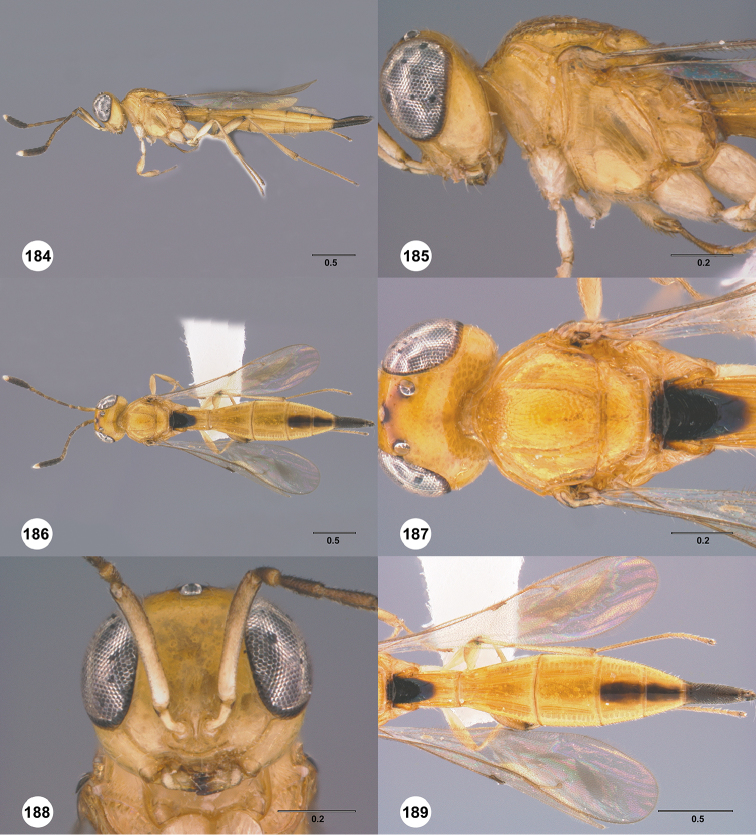
*Calliscelio pararemigio* sp. n., female, holotype (OSUC 458239). **184** Lateral habitus **185** Head and mesosoma, lateral view **186** Dorsal habitus **187** Head and mesosoma, dorsal view **188** Head, anterior view **189** Metasoma, dorsal view. Scale bars in millimeters.

#### Diagnosis.

This species is most similar to *Calliscelio remigio* but can be distinguished by its subglobose head and densely and concentrically striate T1 horn on anterior portion in female. In males, it can be separated from *Calliscelio remigio* by the subglobose head and the largely smooth S3.

#### Etymology.

The specific epithet is a reference to the high degree of similarity with *Calliscelio remigio* and is intended to be used as a noun in apposition.

#### Link to distribution map.

[http://hol.osu.edu/map-full.html?id=362061]

#### Material examined.

Holotype, female: **CUBA**: Santiago de Cuba Prov., La Isabelica, environs of Gran Piedra Mountain, 1100m, 6.XII–7.XII.1995, screen sweeping, L. Masner, OSUC 458239 (deposited in CNCI). Paratypes: (9 females, 5 males) **CUBA**: 1 female, OSUC 458240 (CNCI). **DOMINICAN REPUBLIC**: 8 females, 5 males, CMNH-486,653, 490,811 (CMNH); OSUC 458302, 458315–458324 (CNCI).

### Calliscelio prolixus

Taxon classificationAnimaliaHymenopteraPlatygastridae

Chen & Johnson sp. n.

http://zoobank.org/E287C8E0-BBF5-4850-914A-7B0C4BC1548F

http://bioguid.osu.edu/xbiod_concepts/384175

Figures 190–195

#### Description.

Body length of female: 2.00–2.48 mm (n=11). Color of head: dark brown; orange to pale brown. Color of antennal clava (A7–A12): dark brown to black; A7 yellow, remainder dark brown to black. Shape of head: subglobose. Central keel of frons: present. Setation of upper frons: with dense, short setae. IOS/EH: IOS distinctly less than EH. Sculpture of ventrolateral frons: granulate. Sculpture of frons below median ocellus: smooth to coriaceous. Sculpture of posterior vertex: granulate. Hyperoccipital carina: absent. Occipital carina medially: complete, weakly crenulate throughout. Length of OOL: less than 0.5× ocellar diameter. Sculpture of postgena behind outer orbit: smooth. Ocular setae: absent. A4 in female: distinctly longer than A3. A5 in female: longer than A3, distinctly longer than wide. Shape of female A6: distinctly longer than wide.

Color of mesosoma in female: variably orange to pale brown. Sculpture of dorsal pronotal area: rugose. Sculpture of lateral pronotal area: smooth throughout. Sculpture of netrion: smooth. Notaulus: percurrent or nearly so. Sculpture of mesoscutum: coriaceous. Shape of mesoscutellum: semiellipsoidal. Foveolae of scutoscutellar sulcus between notauli: as large as those along margin of axilla. Sculpture of mesoscutellum: smooth with sparse fine punctures. Shape of metascutellum: posterior margin straight, approximately 4.0× wider than long. Sculpture of metascutellum in female: rugose. Dorsal propodeum in female: not excavate medially, lateral propodeal carinae meeting anteromedially. Sculpture of dorsal propodeum in female: rugose with one or two longitudinal keels lateral median keel. Median keels on propodeum in female: absent. Mesopleural carina: present. Sculpture of mesepisternum below mesopleural depression: smooth. Sculpture of ventral metapleural area: smooth. Color of legs: variably orange to pale brown. Sculpture of hind coxa: smooth.

Color of fore wing: hyaline. Rs+M: nebulose, strongly pigmented. Setae on R: long, erect, surpassing the margin of the wing. Length of R: distinctly longer than r-rs. Length of R1: greater than 3.0× length of r-rs.

Color of metasoma in female: variably yellow to pale brown. Horn on T1 in female: absent. Sculpture of posterior margin of T1 in female: longitudinally striate throughout. Development of longitudinal striae on T2 in female: reaching the middle of T2 medially. Sculpture of T3: smooth. Shape of T6 in female: short, slightly longer than wide. Sculpture of S3: smooth.

**Figures 190–195.**
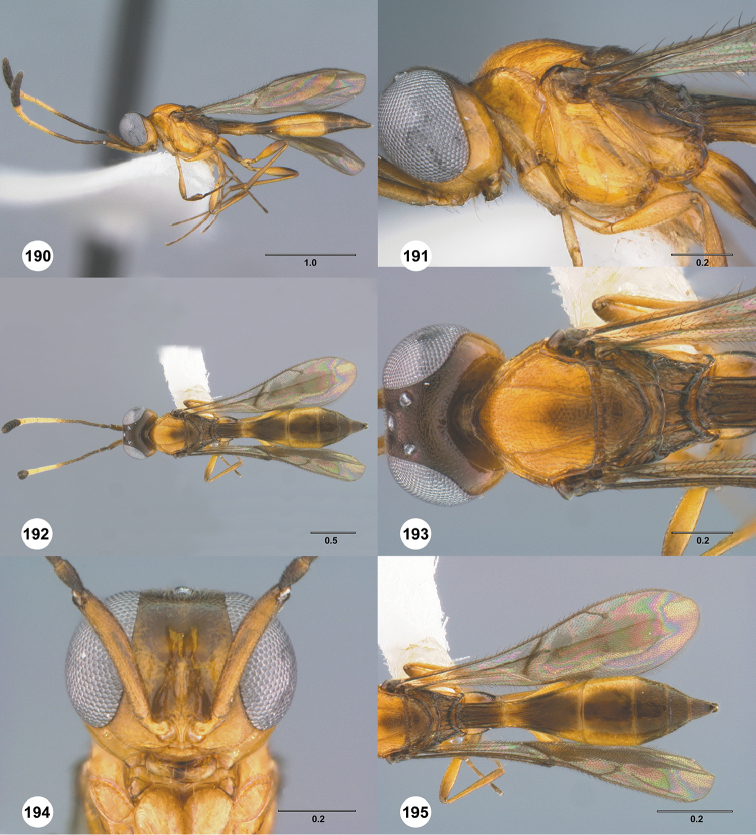
*Calliscelio prolixus* sp. n., female, holotype (OSUC 557587). **190** Lateral habitus **191** Head and mesosoma, lateral view **192** Dorsal habitus **193** Head and mesosoma, dorsal view **194** Head, anterior view **195** Metasoma, dorsal view. Scale bars in millimeters.

#### Diagnosis.

This species is most similar to *Calliscelio longius* in elongate antenna, color and habitus. It can be distinguished by the absence of T1 horn and the subtriangular T6 in female.

#### Etymology.

The epithet is an adjective, Latin word for long, in reference to the elongate antenna.

#### Link to distribution map.

[http://hol.osu.edu/map-full.html?id=384175]

#### Material examined.

Holotype, female: **COLOMBIA**: Valle del Cauca Dept., 650m, 03°26'N, 76°48'W, Farallones de Cali Natural National Park, 19.XII–2.I.2001, Malaise trap, S. Sarria, OSUC 557587 (deposited in CNCI). Paratypes: (10 females) **BRAZIL**: 1 female, OSUC 322538 (OSUC). **COLOMBIA**: 4 females, OSUC 557588–557589, 557591 (CNCI); OSUC 268908 (OSUC). **ECUADOR**: 5 females, OSUC 553448, 553453, 553472, 553516, 553656 (CNCI).

### Calliscelio punctatifrons

Taxon classificationAnimaliaHymenopteraPlatygastridae

Chen & Johnson sp. n.

http://zoobank.org/ACEE562D-FAE5-4C8C-8DE7-92FE3B7ADF08

http://bioguid.osu.edu/xbiod_concepts/363280

Figures 1–3 , 196–201

#### Description.

Body length of female: 1.80–2.56 mm (n=20). Body length of male: 1.45–1.88 mm (n=20). Color of head: black throughout. Color of antennal clava (A7–A12): black. Shape of head: subglobose. Central keel of frons: absent. Setation of upper frons: with dense, long setae. IOS/EH: IOS distinctly less than EH. Sculpture of ventrolateral frons: irregularly punctate. Sculpture of frons below median ocellus: densely punctate. Sculpture of posterior vertex: densely punctate. Hyperoccipital carina: absent. Occipital carina medially: complete, strongly crenulate throughout. Length of OOL: less than 0.5× ocellar diameter. Sculpture of postgena behind outer orbit: granulate with sparse punctures. Ocular setae: dense, long. A4 in female: distinctly shorter than A3. A5 in female: shorter than A3, as long as wide. Shape of female A6: distinctly wide than long. Form of male antennal flagellomeres: filiform, A11 approximately 2.5× longer than wide. Length of A5 tyloid in male: greater than 0.5× length of A5.

Color of mesosoma in female: black throughout. Color of mesosoma in male: black throughout. Sculpture of dorsal pronotal area: areolate. Sculpture of lateral pronotal area: smooth throughout. Sculpture of netrion: striate. Notaulus: percurrent or nearly so. Sculpture of mesoscutum: smooth with sparse punctures; coriaceous anteriorly, smooth with sparse fine punctures posteriorly. Shape of mesoscutellum: semiellipsoidal. Foveolae of scutoscutellar sulcus between notauli: as large as those along margin of axilla. Sculpture of mesoscutellum: smooth with sparse fine punctures. Shape of metascutellum: posterior margin straight, approximately 2.5× wider than long. Sculpture of metascutellum in female: rugose; rugose anteriorly, smooth posteriorly. Sculpture of metascutellum in male: rugose. Dorsal propodeum in female: shallowly excavate medially, with lateral propodeal carinae widely separated. Sculpture of dorsal propodeum in female: rugose. Sculpture of dorsal propodeum in male: rugose. Median keels on propodeum in female: absent. Mesopleural carina: present. Sculpture of mesepisternum below mesopleural depression: largely smooth with a row of foveae along mesopleural carina. Sculpture of ventral metapleural area: largely smooth, rugose ventrally. Color of legs: orange throughout; pale yellow throughout. Sculpture of hind coxa: smooth.

Color of fore wing: subhyaline. Rs+M: nebulose, weakly pigmented. Setae on R: long, erect, surpassing the margin of the wing. Length of R: distinctly shorter than r-rs. Length of R1: approximately as long as 2.0× length of r-rs.

Color of metasoma in female: orange throughout; reddish brown to black. Color of metasoma in male: variably orange to pale brown; brown throughout; reddish brown. Horn on T1 in female: present as a small bulge. Sculpture of T1 horn dorsally: smooth. Sculpture of posterior margin of T1 in female: longitudinally striate throughout. Sculpture of T1 in male: longitudinally striate. Development of longitudinal striae on T2 in female: reaching the middle of T2 medially. Sculpture of T3: smooth. Shape of T6 in female: short, slightly longer than wide. Sculpture of S3: largely smooth with sparse and fine punctures.

**Figures 196–201.**
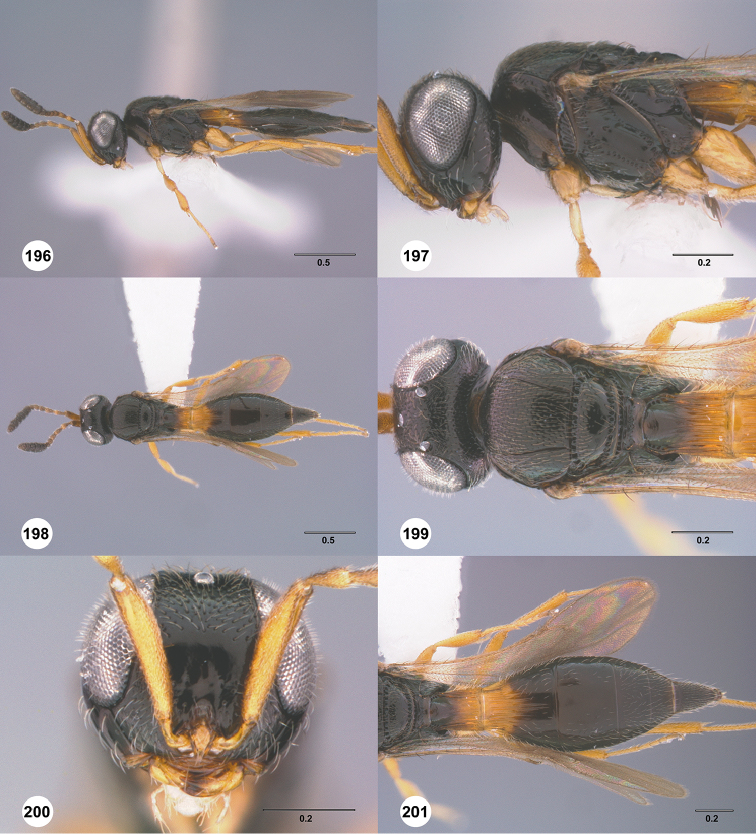
*Calliscelio punctatifrons* sp. n., female, holotype (OSUC 191124). **196** Lateral habitus **197** Head and mesosoma, lateral view **198** Dorsal habitus **199** Head and mesosoma, dorsal view **200** Head, anterior view **201** Metasoma, dorsal view. Scale bars in millimeters.

#### Diagnosis.

This species (especially small specimens) is most similar to *Calliscelio bisulcatus* but can be distinguished by the long setae on upper frons and pigmented Rs+M.

#### Etymology.

The specific epithet refers to the punctate frons and should be treated as a noun in apposition.

#### Link to distribution map.

[http://hol.osu.edu/map-full.html?id=363280]

#### Material examined.

Holotype, female: **COLOMBIA**: Bolívar Dept., La Suiris, M.617, 126m, 09°54'N 75°07'W, Los Colorados Fauna and Flora Sanctuary, 12.IX–15.IX.2000, Malaise trap, E. Deulufeut, OSUC 191124 (deposited in IAVH). Paratypes: (192 females, 55 males) **BELIZE**: 32 females, 13 males, OSUC 534198, 534201, 534204–534205, 534211–534213, 534219–534221, 534276, 534278, 534280–534282, 534285–534289, 534293–534300, 534309, 534312 (CNCI); OSUC 185848, 185852, 243690–243691, 48053, 91681, 91684, 91688, 91691, 91697–91698, 91701, 91704, 93637–93638 (OSUC). **BOLIVIA**: 4 females, OSUC 534185, 534187, 534190–534191 (CNCI). **BRAZIL**: 1 female, 7 males, OSUC 557160–557165, 557296 (CNCI); OSUC 133029 (OSUC). **COLOMBIA**: 20 females, 14 males, OSUC 557409–557413, 557422–557427, 557458–557470, 557609, 557611, 557619 (CNCI); OSUC 191128, 191140 (IAVH); OSUC 182753, 191123, 191125, 191142, 259758 (OSUC). COSTA RICA: 37 females, OSUC 532465, 532469–532470, 532474, 532487–532490, 532497, 532502, 532543, 532558–532560, 532563, 532580–532582, 532623, 532625, 532640–532641, 532644, 532647–532650, 532653, 532697, 532775, 532781, 532823–532824, 532924, 532930, 534137 (CNCI); OSUC 237331 (OSUC). **ECUADOR**: 49 females, 4 males, OSUC 458482, 458484, 458495, 458537–458542, 534238, 553372, 553381, 553413, 553416, 553427–553430, 553432, 553479–553482, 553494, 553528, 553545, 553605, 553630, 553652, 553657–553666, 553668, 553670–553671, 553677, 553689, 553701–553704, 553718, 553731–553732, 553736, 553738 (CNCI). **HONDURAS**: 11 females, OSUC 399378, 399384–399385, 399392, 399394, 399396, 399400, 399405, 410439, 410442, 410446 (MZLU). **MEXICO**: 19 females, 5 males, OSUC 532626, 534015–534017, 534021–534023, 534026, 534461–534462, 534471, 534476, 534483–534486 (CNCI); OSUC 375848–375852, 415020, 55929, 55951 (OSUC). **PANAMA**: 3 females, 10 males, OSUC 534065, 553822, 553892–553894, 553896–553899, 553910, 553912, 553922, 553948 (CNCI). **PERU**: 6 females, 2 males, OSUC 534396–534397, 534399, 534401, 553968, 553988, 554026, 554028 (CNCI). **TRINIDAD AND TOBAGO**: 8 females, OSUC 545997, 546030–546032, 546084, 557319–557321 (CNCI). **URUGUAY**: 2 females, OSUC 534608–534609 (CNCI).

### Calliscelio remigio

Taxon classificationAnimaliaHymenopteraPlatygastridae

Chen & Masner sp. n.

http://zoobank.org/CB62A4C2-F171-4C23-B065-A63A0A197B7A

http://bioguid.osu.edu/xbiod_concepts/362059

Figures 202–207

#### Description.

Body length of female: 2.56–2.96 mm (n=16). Body length of male: 2.45–2.70 mm (n=7). Color of head: black throughout. Color of antennal clava (A7–A12): dark brown to black. Shape of head: strongly transverse. Central keel of frons: absent. Setation of upper frons: with sparse, long setae. IOS/EH: IOS distinctly less than EH. Sculpture of ventrolateral frons: smooth with sparse punctures. Sculpture of frons below median ocellus: smooth. Sculpture of posterior vertex: smooth. Hyperoccipital carina: absent. Occipital carina medially: interrupted. Length of OOL: greater than 0.5× ocellar diameter. Sculpture of postgena behind outer orbit: smooth. Ocular setae: absent. A4 in female: as long as A3. A5 in female: shorter than A3, distinctly longer than wide. Shape of female A6: distinctly longet than wide. Form of male antennal flagellomeres: filiform, A11 approximately 3.0× longer than wide. Length of A5 tyloid in male: approximately 0.3× length of A5.

Color of mesosoma in female: orange with longitudinal, median black strip on anterior mesoscutum. Color of mesosoma in male: orange with longitudinal, median black strip on anterior mesoscutum. Sculpture of dorsal pronotal area: smooth. Sculpture of lateral pronotal area: smooth anteriorly, punctate rugulose posteriorly. Sculpture of netrion: rugulose. Notaulus: percurrent or nearly so. Sculpture of mesoscutum: smooth with sparse punctures; densely punctate. Shape of mesoscutellum: semiellipsoidal. Foveolae of scutoscutellar sulcus between notauli: smaller than those along margin of axilla. Sculpture of mesoscutellum: smooth with sparse fine punctures. Shape of metascutellum: posterior margin straight, approximately 3.0× wider than long. Sculpture of metascutellum in female: finely crenulate. Sculpture of metascutellum in male: finely crenulate. Dorsal propodeum in female: shallowly excavate medially, with lateral propodeal carinae widely separated. Sculpture of dorsal propodeum in female: rugose. Sculpture of dorsal propodeum in male: rugose with one or two longitudinal keels lateral median keel. Median keels on propodeum in female: absent. Mesopleural carina: present. Sculpture of mesepisternum below mesopleural depression: smooth. Sculpture of ventral metapleural area: smooth. Color of legs: coxae to femur white, remainder of the legs pale yellow; pale yellow throughout. Sculpture of hind coxa: smooth.

Color of fore wing: hyaline. Rs+M: spectral. Setae on R: long, erect, surpassing the margin of the wing. Length of R: approximately as long as r-rs. Length of R1: approximately as long as 2.0× length of r-rs.

Color of metasoma in female: variably orange to pale brown. Color of metasoma in male: orange throughout; variably orange to pale brown. Horn on T1 in female: present as a small bulge. Sculpture of T1 horn dorsally: transversely striate. Sculpture of posterior margin of T1 in female: longitudinally striate throughout. Sculpture of T1 in male: longitudinally striate. Development of longitudinal striae on T2 in female: reaching posterior margin of T2. Sculpture of T3: smooth with longitudinal submedian striae. Shape of T6 in female: distinctly elongate, approximately 3.0× longer than wide. Sculpture of S3: densely punctate; punctate rugose.

**Figures 202–207.**
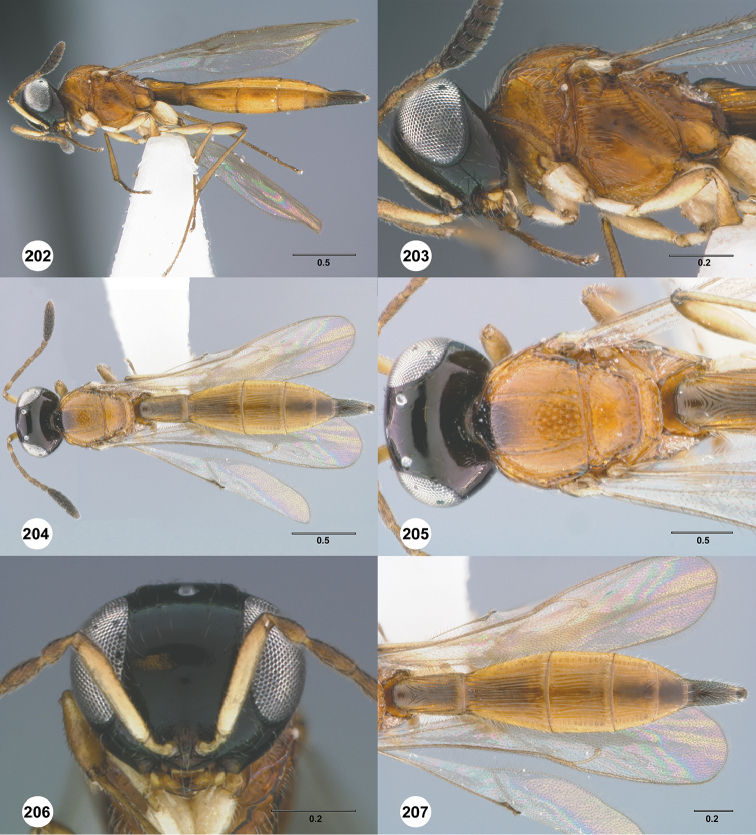
*Calliscelio remigio* sp. n., female, holotype (OSUC 458296). **202** Lateral habitus **203** Head and mesosoma, lateral view **204** Dorsal habitus **205** Head and mesosoma, dorsal view **206** Head, anterior view **207** Metasoma, dorsal view. Scale bars in millimeters.

#### Diagnosis.

This species is most similar to *Calliscelio pararemigio* but can be distinguished by its strongly transverse head and transversely striate small T1 horn in female. In males, it can be separated from *Calliscelio pararemigio* by the strongly transverse head and the densely punctate or punctate rugose S3.

#### Etymology.

The specific epithet refers to the locality, Mt. Remigio, on the label of the holotype and should be treated as a noun in apposition.

#### Link to distribution map.

[http://hol.osu.edu/map-full.html?id=362059]

#### Material examined.

Holotype, female: **DOMINICAN REPUBLIC**: Barahona Prov., Baoruco (Bahoruco) Mts., cloud forest, DR-13, Remigio Knoll, 800m, 26.III.1991, L. Masner, OSUC 458296 (deposited in CNCI). Paratypes: **DOMINICAN REPUBLIC**: 15 females, 7 males, CMNH-486,650, 486,686, 486,897 (CMNH); OSUC 458285–458295, 458297–458301, 458303, 534377–534378 (CNCI).

### Calliscelio rubriclavus

Taxon classificationAnimaliaHymenopteraPlatygastridae

(Ashmead) comb. n.

http://zoobank.org/B78820F5-FCE1-4A15-BC59-12B9A1C1AAE4

http://bioguid.osu.edu/xbiod_concepts/4169

Figures 208–213 , 214–219 , 220–225

Acolus rubriclavus Ashmead, 1887: 99 (original description).
Anteris nigriceps Ashmead, 1893: 225, 226 (original description); Muesebeck 1958: 93 [junior synonym of Ceratoteleia marlatti (Ashmead)], **syn. n.**http://zoobank.org/9C37051A-432A-4772-A8EC-C1345A2F32D4http://bioguid.osu.edu/xbiod_concepts/8446
Caloteleia Marlattii Ashmead, 1893: 212, 214 (original description, keyed); Harrington 1900: 187 (variation); Brues 1903: 126 (emendation of male description), **syn. n.**http://zoobank.org/91C406E3-85AA-4C93-8CE5-D160876C5847http://bioguid.osu.edu/xbiod_concepts/8445
Caloteleia rubriclava (Ashmead): Ashmead 1893: 212, 214 (generic transfer, description, keyed).
Caloteleia grenadensis Ashmead, 1896: 798 (original description); Ashmead 1900: 327 (distribution), **syn. n.**http://zoobank.org/E41DB9D7-F6E1-49BA-BB67-4C8A37430374http://bioguid.osu.edu/xbiod_concepts/8437
Ceratoteleia Marlatti (Ashmead): Kieffer 1908: 121 (generic transfer, emendation).
Ceratoteleia grenadensis (Ashmead): Kieffer 1908: 121 (generic transfer); Kieffer 1926: 501, 505 (description, keyed).
Ceratoteleia rubriclava (Ashmead): Kieffer 1908: 121 (generic transfer); Kieffer 1926: 501, 504 (description, keyed).
Prosanteris nigriceps (Ashmead): Kieffer 1908: 136 (generic transfer); Kieffer 1926: 437, 438 (description, keyed).
Caloteleia rubriclavus (Ashmead): Brues 1908: 33 (emendation).
Prosanteris (Prosanteris) nigriceps (Ashmead): Kieffer 1910b: 87 (subgeneric assignment).
Caloteleia marlattii (Ashmead): Brues 1916: 554 (description); Masner and Muesebeck 1968: 33 (lectotype designation).
Macroteleia ruskini Girault, 1920: 179 (original description); Muesebeck 1958: 93 [junior synonym of Ceratoteleia marlatti (Ashmead)], **syn. n.**http://zoobank.org/94B4FD7F-E840-437B-8E2B-46F87BE377DFhttp://bioguid.osu.edu/xbiod_concepts/8447
Ceratoteleia marlattii (Ashmead): Kieffer 1926: 501, 504 (description, keyed).
Calotelea grenadensis (Ashmead): Masner 1965: 70 (type information).
Calotelea nigriceps (Ashmead): Masner and Muesebeck 1968: 33 (type information).
Calotelea rubriclava (Ashmead): Masner and Muesebeck 1968: 33 (type information).
Calotelea ruskini (Girault): Masner and Muesebeck 1968: 33 (type information, generic transfer).

#### Description.

Body length of female: 1.80–2.55 mm (n=20). Body length of male: 1.60–2.35 mm (n=20). Color of head: black throughout; brown throughout. Color of antennal clava (A7–A12): dark brown to black. Shape of head: subglobose. Central keel of frons: absent. Setation of upper frons: with sparse, short setae. IOS/EH: IOS slightly less than EH; IOS slightly greater than EH. Sculpture of ventrolateral frons: granulate. Sculpture of frons below median ocellus: granulate. Sculpture of posterior vertex: granulate. Hyperoccipital carina: absent. Occipital carina medially: complete, weakly crenulate throughout. Length of OOL: less than 0.5× ocellar diameter; greater than 0.5× ocellar diameter. Sculpture of postgena behind outer orbit: granulate. Ocular setae: absent. A4 in female: distinctly shorter than A3. A5 in female: shorter than A3, as long as wide. Shape of female A6: distinctly wider than long. Form of male antennal flagellomeres: filiform, A11 approximately 2.0× longer than wide. Length of A5 tyloid in male: greater than 0.5× length of A5.

Color of mesosoma in female: orange throughout; black throughout; variably orange to pale brown. Color of mesosoma in male: orange throughout; variably orange to pale brown; black throughout. Sculpture of dorsal pronotal area: rugose. Sculpture of lateral pronotal area: rugose throughout. Sculpture of netrion: rugose. Notaulus: percurrent. Sculpture of mesoscutum: granulate; densely punctate. Shape of mesoscutellum: semiellipsoidal. Foveolae of scutoscutellar sulcus between notauli: smaller than those along margin of axilla. Sculpture of mesoscutellum: granulate. Shape of metascutellum: posterior somewhat rounded or straight, approximately 3.0× wider than long. Sculpture of metascutellum in female: rugose. Sculpture of metascutellum in male: rugose. Dorsal propodeum in female: shallowly excavate medially, with lateral propodeal carinae widely separated. Sculpture of dorsal propodeum in female: rugose. Sculpture of dorsal propodeum in male: rugose. Median keels on propodeum in female: absent. Mesopleural carina: absent. Sculpture of mesepisternum below mesopleural depression: densely punctate. Sculpture of ventral metapleural area: smooth dorsally, densely punctate ventrally. Color of legs: orange throughout; pale brown. Sculpture of hind coxa: smooth.

Color of fore wing: hyaline. Rs+M: spectral. Setae on R: short, decumbent, hardly exceeding the margin of the wing. Length of R: distinctly shorter than r-rs. Length of R1: approximately as long as r-rs.

Color of metasoma in female: black; orange throughout; variably orange to pale brown. Color of metasoma in male: orange throughout; variably orange to pale brown; black throughout. Horn on T1 in female: large and distinct. Sculpture of T1 horn dorsally: granulate; rugose. Sculpture of posterior margin of T1 in female: longitudinally striate throughout. Sculpture of T1 in male: longitudinally striate. Development of longitudinal striae on T2 in female: reaching the middle of T2 medially; reaching posterior margin of T2. Sculpture of T3: smooth; largely smooth with submedian longitudinal striae; longitudinally striate throughout. Shape of T6 in female: short, slightly longer than wide. Sculpture of S3: smooth to coriaceous.

**Figures 208–213.**
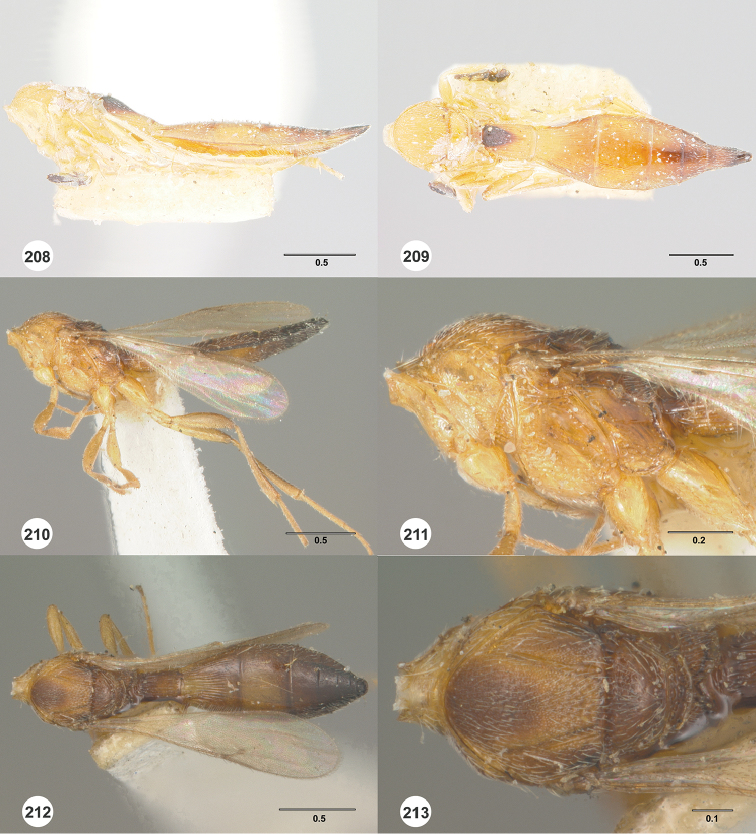
**208–209** *Acolus rubriclavus* Ashmead, female, holotype (USNMENT00989042). **208** Mesosoma and metasoma, lateral view **209** Mesosoma and metasoma, dorsal view **211**–**213** *Anteris nigriceps* Ashmead, male, syntype (USNMENT00989028). **210** Mesosoma and metasoma, lateral view **211** Mesosoma, lateral view **212** Mesosoma and metasoma, dorsal view **213** Mesosoma, dorsal view. Scale bars in millimeters.

**Figures 214–219.**
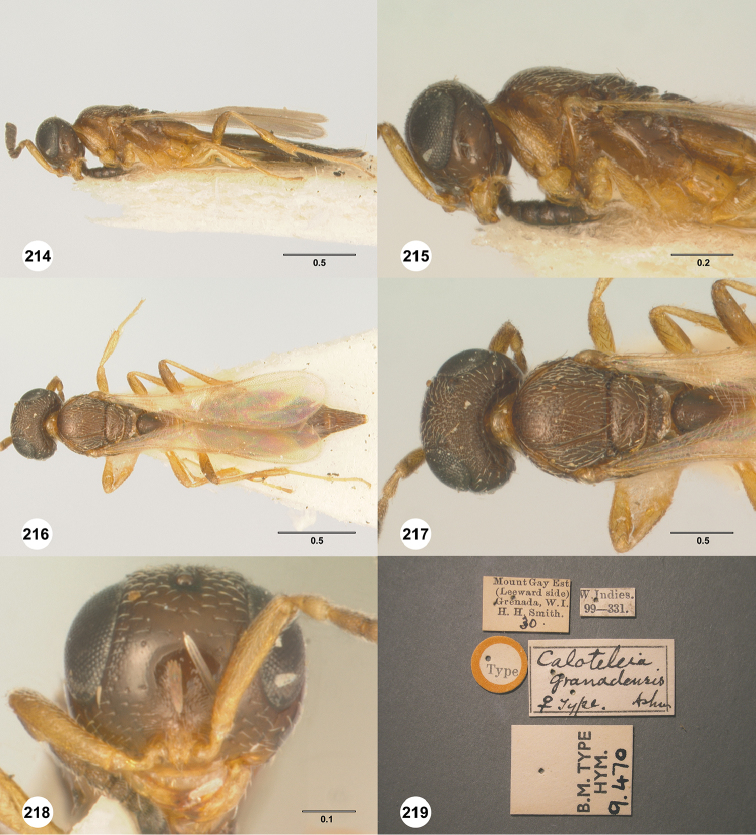
*Caloteleia grenadensis* Ashmead, female, holotype (B.M. TYPE HYM. 9.470). **214** Lateral habitus **215** Head and mesosoma, lateral view **216** Dorsal habitus **217** Head and mesosoma, dorsal view **218** Head, anterior view **219** Specimen labels. Scale bars in millimeters.

**Figures 220–225.**
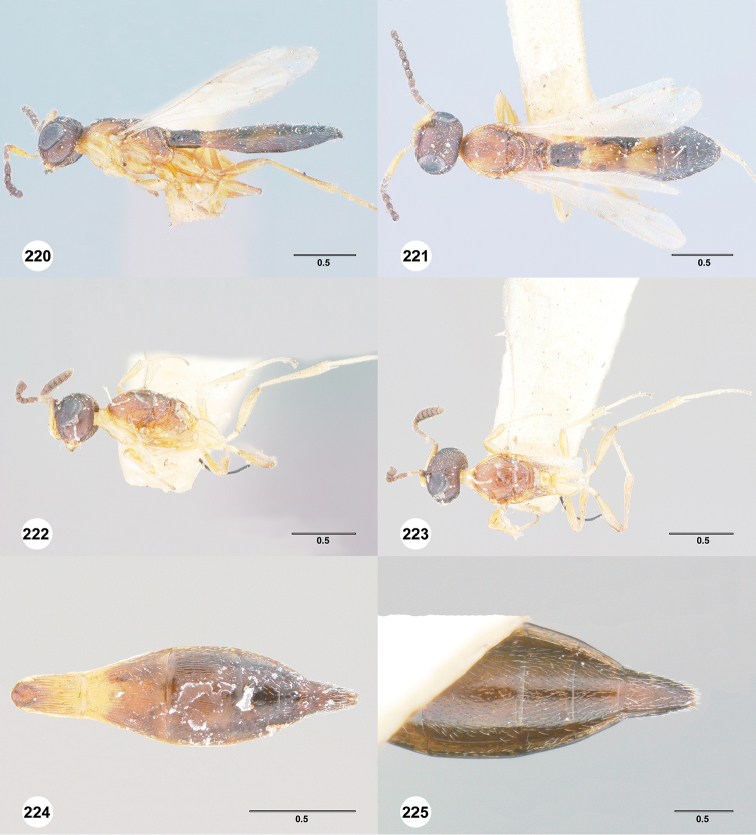
**220–221** *Caloteleia marlattii* Ashmead, male, lectotype (USNMENT00989024). **220** Lateral habitus **221** Dorsal habitus **222–225** *Macroteleia ruskini* Girault, female, holotype (USNMENT00989079) **222** Mesosoma and metasoma, lateral view **223** Mesosoma and metasoma, dorsal view **224** Metasoma, dorsal view **225** Metasoma, ventral view. Scale bars in millimeters.

#### Diagnosis.

This species is easily identified by the combination of the following characters: eye bare; occipital carina complete medially; mesopleural carina absent; mesepisternum below mesopleural depression densely punctate; ventral metapleural area densely punctate ventrally; R1 approximately as long as r-rs.

#### Link to distribution map.

[http://hol.osu.edu/map-full.html?id=4169]

#### Material examined.

Holotype, female, *Calliscelio grenadensis*: **GRENADA**: Saint George Parish, leeward side, Mount Gay Estate, no date, H. H. Smith, B.M. TYPE HYM. 9.470 (deposited in BMNH). Lectotype, male, *C. Marlattii*: **UNITED STATES**: Riley Co., V, Marlatt, USNMENT00989024 (deposited in USNM). Syntype, male, *Anteris nigriceps*: **UNITED STATES**: VA, Arlington Co., Arlington, no date, USNMENT00989028 (deposited in USNM). Holotype, female, *Acolus rubriclavus*: **UNITED STATES**: FL, Duval Co., Jacksonville, ocean beach, Jacksonville Beach (San Pablo), no date, Ashmead, USNMENT00989042 (deposited in USNM). Holotype, female, *Macroteleia ruskini*: **UNITED STATES**: IL, no date, USNMENT00989079 (deposited in USNM). Other material: (1106 females, 610 males) **ARGENTINA**: 11 females, 2 males, OSUC 534125–534131, 534437 (CNCI); OSUC 322989, 63153, 63155 (OSUC); OSUC 577255–577256 (UCRC). **BAHAMAS**: 2 females, 2 males, OSUC 458330–458331, 458383, 534560 (CNCI). **BELIZE**: 9 females, 3 males, OSUC 534193–534197, 534203, 534214–534218, 534222 (CNCI). **BOLIVIA**: 1 female, OSUC 534164 (CNCI). **BONAIRE**: 2 females, OSUC 458377, 458382 (CNCI). **BRAZIL**: 48 females, 15 males, OSUC 534527, 553607, 557180–557181, 557197, 557220, 557246, 557248, 557282–557283, 557337, 557342, 557349, 557361, 557375 (CNCI); OSUC 111348, 111698–111700, 111704–111705, 111714, 111810, 111938, 111957, 112073, 112184–112185, 112189–112190, 112194, 112792, 112797, 113088, 11925, 130662, 130709, 130719, 130755, 130849, 131119, 131431, 132407, 132409, 132411, 132413, 132420–132421, 132433, 132446, 133028, 133046, 133050, 133067, 133130, 134362, 137997, 138091, 232034, 367498, 374725, 413, 583202 (OSUC). **CANADA**: 156 females, 68 males, OSUC 531695–531708, 531710–531720, 531725, 531728–531733, 531802–531865, 531867–531872, 531874–531917, 532076, 532103, 532123–532129, 534491–534493, 534495–534498, 534500, 534504–534507, 534509–534512, 554065–554117 (CNCI). **CHILE**: 16 females, OSUC 458265, 534444–534445, 534447, 534449, 534451–534452, 534454, 534456–534458 (CNCI); OSUC 441184–441187 (INHS); OSUC 576993 (OSUC). **COLOMBIA**: 2 females, 3 males, OSUC 557535, 557537–557539, 557550 (CNCI). **COSTA RICA**: 24 females, 3 males, OSUC 232068–232069, 532463, 532468, 532494, 532537, 532539, 532569, 532656–532658, 532664, 532711–532716, 532726, 532765–532766, 532769, 532777–532778, 532792, 532925 (CNCI); OSUC 245767 (OSUC). **CUBA**: 20 females, 3 males, OSUC 458199, 458201–458202, 458204–458205, 458207–458211, 458367–458375, 458378, 534558, 534597–534598 (CNCI). **DOMINICAN REPUBLIC**: 38 females, CMNH-486,514, 490,550, 490,915 (CMNH); OSUC 458178, 458180–458182, 458184, 458186, 458188–458190, 458192, 458194, 458329, 458358–458366, 458376, 458381, 534362–534371, 534374, 534381 (CNCI). **ECUADOR**: 15 females, OSUC 458519, 458521–458523, 458528, 553368, 553431, 553433, 553601–553602, 553624, 553726–553729 (CNCI). **FRENCH GUIANA**: 4 females, OSUC 546113–546116 (CNCI). **GUATEMALA**: 2 females, 2 males, OSUC 534430–534433 (CNCI). **HAITI**: 2 females, OSUC 534548–534549 (CNCI). **HONDURAS**: 37 females, 2 males, OSUC 534145 (CNCI); OSUC 399364–399366, 399368–399372, 399374–399377, 399379–399382, 399386–399387, 399391, 399393, 399395, 399397–399399, 399401–399404, 399407, 399409, 401745, 410436–410438, 410441, 410444–410445, 410448 (MZLU). **JAMAICA**: 6 females, OSUC 458196–458197, 458379–458380, 534617–534618 (CNCI). **KENYA**: 1 female, OSUC 56760 (OSUC). **MEXICO**: 62 females, 44 males, OSUC 531739, 533998–534000, 534004–534012, 534014, 534024, 534464, 534472–534475, 534490 (CNCI); OSUC 410663–410680, 411743, 414931–414932, 49274, 49276, 55926, 583141–583201 (OSUC). **NICARAGUA**: 2 females, OSUC 534556–534557 (CNCI). **PANAMA**: 16 females, OSUC 553860–553871, 553873–553874, 553913–553914 (CNCI). **PARAGUAY**: 53 females, 8 males, OSUC 150605, 150607, 228804–228807, 276658–276659, 278828, 322986, 322994, 322996–322997, 323036–323037, 323039–323044, 323046–323047, 323049–323053, 323056–323057, 323061–323066, 323068–323070, 323072–323074, 363636, 363708–363710, 363713, 373987–373997, 534682, 534685, 577154 (OSUC). **PERU**: 2 females, 1 male, OSUC 554022 (CNCI); OSUC 583203–583204 (OSUC). **SAINT LUCIA**: 1 female, OSUC 534550 (CNCI). **SOUTH AFRICA**: 2 males, OSUC 223550–223551 (OSUC). **SURINAME**: 1 female, OSUC 534569 (CNCI). **TRINIDAD AND TOBAGO**: 66 females, 1 male, OSUC 545994–545996, 545998, 546000, 546004–546011, 546014–546022, 546034–546049, 546053–546056, 546059, 546061–546076, 546078–546081, 546083, 546087, 546091–546092 (CNCI). **UNITED STATES**: 484 females, 451 males, OSUC 531679–531681, 531683–531685, 531687–531694, 531709, 531721–531724, 531738, 531740–531749, 531751–531775, 531781–531785, 531787–531791, 531793–531801, 531866, 531919–531939, 531948–531980, 531982–532003, 532005–532034, 532037–532044, 532046, 532048–532060, 532062–532063, 532065, 532067–532075, 532078–532099, 532101–532102, 532104–532118, 532120, 532122, 532130, 532247–532303, 532305–532311, 532313–532339, 532391–532399, 532402–532457, 534501–534503, 534508, 554118–554150 (CNCI); OSUC 332917 (MEMU); OSUC 130442, 130451, 130453, 130460, 130462, 130465, 130474, 130477, 130479, 130488, 130491, 130497, 130499–130500, 130513–130515, 130544, 130574, 142758–142787, 142789–142804, 142806, 142808–142809, 142811–142812, 182700, 182725, 207789, 207795–207796, 236917–236918, 236920, 24231, 256432, 256450–256451, 256513–256517, 256536–256538, 256557–256558, 256595, 256634–256644, 256745–256768, 332914–332916, 336798–336804, 336806–336812, 336814–336818, 336966–336986, 336988–337018, 397539–397562, 397641–397650, 410662, 55872–55879, 55881–55889, 55891–55906, 576987–576989, 577075–577089, 582301–582320, 583121, 583123–583126, 583128–583140, 583315, 62475, 62495, 62498, 62593, 628906, 62893–62894, 62906, 79811–79812 (OSUC); UCFC 0 118 312, 539, 545, 653, 906, 972, UCFC 0 119 004, UCFC 0 119 183, UCFC 0 119 232, UCFC 0 119 419, UCFC 0 119 434, UCFC 0 120 030, UCFC 0 132 372, UCFC 0 133 615, UCFC 0 133 662, UCFC 0 133 711, UCFC 0 133 782, UCFC 0 133 892, UCFC 0 133 948, UCFC 0 134 479, UCFC 0 134 588, UCFC 0 134 768, UCFC 0 134 851, UCFC 0 134 882, UCFC 0 134 902, UCFC 0 134 963, UCFC 0 135 895, UCFC 0 136 236, UCFC 0 136 271, UCFC 0 136 716, UCFC 0 138 064, UCFC 0 138 130, UCFC 0 138 265, UCFC 0 138 491, UCFC 0 138 502, UCFC 0 138 634, UCFC 0 138 639, UCFC 0 138 772, UCFC 0 138 795, UCFC 0 138 873, UCFC 0 138 898, UCFC 0 138 975, UCFC 0 139 191, UCFC 0 139 197, UCFC 0 139 395, UCFC 0 139 500, UCFC 0 139 633, UCFC 0 139 715, UCFC 0 140 509, UCFC 0 141 638, UCFC 0 142 229, UCFC 0 142 314, UCFC 0 142 556, UCFC 0 142 751, UCFC 0 142 811, UCFC 0 142 817, UCFC 0 143 684, UCFC 0 143 743, UCFC 0 144 035, UCFC 0 144 069, UCFC 0 144 169, UCFC 0 144 180, UCFC 0 144 188, UCFC 0 144 293, UCFC 0 144 568, UCFC 0 144 723, UCFC 0 144 755, UCFC 0 144 878, UCFC 0 144 974, UCFC 0 165 080, UCFC 0 165 131, UCFC 0 165 138, UCFC 0 165 192, UCFC 0 165 195, UCFC 0 165 206, UCFC 0 165 253, UCFC 0 165 546, UCFC 0 165 794, UCFC 0 165 803, UCFC 0 165 808, UCFC 0 165 925, UCFC 0 165 945, UCFC 0 165 952, UCFC 0 166 051, UCFC 0 166 425, UCFC 0 166 683, UCFC 0 166 795, UCFC 0 167 268, UCFC 0 284 802, UCFC 0 319 189, UCFC 0 373 784, UCFC 0 373 785, UCFC 0 374 236, UCFC 0 374 613, UCFC 0 380 130, UCFC 0 380 135, UCFC 0 380 143, UCFC 0 380 144, UCFC 0 380 702, UCFC 0 381 255, UCFC 0 381 256, UCFC 0 389 068, UCFC 0 389 069, UCFC 0 389 076, UCFC 0 389 077, UCFC 0 389 101, UCFC 0 389 105, UCFC 0 389 106, UCFC 0 389 109, UCFC 0 390 859, UCFC 0 391 400, UCFC 0 391 900, UCFC 0 391 901, UCFC 0 392 443, UCFC 0 392 444, UCFC 0 392 447, UCFC 0 392 449, UCFC 0 392 834, UCFC 0 393 389, UCFC 0 393 402, UCFC 0 393 403, UCFC 0 393 871, UCFC 0 393 872, UCFC 0 393 873, UCFC 0 393 874, UCFC 0 393 875, UCFC 0 393 876, UCFC 0 393 877, UCFC 0 393 878, UCFC 0 393 879, UCFC 0 394 467, UCFC 0 394 622, UCFC 0 394 623, UCFC 0 394 624, UCFC 0 394 625, UCFC 0 394 847, UCFC 0 394 848, UCFC 0 395 201, UCFC 0 395 204, UCFC 0 395 485, UCFC 0 395 896, UCFC 0 395 985 (UCFC); OSUC 157686–157687, 157689–157691 (UCMC). **VENEZUELA**: 23 females, OSUC 545831, 545852, 545862–545863, 545869, 545936, 545948–545950, 546124–546126, 557669–557670, 557674–557675, 557678–557679, 557708, 557711 (CNCI); OSUC 48276, 49158, 64554 (OSUC).

#### Comments.

This species seems to be a very common species in the New World, and we also have seen additional specimens from South Africa. Masner et al. (2009) suggested that this kind of widespread distribution may result from commercial traffic. *Calliscelio rubriclavus* exhibits variation in color, ranging from orange to black, in the shape of metascutellum, and in the sculpture of T1 horn and T3. Specimens collected from Central America tend to be darker, while the variation in the sculpture of T1 horn and T3 show no clear correlations among the distrituion ranges

### Calliscelio ruga

Taxon classificationAnimaliaHymenopteraPlatygastridae

Chen & Johnson sp. n.

http://zoobank.org/B725C08C-0768-4304-92AC-9004D072357D

http://bioguid.osu.edu/xbiod_concepts/385082

Figures 226–231

#### Description.

Body length of female: 1.42–1.74 mm (n=20). Color of head: orange throughout. Color of antennal clava (A7–A12): A7, A8 brown, A9–A12 white to pale yellow. Shape of head: subglobose. Central keel of frons: absent. Setation of upper frons: glabrous. IOS/EH: IOS distinctly less than EH. Sculpture of ventrolateral frons: smooth to granulate. Sculpture of frons below median ocellus: smooth to granulate. Sculpture of posterior vertex: rugose. Hyperoccipital carina: absent. Occipital carina medially: interrupted. Length of OOL: less than 0.5× ocellar diameter. Sculpture of postgena behind outer orbit: largely smooth with small granulate area. Ocular setae: sparse, short. A4 in female: as long as A3. A5 in female: shorter than A3, distinctly longer than wide. Shape of female A6: distinctly longer than wide.

Color of mesosoma in female: orange throughout; variably orange to pale brown. Sculpture of dorsal pronotal area: rugose. Sculpture of lateral pronotal area: smooth throughout. Sculpture of netrion: rugose. Notaulus: percurrent or nearly so. Sculpture of mesoscutum: granulate. Shape of mesoscutellum: semiellipsoidal. Foveolae of scutoscutellar sulcus between notauli: smaller than those along margin of axilla. Sculpture of mesoscutellum: granulate. Shape of metascutellum: posterior margin rounded, approximately 3.0× wider than long. Sculpture of metascutellum in female: smooth with a longitudinal, median carina. Dorsal propodeum in female: not excavate medially, lateral propodeal carinae meeting anteromedially. Sculpture of dorsal propodeum in female: rugose; smooth to rugulose. Median keels on propodeum in female: absent. Mesopleural carina: present. Sculpture of mesepisternum below mesopleural depression: smooth. Sculpture of ventral metapleural area: largely smooth, rugose ventrally. Color of legs: orange yellow. Sculpture of hind coxa: smooth.

Color of fore wing: hyaline. Rs+M: nebulose, weakly pigmented. Setae on R: long, erect, surpassing the margin of the wing. Length of R: distinctly shorter than r-rs. Length of R1: approximately as long as 2.0× length of r-rs.

Color of metasoma in female: variably orange to pale brown. Horn on T1 in female: absent. Sculpture of posterior margin of T1 in female: longitudinally striate throughout; striate rugose. Development of longitudinal striae on T2 in female: reaching the middle of T2 medially; present on the anterior margin of T2. Sculpture of T3: smooth. Shape of T6 in female: short, wider than long. Sculpture of S3: largely smooth with sparse and fine punctures.

**Figures 226–231.**
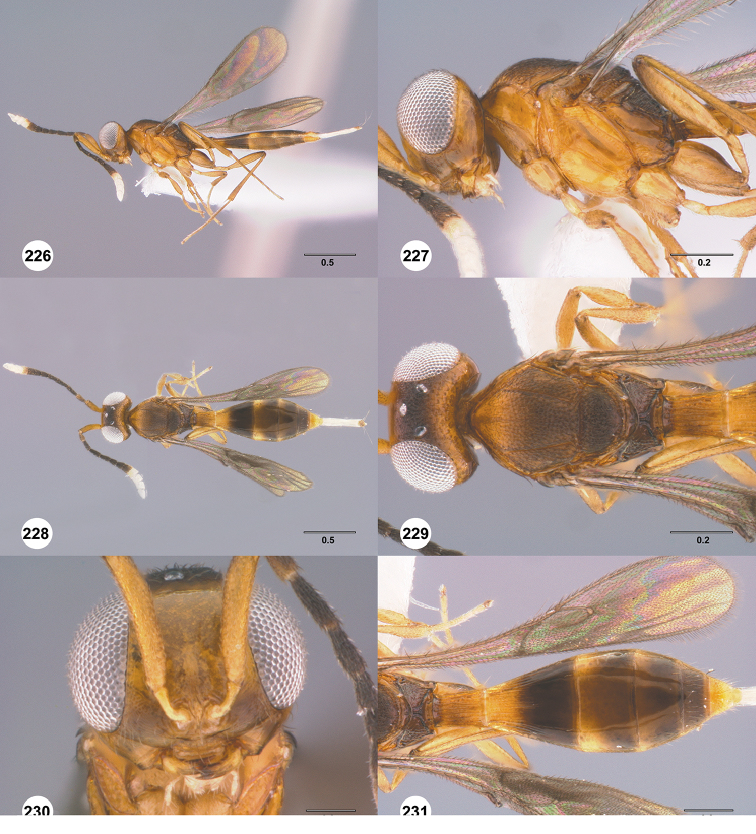
*Calliscelio ruga* sp. n., female, holotype (OSUC 553881). **226** Lateral habitus **227** Head and mesosoma, lateral view **228** Dorsal habitus **229** Head and mesosoma, dorsal view **230** Head, anterior view **231** Metasoma, dorsal view. Scale bars in millimeters.

#### Diagnosis.

This species is most similar to *Calliscelio extenuatus* and *Calliscelio suni* in size and habitus. It can be separated from *Calliscelio extenuatus* by the interrupted occipital carina and the elongate A6, and from *Calliscelio suni* by the hairy compound eyes.

#### Etymology.

The epithet is used as a noun in apposition derived from the Latin word for wrinkle, in reference to the rugose posterior vertex.

#### Link to distribution map.

[http://hol.osu.edu/map-full.html?id=385082]

#### Material examined.

Holotype, female: **PANAMA**: Chiriquí Prov., 1220m, 08°39'N, 82°12'W, La Suiza Farm, 16.VI.2000, flight intercept trap, H. Howden, OSUC 553881 (deposited in CNCI). Paratypes: (31 females) **ECUADOR**: 1 female, OSUC 553619 (CNCI). **PANAMA**: 29 females, OSUC 553758–553760, 553764, 553793, 553800–553803, 553825, 553827, 553829–553830, 553832, 553845, 553847, 553878–553880, 553882–553889, 553936–553937 (CNCI). **PUERTO RICO**: 1 female, OSUC 534561 (CNCI).

### Calliscelio rugicoxa

Taxon classificationAnimaliaHymenopteraPlatygastridae

Chen & Masner sp. n.

http://zoobank.org/ED46B60A-A31E-4165-AD10-C93BFA562781

http://bioguid.osu.edu/xbiod_concepts/362053

Figures 232–237

#### Description.

Body length of female: 3.16 mm (n=1). Color of head: pale brown. Color of antennal clava (A7–A12): black. Shape of head: subglobose. Central keel of frons: present. Setation of upper frons: with dense, short setae. IOS/EH: IOS distinctly less than EH. Sculpture of ventrolateral frons: granulate to finely punctate. Sculpture of frons below median ocellus: granulate. Sculpture of posterior vertex: granulate. Hyperoccipital carina: absent. Occipital carina medially: complete, strongly crenulate throughout. Length of OOL: less than 0.5× ocellar diameter. Sculpture of postgena behind outer orbit: granulate. Ocular setae: dense, long. A4 in female: as long as A3. A5 in female: shorter than A3, distinctly longer than wide. Shape of female A6: distinctly longer than wide.

Color of mesosoma in female: pale brown. Sculpture of dorsal pronotal area: rugose. Sculpture of lateral pronotal area: smooth dorsally, rugulose ventrally. Sculpture of netrion: rugose. Notaulus: percurrent or nearly so. Sculpture of mesoscutum: rugose. Shape of mesoscutellum: semiellipsoidal. Foveolae of scutoscutellar sulcus between notauli: as large as those along margin of axilla. Sculpture of mesoscutellum: densely punctate. Shape of metascutellum: posterior margin straight, approximately 4.0× wider than long. Sculpture of metascutellum in female: smooth with a longitudinal, median carina. Dorsal propodeum in female: not excavate medially, lateral propodeal carinae meeting anteromedially. Sculpture of dorsal propodeum in female: rugose. Median keels on propodeum in female: present. Mesopleural carina: present. Sculpture of mesepisternum below mesopleural depression: rugose anteriorly, smooth posteriorly. Sculpture of ventral metapleural area: rugose. Color of legs: pale brown. Sculpture of hind coxa: rugose.

Color of fore wing: hyaline. Rs+M: nebulose, weakly pigmented. Setae on R: long, erect, surpassing the margin of the wing. Length of R: distinctly shorter than r-rs. Length of R1: greater than 3.0× length of r-rs.

Color of metasoma in female: variably yellow to pale brown. Horn on T1 in female: weakly developed. Sculpture of T1 horn dorsally: rugose. Sculpture of posterior margin of T1 in female: striate rugose. Development of longitudinal striae on T2 in female: reaching posterior margin of T2. Sculpture of T3: largely smooth with submedian longitudinal striae. Shape of T6 in female: distinctly elongate, 2.0× longer than wide. Sculpture of S3: smooth.

**Figures 232–237.**
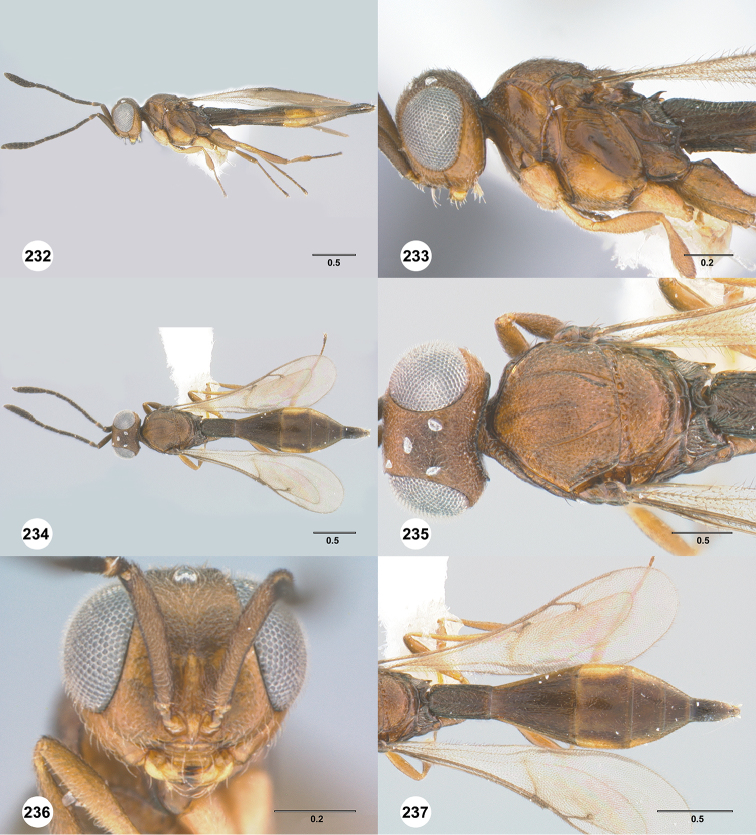
*Calliscelio rugicoxa* sp. n., female, holotype (OSUC 458325). **232** Lateral habitus **233** Head and mesosoma, lateral view **234** Dorsal habitus **235** Head and mesosoma, dorsal view **236** Head, anterior view **237** Metasoma, dorsal view. Scale bars in millimeters.

#### Diagnosis.

This species is most similar to *Calliscelio bidens* but can be distinguished by its rugose hind coxa and rugose T1 horn in the female.

#### Etymology.

The epithet refers to the rugose coxa in this species and is intended to be used as a noun in apposition.

#### Link to distribution map.

[http://hol.osu.edu/map-full.html?id=362053]

#### Material examined.

Holotype, female: **COLOMBIA**: Valle del Cauca Dept., 650m, 03°26'N, 76°48'W, Farallones de Cali Natural National Park, 8.V–19.VI.2001, Malaise trap, S. Sarria, OSUC 458325 (deposited in CNCI).

#### Comments.

We generally avoided describing two or more new species based on single specimens when they were collected at the same locality and time, which is the case for *Calliscelio rugicoxa* and *Calliscelio bidens*, but these two species are easily distinguished from each other (see diagnoses of the two species), and we are convinced they are two different species.

### Calliscelio sfina

Taxon classificationAnimaliaHymenopteraPlatygastridae

Chen & Johnson sp. n.

http://zoobank.org/17D52C03-F10C-4F1C-A73C-0C1C7FE5684C

http://bioguid.osu.edu/xbiod_concepts/363178

Figures 238–243

#### Description.

Body length of female: 1.78–2.97 mm (n=20). Body length of male: 1.67–2.53 mm (n=20). Color of head: orange throughout. Color of antennal clava (A7–A12): dark brown to black. Shape of head: subglobose. Central keel of frons: absent. Setation of upper frons: with sparse, long setae. IOS/EH: IOS distinctly less than EH. Sculpture of ventrolateral frons: transversely rugulose to granulate. Sculpture of frons below median ocellus: granulate. Sculpture of posterior vertex: granulate. Hyperoccipital carina: absent. Occipital carina medially: interrupted. Length of OOL: less than 0.5× ocellar diameter. Sculpture of postgena behind outer orbit: granulate. Ocular setae: absent. A4 in female: distinctly shorter than A3. A5 in female: shorter than A3, as long as wide. Shape of female A6: distinctly wider than long. Form of male antennal flagellomeres: filiform, A11 approximately 2.0× longer than wide. Length of A5 tyloid in male: greater than 0.5× length of A5.

Color of mesosoma in female: orange throughout. Color of mesosoma in male: orange throughout. Sculpture of dorsal pronotal area: rugose. Sculpture of lateral pronotal area: smooth throughout. Sculpture of netrion: smooth. Notaulus: percurrent or nearly so. Sculpture of mesoscutum: granulate. Shape of mesoscutellum: semiellipsoidal. Foveolae of scutoscutellar sulcus between notauli: smaller than those along margin of axilla. Sculpture of mesoscutellum: granulate. Shape of metascutellum: posterior margin rounded, approximately 2.5× wider than long. Sculpture of metascutellum in female: rugose. Sculpture of metascutellum in male: rugose. Dorsal propodeum in female: shallowly excavate medially, with lateral propodeal carinae widely separated. Sculpture of dorsal propodeum in female: rugose. Sculpture of dorsal propodeum in male: rugose. Median keels on propodeum in female: absent. Mesopleural carina: present. Sculpture of mesepisternum below mesopleural depression: smooth. Sculpture of ventral metapleural area: rugose. Color of legs: orange throughout; pale yellow throughout. Sculpture of hind coxa: smooth.

Color of fore wing: hyaline. Rs+M: nebulose, weakly pigmented; spectral. Setae on R: long, erect, surpassing the margin of the wing. Length of R: distinctly shorter than r-rs. Length of R1: approximately as long as 2.0× length of r-rs.

Color of metasoma in female: orange throughout; yellow with variable pale brown patches. Color of metasoma in male: orange throughout; variably orange to pale brown. Horn on T1 in female: present as a small bulge. Sculpture of T1 horn dorsally: with V-shaped striae. Sculpture of posterior margin of T1 in female: longitudinally striate throughout. Sculpture of T1 in male: longitudinally striate. Development of longitudinal striae on T2 in female: reaching posterior margin of T2. Sculpture of T3: smooth medially, longitudinally striate laterally; longitudinally striate throughout. Shape of T6 in female: short, slightly longer than wide. Sculpture of S3: densely punctate medially, longitudinally striate laterally.

**Figures 238–243.**
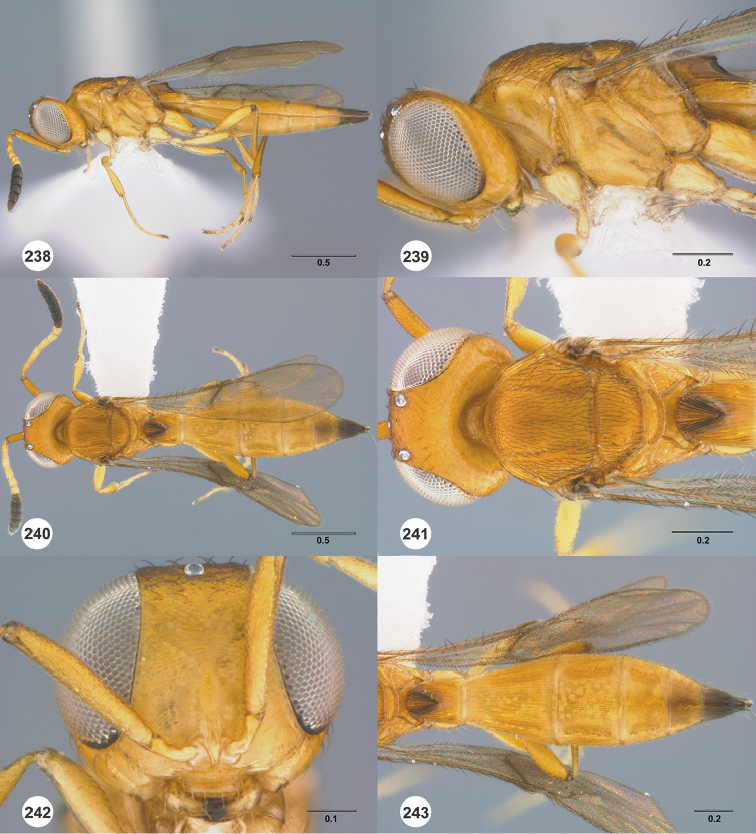
*Calliscelio sfina* sp. n., female, holotype (OSUC 376920). **238** Lateral habitus **239** Head and mesosoma, lateral view **240** Dorsal habitus **241** Head and mesosoma, dorsal view **242** Head, anterior view **243** Metasoma, dorsal view. Scale bars in millimeters.

#### Diagnosis.

This species is most similar to *Calliscelio carinigena* and *Calliscelio crater* in color, size and habitus but it can be distinguished by the absence of the hyperoccipital carina (hyperoccipital carina are absent in *Calliscelio carinigena* and *Calliscelio crater*).

#### Etymology.

The specific epithet is Greek for wedge and should be treated as a noun in apposition. It refers to the “wedge-like” horn on T1 in the female.

#### Link to distribution map.

[http://hol.osu.edu/map-full.html?id=363178]

#### Material examined.

Holotype, female: **BRAZIL**: ES, Duas Bocas Biological Reserve, pt.8, 20°16'21"S 40°28'40"W, Cariacica, 30.IV–1.V.2005, yellow pan trap, A. P. Aguiar et al., OSUC 376920 (deposited in MZSP). Paratypes: (194 females, 236 males) **BOLIVIA**: 2 females, OSUC 534160, 534182 (CNCI). **BRAZIL**: 172 females, 226 males, OSUC 534518, 534525–534526, 557200, 557205–557207 (CNCI); OSUC 577145, 577296, 577299, 577301, 577303, 577369–577370, 577378 (MNHN); OSUC 127850, 150156, 150159, 150785, 150795–150796, 150799, 150803, 150836–150838, 150920, 150957–150958, 150960, 150963, 150980–150983, 150986–150987, 151646, 322521–322524, 322528, 322987, 322999–323000, 323100, 323261–323263, 323267–323268, 323272, 323280, 323282, 323287, 323292–323295, 323298, 323302, 323305–323307, 323309–323310, 323317, 323323, 323325, 323329–323331, 323337, 323513, 323518–323519, 323521, 323527–323528, 323531–323532, 323538, 323542, 323544, 323546–323547, 323549, 323551, 323558–323560, 323563, 323566, 323915–323917, 363868, 363881–363882, 363888, 366644, 366646, 366654–366655, 366658, 372529, 372535, 375247, 375251, 375253, 375263, 375269, 375289, 375291–375292, 375294, 375298, 375302, 376549, 376554, 376917–376919, 376943, 376955, 376962–376963, 381087–381088, 427461, 433802, 433804, 433808, 433810–433811, 433828, 433831, 463311–463312, 576981, 577014, 577032–577033, 577037–577038, 577060–577061, 577070, 577090, 577094, 577096, 577099, 577101, 577103–577108, 577116–577121, 577199–577203, 577220, 577224–577227 (MZSP); OSUC 110064, 111443, 111648, 11923, 131098, 131107, 131280, 131320, 131673, 132179, 136374, 136540, 136832, 136847, 136857, 136867, 136882, 136934, 136937, 137137, 137182, 137237, 137377, 137430, 137433, 137583, 137599, 137698, 137708, 137885, 138539, 138781, 138784, 147789, 147791–147792, 151400, 151551, 151563, 235891, 235955, 235957–235959, 236824, 236826–236827, 237004, 322553, 323515, 323517, 347615, 357224–357225, 357246, 357255–357256, 357271, 357314, 357321–357322, 357324, 358792, 358940, 358975–358978, 358985–358986, 362661–362664, 362678, 366001, 367437–367438, 367442–367444, 375299, 376923, 378584, 378586–378590, 40192, 427453, 427456–427458, 433805, 433809, 433812–433817, 433825, 433829–433830, 433833–433836, 433838–433841, 433843, 433846, 433848, 435143, 436603, 477161–477162, 510908, 55933, 55938–55940, 55942, 55944, 55954, 576994–576999, 577211, 577272–577275, 577287, 583210–583212, 583226–583227, 583230–583232, 583236, 583290–583292, 583296, 61314–61315, 61317, 61322, 61335, 61341, 61357–61358, 61364, 61366, 61370, 61375–61376, 61380, 61383, 61407–61408, 61411, 61425, 61430, 61436, 61441, 61443, 61451–61452, 61460–61461, 61463, 61473, 61476, 61502, 61505, 61511, 61521–61522, 61527–61528, 61535, 61540, 61553, 61558, 63282, 63284, 63291, 63301, 63306, 63322, 63325, 63333, 63348, 63352, 63354, 63381, 63387, 63391–63392, 63401, 63419, 63422, 63428, 63446, 63451, 63491, 63516, 63521, 63565, 63579, 63615, 63663 (OSUC). **COLOMBIA**: 11 females, 2 males, OSUC 170496, 178186, 178198, 193393, 76994 (IAVH); OSUC 170505, 178020, 178168, 182297, 188679, 188946, 188949, 269347 (OSUC). **ECUADOR**: 2 females, 4 males, OSUC 458483 (CNCI); OSUC 534677, 534680–534681 (MAIC); OSUC 534669, 534676 (OSUC). **FRENCH GUIANA**: 1 female, OSUC 534552 (CNCI). **GUYANA**: 2 females, OSUC 534261–534262 (CNCI). PARAGUAY: 1 male, OSUC 278677 (OSUC). **PERU**: 3 females, 1 male, OSUC 323939, 323944, 323946, 323948 (OSUC). **TRINIDAD AND TOBAGO**: 1 female, OSUC 546026 (CNCI). **UNITED STATES**: 1 male, OSUC 61320 (OSUC). **VENEZUELA**: 1 male, OSUC 367474 (USNM).

### Calliscelio storea

Taxon classificationAnimaliaHymenopteraPlatygastridae

Chen & Johnson, sp .n.

http://zoobank.org/ED068E67-CE26-42A0-8DD1-26FD78DD0C72

http://bioguid.osu.edu/xbiod_concepts/384799

**Figures 244–249.**
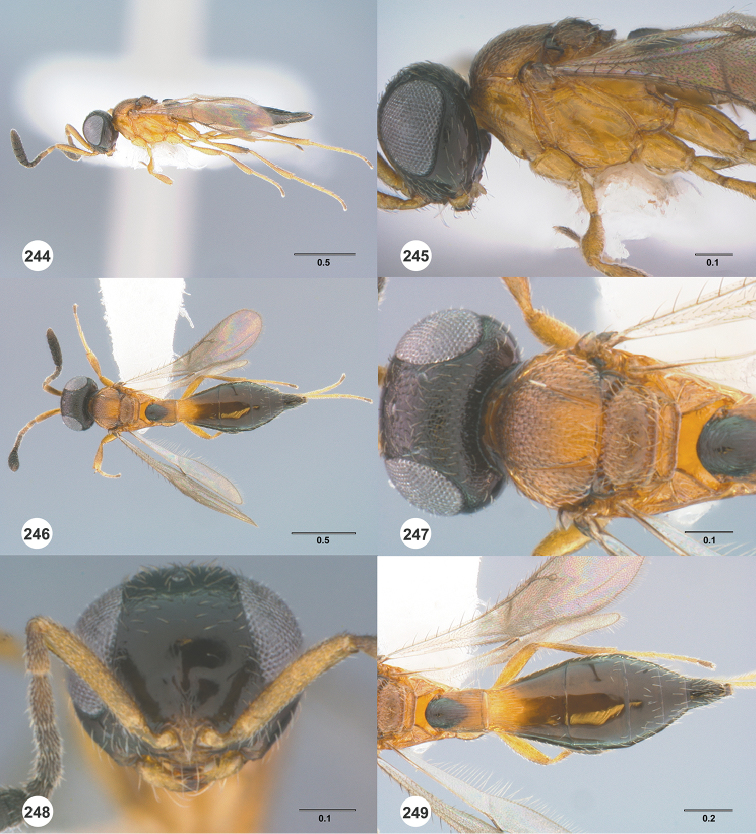
*Calliscelio storea* sp. n., female, holotype (OSUC 546117). **244** Lateral habitus **245** Head and mesosoma, lateral view **246** Dorsal habitus **247** Head and mesosoma, dorsal view **248** Head, anterior view **249** Metasoma, dorsal view. Scale bars in millimeters.

Figures 14 , 244–249

#### Description.

Body length of female: 1.76–2.15 mm (n=20). Color of head: black throughout; variably brown to black. Color of antennal clava (A7–A12): black. Shape of head: subglobose. Central keel of frons: absent. Setation of upper frons: with sparse, long setae. IOS/EH: IOS distinctly less than EH. Sculpture of ventrolateral frons: granulate. Sculpture of frons below median ocellus: granulate. Sculpture of posterior vertex: granulate. Hyperoccipital carina: absent. Occipital carina medially: complete, weakly crenulate throughout. Length of OOL: less than 0.5× ocellar diameter. Sculpture of postgena behind outer orbit: granulate. Ocular setae: sparse, short. A4 in female: distinctly shorter than A3. A5 in female: shorter than A3, as long as wide. Shape of female A6: as long as wide.

Color of mesosoma in female: orange throughout; variably orange to pale brown. Sculpture of dorsal pronotal area: rugose. Sculpture of lateral pronotal area: smooth anteriorly, granulate posteriorly. Sculpture of netrion: rugose. Notaulus: percurrent or nearly so. Sculpture of mesoscutum: granulate. Shape of mesoscutellum: semiellipsoidal. Foveolae of scutoscutellar sulcus between notauli: smaller than those along margin of axilla. Sculpture of mesoscutellum: granulate. Shape of metascutellum: posterior margin straight, approximately 4.0× wider than long. Sculpture of metascutellum in female: rugose. Dorsal propodeum in female: shallowly excavate medially, with lateral propodeal carinae widely separated. Sculpture of dorsal propodeum in female: rugose. Median keels on propodeum in female: absent. Mesopleural carina: present. Sculpture of mesepisternum below mesopleural depression: largely smooth with a row of foveae along mesopleural carina. Sculpture of ventral metapleural area: largely smooth, rugose ventrally. Color of legs: orange throughout. Sculpture of hind coxa: smooth.

Color of fore wing: hyaline. Rs+M: spectral. Setae on R: long, erect, surpassing the margin of the wing. Length of R: distinctly shorter than r-rs. Length of R1: approximately as long as 2.0× length of r-rs.

Color of metasoma in female: variably orange to pale brown. Horn on T1 in female: present as a small bulge. Sculpture of T1 horn dorsally: rugulose. Sculpture of posterior margin of T1 in female: longitudinally striate throughout. Development of longitudinal striae on T2 in female: present on the anterior margin of T2. Sculpture of T3: smooth. Shape of T6 in female: short, slightly longer than wide. Sculpture of S3: smooth.

#### Diagnosis.

This species is most similar to *Calliscelio bisulcatus* and *Calliscelio virga* in the hairy compound eyes, size and habitus. It can be separated from *Calliscelio bisulcatus* by the granulate mesoscutellum and the broad and short metascutellum, and from *Calliscelio virga* by the rugose metascutellum and the rugulose T1 horn in the female.

#### Etymology.

The epithet is used as a noun in apposition derived from the Latin word for mat or rug, in reference to the rugulose T1 horn in female.

#### Link to distribution map.

[http://hol.osu.edu/map-full.html?id=384799]

#### Material examined.

Holotype, female: **FRENCH GUIANA**: Cayenne Arrond., 04°33.562'N, 52°12.425'W, Kaw Mountains, 3.III–27.III.2007, Malaise trap, K. Sarv, OSUC 546117 (deposited in CNCI). Paratypes: (30 females) **BOLIVIA**: 1 female, OSUC 534184 (CNCI). **BRAZIL**: 4 females, OSUC 534619–534620 (CNCI); OSUC 12187, 374720 (OSUC). **ECUADOR**: 11 females, OSUC 458517, 458520, 458524–458527, 534244, 553400, 553623, 553648, 553716 (CNCI). **FRENCH GUIANA**: 3 females, OSUC 546118, 546120, 546123 (CNCI). **MEXICO**: 1 female, OSUC 534622 (CNCI). **SURINAME**: 1 female, OSUC 534580 (CNCI). **VENEZUELA**: 9 females, OSUC 545882–545883, 545885–545886, 545889–545890, 545894, 545896, 545941 (CNCI).

### Calliscelio suni

Taxon classificationAnimaliaHymenopteraPlatygastridae

Chen & Johnson sp. n.

http://zoobank.org/7EAA3B0E-69F9-4155-AEE1-58042D054D4A

http://bioguid.osu.edu/xbiod_concepts/364057

Figures 250–255

#### Description.

Body length of female: 1.80–2.18 mm (n=20). Body length of male: 1.70–2.16 mm (n=20). Color of head: brown throughout; yellow throughout; orange throughout. Color of antennal clava (A7–A12): A7–A9 brown, A10–A12 white; A7–A9 brown, A10–A12 yellow; brown. Shape of head: subglobose. Central keel of frons: absent. Setation of upper frons: with sparse, short setae. IOS/EH: IOS distinctly less than EH. Sculpture of ventrolateral frons: smooth. Sculpture of frons below median ocellus: smooth. Sculpture of posterior vertex: smooth; granulate; transversely striate; granulate to rugulose above hyperoccipital carina, smooth below. Hyperoccipital carina: absent; present. Occipital carina medially: interrupted. Length of OOL: greater than 0.5× ocellar diameter. Sculpture of postgena behind outer orbit: smooth. Ocular setae: absent. A4 in female: as long as A3. A5 in female: shorter than A3, distinctly longer than wide. Shape of female A6: as long as wide. Form of male antennal flagellomeres: filiform, A11 approximately 4.0× longer than wide. Length of A5 tyloid in male: approximately 0.3× length of A5.

Color of mesosoma in female: orange throughout; yellow throughout. Color of mesosoma in male: orange throughout; variably orange to pale brown; yellow throughout. Sculpture of dorsal pronotal area: rugose. Sculpture of lateral pronotal area: smooth throughout. Sculpture of netrion: smooth. Notaulus: percurrent or nearly so. Sculpture of mesoscutum: smooth with sparse punctures; granulate. Shape of mesoscutellum: semiellipsoidal. Foveolae of scutoscutellar sulcus between notauli: smaller than those along margin of axilla. Sculpture of mesoscutellum: anterior half granulate, posterior half smooth. Shape of metascutellum: posterior marging rounded, approximately 3.0× wider than long. Sculpture of metascutellum in female: smooth; rugose. Sculpture of metascutellum in male: rugose. Dorsal propodeum in female: not excavate medially, lateral propodeal carinae meeting anteromedially. Sculpture of dorsal propodeum in female: rugose; smooth to rugulose. Sculpture of dorsal propodeum in male: rugose. Median keels on propodeum in female: absent. Mesopleural carina: absent. Sculpture of mesepisternum below mesopleural depression: smooth. Sculpture of ventral metapleural area: largely smooth, rugose ventrally. Color of legs: orange throughout; pale yellow throughout. Sculpture of hind coxa: smooth.

Color of fore wing: hyaline. Rs+M: nebulose, weakly pigmented. Setae on R: long, erect, surpassing the margin of the wing. Length of R: distinctly shorter than r-rs. Length of R1: approximately as long as 2.0× length of r-rs.

Color of metasoma in female: variably orange to pale brown; yellow throughout. Color of metasoma in male: variably orange to pale brown; brown throughout. Horn on T1 in female: absent. Sculpture of posterior margin of T1 in female: longitudinally striate throughout. Sculpture of T1 in male: longitudinally striate. Development of longitudinal striae on T2 in female: reaching the middle of T2 medially; present on the anterior margin of T2. Sculpture of T3: smooth. Shape of T6 in female: short, wider than long or as long as wide. Sculpture of S3: smooth.

**Figures 250–255.**
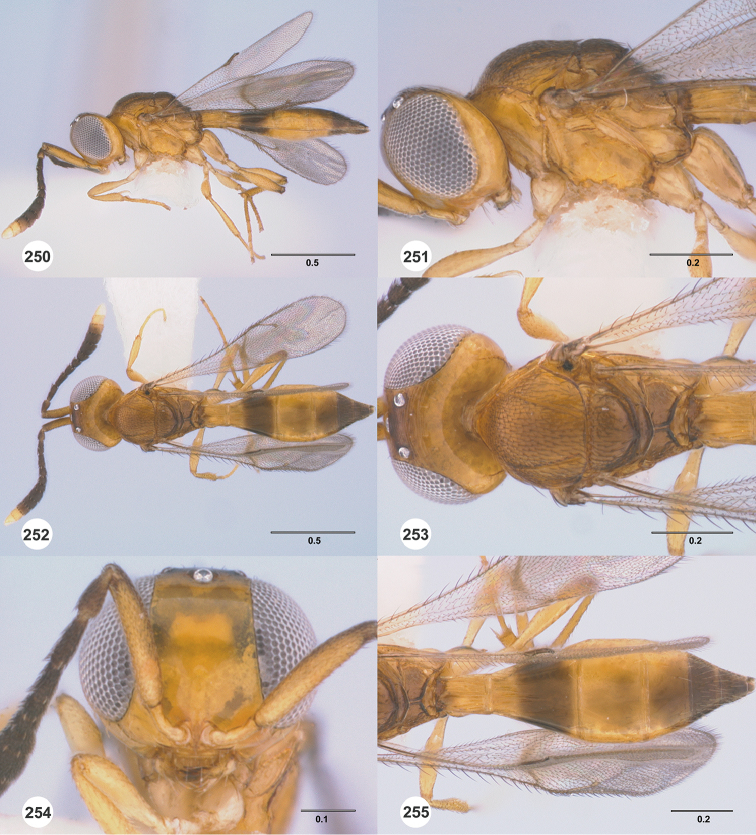
*Calliscelio suni* sp. n., female, holotype (OSUC 182752). **250** Lateral habitus **251** Head and mesosoma, lateral view **252** Dorsal habitus **253** Head and mesosoma, dorsal view **254** Head, anterior view **255** Metasoma, dorsal view. Scale bars in millimeters.

#### Diagnosis.

This species is similar to *Calliscelio absum*, *Calliscelio extenuatus*, *Calliscelio minutia* and *Calliscelio ruga* in size and habitus. It can be separated from *Calliscelio absum* by the percurrent notaulus, from *Calliscelio extenuatus* and *Calliscelio ruga* by the bare compound eyes and from *Calliscelio minutia* by the smooth frons and the entirely hyaline fore wing.

#### Etymology.

The epithet is derived from the name of a famous character in the Chinese classical novel Journey to the West, Sun Wukong, who knows 72 transformations. It refers the remarkable polymorphism of this species and should be treated as a genitive noun.

#### Link to distribution map.

[http://hol.osu.edu/map-full.html?id=364057]

#### Material examined.

Holotype, female: **COLOMBIA**: Chocó Dept., visitor's center, M.815, 2m, 06°01'N 77°20'W, Utría Natural National Park, 28.IX–15.X.2000, Malaise trap, J. Perez, OSUC 182752 (deposited in IAVH). Paratypes: (1756 females, 235 males) **BOLIVIA**: 19 females, OSUC 534033, 534045–534047, 534060–534062, 534147–534148, 534152–534155, 534158, 534162–534163, 534183, 534188–534189 (CNCI). **BRAZIL**: 1297 females, 41 males, OSUC 534534, 534542, 557198–557199, 557203, 557362, 557376–557377 (CNCI); OSUC 251769–251773, 252075–252081, 252083–252085, 252088, 252090–252091, 252093, 252095–252096, 252098–252104, 252107–252108, 252110, 252112–252114, 252116–252126, 252128–252129, 252131–252136, 252138, 252140, 252142–252144, 252146–252147, 322153, 322372–322376, 322378–322380, 322382, 322384, 322533, 322540, 322544, 322549, 322667–322669, 322672–322675, 323006, 323008, 323079, 323082, 323085, 323552, 323929, 323952–323957, 323960–323961, 326190, 326221, 326223, 326225, 326227, 326229, 326231–326234, 326236–326243, 326245–326247, 326396, 326398–326403, 326500, 326504, 326506–326520, 326534–326537, 326539–326542, 326544–326545, 337162–337166, 343651–343653, 346126, 346135, 346141, 346161, 346166–346167, 346176, 346185, 346855, 346872–346873, 346881, 346887, 346948, 346951, 347026, 347031, 347042, 347044, 347061, 347072, 347077, 347091, 347955–347957, 347959, 347962, 347971–347972, 347977–347978, 347980, 347986–347987, 347989, 347995, 347997–347999, 348002–348003, 348006, 348010, 348012, 348066–348067, 348069, 348076, 348101–348102, 348104, 348106, 348116, 348125, 348134, 348139, 348141, 348144, 348147–348148, 348150, 348153–348154, 348264, 348273, 348275, 348278, 348286, 348336, 348347–348348, 348486, 348503, 348506, 348510–348511, 348633, 348640, 348658, 348665, 352050, 352052–352054, 352057, 352060, 352069, 352080–352082, 352360, 352362–352364, 353037, 353040–353044, 353046, 353421–353425, 353428, 353555–353569, 354762, 354793, 354798–354799, 354806, 354813, 354827–354830, 354871–354872, 354893, 354897, 354904–354905, 355027–355030, 355035, 355037, 355039–355040, 355170–355177, 361081, 361098–361102, 361104, 361709–361711, 362559, 362587, 362590, 362596–362605, 363848, 363850–363855, 366691, 366693–366695, 366697, 366699–366700, 366702–366703, 366706–366710, 366713–366720, 366722–366724, 366781, 366783–366789, 366791, 366809–366812, 366937, 366940–366942, 366946–366952, 366967–366973, 366975–366982, 366986–366989, 368048–368059, 368061, 368411–368412, 368421, 368574–368579, 370900–370904, 370909–370914, 370916–370919, 370922, 370925–370928, 371786, 371815–371816, 371831–371832, 371834–371835, 371853–371854, 372545–372548, 374556, 374558–374559, 374562, 374568–374574, 374576–374585, 374588–374590, 374594, 374622, 374631–374636, 374715–374716, 374733–374736, 374738, 374743–374748, 376485, 376487, 376489–376491, 376493–376497, 376501–376504, 376506–376508, 376510–376517, 376525–376526, 376529, 376532–376537, 376539, 376543–376544, 376546–376548, 376930, 376935, 376941–376942, 376967–376975, 376977, 376980, 376983–376984, 377492–377493, 378017–378018, 378020, 378023–378025, 378027–378031, 378036–378040, 378042–378044, 378046, 378048–378051, 378053, 378056–378059, 378067, 378071, 378073–378079, 378081–378084, 378086–378088, 378601–378602, 378964–378966, 378968, 378970–378971, 378974, 378978, 378980–378981, 380190–380192 (MZSP); OSUC 235892, 283519–283520, 322383, 322534, 323962–323963, 323965–323969, 323971, 323975–323982, 343650, 343654–343671, 343675–343676, 344391, 344393, 344406, 344411, 344415, 344477, 344486, 344493–344494, 344499–344501, 344511, 344516, 344518, 344526–344528, 344533–344535, 344545, 345071, 345073, 345075, 345077, 345081, 345083, 345086–345088, 345090, 345094, 345109, 345117–345118, 345124, 345213, 345217, 345225–345226, 345232, 345250, 345252, 345254, 345257, 345302, 345304, 345310, 345320, 345324, 345329, 345334, 345347, 345421, 345425, 345434, 345438, 345441, 345458, 345467, 345476, 345487, 345536, 345540–345543, 345549, 345553, 345561, 345593, 345859–345860, 345871, 346452–346454, 346699, 346719, 346724, 346727, 346736–346737, 346739, 346747, 346780–346781, 346791, 346803, 346807, 346809, 346812–346813, 346820, 346823, 346825–346827, 346994, 346998, 347001–347002, 347008, 347010, 347020, 347203, 347209, 347219, 347225, 347228, 347232, 347235, 347239, 347241–347243, 347255–347256, 347269, 347272, 347288, 347388–347389, 347391, 347393, 347405, 347410, 347413, 347425, 347428–347429, 347433, 347439, 347448–347449, 347473–347474, 347480, 347484–347485, 347494–347495, 347498, 347510, 347512, 347516, 347528–347529, 347531, 347534, 347536, 347538, 347540, 347550, 347552, 347554, 347648, 347651, 347661, 347675–347677, 347680, 347683, 347685, 347735–347736, 347739, 347741, 347752, 347755–347756, 347773–347776, 347842, 347847, 347854, 347876, 347879–347880, 347882–347884, 347886, 347890, 347895, 347898, 347900, 347904, 347910, 348155, 348160, 348958–348959, 348967, 348973, 349002, 349012, 349016, 349020, 349105, 349112–349118, 349120, 349123, 349127, 349141–349144, 349148, 349202–349204, 349207–349210, 349358–349365, 349369, 349371–349375, 349479–349482, 349484–349488, 349493, 349497, 349502, 349507–349508, 349514, 349516, 349638, 349642–349645, 349647–349648, 349650–349651, 349654, 349660–349661, 349664, 349666, 349669, 349673–349674, 349676, 349681, 349684, 349687, 349690, 349694, 349697, 349710, 349712–349713, 349722, 349755, 349757–349762, 349764–349766, 349768–349770, 349772, 349774–349775, 349777–349778, 351244, 351246, 351252, 351262, 351264, 351266, 351269, 351278–351279, 351287, 351294, 351298, 351307, 351313–351314, 351327–351328, 351330–351331, 351397–351398, 351400–351401, 351404–351405, 351408, 351423, 351450, 351453, 351459–351460, 351465, 351475, 351478–351479, 351499, 351502, 351504, 351523–351524, 351626–351627, 351629, 351674–351675, 351703, 351808, 351810–351814, 351816, 351820–351822, 351825, 351827–351828, 351830, 351834–351835, 351848, 351854, 351856, 351858–351861, 351865, 351896–351898, 351900–351902, 351905, 352064, 352070, 352072–352075, 352077, 352079, 352361, 352803–352805, 352807–352809, 352811, 352813, 353178–353180, 353182–353186, 353626, 353645–353647, 353658–353660, 353668–353669, 353708, 353722–353724, 353728–353730, 353733–353738, 353740–353744, 353747–353748, 353752, 353755–353756, 353934–353938, 353940, 353943–353944, 353961–353962, 353967, 354040, 354054, 354059, 354092, 354100, 354102, 354111, 354113, 354716–354717, 354720–354721, 354723–354724, 354747, 354749, 355318, 357018–357020, 357025, 357051, 357057–357059, 359048, 366534, 371787–371814, 371828–371830, 371838, 371845–371848, 373754–373756, 373758–373760, 373763–373769, 373771–373772, 373774–373778, 373780–373783, 375303–375307, 375309, 375311–375314, 375317, 376052–376056, 376058–376062, 376522–376523, 377498–377503, 378016, 378019, 378021–378022, 378026, 378032, 378034, 378045, 378047, 378060–378062, 378064, 378066, 378068, 378070, 378072, 378593–378594, 378596, 378598–378600, 381086, 427455, 495357–495383, 534646, 534648, 534650, 534692–534694, 55907–55908, 55948, 577000–577004, 577017, 577125–577127, 577129–577133, 577135–577136, 577151–577153, 577158, 577160, 577163–577169, 577171–577172, 61523, 63534 (OSUC). **COLOMBIA**: 162 females, 143 males, OSUC 557414–557419, 557428–557443, 557445–557457, 557473–557475, 557477, 557480–557487, 557489–557496, 557498–557501, 557503–557509, 557511–557514, 557516–557517, 557519–557525, 557527–557534, 557552, 557557, 557565, 557604–557607, 557613, 557617–557618, 557642–557644 (CNCI); OSUC 144159–144163, 144166, 152141, 162504, 162507, 162591, 162602, 162620, 166587, 178016–178017, 178090, 178093, 178160, 178178–178180, 182595, 188680, 188683, 188686, 188937, 189185, 189203, 189210, 189221, 189295–189296, 191097, 191109–191111, 191146–191147, 192357, 193292, 193295–193296, 193347–193348, 193401, 193425, 193543, 193548–193549, 193564, 193595, 193698–193699, 193845, 193861–193862, 193865, 193875, 193877–193878, 193938, 202076–202077, 202080, 231810, 231824, 231827, 232299, 232301, 249900–249901, 253459, 253462, 259752-259753, 259761, 259765, 262545, 262601, 262606–262608, 262941, 262948, 262953–262954, 267808, 268909–268910, 269214–269215, 269218–269220, 269222, 272081, 272089, 273458, 275810, 276045, 276185–276186, 276243, 279661, 279841, 279905, 279913, 279918, 279924–279925, 279930, 280113, 280180, 280202, 363598, 372636, 377412–377414, 377417, 76993, 76995 (IAVH); OSUC 143971, 143974, 182487, 182741, 182757–182759, 188550, 188625, 188677, 188940, 188942–188943, 188960–188961, 189213, 189215, 189218–189220, 189266, 189269, 189274, 191107, 191151, 191182, 191200, 191298, 191304, 191310, 191314, 191317, 191364, 191367, 191372, 191375, 191385, 191388–191389, 193142, 193145, 193158–193159, 193177, 193194, 193800, 193813, 193846, 253460, 259756, 267809, 269213, 269221, 269223, 269352–269353, 269357, 269430, 269432, 269436, 269440–269443, 269481, 269937–269940, 274570, 274966, 274972, 275026, 279906–279907, 279910, 279917, 280196, 77001, 77005, 77008–77009, 77011 (OSUC). **COSTA RICA**: 20 females, 1 male, OSUC 532461, 532476, 532495, 532505, 532556, 532575, 532614, 532695–532696, 532709, 532723, 532728, 532730, 532753, 532773, 532786–532787, 532789, 532796, 532831, 532833 (CNCI). **ECUADOR**: 98 females, 14 males, OSUC 458486, 458489–458490, 458504, 458507, 458512–458513, 458534, 458545, 534226, 534248, 553233–553236, 553238–553241, 553244–553245, 553248–553254, 553256–553261, 553351–553353, 553355–553365, 553378–553380, 553388, 553402, 553414, 553418–553426, 553501, 553543, 553550, 553552, 553554–553556, 553560, 553572, 553575–553578, 553586–553589, 553603–553604, 553608–553616, 553654–553655, 553684, 553711, 553713–553715, 553719–553725, 553733–553735, 553745, 577336 (CNCI); OSUC 401740 (MZLU); OSUC 534662 (OSUC). FRENCH GUIANA: 14 females, 2 males, OSUC 458385–458387, 458390, 458409, 458414–458416, 458422, 458426, 458428–458429, 458471, 534551, 546109, 546130 (CNCI). **GUYANA**: 9 females, OSUC 534264–534265, 534267–534273 (CNCI). **MEXICO**: 1 female, OSUC 557254 (CNCI). PANAMA: 78 females, 17 males, OSUC 534069–534081, 534087, 534090, 534092–534096, 534098, 534103–534106, 553750–553751, 553755, 553776–553781, 553785–553792, 553794–553799, 553807, 553811–553813, 553817–553818, 553821, 553824, 553828, 553831, 553833–553835, 553837–553842, 553848–553858, 553890–553891, 553900–553903, 553906, 553908–553909, 553916–553918, 553920–553921, 553923, 553929, 553950 (CNCI). **PERU**: 45 females, 8 males, OSUC 534388, 534407–534412, 534427–534428, 553969, 553971, 553973–553977, 553979–553985, 553987, 553995–553999, 554001, 554003, 554011–554015, 554017–554020, 554027, 554029, 554031, 554052 (CNCI); OSUC 199544 (FSCA); OSUC 570541–570544 (OSUC); OSUC 228072–228073, 231995–231996 (USNM). **PUERTO RICO**: 4 females, 2 males, OSUC 343115–343119, 343124 (OSUC). **TRINIDAD AND TOBAGO**: 5 females, OSUC 534600, 534602–534604, 546001 (CNCI). **UNITED STATES**: 3 females, 6 males, OSUC 374705–374706, 374708–374714 (OSUC). **VENEZUELA**: 1 female, 1 male, OSUC 532725 (CNCI); OSUC 367473 (USNM).

#### Comments.

This species is well supported by many characters, although the sculpture of the posterior vertex and the color of the female antennal clava is extremely variable. The posterior vertex varies from smooth to transversely striate. The hyperoccipital carina also can be present or absent. The female antennal clava varies from entirely brown to having the last two or three segments white. These variations are gradual among specimens. Therefore we consider them as intraspecific rather than interspecific.

### Calliscelio telum

Taxon classificationAnimaliaHymenopteraPlatygastridae

Chen & Johnson sp. n.

http://zoobank.org/390805B1-95C4-48E8-8A4A-AAC99C389894

http://bioguid.osu.edu/xbiod_concepts/364059

Figures 256–261

#### Description.

Body length of female: 2.00–2.89 mm (n=20). Body length of male: 1.99–2.42 mm (n=9). Color of head: orange to pale brown; variably brown to black. Color of antennal clava (A7–A12): dark brown to black. Shape of head: subglobose. Central keel of frons: absent. Setation of upper frons: with dense, long setae. IOS/EH: IOS distinctly less than EH. Sculpture of ventrolateral frons: granulate. Sculpture of frons below median ocellus: granulate. Sculpture of posterior vertex: granulate. Hyperoccipital carina: absent. Occipital carina medially: complete, weakly crenulate throughout. Length of OOL: less than 0.5× ocellar diameter. Sculpture of postgena behind outer orbit: granulate. Ocular setae: dense, short. A4 in female: distinctly shorter than A3. A5 in female: shorter than A3, as long as wide. Shape of female A6: distinctly wider than long.

Color of mesosoma in female: orange throughout. Color of mesosoma in male: orange throughout. Sculpture of dorsal pronotal area: rugose. Sculpture of lateral pronotal area: smooth anteriorly, granulate posteriorly. Sculpture of netrion: smooth. Notaulus: percurrent or nearly so. Sculpture of mesoscutum: granulate. Shape of mesoscutellum: transverse. Foveolae of scutoscutellar sulcus between notauli: as large as those along margin of axilla. Sculpture of mesoscutellum: granulate. Shape of metascutellum: posterior margin straight, approximately 4.0× wider than long. Sculpture of metascutellum in female: granulate. Sculpture of metascutellum in male: rugose. Dorsal propodeum in female: deeply excavate medially, with lateral propodeal carinae widely separated, running subparallel to accommodate T1 horn. Sculpture of dorsal propodeum in female: rugose. Sculpture of dorsal propodeum in male: rugose with one or two longitudinal keels lateral median keel. Median keels on propodeum in female: absent. Mesopleural carina: present. Sculpture of mesepisternum below mesopleural depression: smooth. Sculpture of ventral metapleural area: largely smooth, rugose ventrally. Color of legs: orange yellow. Sculpture of hind coxa: smooth. Form of male antennal flagellomeres: filiform, approximately 3.0× greater than width. Length of A5 tyloid in male: longer than 0.5× length of A5.

Color of fore wing: hyaline. Rs+M: spectral. Setae on R: long, erect, surpassing the margin of the wing. Length of R: distinctly shorter than r-rs. Length of R1: approximately as long as 2.0× length of r-rs.

Color of metasoma in female: orange throughout; variably orange to pale brown. Color of metasoma in male: T5–T7 brown to black, otherwise orange. Horn on T1 in female: large and distinct. Sculpture of T1 horn dorsally: rugulose. Sculpture of posterior margin of T1 in female: longitudinally striate throughout. Sculpture of T1 in male: longitudinally striate. Development of longitudinal striae on T2 in female: reaching posterior margin of T2. Sculpture of T3: smooth; smooth with longitudinal submedian striae. Shape of T6 in female: distinctly elongate, at least 2.0× longer than wide. Sculpture of S3: smooth.

**Figures 256–261.**
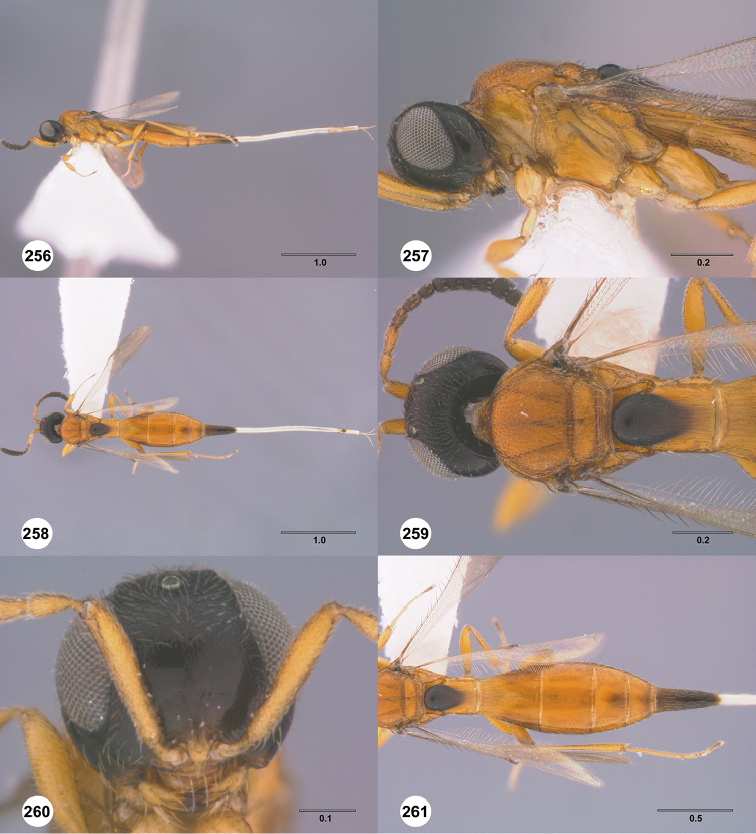
*Calliscelio telum* sp. n., female, holotype (OSUC 276704). **256** Lateral habitus **257** Head and mesosoma, lateral view **258** Dorsal habitus **259** Head and mesosoma, dorsal view **260** Head, anterior view **261** Metasoma, dorsal view. Scale bars in millimeters.

#### Diagnosis.

This species is most similar to *Calliscelio granulatus* in its hairy compound eyes and habitus but can be distinguished by the smooth netrion and rugulose T1 horn.

#### Etymology.

The epithet is used as a noun in apposition derived from the Latin word for sword, in reference to the elongate T6 in female.

#### Link to distribution map.

[http://hol.osu.edu/map-full.html?id=364059]

#### Material examined.

Holotype, female: **BRAZIL**: RO, Rancho Grande Farm, XI.1991, yellow pan trap, S. Passoa, OSUC 48550 (deposited in OSUC). Paratypes: (52 females, 9 males) **ARGENTINA**: 7 females, OSUC 534132–534136 (CNCI); OSUC 577260–577261 (UCRC). **BOLIVIA**: 3 females, OSUC 534031, 534180–534181 (CNCI). **BRAZIL**: 26 females, OSUC 557243, 557338 (CNCI); OSUC 111811, 11920–11922, 11924, 11926, 130907, 131117, 131476, 48560 (MZSP); OSUC 111725, 112704, 130635, 130718, 130750, 130752, 131118, 131188, 132434, 132437, 132572, 374723–374724, 48583 (OSUC). **PARAGUAY**: 12 females, 9 males, OSUC 534116 (CNCI); OSUC 276697, 276699, 276702, 322995, 322998, 323038, 323048, 323071 (MNHNPY); OSUC 276692–276695, 276698, 276700–276701, 276704, 363635, 434081, 583312–583313 (OSUC). **PERU**: 1 female, OSUC 323938 (OSUC). **URUGUAY**: 3 females, OSUC 534607, 534615–534616 (CNCI).

### Calliscelio torqueo

Taxon classificationAnimaliaHymenopteraPlatygastridae

Chen & Johnson sp. n.

http://zoobank.org/97273934-B929-447B-84BE-4E9F0BB09F55

http://bioguid.osu.edu/xbiod_concepts/384798

Figures 262–267

#### Description.

Body length of female: 1.27–2.54 mm (n=20). Body length of male: 1.39–2.10 mm (n=16). Color of head: black throughout; brown throughout; orange throughout. Color of antennal clava (A7–A12): A7–A10 dark brown, A11 and A12 white to pale yellow. Shape of head: subglobose. Central keel of frons: absent. Setation of upper frons: with sparse, short setae. IOS/EH: IOS distinctly less than EH. Sculpture of ventrolateral frons: smooth to rugulose. Sculpture of frons below median ocellus: smooth. Sculpture of posterior vertex: smooth. Hyperoccipital carina: absent. Occipital carina medially: interrupted. Length of OOL: greater than 0.5× ocellar diameter. Sculpture of postgena behind outer orbit: smooth. Ocular setae: absent. A4 in female: distinctly longer than A3. A5 in female: shorter than A3, distinctly longer than wide. Shape of female A6: distinctly longer than wide. Form of male antennal flagellomeres: filiform, A11 approximately 4.0× longer than wide. Length of A5 tyloid in male: approximately 0.3× length of A5.

Color of mesosoma in female: orange throughout; black throughout; variably orange to pale brown. Color of mesosoma in male: orange throughout; variably orange to pale brown; black throughout. Sculpture of dorsal pronotal area: smooth. Sculpture of lateral pronotal area: smooth throughout. Sculpture of netrion: smooth. Notaulus: percurrent or nearly so. Sculpture of mesoscutum: coriaceous; smooth throughout. Shape of mesoscutellum: semiellipsoidal. Foveolae of scutoscutellar sulcus between notauli: smaller than those along margin of axilla. Sculpture of mesoscutellum: smooth with sparse fine punctures. Shape of metascutellum: posterior margin somewhat rounded, approximately 2.5× wider than long. Sculpture of metascutellum in female: smooth. Sculpture of metascutellum in male: smooth. Dorsal propodeum in female: not excavate medially, lateral propodeal carinae meeting anteromedially. Sculpture of dorsal propodeum in female: rugose. Sculpture of dorsal propodeum in male: rugose. Median keels on propodeum in female: absent. Mesopleural carina: absent. Sculpture of mesepisternum below mesopleural depression: smooth. Sculpture of ventral metapleural area: smooth. Color of legs: fore coxa pale yellow, otherwise variably yellow to pale brown; coxae pale yellow, otherwise orange. Sculpture of hind coxa: smooth.

Color of fore wing: hyaline. Rs+M: nebulose, weakly pigmented. Setae on R: long, erect, surpassing the margin of the wing. Length of R: approximately as long as r-rs. Length of R1: greater than 3.0× length of r-rs.

Color of metasoma in female: orange throughout; variably orange to pale brown. Color of metasoma in male: variably orange to pale brown. Horn on T1 in female: weakly developed. Sculpture of T1 horn dorsally: smooth. Sculpture of posterior margin of T1 in female: longitudinally striate throughout. Sculpture of T1 in male: longitudinally striate. Development of longitudinal striae on T2 in female: present on anterior margin of T2 medially, reaching posterior margin of T2 laterally. Sculpture of T3: smooth with longitudinal submedian striae. Shape of T6 in female: short, slightly longer than wide. Sculpture of S3: smooth.

**Figures 262–267.**
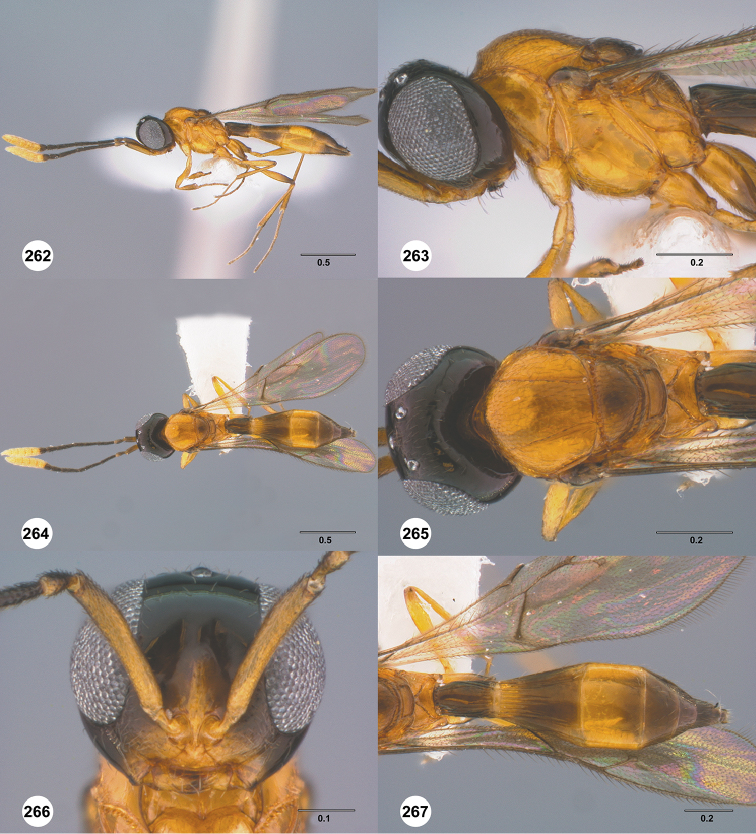
*Calliscelio torqueo* sp. n., female, holotype (OSUC 553598). **262** Lateral habitus **263** Head and mesosoma, lateral view **264** Dorsal habitus **265** Head and mesosoma, dorsal view **266** Head, anterior view **267** Metasoma, dorsal view. Scale bars in millimeters.

#### Diagnosis.

This species is most similar to *Calliscelio glaber* and *Calliscelio paraglaber* in the smooth head and metascutellum. It can be separated from *Calliscelio glaber* by its pigmented Rs+M and smooth small horn, from *Calliscelio paraglaber* by the absence of an occipital carina and T4 is distinctly longer than A3.

#### Etymology.

The epithet is used as a noun in apposition derived from the Latin word for torch, in reference to female antennal club color.

#### Link to distribution map.

[http://hol.osu.edu/map-full.html?id=384798]

#### Material examined.

Holotype, female: **ECUADOR**: Pichincha Prov., 1540m, 00°00'23"N 78°40'36"W, Nanegalito, 27.X–31.X.1999, flight intercept trap, Z. H. Falin, OSUC 553598 (deposited in CNCI). Paratypes: (218 females, 88 males) **COLOMBIA**: 36 females, 16 males, OSUC 557420–557421, 557471–557472, 557476, 557478–557479, 557488, 557497, 557502, 557510, 557515, 557518, 557526, 557566, 557608, 557612, 557614–557616 (CNCI); OSUC 188727–188728, 188731, 193127, 193692, 202074–202075, 202079, 202081, 202084, 269216, 279912, 279916, 279919–279923, 279927, 280183, 280203, 280206, 377411, 377415–377416, 377418–377422, 377425–377426 (OSUC). **COSTA RICA**: 120 females, 38 males, OSUC 232067, 232070, 532466–532467, 532471–532473, 532475, 532477–532478, 532496, 532498, 532500–532501, 532504, 532511–532515, 532520, 532522, 532531, 532534, 532536, 532538, 532540–532542, 532544–532548, 532550, 532555, 532557, 532561–532562, 532564, 532566, 532571, 532579, 532584, 532595–532596, 532609, 532615, 532629, 532642, 532645, 532652, 532654–532655, 532659, 532661–532662, 532671, 532674, 532683, 532690, 532703–532705, 532707, 532727, 532729, 532731, 532733, 532736, 532742–532745, 532747–532748, 532754–532755, 532758–532764, 532768, 532770, 532772, 532776, 532779–532780, 532782, 532793–532795, 532797–532801, 532803–532822, 532826–532830, 532832, 532835–532840, 532918, 532920–532923, 532926–532927, 532931–532934, 534139–534141, 534236 (CNCI); OSUC 244741, 245170, 245172, 245174, 245237–245238, 246300, 246354–246355, 358624, 374055 (OSUC). **ECUADOR**: 14 females, 8 males, OSUC 534252, 553354, 553475, 553489, 553495, 553531–553536, 553539, 553574, 553597, 553599, 553617, 553667, 553672–553674, 553708–553709 (CNCI). **PANAMA**: 25 females, 13 males, OSUC 534086, 534088–534089, 534091, 534097, 534099, 553748–553749, 553752–553754, 553761–553763, 553765–553766, 553772, 553783, 553804–553806, 553815, 553819, 553823, 553836, 553843–553844, 553846, 553859, 553875–553876, 553928, 553933–553934, 553941–553943, 553951 (CNCI). **VENEZUELA**: 23 females, 13 males, OSUC 545830, 545871–545872, 545939–545940, 545955, 545957, 545991–545993, 557657, 557701–557707, 557712 (CNCI); OSUC 146810, 334525, 334545, 334547, 46271, 46275, 46569, 48153, 48213, 48232, 48327, 48481, 48756, 48784, 48789, 63915, 79762 (OSUC).

### Calliscelio virga

Taxon classificationAnimaliaHymenopteraPlatygastridae

Chen & Johnson sp. n.

http://zoobank.org/D2DB314F-6D8F-44B6-BB3C-912BD3DC0029

http://bioguid.osu.edu/xbiod_concepts/364058

Figures 268–273

#### Description.

Body length of female: 1.68–2.10 mm (n=20). Body length of male: 1.64–2.07 mm (n=20). Color of head: brown throughout; variably brown to black. Color of antennal clava (A7–A12): black. Shape of head: subglobose. Central keel of frons: absent. Setation of upper frons: with sparse, long setae. IOS/EH: IOS slightly less than EH. Sculpture of ventrolateral frons: smooth to granulate. Sculpture of frons below median ocellus: largely smooth with sparse fine punctures. Sculpture of posterior vertex: granulate. Hyperoccipital carina: absent. Occipital carina medially: complete, weakly crenulate throughout. Length of OOL: less than 0.5× ocellar diameter. Sculpture of postgena behind outer orbit: largely smooth with small granulate area. Ocular setae: sparse, short. A4 in female: distinctly shorter than A3. A5 in female: shorter than A3, as long as wide. Shape of female A6: distinctly wider than long. Form of male antennal flagellomeres: filiform, A11 approximately 2.0× longer than wide. Length of A5 tyloid in male: greater than 0.5× length of A5.

Color of mesosoma in female: orange throughout; variably orange to pale brown. Color of mesosoma in male: variably orange to pale brown. Sculpture of dorsal pronotal area: rugose. Sculpture of lateral pronotal area: smooth throughout. Sculpture of netrion: rugose. Notaulus: percurrent or nearly so. Sculpture of mesoscutum: smooth with sparse punctures; coriaceous. Shape of mesoscutellum: semiellipsoidal. Foveolae of scutoscutellar sulcus between notauli: as large as those along margin of axilla. Sculpture of mesoscutellum: smooth with sparse fine punctures; granulate. Shape of metascutellum: posterior margin straight, approximately 4.0× wider than long. Sculpture of metascutellum in female: smooth. Sculpture of metascutellum in male: smooth. Dorsal propodeum in female: shallowly excavate medially, with lateral propodeal carinae widely separated. Sculpture of dorsal propodeum in female: rugulose. Sculpture of dorsal propodeum in male: rugose. Median keels on propodeum in female: absent. Mesopleural carina: absent. Sculpture of mesepisternum below mesopleural depression: smooth. Sculpture of ventral metapleural area: smooth. Color of legs: orange throughout. Sculpture of hind coxa: smooth.

Color of fore wing: hyaline. Rs+M: spectral. Setae on R: long, erect, surpassing the margin of the wing. Length of R: approximately as long as r-rs. Length of R1: approximately as long as r-rs.

Color of metasoma in female: orange throughout; variably orange to pale brown. Color of metasoma in male: variably orange to pale brown. Horn on T1 in female: present as a small bulge. Sculpture of T1 horn dorsally: smooth. Sculpture of posterior margin of T1 in female: longitudinally striate throughout. Sculpture of T1 in male: longitudinally striate. Development of longitudinal striae on T2 in female: present on the anterior margin of T2. Sculpture of T3: smooth. Shape of T6 in female: short, wider than long. Sculpture of S3: smooth.

**Figures 268–273.**
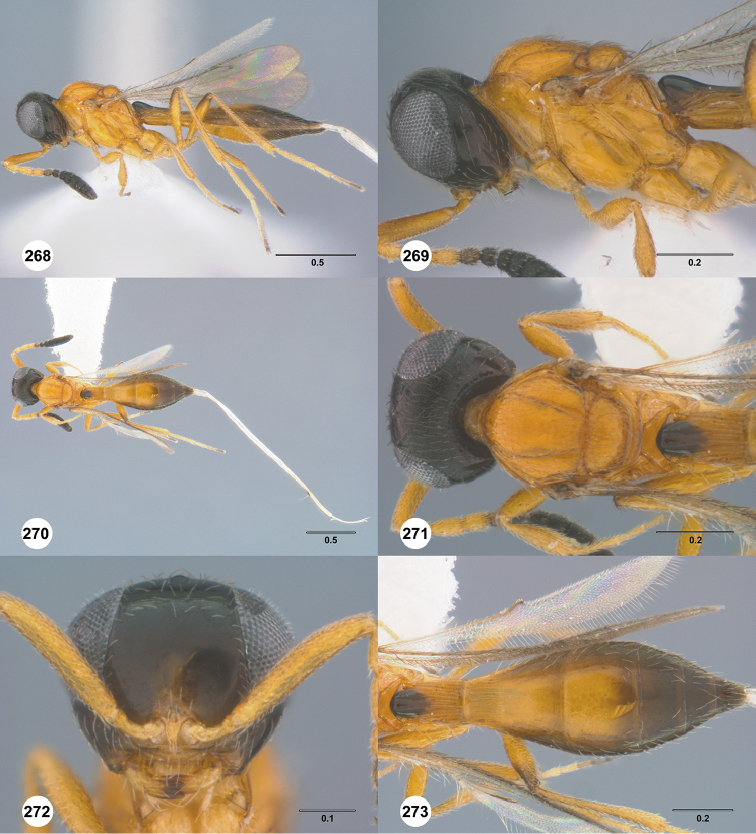
*Calliscelio virga* sp. n., female, holotype (OSUC 534034). **268** Lateral habitus **269** Head and mesosoma, lateral view **270** Dorsal habitus **271** Head and mesosoma, dorsal view **272** Head, anterior view **273** Metasoma, dorsal view. Scale bars in millimeters.

#### Diagnosis.

This species is most similar to *Calliscelio bisulcatus* and *Calliscelio storea* in the hairy compound eyes, size and habitus but can be easily recognized by its narrow and smooth metascutellum.

#### Etymology.

The specific epithet is Latin for rod or wand and should be treated as a noun in apposition. It refers to the narrow smooth metascutellum of this species.

#### Link to distribution map.

[http://hol.osu.edu/map-full.html?id=364058]

#### Material examined.

Holotype, female: **BOLIVIA**: Santa Cruz Dept., Andrés Ibáñez Prov., B-21, pools, 375m, 17°40'S, 63°27'W, El Hondo, 14.V–17.V.1997, yellow pan trap, L. Masner, OSUC 534034 (deposited in CNCI). Paratypes: (169 females, 98 males) **BRAZIL**: 15 females, OSUC 534523, 557298 (CNCI); OSUC 48514, 48520, 55945, 55947, 55952, 583247, 583250, 583252–583255, 583257–583258 (OSUC). **CANADA**: 8 males, OSUC 531726–531727, 531734–531737, 532035, 532077 (CNCI). **COLOMBIA**: 1 female, 1 male, OSUC 557585, 557623 (CNCI). **COSTA RICA**: 1 female, OSUC 532651 (CNCI). **DOMINICAN REPUBLIC**: 2 males, OSUC 534361, 534375 (CNCI). **ECUADOR**: 3 females, OSUC 458516, 458518, 553369 (CNCI). **GUATEMALA**: 1 male, OSUC 534434 (CNCI). **MEXICO**: 5 males, OSUC 534018–534020, 534477–534478 (CNCI). **PARAGUAY**: 3 females, 4 males, OSUC 150470, 534684, 577155, 577191–577192, 577195–577196 (OSUC). **SURINAME**: 4 females, OSUC 534568, 534572, 534587, 553621 (CNCI). **TRINIDAD AND TOBAGO**: 2 females, OSUC 546029, 546085 (CNCI). **UNITED STATES**: 138 females, 76 males, OSUC 531682, 531686, 531750, 531776–531780, 531786, 531792, 531918, 531940–531947, 532061, 532064, 532100, 532119, 532121, 532131–532177, 532180–532246, 532340–532390, 534347–534360 (CNCI); OSUC 142805, 142807, 142810, 207785, 236919, 272939–272940, 576980, 62904, 62907, 62927 (OSUC). **VENEZUELA**: 2 females, 1 male, OSUC 557676 (CNCI); OSUC 55924–55925 (OSUC).

## Supplementary Material

XML Treatment for Calliscelio

XML Treatment for Calliscelio absconditum

XML Treatment for Calliscelio absum

XML Treatment for Calliscelio alcoa

XML Treatment for Calliscelio amadoi

XML Treatment for Calliscelio armila

XML Treatment for Calliscelio bidens

XML Treatment for Calliscelio bisulcatus

XML Treatment for Calliscelio brachys

XML Treatment for Calliscelio brevinotaulus

XML Treatment for Calliscelio brevitas

XML Treatment for Calliscelio carinigena

XML Treatment for Calliscelio crater

XML Treatment for Calliscelio crena

XML Treatment for Calliscelio eboris

XML Treatment for Calliscelio elegans

XML Treatment for Calliscelio extenuatus

XML Treatment for Calliscelio flavicauda

XML Treatment for Calliscelio foveolatus

XML Treatment for Calliscelio gatineau

XML Treatment for Calliscelio glaber

XML Treatment for Calliscelio granulatus

XML Treatment for Calliscelio laticinctus

XML Treatment for Calliscelio latifrons

XML Treatment for Calliscelio levis

XML Treatment for Calliscelio longius

XML Treatment for Calliscelio magnificus

XML Treatment for Calliscelio migma

XML Treatment for Calliscelio minutia

XML Treatment for Calliscelio paraglaber

XML Treatment for Calliscelio pararemigio

XML Treatment for Calliscelio prolixus

XML Treatment for Calliscelio punctatifrons

XML Treatment for Calliscelio remigio

XML Treatment for Calliscelio rubriclavus

XML Treatment for Calliscelio ruga

XML Treatment for Calliscelio rugicoxa

XML Treatment for Calliscelio sfina

XML Treatment for Calliscelio storea

XML Treatment for Calliscelio suni

XML Treatment for Calliscelio telum

XML Treatment for Calliscelio torqueo

XML Treatment for Calliscelio virga

## Appendix 1

**Table:** URI Table matching terms and concepts used in this revision with the Hymenoptera Anatomy Ontology database.

	A1	http://purl.obolibrary.org/obo/HAO_0000908
	A2	http://purl.obolibrary.org/obo/HAO_0000706
	A3	http://purl.obolibrary.org/obo/HAO_0001148
	A7	http://purl.obolibrary.org/obo/HAO_0001885
	A12	http://purl.obolibrary.org/obo/HAO_0001884
	antenna	http://purl.obolibrary.org/obo/HAO_0000101
	antennomere	http://purl.obolibrary.org/obo/HAO_0000107
	area	http://purl.obolibrary.org/obo/HAO_0000146
	body	http://purl.obolibrary.org/obo/HAO_0000182
	carina	http://purl.obolibrary.org/obo/HAO_0000188
	central keel	http://purl.obolibrary.org/obo/HAO_0000109
cpa	cervical pronotal area	http://purl.obolibrary.org/obo/HAO_0000194
	clava	http://purl.obolibrary.org/obo/HAO_0000203
	clypeus	http://purl.obolibrary.org/obo/HAO_0000212
	compound eye	http://purl.obolibrary.org/obo/HAO_0000217
	coxa	http://purl.obolibrary.org/obo/HAO_0000228
	depression	http://purl.obolibrary.org/obo/HAO_0000241
dpa	dorsal pronotal area	http://purl.obolibrary.org/obo/HAO_0000267
	egg	http://purl.obolibrary.org/obo/HAO_0000286
	epomial carina	http://purl.obolibrary.org/obo/HAO_0000307
	eye	http://purl.obolibrary.org/obo/HAO_0000217
	femur	http://purl.obolibrary.org/obo/HAO_0000327
	fore wing	http://purl.obolibrary.org/obo/HAO_0000351
	frons	http://purl.obolibrary.org/obo/HAO_0001523
	gena	http://purl.obolibrary.org/obo/HAO_0000371
	head	http://purl.obolibrary.org/obo/HAO_0000397
	hind coxa	http://purl.obolibrary.org/obo/HAO_0000587
	hind tibia	http://purl.obolibrary.org/obo/HAO_0000631
	hind wing	http://purl.obolibrary.org/obo/HAO_0000400
	inner orbit	http://purl.obolibrary.org/obo/HAO_0000419
	interantennal process	http://purl.obolibrary.org/obo/HAO_0000422
	lateral lobe of mesoscutum	http://purl.obolibrary.org/obo/HAO_0000466
	lateral ocellus	http://purl.obolibrary.org/obo/HAO_0000481
LOL	lateral ocellar line	http://purl.obolibrary.org/obo/HAO_0000480
lpa	lateral pronotal area	http://purl.obolibrary.org/obo/HAO_0000483
	malar sulcus	http://purl.obolibrary.org/obo/HAO_0000504
	mandible	http://purl.obolibrary.org/obo/HAO_0000506
lpa	margin	http://purl.obolibrary.org/obo/HAO_0000510
	mesepisternum	http://purl.obolibrary.org/obo/HAO_0001872
med	mesopleural depression	http://purl.obolibrary.org/obo/HAO_0000326
	mesopleuron	http://purl.obolibrary.org/obo/HAO_0000566
	mesoscutellum	http://purl.obolibrary.org/obo/HAO_0000574
	mesoscutum	http://purl.obolibrary.org/obo/HAO_0001490
	mesosoma	http://purl.obolibrary.org/obo/HAO_0000576
	metapleuron	http://purl.obolibrary.org/obo/HAO_0000621
	metascutellum	http://purl.obolibrary.org/obo/HAO_0000625
	metasoma	http://purl.obolibrary.org/obo/HAO_0000626
	midlobe of mesoscutum	http://purl.obolibrary.org/obo/HAO_0000520
	netrion	http://purl.obolibrary.org/obo/HAO_0000644
	notauli (notaulus)	http://purl.obolibrary.org/obo/HAO_0000647
	occipital carina	http://purl.obolibrary.org/obo/HAO_0000653
	ocellus	http://purl.obolibrary.org/obo/HAO_0000661
ot	ocellar triangle	http://purl.obolibrary.org/obo/HAO_0000430
OOL	ocular ocellar line	http://purl.obolibrary.org/obo/HAO_0000662
	orbit	http://purl.obolibrary.org/obo/HAO_0000672
POL	posterior ocellar line	http://purl.obolibrary.org/obo/HAO_0000759
	process	http://purl.obolibrary.org/obo/HAO_0000822
	propodeum	http://purl.obolibrary.org/obo/HAO_0001248
	S1	http://purl.obolibrary.org/obo/HAO_0001997
	S2	http://purl.obolibrary.org/obo/HAO_0001829
	S3	http://purl.obolibrary.org/obo/HAO_0001831
	S4	http://purl.obolibrary.org/obo/HAO_0001832
	S5	http://purl.obolibrary.org/obo/HAO_0001833
	S6	http://purl.obolibrary.org/obo/HAO_0001834
	S7	http://purl.obolibrary.org/obo/HAO_0002185
	sculpture	http://purl.obolibrary.org/obo/HAO_0000913
	sternite	http://purl.obolibrary.org/obo/HAO_0001654
	sulcus	http://purl.obolibrary.org/obo/HAO_0000978
	T1	http://purl.obolibrary.org/obo/HAO_0000053
	T2	http://purl.obolibrary.org/obo/HAO_0000056
	T3	http://purl.obolibrary.org/obo/HAO_0000057
	T4	http://purl.obolibrary.org/obo/HAO_0000058
	T5	http://purl.obolibrary.org/obo/HAO_0000059
	T6	http://purl.obolibrary.org/obo/HAO_0000060
	T7	http://purl.obolibrary.org/obo/HAO_0000061
	tergite	http://purl.obolibrary.org/obo/HAO_0001783
	tibia	http://purl.obolibrary.org/obo/HAO_0001017
	tyloid	http://purl.obolibrary.org/obo/HAO_0001199
	vein	http://purl.obolibrary.org/obo/HAO_0001095
	vertex	http://purl.obolibrary.org/obo/HAO_0001077
	vertical epomial carina	http://purl.obolibrary.org/obo/HAO_0000307
	wing	http://purl.obolibrary.org/obo/HAO_0001089
